# Small-Molecule Activators of PRMT1: Discovery, SAR Analyses, and Proapoptotic Effects in Pancreatic Cancer Cells

**DOI:** 10.1021/acs.jmedchem.5c03281

**Published:** 2026-08-20

**Authors:** Sepideh Salehipour-Bavarsad, Christian Iking, Jonas Kammertöns, Caroline Bouchard, Marion Meixner, Michael Daude, Wieland Steinchen, Felix Terwesten, Johanna Senst, David Vonhören, Sinja Rakow, Anne Kagerhuber, Sona Martirosian, Katrin Roth, Daniel Hilger, Stefanie Dörr, Gina Bach, Van Tuan Trinh, Anett Hauser, Frank Abendroth, Olalla Vázquez, Gert Bange, Jörg Walter Bartsch, Detlef Klaus Bartsch, Peter Kolb, Wibke E. Diederich, Uta-Maria Bauer

**Affiliations:** a 9377 Philipps-Universität Marburg, Institute for Molecular Biology and Tumor Research (IMT), BMFZ, Hans-Meerwein-Str. 2, Marburg 35043, Germany; b Philipps-Universität Marburg, Department of Medicinal Chemistry, Center for Tumor and Immune Biology (ZTI), Hans-Meerwein-Str. 3, Marburg 35043, Germany; c Philipps-Universität Marburg, Institute of Pharmaceutical Chemistry, Marbacher Weg 8, Marburg 35032, Germany; d Philipps-Universität Marburg, Center for Synthetic Microbiology (SYNMIKRO), Karl-von-Frisch-Str. 14, 35043 Marburg, Germany; e 54308 Philipps-Universität Marburg, Department of Chemistry, Hans-Meerwein-Str. 4, Marburg 35043, Germany; f Philipps-Universität Marburg, Imaging Core Facility, Center for Tumor Biology and Immune Biology, Hans-Meerwein-Str. 3, Marburg 35043, Germany; g University Hospital Marburg, Department of Neurosurgery, Baldingerstraße, Marburg 35043, Germany; h University Hospital Marburg, Department of Visceral, Thoracic and Vascular Surgery, Baldingerstraße, Marburg 35043, Germany

## Abstract

A substantial body of research implicates PRMTs in the pathogenesis of human diseases, primarily as oncoproteins or tumor suppressors in cancer. Starting from structure-based *in silico* screening of small-molecule databases, we predicted, synthesized, and assayed compounds aimed at specifically inhibiting selected PRMT family members. Unexpectedly, among several PRMT-inhibitory molecules we identified TR-07, a compound that selectively enhances the catalytic activity of PRMT1 *in vitro*. SAR analyses led to the design and synthesis of derivatives with increased potency in activating PRMT1 compared to TR-07 but at the cost of selectivity. TR-07 and its derivatives bind to the αY helix near the catalytic core of PRMT1. Treatment of pancreatic tumor cells with these PRMT1 activators enhanced global ADMA levels and augmented PRMT1’s apoptotic function in cell culture and mouse models. Our results establish TR-07 as the first selective, cell-active PRMT1 activator and underscore the therapeutic promise of this novel class of modulators.

## Graphical abstract

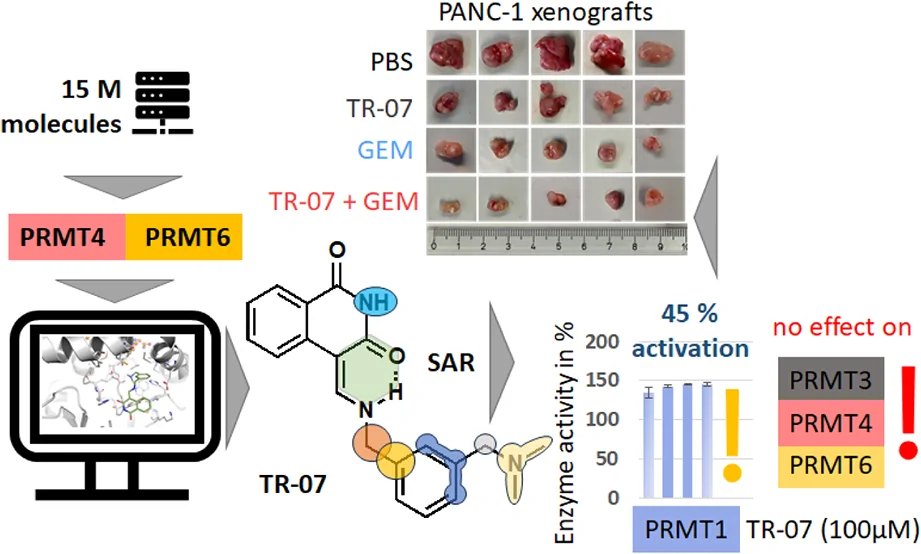

## Introduction

Protein arginine methyltransferases (PRMTs) constitute a family of nine enzymes in mammals that transfer methyl groups from the ubiquitous cofactor S-adenosyl-L-methionine (SAM) to the terminal guanidino nitrogen atoms of arginine residues in proteins. A plethora of nuclear and cytoplasmic proteins are subject to this posttranslational modification, encompassing monomethyl-arginine (MMA) catalyzed by all PRMT members, asymmetric dimethyl-arginine (ADMA) by type I PRMTs, and symmetric dimethyl-arginine (SDMA) by type II PRMTs.^1,2^ The enzymatic activity of PRMTs is indispensable for their biological function of PRMTs.^3^ PRMTs are important epigenetic regulators by methylating histones and other chromatin-associated proteins.^4^ Furthermore, they are involved in DNA damage response, mRNA splicing, translation, and various signal transduction pathways triggered by external stimuli. As a result, these enzymes play pivotal roles in critical cellular decisions, such as proliferation, differentiation, senescence, and apoptosis.^5^

Dysregulation of PRMTs is associated with the onset of various diseases, including cancer.^1,2^ Depending on the cellular context and the cancer subtype, PRMTs can act either as oncoproteins or tumor suppressors. Overexpression of PRMT1, PRMT4, and PRMT6, due to gene amplification or aberrant hypomethylation of CpG islands, has been reported in lung, colon, bladder, and breast cancer, correlating with higher cancer grades and poor patient prognosis.^6–11^ Alternatively, the function of PRMTs can also be altered through mutations in other genes, such as chromosomal translocations in acute myeloid leukemia. These translocations result in the generation of chimeric fusion proteins that aberrantly interact with histone modifiers, such as the major type I enzyme PRMT1. Consequently, PRMT1 becomes misrecruited to inappropriate genomic sites, leading to the erroneous deposition of its histone mark H4R3me2a, and the induction of an oncogenic transcriptional response.^12,13^ Although mutations in the protein-coding region of *PRMT* genes are generally rare, a recent study has identified cancer-associated missense mutations in *PRMT1* that impair the essential dimerization capability of the enzyme, thus causing reduced cellular PRMT1 activity.^14^ Additionally, elevated PRMT expression levels associate with better prognostic outcomes in certain tumor entities, as reported for PRMT1 in neuroblastoma, renal cell carcinoma, gastric cancer, and pancreatic ductal adenocarcinoma (PDAC).^15–18^

The discovery of the pathological implications of PRMTs in a variety of human malignancies has prompted the development of molecules targeting these enzymes.^19^ So far, mainly PRMT inhibitors have been designed, with their inhibitory mechanisms ranging from substrate-competitive, cofactor-competitive to allosteric-binding.^20^ A subset of PRMT inhibitory compounds, most notably those targeting the type II enzyme PRMT5, has advanced to clinical trials and shows great promise as cancer therapeutic drugs.^21^ However, the currently available type I inhibitors are predominantly nonselective but dual- or pan-specific for several PRMT members. Despite the high sequence conservation within the catalytic domain of PRMTs, we and others have identified distinguishing structural features of individual family members using published PRMT crystal structures, protein-protein docking approaches and methyltransferase motif analysis by mass spectrometry.^22–25^ Capitalizing on these findings, we aimed to develop selective, cell-active compounds targeting a subset of type I PRMTs. To this end, we performed structure-based *in silico* screening of small-molecule databases via docking to published crystal structures of PRMTs. These screenings resulted in the selection of compounds predicted to inhibit PRMT4 or PRMT6. Unexpectedly, among several inhibitory compounds, we also identified a PRMT1-activating molecule (K002FT017). In the further course of this study, we focused on the novel PRMT1 activator, deduced potent derivatives and characterized their selectivity and mechanism of action (MOA). Eventually, we investigated the *in vivo* impact of PRMT1’s catalytic activation in pancreatic tumor cells and in a PDAC xenograft mouse model.

## Results

### Design and Screening of Small-Molecule Compounds to Selectively Target Type I PRMTs

In the initial phase of this project, we virtually screened large libraries of commercially available molecules intending to identify selective PRMT4 and PRMT6 inhibitors, as depicted in Figure 1A. Details on the various crystal structures and docking programs are summarized in SI Table S1. To evaluate the effects of the selected candidate molecules on the catalytic activity of type I enzymes, we employed a quantitative fluorescence-based *in vitro* PRMT activity (F-PRMT) assay using recombinant FLAG-tagged PRMT proteins expressed and affinity-purified from baculovirus-infected Sf9 cells (SI Figure S1, SI Figure S2A).^26^ To validate the reliability of the F-PRMT assay for quantifying the inhibitory activity of novel molecules, we determined the IC_50_ values of the pan-type I PRMT inhibitors MS023 and MS049 as well as the PRMT4 inhibitor TP-064 using this assay (SI Figure S2B-E).^27–29^ Our results closely recapitulated the trends of the published IC_50_ values, as for example MS023 inhibited PRMT6 more efficiently (five- to 7-fold) than PRMT1 (SI Figure S2B, C).^28^ The IC_50_ values obtained in the F-PRMT assay were in general higher (1–2 orders of magnitude) than the affinities originally measured in radioactive assays due to the specific assay conditions, as previously reported.^26^

**Figure 1:**
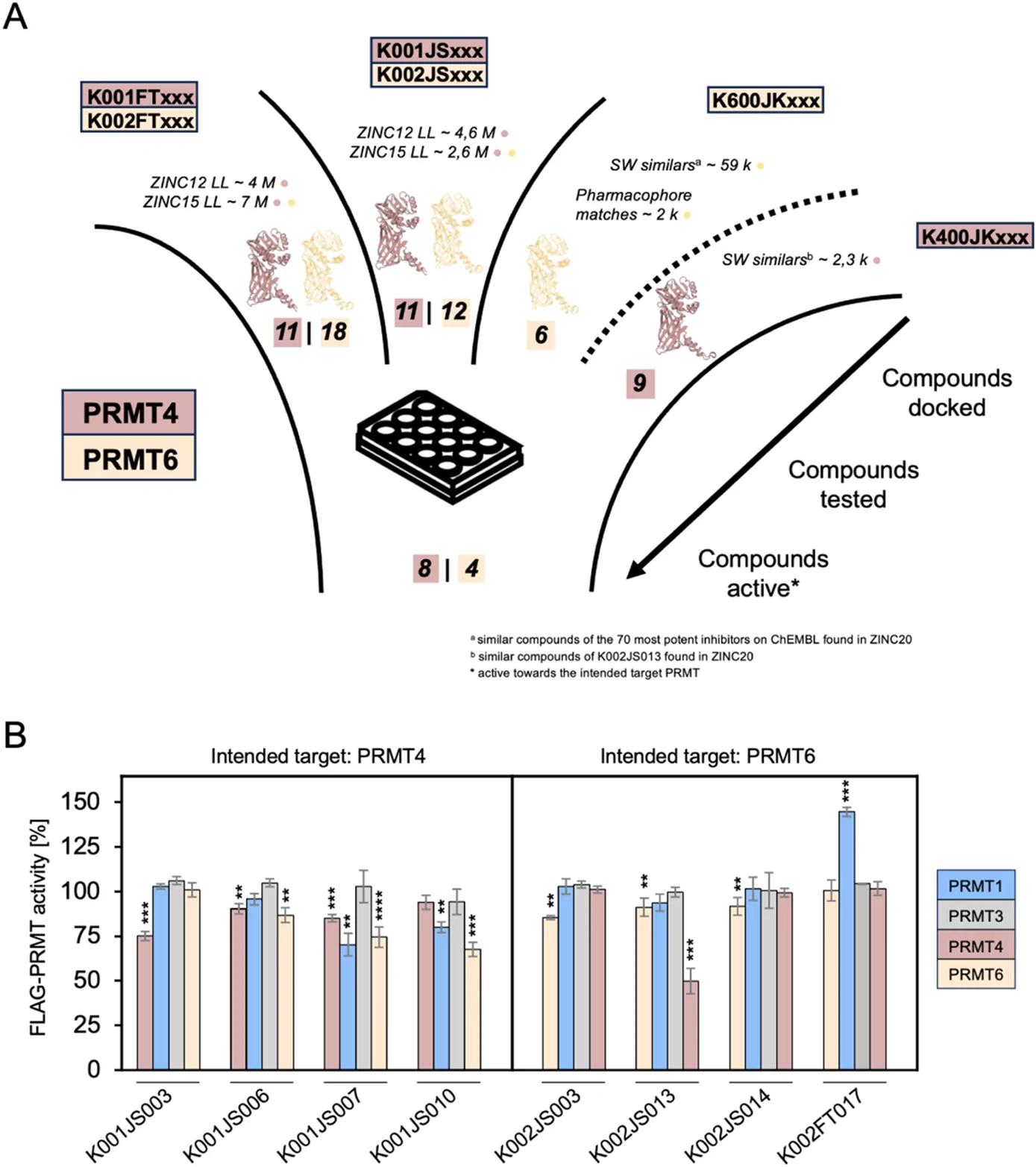
Design and screening of small molecules to inhibit PRMT4 and PRMT6. **A:** Depiction of the docking campaigns, in each case going from the compounds in each database (compounds docked) to each purchased and in F-PRMT assay tested compound (compounds tested). Final number of in F-PRMT assay active (meaning inhibitory for PRMT4 or PRMT6) compounds refers to unique compounds overall (compounds active). Each target PRMT is colored individually: PRMT4 in red and PRMT6 in yellow. Colored dots indicate the intended target for each series of molecules and which database was used. Further details can be found in the Methods. **B:** Selected compounds (100 μM) were tested for their effect on the catalytic activity of the intended target PRMT (as indicated either PRMT4 in red or PRMT6 in yellow) as well as PRMT1 (in blue) or PRMT3 (in gray) using the F-PRMT assay (2 μM of each PRMT). The enzyme activities were calculated based on the mean fluorescence intensities. Activities of compound-treated enzymes are shown in % relative to the solvent-treated enzyme activity, which was set to 100% (mean ± SD, n = 3). Significant p-values for altered activities upon compound treatment are indicated (**: *p*≤ 0.01, ***: *p*≤ 0.001, ****: *p*≤ 0.0001, using the student *t*-test). No indication of a p-value means that the alteration was not significant. All p-values are additionally summarized in Table 1.

Overall, 67 molecules were tested in the F-PRMT assay for their ability to inhibit either PRMT4 or PRMT6 (Table 1, SI Table S2). Although the compounds that emerged from these *in silico* screenings exhibited only modest inhibitory effects, the hit rates were substantial. Between 26% and 11% of the examined compounds were found to interfere with the enzymatic activity of the intended PRMT, PRMT4 and PRMT6, respectively (Table 1, SI Table S2). Besides their low potency, most of these compounds were not selective when evaluated for their impact on the activity of other type I PRMTs, including PRMT1 and PRMT3. As exemplified for eight small molecules (Figure 1B, Table 1), we identified significantly potent PRMT4 inhibitors and PRMT6 inhibitors, with the majority of these molecules showing no preferential effect on their intended target PRMT. To our surprise, K002FT017, which was originally designed to inhibit PRMT6, was found to significantly activate PRMT1, resulting in a 45% enhancement of its enzymatic activity, while neither positively nor negatively influencing the catalytic activities of PRMT3, PRMT4, and PRMT6 (Figure 1B, Table 1). So far, compounds stimulating PRMT1 activity have not been reported. Given that such activators could constitute valuable tools for further elucidating the biological function of the enzyme and testing therapeutic hypotheses, we decided to focus on K002FT017 as a compound with a selective, PRMT1-activating capability.

**Table 1:**
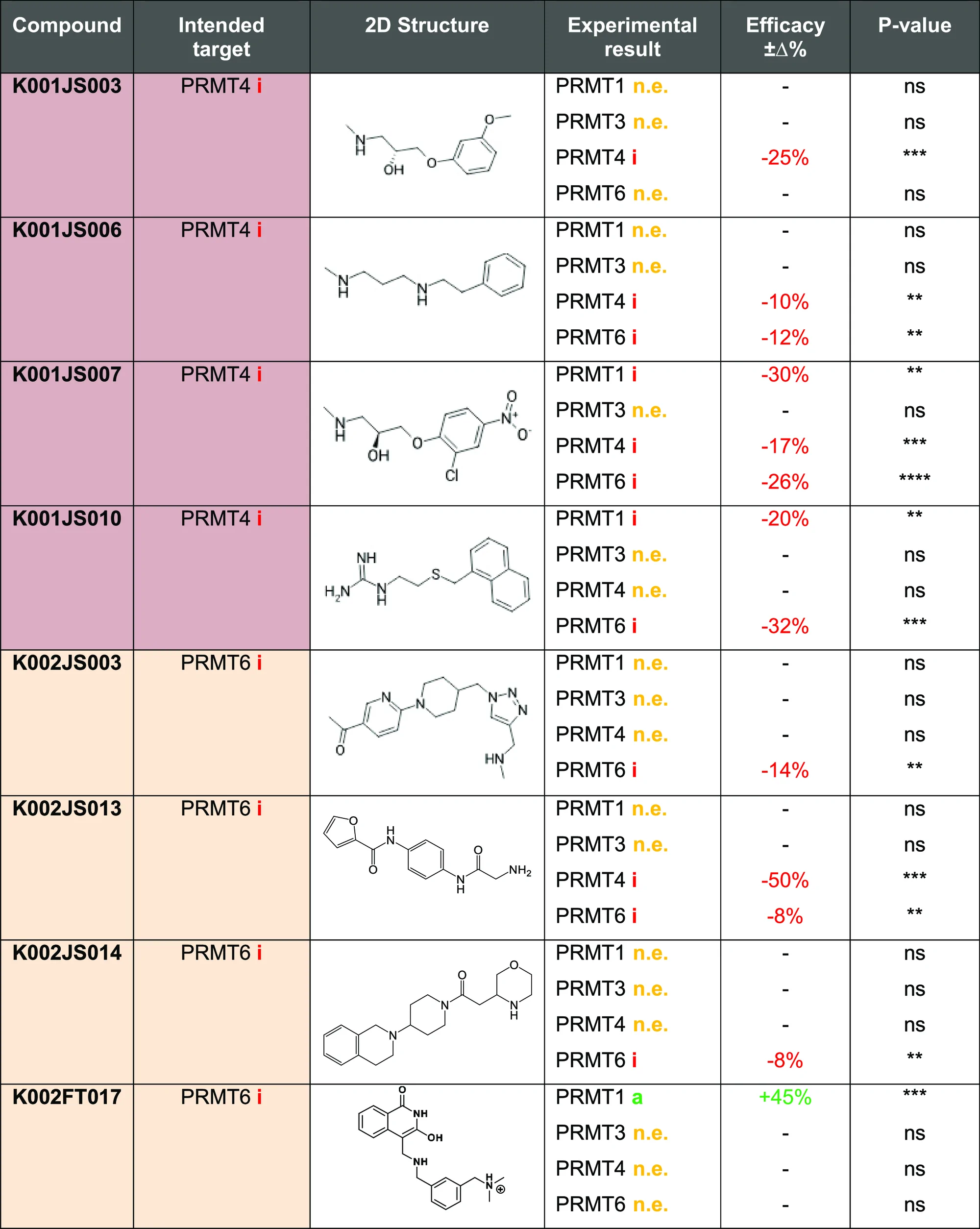
Summary of 8 selected compounds designed to inhibit PRMT4 or PRMT6, with respect to their effects on the catalytic activity of the intended target PRMT and other type I PRMTs^a^

a Compound names, intended PRMT to inhibit (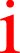), 2D structure, experimental results of the F-PRMT assays, and compounds’ efficacy in altering PRMT activity (±Δ%), and the corresponding p-values of efficacy are summarized. Two-dimensional structures depict the enantiomer that emerged from the docking calculations, whereas racemates were used in all experiments with docking-derived compounds. The PRMT activity upon compound treatment (100 μM) was calculated as % relative to the activity in the presence of solvent (control), for which the activity was set to 100% (n = 3). Changes upon compound treatment (=efficacy) were expressed as mean % differences of inhibition (
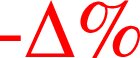
) or activation of catalysis (
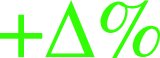
). In case of an inhibitory (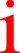) effect for the experimental result, the compound-treated activity was lower by 
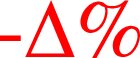
 than the solvent-treated activity, whereas in case of an activatory (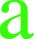) effect, the compound-treated activity was higher by 
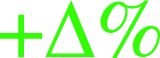
 than the solvent-treated activity. Compounds, which did not significantly affect the catalytic activity (less than ±6% change or not significant p-value), are marked with 
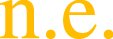
 (no effect) for the experimental result and with – for the efficacy. P-values of efficacy were calculated using the student *t*-test (ns: not significant, **: *p*≤ 0.01, ***: *p*≤ 0.001, ****: *p*≤ 0.0001).

### Revision of the Chemical Structure of K002FT017 and Synthesis/Tautomer Analysis of TR-07

As larger amounts of K002FT017 were required throughout this project and the compound was no longer commercially available, we decided to establish a synthesis strategy for K002FT017 (Figure 2A) that would additionally allow the generation of derivatives, thereby facilitating the deduction of structure-activity-relationships (SAR). Briefly, the reaction of homophthalic acid with urea furnished isoquinoline-1,3-(2*H*,4*H*)-dione (TR-01), which was then reacted with trimethyl orthoformate to form the α,β-unsaturated isoquinoline-1,3-(2*H*,4*H*)-dione (TR-02). The key intermediate TR-07 was obtained by reacting TR-02 further with 1-[3-(aminomethyl)­phenyl]-*N*,*N*-dimethylmethaneamine *2HCl. In the last synthesis step, we envisaged to reduce the imine function to the corresponding secondary amine, thus yielding K002FT017. Despite numerous attempts using various reducing agents, including NaCNBH_3_, NaBH_4_, H_2_/Raney nickel, or H_2_/Pd/C under different reaction conditions, we were unable to achieve the conversion of the imine (Figure 2A).

**Figure 2:**
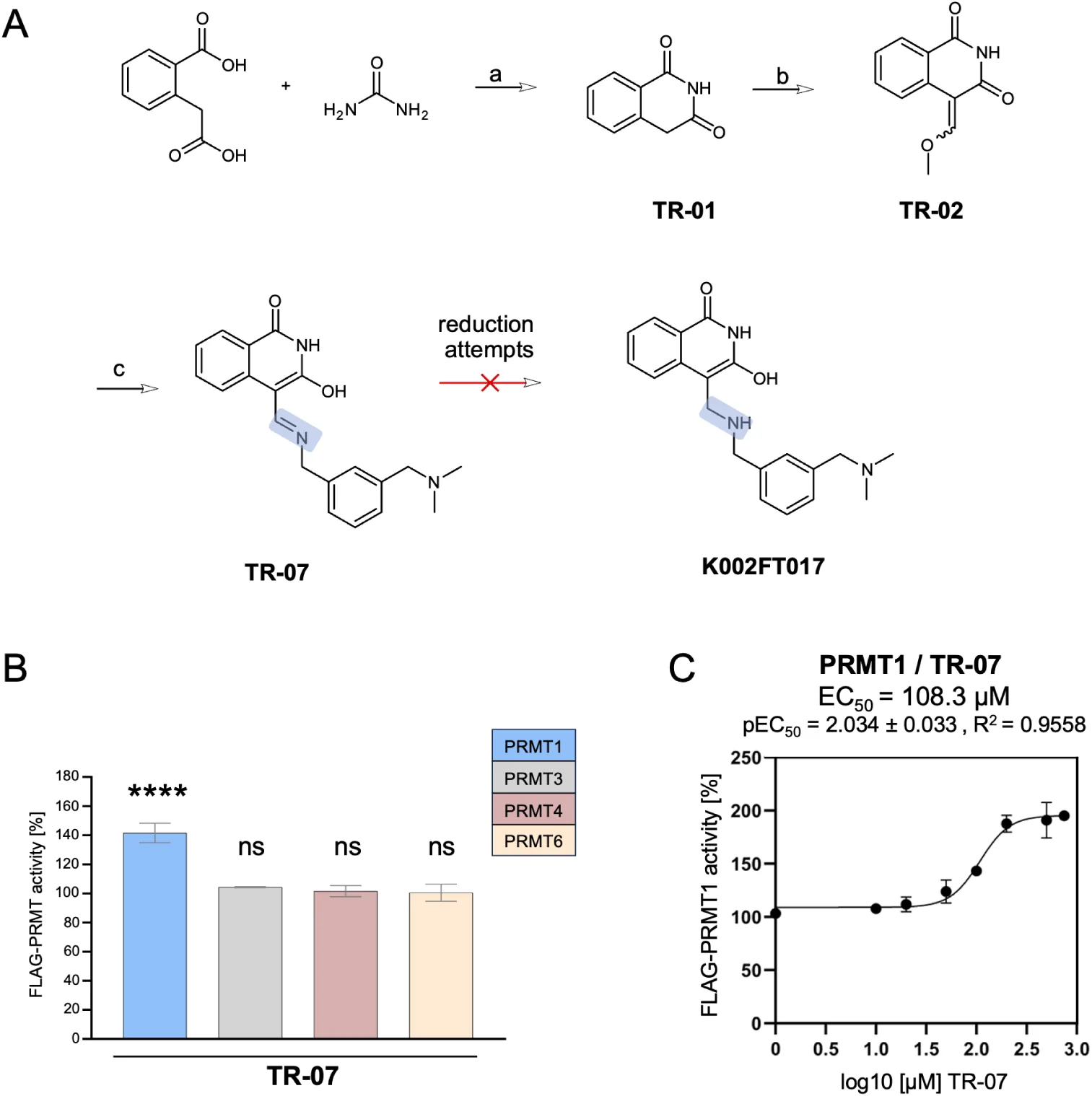
Synthesis of K002FT017 and characterization of its alias TR-07. **A:** Synthesis scheme of K002FT017. Reagents and conditions: a) AcOH, 110 °C, 3 h, 76%; b) trimethyl orthoformate, Ac_2_O, DMF, @MW 125 °C, 30 min, 84%; c) 1-[3-(aminomethyl)­phenyl]-*N*,*N*-dimethylmethaneamine *2HCl, Et_3_N, MeOH, @MW 125 °C, 30 min, 44%. **B:** The activities of the indicated FLAG-tagged PRMTs (2 μM) were measured in the absence (solvent control) or presence of 100 μM TR-07 using the F-PRMT assay and calculated based on the mean fluorescence intensities. Activities of compound-treated enzymes are shown in % relative to the solvent-treated enzyme activity, which was set to 100% (mean ± SD; n = 4–6; ns: not significant; ****: *p* ≤ 0.0001; using the student *t*-test). **C:** The half-maximal effective concentration (EC_50_) of TR-07 for PRMT1 was determined by applying 1, 10, 20, 50, 100, 200, 500, and 750 μM TR-07 to FLAG-tagged PRMT1 (2 μM) in the F-PRMT-assay. The enzyme activity was calculated based on the mean fluorescence intensities. Activity of compound-treated PRMT1 is shown in % relative to the solvent-treated enzyme activity, which was set to 100% (mean ± SD, n = 3), and then plotted against the logarithm of the compound concentrations. Concentration-activity curves were fitted by nonlinear regression, with the EC_50_ value, the pEC_50_ ± SEM and the coefficient of determination (R^2^) indicated.

Nevertheless, we tested TR-07 in the F-PRMT assays and were surprised to find that it also selectively increased the activity of PRMT1 (+45%) comparable to the effect observed for K002FT017 (Figure 2B, C). Consequently, to verify the identity of K002FT017, we recorded a high-resolution mass spectrum and found that it had exactly the same mass and chemical formula as its presumed precursor TR-07, indicating that K002FT017 and TR-07 are the same substance (SI Figure S3A, B). Given that three tautomeric forms of TR-07 are considered plausible (enol-imine, keto-imine, and keto-enamine tautomerism), but only a single set of signals was observed in both the ^1^H NMR and ^13^C NMR spectra, we utilized NMR spectroscopy to confirm that TR-07 exists, at least in solution, as the keto-enamine tautomer (SI Figure S3C-E). The energetically favored *Z*-configuration due to the formation of a hydrogen bond between the enamine nitrogen and the carbonyl oxygen was exemplarily verified by a 1D NOESY NMR experiment with TR-014, a TR-07 derivative later synthesized for the SAR analysis, assuming that this configuration is adopted by all derivatives of this structural type (SI Figure S3F, G). In summary, we demonstrated that the purchased and docked molecule K002FT017 is not the compound postulated by the manufacturer, but instead is identical to its synthetic precursor TR-07, which selectively stimulates the activity of PRMT1.

### Structure-Activity-Relationship (SAR) Analyses of TR-07

We now aimed to develop derivatives with increased potency to activate PRMT1 while maintaining selectivity. Since no information was available at that time regarding a possible binding mode of TR-07 in PRMT1, we pursued a classical ligand-based approach to deduce the SAR. We used this strategy to synthesize a total of 37 TR-07 derivatives from four different molecular series I–IV (Figure 3A, SI Table S3) and tested these derivatives in the F-PRMT assay using FLAG-tagged PRMT enzymes (Figure 3B, C). In addition to reducing the molecular complexity (series I), we systematically varied both the substitution pattern on the benzyl substituent (series II) and on the aniline moiety (series III), and finally increased the rigidity of the ligand (series IV) (Figure 3A).

**Figure 3:**
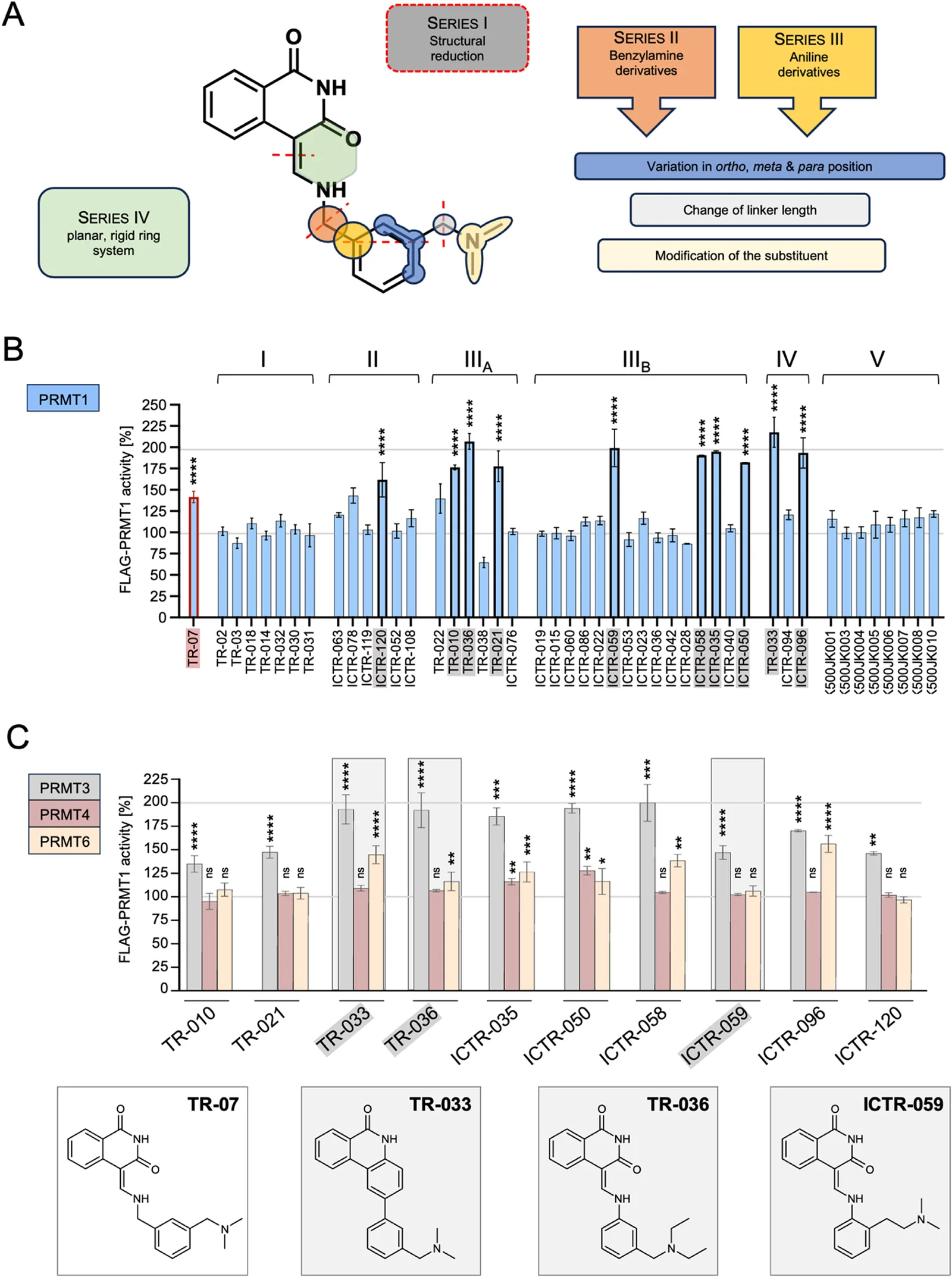
Ligand-based screening and focused docking approach to derive compounds similar to TR-07 for SAR analysis. **A:** Overview of the design concept of the different compound series (I, II, III, IV) derived from the ligand-based approach. **B:** TR-07 and its 45 derivatives (summarized in SI Table S3) deduced from ligand-based screening (in A: series I–IV) and focused docking (series V) were tested for their effect (at a concentration of 100 μM) on the catalytic activity of FLAG-tagged PRMT1 (2 μM) using the F-PRMT assay. PRMT1 activities were calculated based on the mean fluorescence intensities. Compound-treated PRMT1 activities are shown in % relative to the solvent-treated activity, which was set to 100% (mean ± SD, n = 3–6). Ten compounds highlighted were selected with p-values indicated (**: *p* ≤ 0.01, ***: *p* ≤ 0.001, ****: *p* ≤ 0.0001, using the student *t*-test) and subsequently underwent a type I PRMT selectivity testing in C. P-values for all compounds are summarized in SI Table S3. **C:** Selected compounds from B (100 μM) were tested for their effect on the catalytic activity of other type I enzymes (PRMT3 in gray, PRMT4 in red, and PRMT6 in yellow) using the F-PRMT assay. Enzymatic activities were calculated based on the mean fluorescence intensities. Compound-treated enzyme activities are shown in % relative to the solvent-treated activity, which was set to 100% (mean ± SD, n = 3–6; ns: not significant, **: *p* ≤ 0.01, ***: *p* ≤ 0.001, ****: *p* ≤ 0.0001, using the student *t*-test; with all p-values additionally summarized in SI Table S3). The most potent and semi/dual-selective TR-07 derivatives, TR-033, TR-036, and ICTR-059, are highlighted and their structural formulas are displayed (including TR-07).

*Series I: Reduction of molecular complexity.* In this series of molecules (Figure 3A, SI Table S3), we reduced the molecular complexity by removing the substituents on the benzylic moiety (TR-03), replacing the benzylic moiety with an aminoalkyl chain (TR-018) or a phenyl substituent (TR-014). In addition, the benzyl moiety was exchanged for a 3-pyridyl (TR-032) or the aniline moiety for a 2-pyridyl (TR-030) or a 3-pyridyl (TR-031) substituent. Testing revealed that the compounds had no significant effect (TR-02, TR-014, TR-030, and TR-031) or even a slight inhibitory effect (TR-03) on PRMT1 (Figure 3B). Both the alkyl derivative (TR-018) and the pyridyl-substituted derivative (TR-032) activated PRMT1 in a significant manner, albeit to a considerably lesser extent than TR-07.

*Series II: Modifications in the Southern Region—benzylamine derivatives.* Within this series of compounds, we focused on the substitution pattern of the benzylic moiety and synthesized the structural isomers ICTR-063 and ICTR-078 [ortho vs para-substitution] as well as ICTR-119 and ICTR-120 [ortho vs meta-substitution]. For comparison, ICTR-052 and the alkylated and thus permanently charged derivative of TR-07, namely ICTR-108, were also synthesized. F-PRMT activity assays indicated that ortho-substitution is less favored since ICTR-063 and ICTR-119 showed a reduced activating effect on PRMT1 and no significant effect, respectively, compared to TR-07 (Figure 3B, SI Table S3). Replacement of the dimethylaminomethyl group of TR-07 with a diethylaminomethyl moiety (ICTR-120) resulted, on the one hand, in a significant increase in PRMT1 activity to 60%, but on the other hand also led to stimulation of PRMT3 activity by 45% (Figure 3C). The para-dimethylaminomethyl substituted derivative ICTR-078 was selective for PRMT1 (SI Table S3), but less potent than TR-07, with an activity increase of 40%. Both ICTR-052 and the alkylated derivative ICTR-108 had no effect or a modest activating effect on PRMT1 activity compared to TR-07.

*Series IIIA: Aniline Derivative.* Since a significant increase in PRMT1 activation was observed when the benzyl-substituted derivative TR-03 was modified by introducing an additional aminoalkyl moiety at the benzyl group as in TR-07, we investigated in series IIIA whether this effect could also be observed when starting with the aniline derivative TR-014. We therefore installed a (dimethylamino)­methyl group either in ortho (TR-022), meta (TR-010) or para (TR-021) position and, for comparison, a (diethylamino)­methyl moiety (TR-036) as well as a morpholino substituent (TR-038) in meta position. In ICTR-076, an iso-butyl substituent was introduced as a carbon analog of TR-021 to assess the effect of the protonatable nitrogen on PRMT1 activation. With the exception of the compounds TR-038 and ICTR-076, which showed either a strong inhibitory effect or no effect on the activity of PRMT1, the other compounds led to an equal (TR-022) or significantly enhanced activation of PRMT1, compared to TR-07, but often paired with a decrease in selectivity (Figure 3B, C). Notably, ICTR-076 containing the 4-iso-butyl substituent turned out to be particularly interesting as it showed no activity compared to TR-021, supporting the hypothesis that a basic nitrogen is obviously essential for activity.

*Series IIIB: Ortho substituted Aniline Derivatives.* As a substituent in ortho position appeared to promote selectivity toward PRMT1, and the aniline derivatives showed higher activity than the benzyl derivatives, we systematically modified the ortho position of the aniline system. Compounds ICTR-019, ICTR-015, and ICTR-060 showed no effect on PRMT1 activity, suggesting that the aniline moieties are too weak bases (Figure 3B). Similarly, the triazole (ICTR-042) and pyrrole (ICTR-036) derivatives exhibited no significant or minimal inhibitory activity toward PRMT1. Taken together, these observations confirmed that the basicity of the substituent plays a crucial role for PRMT1 activation. Within this series, the piperazine derivatives stood out: all three compounds, ICTR-058, ICTR-035 and ICTR-050, showed agonist effects, with increases in PRMT1 activity up to 95% compared to 45% for TR-07. Yet, these significant increases were again accompanied by a substantial loss of selectivity (Figure 3B, C, SI Table S3). Likewise, the compound ICTR-059 demonstrated a notable increase in PRMT1 activity by 100%, albeit again at the expense of selectivity due to concomitant PRMT3 activation.

*Series IV: Planar, rigid Ring System.* As demonstrated (SI Figure S3) the compounds exist in solution as the Z-configured keto-enamine tautomer, which allows a hydrogen bond between one of the carbonyl oxygens of the ureide functionality and the NH of the amine, thereby forming a “pseudo” ring system. To investigate the biological relevance of this interaction, a three-membered ring system was synthesized. Since a further substitution at the pyridine nitrogen would lead to the introduction of a permanent charge, we decided on the commercially available 6-(5*H*)-phenanthridinone as the new core scaffold, where further substituents can be introduced at the 2-position, resulting in TR-033, ICTR-094 and ICTR-096. The testing in the F-PRMT assay confirmed the design hypothesis, as the compounds TR-033 and ICTR-096 significantly increased PRMT1 activity, but again paired with a decrease in selectivity (Figure 3B, C, SI Table S3). Shifting the *N*,*N*-diethylamino substituent to the ortho position (ICTR-094) resulted in a significantly lower activation of PRMT1 compared to TR-07.

Given that all these derivatives were synthesized from the central intermediate TR-02 (Figure 2A, SI Table S3), modifications to the central core, an isoquinoline-1,3-(2*H*,4*H*)-dione, were set aside. However, we explored such modifications with eight compounds based on a focused docking screen of compounds similar to TR-07 (series V, SI Table S3). The testing of these series V compounds in the F-PRMT assay revealed that they activate PRMT1 not or less effectively than TR-07, indicating that variations at this end of the ligand are not beneficial (Figure 3B). Despite extensive efforts, any attempt to derive molecules with higher potency than TR-07 while preserving PRMT1 selectivity remained unsuccessful.

In summary, among the total 45 SAR compounds analyzed (SI Table S3), we identified 10 TR-07 derivatives (TR-010, TR-021, TR-033, TR-036, ICTR-035, ICTR-050, ICTR-058, ICTR-059, ICTR-096, ICTR-120) that enhanced the catalytic activity of PRMT1 by at least 60% and thus displayed a better potency than TR-07 (highlighted in Figure 3B). As the remaining derivatives either did not influence or only weakly elevated (less than TR-07) or even inhibited the activity of PRMT1, they were not considered further. In addition to the *in vitro* activation potency, selectivity was another key criterion for choosing derivatives for subsequent functional characterization. Therefore, these 10 most efficacious PRMT1 activators underwent a specificity screening to evaluate their impact on the catalytic activities of PRMT3, PRMT4, and PRMT6 (Figure 3C). None of the compounds was as selective for PRMT1 as the initial compound TR-07, since all enhanced the activity of at least one additional type I PRMT member of the tested enzyme subset. The compounds were classified as dual-selective, if they activated PRMT3 in addition to PRMT1 (TR-010, TR-021, ICTR-059, and ICTR-120). Semiselective compounds stimulated PRMT3 and PRMT6 in addition to PRMT1 (TR-033, TR-036, ICTR-058, and ICTR-096). The compounds that activated all tested type I PRMTs (PRMT1, PRMT3, PRMT4, and PRMT6) were categorized as nonselective type I activator compounds (ICTR-035 and ICTR-050). For the follow-up analyses, SAR-derived molecules with at least 2-fold *in vitro* activation potency and either dual or semiselectivity were chosen. Among the dual-selective TR-07 derivatives, ICTR-059 demonstrated the strongest effect in enhancing the PRMT1 activity by 2-fold (up to 200% compared to the solvent-treated activity of 100%) (Figure 3B). In the group of semiselective compounds, TR-033 and TR-036 were even more potent than ICTR-059, increasing the PRMT1 activity more than 2-fold (up to 215–225%). In summary, these results identified the original lead compound TR-07 as the most selective, but moderately potent PRMT1 activator, and its derivatives ICTR-059, TR-033, and TR-036 as more potent (≥2-fold), but dual- or semiselective. Accordingly, these four compounds were selected for subsequent analyses.

### Impact of TR-07 and Its Derivatives on PRMT1-Mediated Methylation of Natural Substrates and on Cellular Arginine Methylation Levels

Given that the effects of the PRMT1 activators had been screened and quantified so far using the F-PRMT assay, we next assessed the efficacy of the selected compounds in stimulating PRMT1-mediated methylation of natural substrates. To this end, we conducted *in vitro* methyltransferase assays using recombinant PRMT1 and core histones or GST-tagged GAR (glycine-arginine-rich domain of fibrillarin) as substrates of PRMT1.^30,31^ Addition of the cofactor [^14^C]-methyl-SAM allowed for the visualization of radiolabeled methyl group incorporation into the substrate proteins by SDS-PAGE and autoradiography or by liquid scintillation counting. Upon application of the PRMT1 activator TR-07 and its derivatives (TR-033, TR-036, and ICTR-059), the methylation activity of both FLAG-tagged and His-tagged PRMT1 was increased toward its substrates, histones H2A and H4 as well as GAR, in comparison to the DMSO-treated controls (Figure 4A–D). It is important to note that PRMT1 methylates both H2A and H4, albeit with variable efficiency. In some experiments, H2A was found to be stronger methylated than H4, and vice versa, while in other experiments both histones exhibited equivalent methylation levels (Figure 4A, B, SI Figure S1). Consistently, the magnitude of activation by TR-07 and its derivatives varied with regard to methylation of H2A and/or H4. In some instances, the enhancement was more pronounced for H2A or H4, or was comparable for both histones. As a result, the methylation signals of both histones were considered in the quantification process (Figure 4A, B). Altogether, these findings were consistent with those obtained in the F-PRMT assay and confirmed that TR-07 and its selected derivatives enhance the enzymatic activity of PRMT1 also toward natural and physiologically relevant substrates *in vitro*.

**Figure 4:**
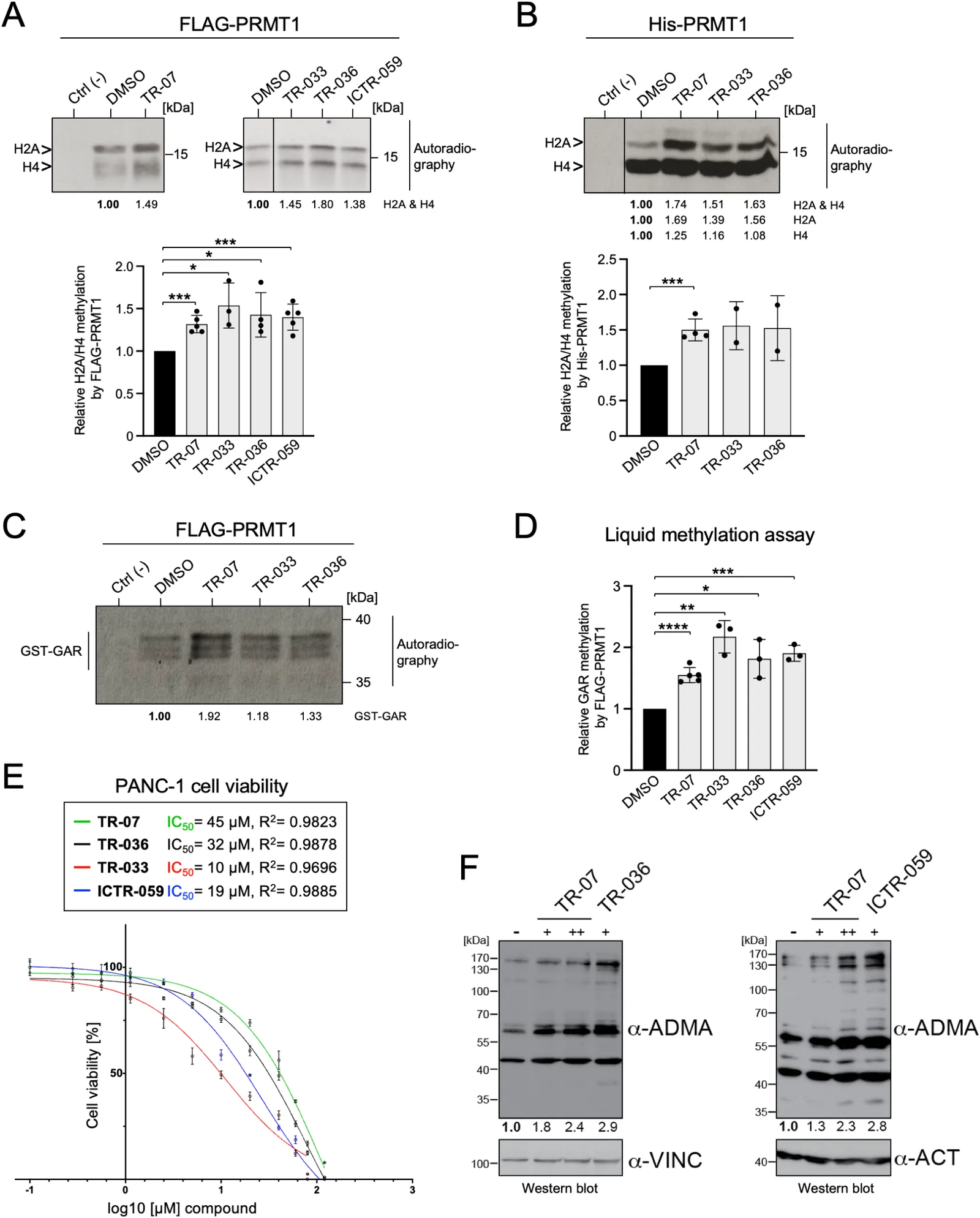
Impact of TR-07 and its derivatives on PRMT1-mediated *in vitro* methylation of natural substrates and on global arginine methylation levels *in vivo*. **A-C:** FLAG-tagged PRMT1 (A, C) or His-tagged PRMT1 (B) was subjected to *in vitro* methyltransferase assays using core histones (A, B) or GST-GAR protein (C) as the substrates together with [^14^C-methyl]-SAM in the absence (DMSO-treated) or presence of TR-07, TR-033, TR-036, or ICTR-059 (100 μM). As negative control (Ctrl (−)), PRMT1 enzymes and [^14^C-methyl]-SAM were incubated without substrates. Subsequently, samples were separated by SDS-PAGE, blotted, and analyzed by autoradiography. Substrates (H2A, H4 or GST-GAR) and molecular weights (kDa) of the protein marker are indicated. Black lines designate composite images, assembled from the same blots and exposure times. The methylation signals of the substrates were densitometrically quantified, and expressed relative to the DMSO-treated control, as indicated by the numbers below the autoradiographs. Representative results are depicted, with quantification graphs (below the autoradiographs in A and B) summarizing biological replicates. The PRMT1 methylation activity toward its substrates in the presence of compounds is displayed relative to the DMSO control, which was set to 1 (mean ± SD, n = 2–5; *: *p* ≤ 0.05, ***: *p* ≤ 0.001, using the student *t*-test). **D:** FLAG-tagged PRMT1 was subjected to liquid *in vitro* methyltransferase assays using GST-GAR protein as the substrate together with [^14^C-methyl]-SAM in the absence (DMSO-treated) or presence of TR-07, TR-033, TR-036, or ICTR-059 (200 μM, except for TR-033 100 μM). Subsequently, GST-GAR was immobilized on glutathione-beads and PRMT1 activity was measured by quantifying the incorporation of ^14^C-methyl groups (in cpm) using liquid scintillation counting. The activity in the presence of the compounds is displayed relative to the DMSO control, which was set to 1 (mean ± SD, n = 3–5; *: *p* ≤ 0.05, **: *p* ≤ 0.01, ***: *p* ≤ 0.001, ****: *p* ≤ 0.0001, using the student *t*-test). **E:** PANC-1 cells were cultured for 96 h in the presence of DMSO (solvent control) or the activator compounds TR-07, TR-033, TR-036, and ICTR-059, each at 11–12 different concentrations. Cell viability was quantified using the CellTiter-Glo assay. Viability of compound-treated cells is shown in % relative to the viability of solvent-treated cells, which was set to 100% (mean ± SD, n = 3), and then plotted against the logarithm of the compound concentrations. Concentration-viability curves were fitted by nonlinear regression, with the half-maximal inhibitory concentration (IC_50_) of the compounds for PANC-1 cell viability and the coefficient of determination (R^2^) indicated. **F:** PANC-1 cells were cultured for 72 h in the presence of DMSO (−) as solvent control or in the presence of the PRMT1 activators TR-07, TR-036, ICTR-059 (+: 10 μM, ++: 20 μM). Subsequently, whole-cell extracts were prepared and analyzed by Western blot using antibodies against ADMA, ACTIN (ACT), and VINCULIN (VINC), of which the latter two stainings served as loading controls. Molecular weights (kDa) of the protein marker are indicated. All the ADMA signals per lane were densitometrically quantified, normalized to the signal of the respective loading control and expressed relative to the DMSO-treated control, as indicated by the numbers below the immunostainings.

Next, we applied TR-07 and its derivatives to the pancreatic tumor cell line PANC-1 to explore their effects on cell viability and the cellular arginine methylation levels. Dose-response curves for PANC-1 cell viability were generated to determine the half-maximal inhibitory concentration (IC_50_) of each compound (Figure 4E). TR-07 demonstrated the lowest level of cytotoxicity among the four compounds (IC_50_: 45 μM) followed by TR-036 (IC_50_: 32 μM) and ICTR-059 (IC_50_: 19 μM), whereas TR-033 was found to be the most toxic (IC_50_: 10 μM). Subsequently, PANC-1 cells were cultured in the presence of DMSO or TR-07 and its derivatives at concentrations at least 2-fold below their respective IC_50_ concentrations. The cells were then analyzed by Western blot using antibodies against asymmetric dimethyl arginine (ADMA) in proteins. TR-07 treatment resulted in a concentration-dependent increase in global arginine methylation in cells compared to DMSO-treated (−) control cells (Figure 4F). Similar results were obtained for TR-036 and ICTR-059 (Figure 4F), whereas TR-033 did not produce consistent effects, likely due to impaired cell viability even at low concentrations (2.5 and 5 μM; data not shown). Collectively, these results indicated that TR-07, TR-036, and ICTR-059 are effective activators of type I PRMT enzymes at nontoxic concentrations in a cellular context.

### Identification of the Binding Region of TR-07 and Its Derivatives in PRMT1 and Their Structural Effects

K002FT017 was initially identified by docking into the substrate binding site of PRMT6, a site commonly targeted by competitive inhibitors. However, given the experimental results showing that TR-07 (the revised chemical structure of K002FT017) leads to increased PRMT1 activity, a compound binding site that overlaps with the substrate binding site seemed counterintuitive. Therefore, unbiased docking calculations using the entire surface of the protein appeared as a way to determine other possible binding regions. Given the number of rotatable bonds in TR-07, we decided to first dock only its rigid ring system onto the surface of a dimeric structure of human PRMT1 (SI Figure S4). After visual inspection of the poses and considering the SAR results (Figure 3A, B), we ended up with four possible binding regions. Three of these regions, indicated by no. 1–3 in SI Figure S4, were bordering the dimerization arm of one of the monomers, while the fourth one (no. 4 in SI Figure S4, Figure 5A) was located right at the entry of the substrate binding site, suggesting a highly probable binding region in the space between residues E47 and Y148. When taking into account that a protonatable nitrogen attached to the lower benzene ring is important for the compound’s PRMT1 enhancing activity (Figure 3A, B), we found that one of the three dimerization arm-proximal regions (no. 3 in SI Figure S4) contains a possible complement in the form of an acidic side chain (E216), which could interact with the protonated nitrogen. This region is also very close to an already known binding epitope for allosteric PRMT3 inhibitors.^32^ Remarkably, the glutamic acid is conserved among PRMT1 homologues from various organisms^33^ and is located at the same position in PRMT3 and PRMT6, but is replaced by a glutamine in PRMT4 (SI Figure S5), which would be consistent with our observation that PRMT4 cannot be activated by the majority of the SAR-deduced TR-07 derivatives (Figure 3C).

**Figure 5:**
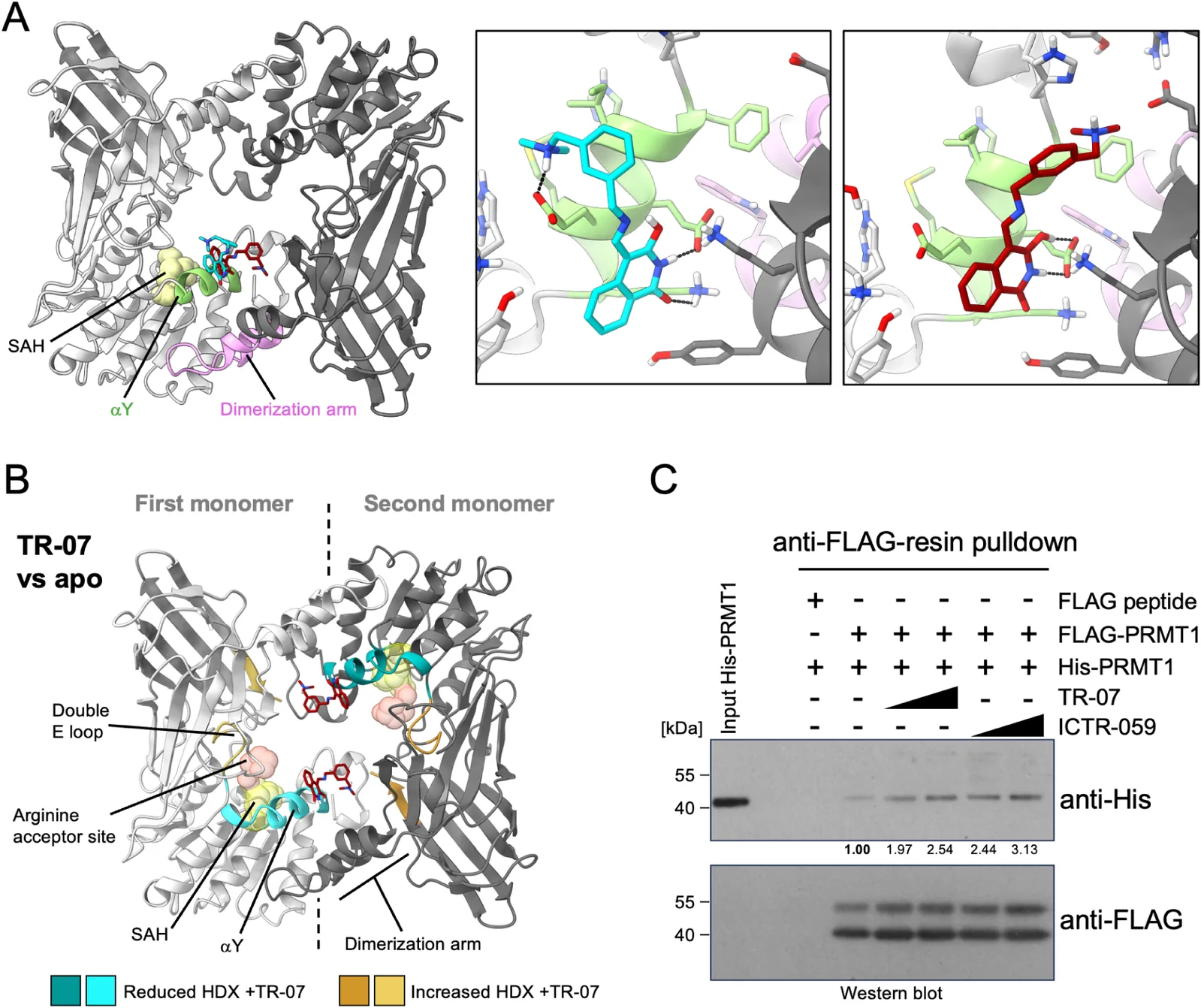
Characterization of the MOA of TR-07 and its derivatives by HDX-MS and protein–protein interaction assays. **A:** Two possible docking poses of TR-07 in the region of the human PRMT1 dimer (PDB 6NT2) identified by HDX-MS as a potential binding site. The poses were obtained by docking with HYBRID onto previously obtained docking poses of TR-033 (TR-07 in cyan) and TR-036 (TR-07 in red). The two monomers are colored in dark and light gray. The dimerization arm of the right monomer is colored in pale pink. The αY helix of the other monomer is colored pale green. The SAH location (derived from a superposition with PDB ID 6NT2) is indicated in pale yellow, but the molecule was not present during the docking calculations. The two images on the right show a close-up view of each docking pose separately. **B:** Differences in deuterium incorporation of TR-07-bound FLAG-tagged PRMT1 (holo state, + TR-07) compared to the apo state (- TR-07) were plotted onto the human PRMT1 dimer (PDB ID 6NT2) as in A. The TR-07 pose (in red) was obtained by docking with HYBRID onto the previously obtained docking pose of TR-036. The dashed line separates the first and the second monomer. Dark/bright cyan colors correspond to lower deuterium incorporation (<5%) and dark/bright yellow colors to higher incorporation (>5%) in the holo state compared to the apo state (TR-07 vs apo) occurring at any time point of HDX (SI Figure S6C). The *N*-terminal αY helix, the SAH location (derived from a superposition with PDB ID 3Q7E), the arginine acceptor site (derived from a superposition with PDB ID 1OR8), the Double E loop, and the dimerization arm are indicated. **C:** For pulldown assays, FLAG-tagged PRMT1 immobilized to anti-FLAG resin and eluted His-tagged PRMT1 were incubated in the presence of DMSO (− TR-07/ICTR-059) as solvent control or in the presence of increasing amounts (200 and 500 μM) of TR-07 and ICTR-059. Subsequently, FLAG-beads were washed and bead-bound proteins were analyzed together with an His-tagged PRMT1 input (20 ng) by Western blot using antibodies against the His- and FLAG-tags. As negative control, anti-FLAG resin saturated with FLAG peptide was incubated with His-tagged PRMT1. The His-PRMT1 signals were densitometrically quantified, corrected to the FLAG-PRMT1 signals, and expressed relative to the solvent control, as specified by the numbers below the immunostainings. Molecular weights (kDa) of the protein marker are indicated.

To gain further insights into the mechanism of action (MOA) of the activating compounds, we conducted Hydrogen/Deuterium exchange mass spectrometry (HDX-MS) on the selective lead compound TR-07 and the potent semiselective derivative TR-036. FLAG-tagged PRMT1 was incubated over a range of times without or with compound added in D_2_O-containing buffer. After digestion of the protein into peptides, the deuterium incorporation was quantified by mass spectrometry. The peptides analyzed for their HDX covered approximately 95% of the FLAG-PRMT1 amino acid sequence (SI Figure S6A, S7A, SI Data S1). The HDX fingerprint mirrored the overall PRMT1 topology in that the Rossmann fold and β-barrel domains incorporated deuterium at a lower rate than the *N*-terminal 40 amino acid residues, indicating that the latter are rather disordered (SI Figure S6B, S7B).^33^

Next, the HDX patterns of the ligand-bound (holo) versus the ligand-free (apo) state of PRMT1 were compared to map the putative epitope(s) of compound binding, assuming that the activator molecules interact directly with PRMT1. Compound binding would be expected to reduce deuterium incorporation specifically in regions of PRMT1 that mediate the interaction, leading to a decrease of deuterium exchange (protection). Compound-induced conformational changes were anticipated to cause increased deuterium incorporation and therefore a gain of exchange (deprotection) in the affected protein regions.

In comparison to the apo state, we found that TR-07 induced a decrease in deuterium incorporation predominantly in the dynamic αY helix (aa 41–51) at the *N*-terminus of PRMT1 (Figure 5B, SI Figure S6B-D). The αY helix regulates cofactor as well as substrate accessibility^33,34^ and is adjacent to the bipartite catalytic core that consists of the Rossman fold (aa 51–175) responsible for SAM-binding through conserved motifs (I, post I, II, III) and the β-barrel domain (aa 176–353) encompassing the dimerization arm. Furthermore, TR-07-induced deprotection of areas, marked by increased deuterium uptake, was detected in the Double E loop, a region within the Rossman fold that forms the active site together with the THW loop (Figure 5B, SI Figure S6B-D). Similar to TR-07, TR-036 resulted in a protection of the αY helix and in a deprotection of the Double E loop compared to the PRMT1 apo state (SI Figure S7B-D). Additionally, TR-036 protected a region adjacent to the signature motif II in the Rossman fold. In general, the magnitude of HDX changes compared to the PRMT1 apo state was slightly higher with TR-036 (maximal difference of 18.5%) than with TR-07 (maximal difference of 12.7%). Collectively, our data showed that both TR-07 and TR-036 interact with the αY helix upstream of PRMT1’s catalytic core and cause conformational changes within the Double E loop, which is part of the active site and critical for catalysis.^33^

Homodimerization of PRMTs is essential for their catalytic activity.^14,34,35^ Given that the αY helices and dimerization arms contact each other within the PRMT1 dimer^34^ and that αY exhibited HDX changes in the presence of TR-07 and TR-036 compounds, we questioned whether the activators could potentially affect the dimerization capability of PRMT1. To this end, we performed pulldown assays using FLAG-tagged PRMT1 immobilized to anti-FLAG resin, which was incubated with eluted His-tagged PRMT1, either in the absence or presence of the activator compounds TR-07, TR-033, TR-036, and ICTR-059. Resin-bound proteins were analyzed by SDS-PAGE and Western blot with antibodies against the His- and FLAG-tag. As expected, an interaction between the two PRMT1 species, FLAG-tagged PRMT1 and His-tagged PRMT1, was detected in the absence of compounds (Figure 5C, SI Figure S8). Addition of the four compounds caused an elevated interaction of the two PRMT1 species, as indicated by the increased detection of His-tagged PRMT1 upon compound treatment and displayed in representative experiments (Figure 5C, SI Figure S8) as well as in the quantification of biological replicates for TR-07 (SI Figure S9). Anti-FLAG resin saturated with FLAG peptides served as negative control and showed no pulldown of His-tagged PRMT1 protein. These results indicated that TR-07 and its derivatives have a positive impact on the homooligomeric interaction of PRMT1.

### Effect of TR-07 and Its Derivatives on the Pro-Apoptotic Activity of PRMT1

PRMT1 stimulates apoptosis by methylating pro-apoptotic proteins, such as the tumor suppressor p14^ARF^, the BCL-2 antagonist BAD, as well as the transcription factors FOXO1 and E2F-1.^18,36–38^ Our recent work identified PRMT1 as a positive regulator of intrinsic apoptosis triggered by the chemotherapeutic gemcitabine in pancreatic cancer cells.^18^ Accordingly, we focused here on characterizing the PRMT1 activator compounds in terms of their *in vivo* efficacy to enhance PRMT1-mediated apoptosis in PDAC cell lines. To this end, PANC-1 and PaTu8988T cells were treated with increasing concentrations of gemcitabine in the presence of either DMSO or TR-07. Caspase 3/7 activity assays revealed that both PDAC cell lines underwent apoptosis in a gemcitabine concentration-dependent manner, which was further significantly enhanced by co-treatment with TR-07 (Figure 6A). In contrast, gemcitabine-induced apoptosis was diminished in the presence of the type I PRMT inhibitor MS023, known to efficiently inhibit PRMT1,^28^ in agreement with our previous observations.^18^ These findings were further corroborated by AnnexinV/propidium iodide (AV/PI) staining measured by flow cytometry, which revealed a significant increase in the early and late apoptotic stages of both PDAC cell lines upon co-treatment with gemcitabine and TR-07 compared to gemcitabine monotreatment (Figure 6B, C). Application of TR-07 alone did not significantly induce apoptosis (Figure 6A-C). Remarkably, the dual-specific PRMT1 activator compound ICTR-059 weakly enhanced the gemcitabine-induced apoptosis in PaTu8988T cells, but had no effect in PANC-1 cells, whereas the two pan-specific activators TR-033 and TR-036 rather diminished the gemcitabine-triggered cell death (SI Figure S10). It is important to note that TR-07 and its derivatives were used in these apoptosis assays at concentrations at least 2-fold below their respective IC_50_ values for cell viability (Figure 4E, SI Figure S11), to avoid interference of the apoptosis readouts with global cytotoxic effects. These findings indicated that modulation of PRMT1 activity through a selective activator compound, such as TR-07, can enhance the pro-apoptotic function of PRMT1. In contrast, unselective type I activators, such as TR-033 and TR-036 predominantly lead to an anti-apoptotic response, potentially due to composite pan-specific effects.

**Figure 6:**
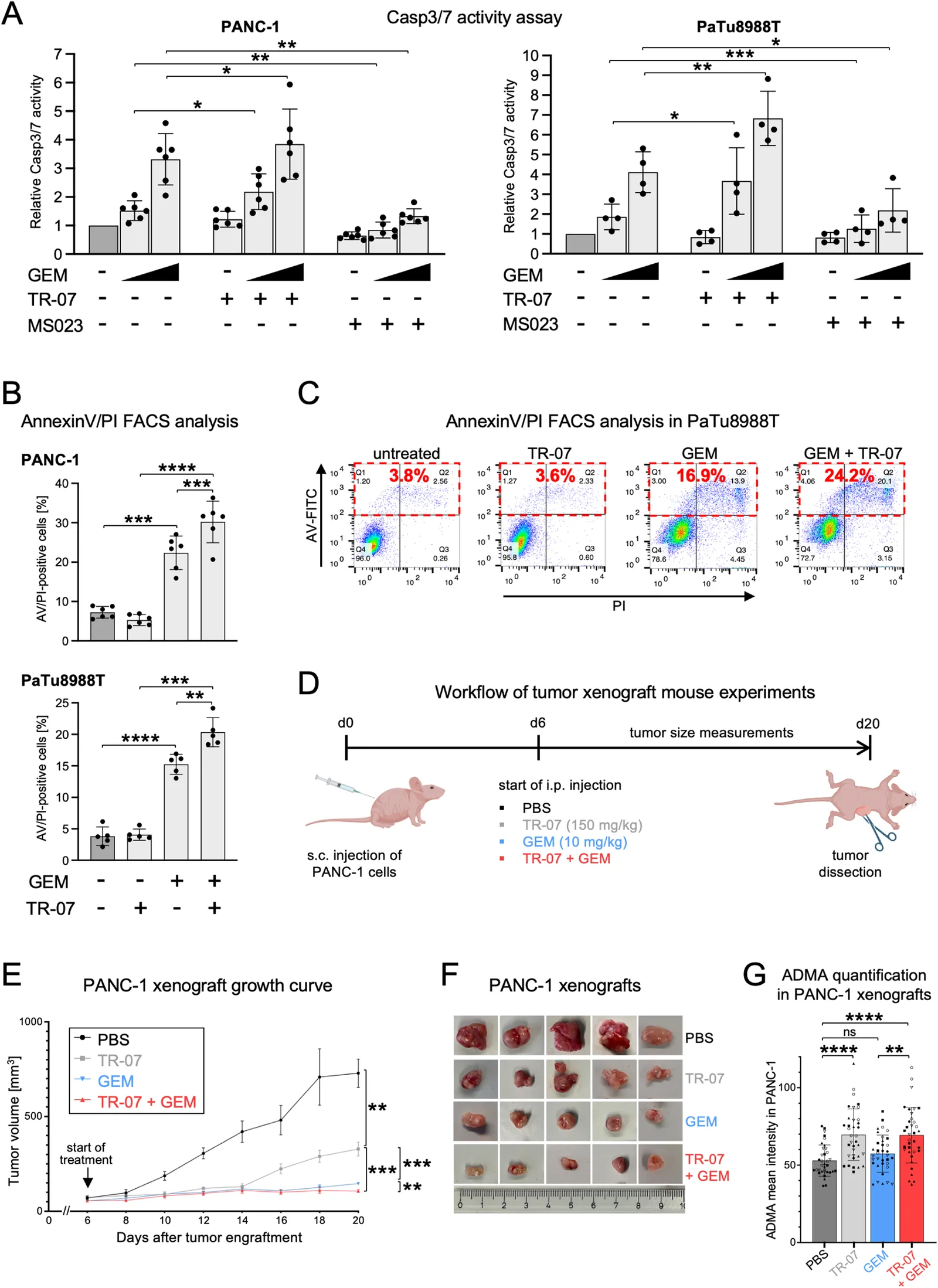
Effect of small-molecule modulators on gemcitabine-induced apoptosis and tumor growth of PDAC cells. **A:** PANC-1 and PaTu8988T cells were cultured in the absence (−) or presence (+) of TR-07 or MS023 (10 μM). After 2 h, gemcitabine (GEM) was added at increasing concentrations (10 and 25 nM) or not (−) for another incubation of 72 h. Subsequently, the luminescence-based caspase3/7 assay was performed. The activity of caspase 3/7 in treated cells was normalized to the activity measured in untreated cells (dark gray bar), which was set to 1 (mean SD, n = 6 for PANC-1 and n = 4 for PaTu8988T; *: *p* ≤ 0.05, **: *p* ≤ 0.01, ***: *p* ≤ 0.001, using the student *t*-test). **B, C:** PANC-1 and PaTu8988T cells were cultured in the absence (−) or presence (+) of TR-07 (10 μM). After 24 h, gemcitabine (GEM, 1 μM) was added (+) or not (−) for another incubation of 48 h. Subsequently, the apoptotic cell fraction was quantified in % by flow cytometry using FITC-labeled Annexin V (AV) and propidium iodide (PI) staining (in B: mean ± SD, n = 6 for PANC-1 and n = 5 for PaTu8988T; **: *p* ≤ 0.01, ***: *p* ≤ 0.001, ****: *p* ≤ 0.0001, using the student *t*-test). In C, a representative flow cytometry experiment of B is displayed. Viable cells are AV/PI double-negative (AV–/PI–, lower left quadrant, Q4), early apoptotic cells are AV single-positive (AV+/PI– upper left quadrant, Q1), late apoptotic cells are AV/PI double-positive (AV+/PI+, upper right quadrant, Q2), and necrotic cells are PI single-positive (AV–/PI+, lower right quadrant, Q3). The sum of early and late apoptotic cell numbers (Q2+Q3 in %) is indicated in red. **D-G:** The workflow of the PDAC xenograft experiments in mice is depicted in D. PANC-1 cells (2 × 10^6^ cells/mouse) were subcutaneously (s.c.) injected in the right flank of female athymic nude mice. Small tumor nodules appeared 6 days post-engraftment. Mice were then randomly divided into four cohorts that received either PBS (solvent control), TR-07, gemcitabine (GEM), or a combination of TR-07 and GEM (TR-07 + GEM) for 2 weeks. Tumor volumes were measured every second day using a digital caliper. The tumor volumes of each treatment cohort are displayed in E (mean ± SEM, n = 5 for each cohort; **: *p* ≤ 0.01, ***: *p* ≤ 0.001, using the student *t*-test for the last time point). At the end of the 2 weeks, mice were sacrificed and tumors were dissected (F). Cellular ADMA levels (G) were analyzed in the dissected PANC-1 xenograft tissues using an antibody against ADMA in immunofluorescence (IF) staining (representative IF images for PBS and TR-07 are shown in SI Figure S13). The mean intensity of ADMA-positive PANC-1 cells was quantified for 7 independent, randomly selected standardized areas per xenograft of 5 animals of each the PBS, TR-07, GEM and TR-07 + GEM treatment cohort (mean ± SD, n = 5 × 7; ns: not significant, **: *p* ≤ 0.01, ****: *p* ≤ 0.0001, using the student *t*-test). The 7 mean intensity values per mouse are depicted with the following symbols: ●, ○, ▲, ▽, or ■, □.

Given the cooperative effect of TR-07 and gemcitabine in inducting apoptosis of human PDAC cell lines, our next objective was to examine the effect of TR-07 and gemcitabine co-treatment in a PDAC xenograft mouse model. PANC-1 cells were subcutaneously injected into the flank of female, immunodeficient athymic nude mice (Figure 6D). Six days post-engraftment, the mice were randomly assigned to four treatment cohorts: a control group receiving only PBS (solvent), two monotherapy groups receiving either TR-07 or gemcitabine, and a combination treatment group receiving both drugs. The intraperitoneal injections were administered for a period of 2 weeks, during which the tumor sizes were measured every second day (Figure 6D, E). The applied doses of TR-07 and gemcitabine were well tolerated by the mice, exhibiting no significant adverse or systemic toxic effects, as indicated by the monitoring of mouse body weights (SI Figure S12). Subsequently, the mice were sacrificed, and the tumors were dissected (Figure 6F). Individual treatments with either gemcitabine or TR-07 significantly reduced the tumor growth compared to the control mice (Figure 6E, F). Co-treatment of gemcitabine and TR-07 led to a further significant reduction in tumor growth compared to the monotherapies. Immunofluorescence staining of the PANC-1 xenografts demonstrated elevated mean intensity ADMA levels upon TR-07 application, both as monotherapy or in combination with gemcitabine, compared to the cohorts treated with PBS or gemcitabine alone (Figure 6G, SI Figure S13). These results substantiated the *in vivo* efficacy and bioavailability of the activator compound TR-07. Moreover, these findings in the PDAC xenograft mouse model are consistent with the results obtained in PDAC cell cultures (Figure 6A-C), indicating that the PRMT1 activity cooperates with gemcitabine to exert an anti-tumor effect and seems to enhance the sensitivity to gemcitabine-induced apoptosis.

## Discussion and Conclusion

The original objective of this study was to identify novel inhibitors that specifically target the substrate binding site of PRMT4 or PRMT6, taking into account their unique structural features.^22–25^ To this end, a structure-based *in silico* screening of small-molecule databases was conducted in conjunction with testing of screening hits using a quantitative F-PRMT assay on an artificial substrate molecule.^26^ To our surprise, the compound K002FT017, which was initially docked to inhibit PRMT6, was discovered to be a selective and moderately efficient PRMT1 activator. Given the significant focus on identifying small molecules that inhibit PRMT enzyme function,^19^ there have been no reports to date of compounds stimulating PRMT1 activity, though PRMT4 enhancers have been described.^39^ These PRMT4 enhancers also derived as unexpected hits from an inhibitor screen for PRMT1 and are ureidoacetamido-indole derivatives. Testing of these enhancer molecules in *in vitro* and cellular assays on natural PRMT4 substrates revealed a selective, albeit substrate-specific activity, with an efficiency similar to that observed here for the PRMT1 activator. Direct interactions of the PRMT4 enhancer molecules with substrates or the enzyme were excluded by biophysical measures, leaving the MOA unclear.^39^ In light of the potential value of gain-of-function reagents in (i) further elucidating the intricate biological PRMT functions and (ii) therapeutic application in contexts of disease-protective enzyme contributions as well as loss-of-function conditions, we decided to characterize the PRMT1-activating small molecule identified in the present work in more depth.

A second unexpected result emerged when we encountered difficulties synthesizing K002FT017. High-resolution mass spectrometry and NMR analysis demonstrated that the purchased K002FT017 compound did not match the structure provided by the manufacturer, but had exactly the same mass, chemical formula and activity profile as its synthetic precursor TR-07. SAR analyses using TR-07 as the hit compound identified derivatives that stimulated the catalytic activity of PRMT1 with a similar or better potency relative to TR-07. However, none of the more potent derivatives exhibited the same level of selectivity for PRMT1 as the starting compound. Instead, the activity of at least one additional type I PRMT member was enhanced. Overall, the selectivity of the PRMT1 activators was evaluated exclusively on a subset of type I PRMTs, omitting PRMT2 and PRMT8 (the close brain-specific relative of PRMT1) as well as type II and type III PRMTs,^4^ or lysine methyltransferases.

To gain insights into the MOA of the PRMT1 activators, HDX-MS was conducted for TR-07 and TR-036. Both molecules were found to interact with the αY helix upstream of the catalytic core of PRMT1 and cause conformational changes within the Double E loop, which is part of the active site and critical for catalysis.^33^ As the αY helices and the dimerization arms have been shown to contact each other in the PRMT1 dimer,^34,40^ we investigated the impact of the activators on PRMT1 dimerization, which is pivotal for the enzymatic activity.^14,33,35^ Protein-protein interaction assays revealed that TR-07 and its derivatives enhanced the homodimerization of PRMT1, suggesting that they may act as allosteric regulators, potentially promoting substrate accessibility and affinity, thereby accelerating PRMT1’s catalytic reaction. To identify amino acids crucial to the interaction between PRMT1 and TR-07, we performed site-directed mutagenesis (data not shown) and individually mutated amino acids in the αY helix (G43, E47), in the double E loop close to the active site (Y148) or in the dimerization arm (E216). Based on our SAR, HDX-MS, and unbiased docking results, these amino acid positions were hypothesized to be in close proximity to the compounds’ interaction site or to directly interact with the compounds. However, the mutant PRMT1 proteins did not show reduced TR07-mediated activation in F-PRMT assays, indicating that none of the targeted residues contributes exclusively to the binding region of the compound (data not shown). Instead, TR-07 and its derivatives might exhibit modest affinities for multiple residues in PRMT1, potentially bind a composite cluster of residues, or interact with allosteric, transient, or cryptic sites that only form under specific conformational states of PRMT1, which are challenging to capture by single point mutations. Therefore, additional structural and biophysical methods are required in the future to accurately determine the binding location of TR-07 and its derivatives and to enable a more rational design of improved compounds with higher potency and maintained selectivity.

PRMT1 is the predominant type I enzyme in mammalian cells, accounting for more than 85% ADMA activity in cells.^41^ Deletion of the gene in mice results in embryonic lethality.^42^ In addition, the enzyme regulates various essential physiological processes during adulthood.^43^ To investigate whether TR-07 and the selected derivatives would also enhance the catalytic activity of PRMT1 toward physiologically relevant substrates, including histone H4 arginine 3 and the GAR domain of fibrillarin,^30,31^ *in vitro* methyltransferase assays were performed. The PRMT1 activators demonstrated comparable potency and activity profiles on both histone and nonhistone substrates to those obtained in the F-PRMT assay. In addition, as determined by Western blot and IF staining of ADMA levels, the PRMT1 activators stimulated *in vivo* methylation in a pancreatic tumor cell line and in tumor xenograft tissues. Nevertheless, unbiased approaches to assess TR-07-mediated global changes in cellular ADMA as well as MMA levels using mass spectrometry should be considered in future studies.

Extensive research over the past years has revealed both oncogenic and tumor-suppressing functions of PRMT1 in cancer initiation, progression and metastasis, highlighting its therapeutic potential. In cancers in which the oncogenic impact of PRMT1 predominates, its pharmacological inhibition shows great therapeutic promise.^19,44^ In contrast, in cases of malignancies, such as neuroblastoma, renal cell carcinoma, gastric cancer, and PDAC, where PRMT1 expression levels have been linked to a more positive outcome, suggesting a tumor-suppressive role for PRMT1, activator compounds, such as TR-07 and its derivatives, could represent valuable tools to provide therapeutic benefits.^15–18^ In liver cancer, ADMA produced by PRMT1 inhibits the inducible NO synthase, thereby attenuating oxidative stress and inflammatory responses, which in turn reduces hepatic tumor growth.^45^ By methylating RIP3, a central pro-necroptotic factor, PRMT1 prevents necroptosis and progression of tumor immune escape in colon cancer.^46^ Our previous work and that of other researchers have demonstrated that PRMT1 increases the apoptosis of cancer cells, e.g., in the context of chemotherapy-induced cell death, by direct interaction and methylation of anti- and pro-apoptotic regulators, such as p14^ARF^, BAD, FOXO1, E2F1, ATF4 and CFLAR_L_.^18,36–38,47,48^ Consequently, in these cancer entities, gain of function reagents for PRMT1 might enhance the anti-tumor effects of PRMT1 in monotherapy or in combination with chemotherapy. Likewise, the impaired dimerization capability of cancer-associated missense mutations in PRMT1 might be counteracted by PRMT1 activators.^14^

To assess the therapeutic potential of the PRMT1 activator compounds identified in this study, we leveraged our prior findings demonstrating that, under chemotherapy-induced genotoxic stress conditions, such as gemcitabine application, PRMT1 promotes apoptosis of PDAC tumor cells.^18^ Our findings here revealed that the PRMT1-selective activator TR-07 enhances the sensitivity to gemcitabine-induced apoptosis, both in cell culture and in a PDAC xenograft mouse model. In contrast, the unselective type I activators TR-033 and TR-036, which stimulate the activity of PRMT3 to a similar extent as the activity of PRMT1, led to a predominantly anti-apoptotic response in PDAC cell lines. Notably, a recently developed PRMT3 degrader has been shown to induce intrinsic apoptosis in cancer cells.^49^ Therefore, the effect of TR033 and TR036 observed here could be due to their ability to reduce cellular apoptosis via PRMT3 and a possible dominance of PRMT3 in regulating apoptosis in our cell system.

While the treatment of TR-07 alone displayed no effect on proliferation and apoptosis of PDAC cell cultures, TR07 monotherapy in the mouse xenograft experiments demonstrated certain anti-tumor activity. This discrepancy likely stems from the stark differences in the experimental setup of both assays. In cell culture, TR-07 was applied once for a duration of 72–96 h at concentrations at least 2-fold below the IC_50_ value for *in vitro* cell viability, to minimize a potential interference of apoptosis readouts with global cytotoxic effects. In contrast, the mouse experiments were conducted over a 14-day period, with compound administration every second day in a concentration similar to the common application of PRMT inhibitors. In addition, a direct comparison between drug concentrations in cell culture (μM) and the dose in animal models (mg/kg) is challenging due to the fact that they display different characteristics. Furthermore, pharmacokinetic data remain to be elucidated, as the monitoring of such in-depth profiles appears to be only reasonable at higher potency of the activator compounds, which necessitates further refinement of TR-07 in the future, as discussed above. Collectively, the results of the present study identified PRMT1-selective activator compounds that have the potential to provide a therapeutic benefit in cancer types where PRMT1 executes tumor-suppressive functions.

## Experimental Section

### Computational Methods

#### First Round of Large-Scale Docking Calculations Against PRMT4 and PRMT6 (K00xFT0xx)

The structures for the human homologues of PRMT4 and PRMT6 were taken from the PDB: PDB ID 5DXA^50^ for PRMT4 and PDB ID 4Y30 ^51^ for PRMT6. Both structures were prepared in MOE2015 (Chemical Computing Group Inc., 2016). Docking was performed with DOCK 3.6 ^52–54^ and the “lead-like” subsets from ZINC12 ^55^ (PRMT4) and ZINC15 ^56^ (PRMT6). Each subset was docked considering different protonation states of the molecules, which lead to a total of ∼4 million compounds/protomers docked to PRMT4 and ∼7 million docked to PRMT6. We visually inspected the top-ranked molecules (1500 in the case of PRMT4 and 3000 in the case of PRMT6) for steric complementarity, lack of stranded hydrogen bond donors and acceptors and absence of charge mismatches.

#### Second Round of Large-Scale Docking Calculations Against PRMT4 and PRMT6 (K00xJS0xx)

Differing from the first round of calculations, the structures used were PDB IDs 6ARV,^57^ 6DVR , 5DWQ,^50^ and 5DX0 ^50^ for PRMT4 and 4Y30 ^51^ for PRMT6. Those structures were prepared using Wit!P (Witnotp – Biomolecular NMR Spectroscopy Platform (BNSP), ETH Zurich (2017): https://bnsp.ethz.ch/softwar/witnotp.html), with uncertain protonations of histidines resulting in separate preparations. At first, DOCK3.6 ^52–54^ and the ZINC12 ^55^ database were used, while later docking calculations employed DOCK3.7 ^58^ and ZINC15.^56^ A detailed overview which structure/program/database was used can be found in SI Table S1. The restrictions regarding the estimated partition coefficient and molecular weight for the sets were kept for the ZINC12 ^55^ dockings. For ZINC15-based dockings, the in-stock lead-like subset was used. Each subset was docked considering different protonation states of the ligands, which lead to a total of ∼4.6 million compounds/protomers docked to PRMT4 using the ZINC12 ^55^ library and ∼2.6 million compounds/protomers docked to PRMT4 and PRMT6 using the ZINC15 ^56^ library. We visually inspected the top-ranked molecules for steric complementarity, lack of stranded hydrogen bond donors and acceptors and absence of charge mismatches.

#### Docking Calculations of Ligands Similar to the Ones Identified in Rounds 1 and 2 Against PRMT1 (K500JKxxx), PRMT4 and PRMT6 (Kx00JK0xx)

##### PRMT1 (K500JKxxx)

A ligand-based virtual screening campaign was initiated using TR-033 and TR-036, the most potent activators identified at that stage, as query molecules. The similarity search was conducted in ZINC20 ^59^ using SmallWorld (https://sw.docking.org/search.html; default settings), resulting in a focused set of structurally related compounds. 3D conformers of all retrieved molecules were generated using OMEGA.^60^ A binding pose of TR-033 in the target protein was used to construct the receptor model for subsequent docking. The set of structurally related compounds was docked into the protein structure PDB ID 6NT2 ^61^ using both FRED^62–64^ and HYBRID (OpenEye),^62,64^ providing complementary scoring and pose prediction. The top 500 scoring poses from each docking method were manually evaluated with a focus on meaningful interactions involving positively charged nitrogen atoms, which were known to be key contributors to activity in this series. Promising poses were subjected to a 2-step minimization protocol: initial geometry refinement was performed with SZYBKI (http://www.eyesopen.com), while keeping the protein rigid, followed by a second round of minimization in MOE (Amber10:EHT force field; Chemical Computing Group Inc., 2016) allowing limited flexibility of protein side chains to further optimize the binding geometry. This process yielded 13 high-quality protein–ligand complexes suitable for further analysis. In the end, 8 compounds (series V, Figure 3B) were bought.

##### PRMT4 (K400JKxxx)

A similarity search was performed using SmallWorld with K002JS013 as the query molecule and ZINC20 as the library. The retrieved subset was docked into PRMT4 using OpenEye HYBRID,^62,64^ employing the crystal structure of PRMT4 (PDB ID: 2Y1X).^65^ This structure includes a ligand bearing an α-amino acid motif, making it a suitable template for the alignment and docking of K002JS013-like compounds. After docking, poses were minimized using OpenEye’s SZYBKI (http://www.eyesopen.com) tool to refine geometry and optimize interactions. In the end, 9 compounds were bought.

##### PRMT6 (K600JKxxx)

To identify novel inhibitors of PRMT6 targeting the substrate binding site, a ligand-based virtual screening approach was employed. Bioactive compounds with reported IC_50_ values ≤ 10 μM were extracted from the ChEMBL^66^ database, yielding a curated set of approximately 100 molecules. These compounds served as input for a similarity search using SmallWorld (https://sw.docking.org/search.html; default settings) across the ZINC database, resulting in a library of ∼59,000 structurally related analogs. To further enrich for compounds likely to bind the PRMT6 active site, pharmacophore-based filtering was performed using a merged model, constructed from all available PRMT6 crystal structures cocrystallized with orthosteric ligands (PDB IDs: 4Y2H,^51^ 4Y30,^51^ 5E8R,^28^ 5EGS,^67^ 6P7I,^68^ and 7NR4 ^69^) in LigandScout.^70,71^ Approximately 2,000 compounds matched this pharmacophore model and were subsequently converted into 3D conformers using OMEGA. These were docked into the six PRMT6 structures using both FRED^62–64^ and HYBRID (OpenEye),^62,64^ resulting in 12 independent docking campaigns. The top 500 scoring poses from each run were assessed for the presence of key interactions with Tyr51, Glu155, Glu164, and His317 – residues consistently involved in ligand binding across human PRMT6 structures. Selected poses exhibiting favorable interaction patterns were energy-minimized in MOE (Chemical Computing Group Inc., 2016) using the Amber10:EHT force field, allowing for limited flexibility of the ligand (max. 1.5 Å) and binding site residues (max. 0.5 Å). Where necessary, the protonation state of His317 was adjusted to optimize hydrogen bonding. This refinement resulted in 75 high-quality protein–ligand complexes. Following manual curation to eliminate duplicates and structurally redundant analogs (e.g., minor substitutions such as cyclopropyl vs ethynyl), a final set of 26 distinct protein–ligand complexes was retained for further analysis. In the end, 6 compounds were bought.

#### Unbiased Docking Calculations

We used chains A and D of the structure with PDB ID 6NT2,^61^ which we prepared by using the QuickPrep function of MOE2019 (Chemical Computing Group Inc., 2016) with the default settings. For the docking, we used the SwissDock web-service^72,73^ with default settings.

### Synthesis

#### Purity Statement

The vast majority of the final products have a purity of ≥ 95%, as determined by elemental analysis or qNMR (SI Figure S18–S97). Compounds ICTR-035 (92.1%), ICTR-040 (94.2%), and TR-033 (93.7%) have a slightly lower purity according to qNMR, which may be caused by residual water. For Compounds TR-07, TR-033, TR-036, and ICTR-059 the purity was additionally determined by HPLC-analyses (SI Figure S14-S17).

#### General Information

All reactions involving moisture- or oxygen-sensitive reagents were carried out in heated (450 °C) glass apparatus under inert gas atmosphere (Argon N50, Air Liquide). Commercially available solvents and reagents were used without further purification, unless otherwise noted. The reaction control was performed by thin-layer chromatography using aluminum-coated TLC plates (Merck TLC Silica gel 60, F_254_) or by HPLC-MS [1260 Infinity (Agilent), column: Poroshell 120, EC-C18, 2.7 μM, 4.6 mm × 50 mm (Agilent), MS: Expression S CMS (Advion)]. If required for TLC evaluation, Ehrlich’s reagent was used for staining. The reactions using microwave radiation were carried out in specially designed pressure-resistant glass reactors in a microwave reactor (Discover system with Explorer autosampler, CEM). Cooling was carried out either with ice or dry ice. Drying of synthesis products was carried out under reduced pressure in a high vacuum system, optionally at elevated temperature. The drying time varied from a few hours to several days. The pump used was a Chemistry Hybrid Pump RC 6 equipped with a VAP 5 manometer (both vacuum fringe), combined with a Glass Oven B-585 from Büchi. Two systems were used for purification by MPLC, Reveleris X2 (Grace, now Büchi) and pump module C-601 (x2), pump manager C-615, UV detector C-630 and fraction collector C-660 (all Büchi). Prepacked cartridges of various sizes (particle size: 15, 30, or 40 μM) from different manufacturers were used (FlashPure from Büchi, PuriFlash from Interchim). Purification by HPLC was performed on a Pure C-850 FlashPrep system from Büchi with a PrepPure column (C18, 100 Å, 10 μM, 250 mm × 20 mm, Büchi). Unless otherwise stated, the standard program (5–95% MeCN in H_2_O over 1 h) was used for purification with preparative HPLC without the further addition of acids or bases. The Delta 5.0.4.4 software (JEOL) was used for processing and interpreting of the NMR spectra. The indicated chemical shifts (in ppm) were referenced to the respective residual solvent signal according to the literature.^100^ The following abbreviations were used to indicate spin multiplicities: s (singlet), br s (broad singlet), d (doublet), dd (doublet of doublets), ddd (doublet of doublet of doublets), t (triplet), dt (doublet of triplets), q (quartet), m (multiplet), and sm (symmetric multiplet). The measurements were performed in the analytical departments for mass spectrometry and elemental analysis and NMR spectroscopy of the chemistry and pharmacy departments. The spectrometers used were either an AV III HD 250 MHz and AV III 300 MHz (Bruker) or an ECX400 and ECA500 (JEOL). Measurements with high-resolution mass spectra were performed on an AccuTOF-GCv (JEOL),an LTQ-FT system or an Orbitrap Q Exactive plus mass spectrometer (both Thermo Fisher Scientific). Elemental composition was determined using a CHN­(S) analyzer vario MICRO CUBE (Elementar). Melting points were measured with an MPM-M2 (Schorpp) or M 5000 (Krüss). All melting points are uncorrected.

***Isoquinoline-1,3-(2H,4H)-Dione (TR-01)***. Homophthalic acid (5.00 g, 27.80 mmol, 1.00 equiv) was suspended in AcOH and heated to 110 °C. Subsequently, urea (11.90 g, 197.40 mmol, 7.10 equiv) was added in portions over 2 min and the reaction mixture was stirred at 110 °C for 3 h. After cooling to room temperature, the mixture was poured into 300 mL of ice water and stirred for an additional 30 min. The resulting precipitate was filtered, washed with H_2_O (2 × 50.00 mL) and MeOH (3 × 50.00 mL) and then dried under high vacuum. Compound **TR-01** (3.40 g, 21.10 mmol) was obtained as a colorless to pale yellow solid with a yield of 76%. mp 230.2 °C.^74^ ^1^H NMR (500 MHz, DMSO-*d*_6_, 300 K): δ_H_ (ppm) = 11.26 (s, 1H), 8.01 (ddd, ^3^ *J* = 7.7 Hz, ^4^ *J* = 1.4 Hz, ^5^ *J* = 0.6 Hz, 1H), 7.64 (td, ^3^ *J* = 7.5 Hz, ^4^ *J* = 1.4 Hz, 1H), 7.45 (td, ^3^ *J* = 7.6 Hz, ^4^ *J* = 1.2 Hz, 1H), 7.38 (ddd, ^3^ *J* = 7.7 Hz, ^4^ *J* = 1.2 Hz, ^5^ *J* = 0.6 Hz, 1H), 4.03 (s, 2H,) ppm.^75^ ^13^C NMR (125 MHz, DMSO-*d*_6_, 300 K): δ_C_ (ppm) = 170.9, 165.3, 136.6, 133.4, 127.8, 127.4, 127.1, 125.0, 35.9.^75^ HR-MS: calcd for C_9_H_6_N_1_O_2_ [M-H]^−^ 160.0404; found: 160.0403.^75^ Purity was determined by qNMR using maleic acid as an internal standard: 94.6%.

***(E/Z)-4-(Methoxymethylene)­isoquinoline-1,3­(2H,4H)-dione (TR-02).*** To a suspension of **TR-01** (3.00 g, 18.6 mmol, 1.00 equiv) in DMF (8.00 mL), trimethyl orthoformate (8.14 mL, 74.4 mmol, 4.00 equiv) and Ac_2_O (12.0 mL, 0.65 mL/mmol) were added and stirred for 30 min under microwave conditions (125 °C, 300 W). After cooling to room temperature, Et_2_O (300 mL) was added and the mixture was stirred for an additional 5 min at room temperature. The resulting precipitate was filtered, washed with Et_2_O (3 × 50.0 mL) and dried under high vacuum (10^–3^ mbar) to obtain **TR-02** (3.19 g, 15.7 mmol) as an orange to yellow solid with a yield of 84%. mp decomposition at >260 °C.^74^ ^1^H NMR (500 MHz, DMSO-*d*_6_, 300 K): δ_H_ (ppm) = 11.24 (s, 1H), 8.25 (d, ^3^ *J* = 8.0 Hz, 1H), 8.07 (ddd, ^3^ *J* = 7.8 Hz, ^4^ *J* = 1.1 Hz, ^5^ *J* = 0.5 Hz, 1H), 8.03 (s, 1H), 7.68 (td, ^3^ *J* = 7.8 Hz, ^4^ *J* = 1.4 Hz, 1H), 7.41 (td, ^3^ *J* = 7.6 Hz, ^4^ *J* = 1.1 Hz, 1H), 4.22 (s, 3H).^74^ ^13^C NMR (125 MHz, DMSO-*d*_6_, 300 K): δ_C_ (ppm) = 165.8, 165.5, 164.2, 133.7, 133.1, 127.6, 126.6, 125.8, 122.8, 103.5, 64.4. HR-MS: calcd for C_11_H_8_N_1_O_3_ [M-H]^−^: 202.0510; found: 202.0510. Purity was determined by qNMR using maleic acid as an internal standard: 94.6%.

(Z)-1-[3-({[(1,3-Dioxo-2,3-dihydroisoquinolin-4­(1H)-lidene)­methyl] amino}­methyl) phenyl]-N,N-dimethylmethanaminium chloride TR-07*HCl. To a solution of *N*-[3-(aminomethyl)­benzyl]-*N*,*N*-dimethylaminedihydrochloride (700 mg, 2.95 mmol, 1.20 equiv) in MeOH (7.0 mL), Et_3_N (1.02 mL, 3.00 equiv) was added dropwise, stirred for 10 min, and then **TR-02** (500 mg, 2.46 mmol, 1.0 equiv) was added and stirred for 1 h under microwave conditions (20 mL vessel, 110 °C, 300 W). Three separate batches were combined and the solvent was removed under reduced pressure. The remaining residue was resuspended in EtOAc (50.0 mL), treated with 4 *M* HCl in dioxane, and stirred overnight at room temperature to obtain the corresponding hydrochloride. The solvent was removed under reduced pressure and the crude product (max. 400 mg) was dissolved in 8.0 mL of 5% MeCN in H_2_O and purified by preparative HPLC through several runs, yielding altogether 1.629 g (4.38 mmol, 59%) of product TR-07*HCl as a white to pale beige solid. mp 256.3 °C. ^1^H NMR (500 MHz, DMSO-*d*_6_, 300 K): δ_H_ (ppm) = 10.97 (br s, 2H), 10.66 (dt, ^3^ *J* = 13.0 Hz, ^3^ *J* = 6.4 Hz, 1H), 8.67 (d, ^3^ *J* = 13.2 Hz, 1H), 7.98 (dd, ^3^ *J* = 8.0 Hz, ^4^ *J* = 1.4 Hz, 1H), 7.86 (d, ^3^ *J* = 8.3 Hz, 1H), 7.59–7.56 (m, 2H), 7.55 (td, ^3^ *J* = 8.2 Hz, 7.7 Hz, ^4^ *J* = 1.4 Hz, 1H), 7.48 (t, ^3^ *J* = 7.7 Hz, 1H), 7.47 (dd, ^4^ *J* = 2.2 Hz, 0.9 Hz, 1H), 7.17 (td, ^3^ *J* = 7.6 Hz, ^4^ *J* = 0.9 Hz, 1H), 4.76 (d, ^3^ *J* = 6.3 Hz, 2H), 4.27 (s, 2H), 2.67 (s, 3H). ^13^C NMR (125 MHz, DMSO-*d*_6_, 300 K): δ_C_ (ppm) = 166.1, 163.9, 154.1, 139.0, 137.6, 132.9, 130.9, 130.2, 130.0, 129.2, 128.5, 127.5, 123.0, 120.2, 118.7, 92.3, 59.2, 52.0, 41.4 (2C). HR-MS: calcd for C_20_H_22_N_3_O_2_ [M]^+^: 336.1707; found: 336.1700. Elemental analysis calcd (%) for C_20_H_22_Cl_1_N_3_O_2_ x 0.75 H_2_O: C: 62.33, H: 6.15, N: 10.90; found: C: 62.31, H: 6.36, N: 10.89. Purity was determined by qNMR using maleic acid as an internal standard: 100.5%.

***2-{3-[(Dimethylamino)­methyl]­phenyl}­phenanthridin-6­(5H)-One TR-033***. In a preheated round-bottom flask, **TR-027** (490 mg, 1.79 mmol, 1.00 equiv), {3-[(dimethylamino)­methyl]­phenyl}­boronic acid hydrochloride (500 mg, 2.32 mmol, 1.30 equiv), and K_2_CO_3_ (742 mg, 5.37 mmol, 3.00 equiv) were suspended in a mixture of dioxane/H_2_O (4:1) under argon. Then, tetrakis­(triphenylphosphine)­palladium(0) (104 mg, 5 mol %) was added, and the solvent was degassed again with argon. The mixture was heated to 100 °C and stirred under reflux until the reaction was complete, as indicated by TLC. After cooling to room temperature, the reaction mixture was filtered over a Celite pad, washed with MeOH (3 × 15 mL) and the solvent was removed under reduced pressure. The crude product was adsorbed on silica gel and purified by MPLC (5–15% MeOH in DCM over 20 CV), yielding 141 mg (0.43 mmol, 24%) of **TR-033** as a pale brown solid. mp 198.1 °C. ^1^H NMR (400 MHz, DMSO-*d*_6_, 300 K): δ_H_ (ppm) = 11.75 (s, 1H), 8.70 (d, ^3^ *J* = 8.2 Hz, 1H), 8.61 (d, ^4^ *J* = 1.8 Hz, 1H), 8.35 (dd, ^3^ *J* = 8.0 Hz, ^4^ *J* = 1.4 Hz, 1H), 7.87 (ddd, ^3^ *J* = 7.6 Hz, ^4^ *J* = 1.4 Hz, 1H), 7.80 (dd, ^3^ *J* = 8.5 Hz, ^4^ *J* = 1.8 Hz, 1H), 7.71 (s, 1H), 7.70–7.69 (m, 1H), 7.66 (dd, ^3^ *J* = 8.0 Hz, 7.4 Hz, 1H), 7.46 (d, ^3^ *J* = 8.5 Hz, 1H), 7.43 (dd, ^3^ *J* = 8.2 Hz, 7.6 Hz, 1H), 7.29 (d, ^3^ *J* = 7.6 Hz, 1H), 3.49 (s, 2H), 2.19 (s, 6H). ^13^C NMR (100 MHz, DMSO-*d*_6_): δ_C_ (ppm) = 160.7, 139.6, 139.5, 135.9, 134.3, 134.2, 132.7, 128.7, 128.2, 128.0, 127.6, 127.4, 127.0, 125.8, 125.4, 123.0, 121.0, 117.9, 116.7, 63.4, 44.9 (2C). HR-MS: calcd for C_22_H_21_N_2_O_1_ [M + H]^+^: 329.1648; found: 329.1639. Purity was determined by qNMR using maleic acid as an internal standard: 93.7%.

**(** *Z* **)-** *N-* **(3-{[(1,3-Dioxo-2,3-dihydroisoquinoline-4­(1** *H* **)-ylidene)­methyl]­amino}­benzyl)-** *N* -ethylethanaminium chloride TR-036*HCl. To a solution of ICTR-122*2HCl (1.869 g, 7.44 mmol, 1.30 equiv) in MeOH (50.0 mL), Et_3_N (2.58 mL, 3.25 equiv) was added dropwise and stirred for 10 min. Subsequently, **TR-02** (1.162 g, 5.72 mmol, 1.00 equiv) was added, and the mixture was stirred at room temperature. After 6 h (no starting material remained according to TLC), the solvent was removed under reduced pressure. The remaining residue was resuspended in dioxane (50.0 mL), treated with 4 *M* HCl (dioxane), and stirred overnight at room temperature to obtain the corresponding hydrochloride. The solvent was removed under reduced pressure, and the crude product was dissolved in 8.0 mL of 5% MeCN in H_2_O and purified by preparative HPLC through several runs (max. 400 mg crude product per run), yielding 1.46 g (3.78 mmol, 66%) of product TR-036*HCl as a yellow solid. mp 248.6 °C. ^1^H NMR (500 MHz, DMSO-*d*_6_, 300 K): δ_H_ (ppm) = 12.36 (d, ^3^ *J* = 12.3 Hz, 1H), 11.34 (s, 1H), 11.02 (br s, 1H), 9.01 (d, ^3^ *J* = 12.3 Hz, 1H), 8.34 (d, ^3^ *J* = 8.3 Hz, 1H), 8.12 (dd, ^4^ *J* = 2.3 Hz, 1.7 Hz, 1H), 8.03 (dd, ^3^ *J* = 8.0 Hz, ^4^ *J* = 1.2 Hz, 1H), 7.60 (ddd, ^3^ *J* = 7.9 Hz, 7.6 Hz, ^4^ *J* = 1.4 Hz, 1H), 7.58 (ddd, ^3^ *J* = 8.0 Hz, ^4^ *J* = 2.0 Hz, ^4^ *J* = 1.2 Hz, 1H), 7.47 (dd, ^3^ *J* = 8.0 Hz, 7.7 Hz, 1H), 7.37 (dd, ^3^ *J* = 7.6 Hz, ^4^ *J* = 1.2 Hz, 1H), 7.30 (ddd, ^3^ *J* = 7.6 Hz, ^4^ *J* = 1.1 Hz, 1H), 4.32 (d, ^3^ *J* = 5.7 Hz, 2H), 3.13–3.03 (m, 4H), 1.29 (t, ^3^ *J* = 7.3 Hz, 6H). ^13^C NMR (125 MHz, DMSO-*d*_6_, 300 K): δ_C_ (ppm) = 166.9, 163.8, 144.4, 139.6, 136.3, 133.2, 132.1, 130.0, 127.5, 126.6, 124.5, 121.1, 120.3, 119.4, 118.7, 96.2, 54.7, 45.7 (2C), 8.10 (2C). HR-MS: calcd for C_21_H_24_N_3_O_2_ [M]^+^ 350.1863; found: 350.1857. Elemental analysis calcd (%) for C_21_H_24_Cl_1_N_3_O_2_ x 0.75 H_2_O: C: 63.15, H: 6.44, N: 10.52; found: C: 63.12, H: 6.23, N: 10.47. Purity was determined by qNMR using maleic acid as an internal standard: 98.8%.

(Z)-2-(2-{[(1,3-Dioxo-2,3-dihydroisoquinolin-4­(1H)-ylidene)­methyl]­amino}­phenyl)-N,N-dimethylethan-1-aminium chloride ICTR-059*HCl. In a microwave vessel (20 mL), **TR-02** (152 mg, 0.75 mmol, 1.00 equiv) and crude **ICTR-056** (160 mg, 0.97 mmol, 1.30 equiv) were suspended in MeOH (5.00 mL) and stirred in the microwave reactor (125 °C, 300 W) for 30 min. After cooling to room temperature, the solvent was subsequently removed under reduced pressure, and the crude product was adsorbed on silica gel. Purification by MPLC (0–20% MeOH in DCM over 35 min) gave rise to **ICTR-059** (209 mg, 0.62 mmol) as a yellow solid in an 83% yield. This was then converted to the corresponding hydrochloride ICTR-059*HCl by stirring in 2 M HCl in Et_2_O. mp 245.2 °C. ^1^H NMR (500 MHz, DMSO-*d*_6_, 300 K): δ_H_ (ppm) = 12.78 (d, ^3^ *J* = 12.0 Hz, 1H), 11.40 (s, 1H), 11.01 (br s, 1H), 8.97 (d, ^3^ *J* = 12.0 Hz, 1H), 8.20 (d, ^3^ *J* = 8.3 Hz, 1H), 8.04 (dd, ^3^ *J* = 7.7 Hz, ^4^ *J* = 1.2 Hz, 1H), 7.90 (d, ^3^ *J* = 7.7 Hz, 1H), 7.63 (ddd, ^3^ *J* = 7.9 Hz, 7.6 Hz, ^4^ *J* = 1.4 Hz, 1H), 7.41 (dd, ^3^ *J* = 8.3 Hz, 7.7 Hz, 1H), 7.37 (d, ^3^ *J* = 7.5 Hz, 1H), 7.29 (t, ^3^ *J* = 7.5 Hz, 1H), 7.20 (t, ^3^ *J* = 7.5 Hz, 1H), 3.31 – 3.28 (m, 2H), 3.19 – 3.15 (m, 2H), 2.86 (s, 6H). ^13^C NMR (125 MHz, DMSO-*d*_6_, 300 K): δ_C_ (ppm) = 167.3, 163.7, 145.6, 137.6, 136.3, 133.2, 130.6, 128.5, 127.5, 125.8, 124.9, 124.4, 120.9, 120.1, 117.0, 96.1, 55.3, 42.0 (2C), 25.4. HR-MS: calcd for C_20_H_22_N_3_O_2_ [M + H]^+^: 336.1707; found: 336.1714. Elemental analysis calcd (%) for C_20_H_22_ClN_3_O_2_ x 1.0 H_2_O: C: 61.61, H: 6.20, N: 10.78; found: C: 61.77, H: 6.19, N: 10.67.

### Hydrogen/Deuterium Exchange Mass Spectrometry (HDX-MS)

Purified FLAG-tagged PRMT1 (195 μL) was mixed with 5 μL 25 mM TR-07 (experiment 1) or TR-036 (experiment 2) dissolved in DMSO yielding 50 μM and 625 μM final concentrations, respectively. For the apo control the same volume of DMSO was employed as mock (SI Data S1).

Individual HDX reactions were prepared from these sample stocks with a two-arm robotic autosampler (LEAP Technologies). The FLAG-tagged PRMT1 sample (7 μL) was diluted 10-fold with a buffer (20 mM HEPES-Na pH 8.0, 50 mM NaCl, 1 mM EDTA, 0.5 mM DTT, 2.5% (v/v) DMSO) prepared with 99.9% D_2_O. In the respective ligand-containing states, TR-07 or TR-036 was supplemented to this exchange buffer at 625 μM to prevent dilution. After incubation at 25 °C for different time intervals (10, 30, 100, 1,000, and 10,000 s) the reaction was quenched at 1 °C by mixing with an equal volume of 400 mM KH_2_PO_4_/H_3_PO_4_ + 2 M Guanidine-HCl pH 2.2, and subsequently injected into an ACQUITY UPLC M-Class System with HDX Technology (Waters). The samples were directed to a column containing immobilized porcine pepsin operating at 12 °C with H_2_O + 0.1% (v/v) formic acid at 100 μL/min flow rate and the resulting peptides trapped on a short C18 column kept at 0.5 °C. After 3 min of digestion and trapping, peptides were separated on an ACQUITY UPLC BEH C18 1.7 μm 1.0 × 100 mm column (Waters) operating at 0.5 °C by employing a gradient of H_2_O + 0.1% (v/v) formic acid (eluent A) and acetonitrile +0.1% (v/v) formic acid (eluent B) at 60 μL/min flow rate as follows: 0–7 min/95–65% A, 7–8 min/65–15% A, 8–10 min/15% A. Peptides were ionized by electrospray ionization (capillary temperature 250 °C, spray voltage 3.0 kV) and mass spectra acquired over a range of 50 to 2,000 *m*/*z* in enhanced high definition MS (HDMS^E^) or high definition MS (HDMS) mode for nondeuterated and deuterated samples, respectively, on a G2-Si HDMS mass spectrometer with ion mobility separation (Waters). [Glu1]-Fibrinopeptide B standard (Waters) was employed for lock mass correction. Three technical replicates (individually prepared HDX reactions) were measured for each incubation time. Nondeuterated samples were prepared similarly with H_2_O-based buffers. Data were analyzed using ProteinLynx Global SERVER (PLGS) version 3.0.1 and DynamX 3.0 softwares (both Waters) as described previously.^76^

### Cell Lines, Reagents, and Plasmids

Human cell lines (PaTu8988T, PANC-1) were obtained from ATCC (American Type Culture Collection) and cultured in DMEM supplemented with 10% fetal calf serum (FCS, Gibco/BRL), 1% penicillin/streptomycin at 37 °C and 5% CO_2_. Sf9 (*Spodoptera frugiperda* clone 9) insect cells were grown in suspension (at 120 rpm rotation) or adherent culture in Sf-900 II serum-free medium (SFM) at 27 °C and optimal cell density of 0.5–2 × 10^6^ cells/mL. For PRMT1 inhibition, cells were treated with type I PRMT inhibitor MS023 hydrochloride (Biomol). For induction of genotoxic stress, cells were treated with gemcitabine (LC Laboratories). PBS-, HEPES- or Tris-buffered solutions of DMSO-solved compounds (e.g., TR-07, TR-033, TR-036, and ICTR-059) at 100–500 μM displayed a neutral pH of 7–8. The following antibodies were used: rabbit anti-PRMT1 (07-404, Millipore), mouse anti-FLAG (F3165, Sigma-Aldrich), mouse anti-His-HRP (A7058, Sigma-Aldrich), rabbit anti-ADMA (13522, Cell Signaling), mouse anti-CK8/18 (sc-52325, Santa Cruz), mouse anti-VINCULIN (sc-73614, Santa Cruz), mouse anti-β-ACTIN (A5441, Sigma-Aldrich), donkey anti-rabbit IgG-HRP (NA934, Amersham Biosciences), sheep anti-mouse IgG-HRP (NXA931, Amersham Biosciences), goat anti-mouse IgG-DyLight488 (115–545–062, Jackson ImmunoResearch), and goat anti-rabbit IgG-Cy3 (111–165–045, Jackson ImmunoResearch). The following plasmids were used: pFASTBAC-FLAG-rat PRMT1, pFASTBAC-FLAG-mouse PRMT4,^77^ and pGEX-GAR.^78^ The complete ORFs of human PRMT3 and PRMT6 were inserted into the pFASTBAC HT-3xFLAG B vector via *Bam*HI/*Hin*dIII and *Bam*HI/XhoI sites, respectively. The ORF of human PRMT1 (variant 5)^79^ was inserted into the pRSETA vector via *Bam*HI/*Eco*RI.

### Recombinant Protein Purification from Sf9 Cells and Bacteria

For PRMT protein expression in insect cells, recombinant baculovirus (bacmid) was generated using the Bac-to-Bac baculovirus system (Invitrogen). Sf9 cells were transfected with bacmid DNA using X-tremeGENE HP DNA transfection reagent (Sigma-Aldrich) according to the manufacturer's instructions. After 3–5 days, supernatants were collected, filtered, and used to infect Sf9 cells to amplify the virus. After 2 rounds of virus amplification and estimation of the final P_3_ virus titer, the P_3_ virus (2–5 μL/10^6^ cells) was applied for large-scale infection of 200 × 10^6^ Sf9 cells in suspension culture at a density of 1 × 10^6^ cells/mL. Cells were harvested 5–6 days post-infection by centrifugation at 500 g for 5 min at room temperature. Protein purification from baculovirus-infected Sf9 cells was performed as previously described.^18,24^ Cells were lysed in BC buffer (20 mM HEPES pH 7.9, 250 mM NaCl, 10% (v/v) glycerin, 0.4 mM EDTA, 1 mM DTT) containing a protease inhibitor cocktail (Roche) by 3 freeze-thaw cycles. After centrifugation at 11,000 rpm (Sorval SS34 rotor) and 4 °C for 1 h, the supernatant (whole cell protein extract) was incubated with anti-FLAG M2 Affinity Gel (Cytiva) at 4 °C and under agitation for 4 h. Subsequently, beads were washed in BC buffer containing various NaCl concentrations (once in BC with 1 M NaCl, twice in BC with 500 mM NaCl, twice in BC with 250 mM NaCl and twice in BC with 100 mM NaCl). FLAG-tagged proteins were either maintained bead-bound as a 50% (v/v) slurry in BC buffer with 100 mM NaCl and stored at −80 °C or were eluted in the appropriate elution buffer (depending on the subsequent assay) containing 0.25 mg/mL 3xFLAG peptides (Sigma-Aldrich) at 4 °C for 30–45 min. To remove the FLAG-peptides from the eluate, dialysis was performed in elution buffer containing 10% (v/v) glycerol at 4 °C for 4–6 h using Slide-A-Lyzer Dialysis Devices (Thermo Fisher Scientific) according to the manufacturer’s recommendations.

For PRMT1 protein expression in bacteria, the pRSET plasmid encoding human PRMT1 was transformed in *E. coli* BL21. Bacteria were cultivated in the presence of 1 mM IPTG at 30 °C for 3 h, followed by an overnight incubation at room temperature. Cells were harvested and lysed in TC250 buffer (50 mM HEPES pH 7.9, 250 mM NaCl, 15% (v/v) glycerin, 1 mM DTT) containing a protease inhibitor cocktail (Roche) by 3 freeze-thaw cycles. The lysate was sonicated 10 times for 20 s at 10% amplitude (Branson W-250 D sonifier) and then centrifuged at 15,000 g (Sorval SS34 rotor) and 4 °C for 1 h. The supernatant was incubated with Ni-NTA beads (Qiagen) in the presence of 30 mM imidazole at 4 °C overnight. Subsequently, beads were washed in TC250 buffer containing 100 mM imidazole. Bead-bound His-tagged PRMT1 was eluted in TC250 buffer containing 1 M imidazole at 4 °C for 1–2 h. To remove the imidazole, the eluates were dialyzed twice in dialysis buffer (50 mM Tris/HCl pH 8.0, 50 mM NaCl, 1 mM EDTA, 0.5 mM DTT) at 4 °C for 4 h using Slide-A-Lyzer Dialysis Devices (Thermo Fisher Scientific) according to the manufacturer’s recommendations. For GST-GAR protein expression in bacteria, pGEX-GAR transformed bacteria were induced with 1 mM IPTG at 37 °C for 3 h. Cells were harvested and lysed in PBS containing 1% (v/v) Triton X-100 and protease inhibitors (aprotinin, leupeptin, PMSF; Sigma-Aldrich). Lysates were sonicated and centrifuged. The supernatant was incubated with glutathione-agarose beads (Machery and Nagel) at 4 °C overnight. After washing, bead-bound GST-GAR was eluted (50 mM Tris/HCl pH 8.0; 25 mM glutathione) and dialyzed (Slide-A-Lyzer Mini Dialysis Units; Thermo Fisher Scientific) in PBS containing 10% (v/v) glycerol. Expression, purification, elution, and concentration of the respective recombinant proteins were analyzed by SDS-PAGE and Coomassie staining (Coomassie Brilliant Blue G250; Fluka) or immunoblotting.

### Fluorescence-Based in Vitro PRMT Assay (F-PRMT Assay)

The F-PRMT-assay published by Kramer et al. (2018) is composed of two consecutive reaction steps.^26^ In the first step, the fluorogenic substrate Nα-Benzoyl-L-arginine-7-amido-4-methylcoumarin (Bz-Arg-AMC, Sigma-Aldrich) undergoes methylation by PRMTs upon the addition of the cofactor SAM. In the second reaction step, trypsin cleaves the nonmethylated substrate, leading to the generation of the 7-amino-4-methylcoumarin product, which can be quantified by subsequent fluorescence measurement (excitation: 355 nm, emission: 460 nm).^26^ The first reaction was conducted with bead-bound PRMTs at a total volume of 100 μL. Each condition was performed as a technical triplicate to determine the mean and the standard deviation. The reaction mixtures, comprising 2 μM PRMT in assay buffer (50 mM Tris/HCl pH 8.0, 150 mM NaCl), 100 μM substrate Bz-Arg-AMC (dissolved in DMSO), 2.5% (v/v) glycerol, and 0.009% (v/v) Triton X-100, were incubated with DMSO (solvent control) or 1 μL PRMT modulator at various concentrations (dissolved in DMSO) at room temperature and under agitation (1,000 rpm) for 30 min protected from light. To start the methylation reaction, 10 μL SAM (625 μM final concentration, dissolved in assay buffer) were added. For the negative controls, SAM was substituted with assay buffer. The incubation was continued at 37 °C and under agitation (1,000 rpm) for a duration of 2 h. Subsequently, the respective PRMT modulator was also added to the reactions, which were initially without modulator, for an additional 30 min incubation at room temperature and under agitation to control for potential fluorescence activity of the compounds, which could interfere with the later fluorescence measurements. The enzymes were then immediately inactivated by incubating the reactions at 95 °C for 5 min. For the substrate cleavage by trypsin, 80 μL of each reaction was transferred into a black flat-bottom 96-well plate (Thermo Fisher Scientific). At first, the mean background fluorescence intensity (μ_b_) was quantified using a spectrophotometer (Mithras LB 940, Berthold) based on 3 independent measurements with 1 min measuring interval each. By addition of 20 μL trypsin (1 mg/mL, dissolved in nuclease-free water) to each well, the second reaction step was initiated. The fluorescence intensity was measured for 1 h at a rate of 1 measurement per minute. The constant fluorescence intensity μ_c_ was calculated as the mean of the last 20 data points. Then, μ_b_ was subtracted from μ_c_ for each well (μ_c_-μ_b_). Mean fluorescence (μ) and standard deviation (σ) were determined for the triplicates of all conditions. To assess the assay reliability, the Z-factor was calculated using the following formula: Z = 1 – $\frac{\left(3\sigma_{c}+3\sigma_{c-}\right)}{\left|\mu_{c}-\mu_{c-}\right|}$ , with the mean fluorescence (μ) and standard deviation (σ) of both the positive control c (+SAM) and the negative control c– (−SAM). For a reliable assay, the Z-factor should fall within the range of 0.5 and 1. The compound-treated enzyme activities of reliable assays were calculated (based on the mean fluorescence intensities) in % relative to the solvent-treated control,which was set to 100%, using the following formula: Activity [%] = $\frac{\mu_{c-}-f_{i}}{\mu_{c-}-\mu_{c}}\times 100$ , with f_i_ representing the measured fluorescence intensity following the application of a small-molecule modulator. Subsequently, the % mean activity upon modulator application and the standard deviation (SD) were determined from at least 3 repetitions (*n* ≥ 3). However, the number of repetitions in certain experiments varied between compounds, ranging from 3 to 6, because compounds that exhibited novel and intriguing effects were prioritized for the testing. Additionally, the inclusion of new compound batches necessitated additional testing of a compound. To calculate the half-maximal effective concentration (EC_50_ values) for the PRMT1 activator and the PRMT inhibitors, the compounds were tested at 4–8 different concentrations. Compound-treated enzyme activities in % were plotted against the logarithm of the compound concentrations. Concentration-response curves were fitted by nonlinear regression in GraphPad Prism, using a four-parameter logistic model according to the following formular: $E=E_{\min}+\frac{E_{\max}-E_{\min}}{1+10^{\left(\log_{10}{\mathrm{EC}}_{50}-\log_{10}C\right)\cdot h}}$ , with the % effect (E) activation/inhibition, the compound concentration (C), and the Hill slope (h). EC_50_ values are reported as mean from 3 experiments (n = 3), with the coefficients of determination (R^2^) indicated.

### Radioactive in Vitro Methyltransferase Assays

For the autoradiography-based methyltransferase assays, the reaction mixtures, containing 1–2 μg recombinant FLAG-tagged PRMTs or His-tagged PRMT1 and as the substrates 5–10 μg bulk histones from calf thymus (Sigma-Aldrich) or 1–2 μg GST-GAR protein in PBS at a total volume of 20 μL, were preincubated in the absence (DMSO) or presence of compounds at room temperature and under agitation (800 rpm) for 30 min. The enzymatic reactions were initiated by addition of 0.02 μCi [14C-methyl]-SAM (20 μCi/mL; PerkinElmer) and performed at 37 °C for 1–2 h. Reactions were separated by SDS-PAGE, blotted, and analyzed by autoradiography using X-ray films (Hyperfilm; Amersham) and intensifying screens (Kodak) at −80 °C. For Ponceau S staining, the blot membranes were incubated in Ponceau S solution (Sigma-Aldrich) at room temperature for 10–15 min and subsequently rinsed with distilled water until the background was fully destained.

For the liquid methyltransferase assays, the reaction mixtures, containing 1–2 μg eluted FLAG-tagged PRMT1 and as the substrate 1–2 μg eluted GST-GAR protein at a total volume of 20 μL in PBS, were preincubated in the absence (DMSO) or presence of compounds at room temperature and under agitation (800 rpm) for 30 min. The enzymatic reactions were initiated by addition of 0.02 μCi [14C-methyl]-SAM (20 μCi/mL; Revvity) and performed at 37 °C for 5 min. Subsequently, GST-GAR was collected at room temperature for 1 h using glutathione-sepharose beads (Cytiva) in PBS containing 0.05% Tween 20 and 100 μM MS023 to terminate the reactions. After washing, 1 mL scintillation liquid (Rotiszint ecoplus, Roth) was added to the bead-bound GST-GAR and incorporated ^14^C-methyl groups were quantified using a scintillation counter (LS6500, Beckman Coulter). Background signal was determined using 1 mL of scintillation fluid alone and was subtracted from the measured counts of the enzymatic reactions. Compound-treated PRMT1 activity was expressed as background-corrected counts (in cpm) normalized to the DMSO control.

### Pulldown Assay

To evaluate the effect of small-molecule PRMT1 modulators on the dimerization of His-tagged and FLAG-tagged PRMT1, pulldown assays were conducted. For the binding reactions, 1 μg of bead-bound FLAG-tagged PRMT1 was preincubated with or without compounds at a total volume of 200 μL IPH buffer (50 mM Tris/HCl pH 8.0, 5 mM EDTA, 0.5% (v/v) NP-40, 150 mM NaCl) containing protease inhibitors (leupeptin, aprotinin, PMSF; Sigma-Aldrich) at 4 °C and under gentle agitation for 30 min. Subsequently, 0.02 μg His-tagged PRMT1 was added per reaction and incubated at 4 °C and under gentle agitation for 15 min. Finally, the beads were washed 3 times in IPH buffer containing 400 mM NaCl and analyzed by SDS-PAGE and immunoblotting.

### Protein Extraction, SDS-PAGE, and Immunoblotting

For protein extraction, human cell lines were lysed in IPH buffer (50 mM Tris/HCl pH 8.0, 5 mM EDTA, 0.5% (v/v) NP-40, 150–400 mM NaCl) containing protease inhibitors (leupeptin, aprotinin, PMSF; Sigma-Aldrich). Protein concentrations were measured using Bradford reagent and BSA (Sigma-Aldrich) as a standard. For immunoblotting, usually 10–50 μg protein of the total cell lysates were separated by SDS-PAGE on 7.5–15% (w/v) polyacrylamide gels and transferred onto PVDF membrane (Amersham). Blot membranes were blocked in TBS (50 mM Tris/HCl pH 7.9, 150 mM NaCl) containing 0.1% (v/v) Tween and 4% (w/v) milk powder or 5% (w/v) BSA. Subsequently, primary antibodies were diluted in blocking solution and incubated for 1–2 h at temperature to overnight at 4 °C. After washing, blots were stained with HRP-coupled secondary antibodies. Chemiluminescence reaction (Millipore) and X-ray films (Kodak) were used to detect antibody binding. Quantification of protein bands was conducted using Fiji ImageJ.

### Cell Viability Assays

Cell viability was assessed using the CellTiter-Glo assay (Promega) according to the manufacturer’s instructions. Briefly, PANC-1 and PaTu8988T cells were seeded as triplicates in 96 well plates (5 × 10^3^ cells/well) and allowed to adhere for 3–4 h, followed by treatment with the compounds at 11–12 concentrations spanning 0.028–120 μM for 96 h, with DMSO (solvent) treated cells serving as control. Subsequently, cell viability was quantified by measuring luminescence on a microplate reader, and the signal of compound-treated cells was normalized to the signal of the solvent-treated control, which was set to 100%. Cell viability in % was plotted against the logarithm of the compound concentration, and concentration-response curves were fitted by nonlinear regression using a three-parameter logistic model to determine half-maximal inhibitory concentrations (IC_50_). Curve fitting was performed in GraphPad Prism, and IC_50_ values are reported as mean from 3 experiments (n = 3), with the coefficients of determination (R^2^) indicated.

### Apoptosis Assays

For quantification of apoptosis using the CaspaseGlo 3/7 assay (Promega), PANC-1 and PaTu8988T cells were seeded in triplicates in white 96-well plate (5 × 10^3^ cells/well) and cultured in the absence or presence of compounds. After 2 h, as soon as the cells had settled, gemcitabine was added or not, and cells were incubated for another 72 h. Subsequently, the caspase3/7 activity was measured using the luminescence-based CaspaseGLO-3/7 assay (Promega). To this end, the CaspaseGlo reagent was subsequently added to the cells at a 1:1 ratio and incubated at room temperature and under agitation for 30 min protected from light. The luminescence measurements were carried out using a microplate reader (Mithras LB 940, Berthold).

For quantification of apoptosis by Fluorescence Activated Cell Sorting (FACS), PANC-1 and PaTu8988T cells were seeded in 6-well plates (250 × 10^3^ cells/well) and cultured in the absence or presence of compounds. After 24 h, gemcitabine was added or not for another 48 h. After harvesting and washing, unfixed cells (0.5–1 × 10^6^) were incubated with FITC-labeled Annexin V and/or PI (BD Pharmingen) according to the manufacturer’s instructions. All samples were analyzed using a BD FACSCalibur flow cytometer (BD Bioscience). Data were acquired using the CellQuestPro program and analyzed with the FlowJo (FlowJo LLC) software.

### Mouse Xenograft Model

As recipient mice for transplantation of human PDAC cells, adult female immune-deficient athymic nude mice (aged 9–15 weeks, weighing 20–30 g) were purchased from Janvier Laboratories. Tumor growth was initiated upon subcutaneous injection of 2 × 10^6^ PANC-1 cells resuspended in 100 μL PBS into the right flank of the recipients. Single and combined treatments with the PRMT1 activator TR-07 and gemcitabine as well as no treatment (control group) was initiated 6 days post-engraftment, when the tumor nodules had formed. The administered doses were 150 mg/kg TR-07 daily and 10 mg/kg gemcitabine every second day. The treatment continued for a duration of 14 days, during which parameters such as body condition score (BCS), body weight, tumor size, health status and physical condition of the mice were closely monitored on a daily basis. Tumor sizes were measured every second day using a caliper, and tumor volumes were subsequently calculated using the following formula: V [mm^3^] = (length×width^2)2 . At the end of the 2 weeks, the experiment was terminated, the mice were sacrificed and tumors were finally dissected. All animal experiments were conducted in accordance with the ethical guidelines and were approved by the Regierungspräsidium Giessen (TVA G14/2023) and the University Marburg.

### Immunofluorescence (IF) Staining of Xenograft Tumor Tissue

For IF staining, sections (3 μm) of formalin-fixed and paraffin-embedded PANC-1 xenograft tissues from mice, which were treated for 2 weeks with PBS (vehicle control), TR-07, gemcitabine or a combination of TR-07 and gemcitabine, and subsequently sacrified, were heated to 60 °C for 1 h, deparaffinized using xylene and hydrated by a graded series of ethanol washes. For antigen retrieval, tissue sections were steam-heated for 35 min in citrate buffer (pH 6.0), cooled down to room temperature, and then washed in PBS. For reducing the background staining, sections were incubated twice 5 min in 100 mM glycine, followed by rinsing 2 times in TBS with 0.1% Tween (TBS-T), rinsing once in TBS, and incubation in blocking solution (PBS; 10% chicken serum; 0.3% Triton-X100) at room temperature for 60 min. Incubation with primary antibodies was performed in blocking solution with 5% chicken serum at 4 °C overnight. Subsequently, the sections were washed 3 times in TBS-T and blocked for 30 min in blocking solution followed by incubation with secondary antibodies and DAPI (0.15 mg/mL; Sigma-Aldrich) for 90 min at room temperature. After 3 additional washing steps in TBS-T and one washing step in water, the sections were embedded in mounting medium (Mowiol4–88 with DABCO, Roth) and stored at 4 °C. Fluorescence images were acquired using the Confocal Laser Scanning microscope Leica SP8i and analyzed with IMARIS 9.9.0 (Bitplane-Oxford Instruments, Abingdon, GB) using the Cell module. The mean intensity signal of ADMA-positive PANC-1 cells was quantified from 7 independent, randomly selected standardized areas (290 × 290 μm at 40-fold magnification) per xenograft tumor of 5 animals (n = 5) of each treatment cohort (PBS, TR-07, GEM and TR-07+GEM) with a minimum intensity threshold of 5.5 (arbitrary unit).

### Statistical Analysis

All experiments were independently performed at least 3 times (biological replicates). Error bars represent ± standard deviation (SD) of the mean, with the exception of the pEC_50_/pIC_50_ and the xenograft growth curves, for which ± SEM is displayed. The corresponding statistical tests are specified in the figure legends. For autoradiographs and immunostainings, reproducible and representative data sets are displayed.

## Supplementary Material









## Notes

Data S1: Additional data obtained by HDX-MS (XLSX)
Data S2: PDB Files of docking models for all series (ZIP)
Data S3: Molecular formular strings (SMILES) of all docked and synthesized compounds (CSV)
Additional biological data, high-resolution mass spectra of K002FT017/TR-07, unbiased docking calculations, and HDX-MS analyses (Figures S1–13); Overview of docking set-ups (Table S1); Summary of compounds of the PRMT4/PRMT6 inhibitor screen (Table S2); Summary of compounds of the SAR analyses (Table S3); Synopsis of the experimental details of all synthesized compounds HPLC tracks for lead compounds (Figures S14–S17); Summary of the NMR spectra of all synthesized compounds (Figures S18–S97) (PDF)
