# Supplementary material for: Small-Molecule Activators of PRMT1: Discovery, SAR Analyses, and Proapoptotic Effects in Pancreatic Cancer Cells
Source: J Med Chem. 2026 Aug 20;69(17):20116–37. doi: 10.1021/acs.jmedchem.5c03281 (PMC13576449; doi:10.1021/acs.jmedchem.5c03281)

Supporting Information (SI)

Small-Molecule Activators of PRMT1: Discovery, SAR Analyses, and Proapoptotic Effects in Pancreatic Cancer Cells

Sepideh Salehipour-Bavarsad [a,♦], Christian Iking [b,♦], Jonas Kammertöns [c,#], Caroline Bouchard [a,#], Marion Meixner [a,#], Michael Daude [b,#], Wieland Steinchen [d,e], Felix Terwesten [c], Johanna Senst [c], David Vonhören [a], Sinja Rakow [a], Anne Kagerhuber [a], Sona Martirosian [a], Katrin Roth [f], Daniel Hilger [c], Stefanie Dörr [c], Gina Bach [b], Van Tuan Trinh [e], Anett Hauser [e], Frank Abendroth [e], Olalla Vázquez [d,e], Gert Bange [d,e], Jörg Walter Bartsch [g], Detlef Klaus Bartsch [h], Peter Kolb [*,c,d], Wibke E. Diederich [*,b], Uta-Maria Bauer [*,a]

[a] Philipps-Universität Marburg, Institute for Molecular Biology and Tumor Research (IMT), BMFZ, Hans-Meerwein-Str. 2, 35043 Marburg, Germany.
[b] Philipps-Universität Marburg, Department of Medicinal Chemistry, Center for Tumor and Immune Biology (ZTI), Hans-Meerwein-Str. 3, 35043 Marburg, Germany.
[c] Philipps-Universität Marburg, Institute of Pharmaceutical Chemistry, Marbacher Weg 8, 35032 Marburg, Germany.
[d] Philipps-Universität Marburg, Center for Synthetic Microbiology (SYNMIKRO), Karl-von-Frisch-Str. 14, 35043 Marburg, Germany.
[e] Philipps-Universität Marburg, Department of Chemistry, Hans-Meerwein-Str. 4, 35043 Marburg, Germany.
[f] Philipps-Universität Marburg, Imaging Core Facility, Center for Tumor Biology and Immune Biology, Hans-Meerwein-Str. 3, 35043 Marburg, Germany.
[g] University Hospital Marburg, Department of Neurosurgery, Baldingerstraße, 35043 Marburg, Germany.
[h] University Hospital Marburg, Department of Visceral, Thoracic and Vascular Surgery, Baldingerstraße, 35043 Marburg, Germany.

Author Contributions

♦ These authors are joint first authors and contributed equally to the work.

\# These authors contributed equally to the work.

* These authors are co-corresponding.

Table of content

Figure S 1: Validation of the catalytic activity of recombinant PRMT enzymes. 8
Figure S 2: Establishment of the F-PRMT assay. 9
Figure S 3: Structure revision of K002FT017 and structure elucidation of TR-07. 10
Figure S 4: Unbiased docking calculations of a TR-07 subfragment to human PRMT1.................. 13
Figure S 5: Comparison of the docking pose of TR-036 in human PRMT1, focusing on the region encompassing E216, with the corresponding regions in PRMT3, PRMT4, and PRMT6. 14
Figure S 6: HDX-MS analysis of TR-07 binding to PRMT1... 15
Figure S 7: HDX-MS analysis of TR-036 binding to PRMT1... 16
Figure S 8: Impact of TR-033 and TR-036 on homodimerization of PRMT1................................. 17
Figure S 9: Quantification of the effect of TR-07 on the homooligomeric interaction of PRMT1 from biological replicates. 18
Figure S 10: Effect of PRMT1 activators TR-033, TR-036, and ICTR-059 on gemcitabine-induced apoptosis in PDAC cell lines. 19
Figure S 11: Effect of PRMT1 activators TR-07, TR-033, TR-036, and ICTR-059 on the viability of PaTu8898T cells. 20
Figure S 12: Monitoring of the body weight of mice bearing PANC-1 xenografts............................ 21
Figure S 13: Effect of TR-07 on the cellular ADMA levels in PANC-1 tumor xenografts................ 22
Table S1: Overview of docking setups. 23
Table S2: Summary of the remaining 59 compounds of the original docking screens with respect to their effects on the catalytic activity of the intended target PRMT, either PRMT4 or PRMT6. 24
Table S3: Summary of TR-07 and 45 derivates as well as new molecules, with respect to their effects on the catalytic activity of PRMT1 and other type I PRMTs. 30
General Working Procedure (GWP) 37
GWP 1: Nucleophilic Substitution under Microwave Irradiation 37
GWP 2: Iron Reduction of 3/4-Substituted Nitrobenzylamines 37
GWP 3: Iron Reduction of 2-Substituted Nitrobenzylamines 37
GWP 4: Reduction of the Nitro Group with Ammonium Formate 37
GWP 5: Reduction of the Nitrile Group with $LiAlH_4$ 38
GWP 6: Coupling with Precursor TR-02 38
Synthesis of K002FT017, alias TR-07[a] 39
Isoquinoline-1,3(2*H*,4*H*)-dione (TR-01) 39
(*E*/*Z*)-4-(Methoxymethylene)isoquinoline-1,3(2*H*,4*H*)-dione (TR-02).. 40
(*Z*)-1-[3-({[(1,3-Dioxo-2,3-dihydroisoquinolin-4(1*H*)-lidene)methyl] amino}methyl) phenyl]-*N*,*N*-dimethylmethanaminiumchloride TR-07*HCl 40
Structure-activity-relationship (SAR) analyses of TR-07... 41
Synthesis of the benzylic amine precursors and aniline derivatives 41
Series I: Reduction of molecular complexity.. 41
(*Z*)-4-[(Benzylamino)methylene]isoquinoline-1,3(2*H*,4*H*)-dione TR-03 41
(*Z*)-4-[(Phenylamino)methylene]isoquinoline-1,3(2*H*,4*H*)-dione TR-014....................................... 42
(*Z*)-4-({[3-(Dimethylamino)propyl]amino}methylene)isoquinoline-1,3(2*H*,4*H*)-dione TR-018 42
(*Z*)-4-[(Pyridin-2-ylamino)methylene]isoquinoline-1,3(2*H*,4*H*)-dione TR-030 42

(*Z*)-4-[(Pyridin-3-ylamino)methylene]isoquinoline-1,3(2*H*,4*H*)-dione TR-031 43
(*Z*)-4-{[(Pyridin-3-ylmethyl)amino]methylene}isoquinoline-1,3(2*H*,4*H*)-dione TR-032 43
Series II: Modifications in the Southern Region - benzylamine derivatives 44
Synthesis of ICTR-108 44
tert-Butyl [3-(aminomethyl)benzyl]carbamate ICTR-103 44
1-(3-{[(*tert*-Butoxycarbonyl)amino]methyl}phenyl)-*N*,*N*,*N*-trimethylmethanami-niumiodide ICTR-104 45
1-[3-(Ammoniomethyl)phenyl]-*N*,*N*,*N*-trimethylmethanaminiumdiiodide ICTR-105 45
(*Z*)-1-[3-({[(1,3-Dioxo-2,3-dihydroisoquinoline-4(1*H*)-ylidene)methyl]amino} methyl)phenyl]-*N*,*N*,*N*-trimethylmethanaminiumiodide ICTR-108 45
(*Z*)-4-({[2-(Dimethylamino)benzyl]amino}methylene)isoquinolin-1,3(2*H*,4*H*)-dione ICTR-052 45
Synthesis of ICTR-063 46
2-[(Dimethylamino)methyl]benzonitrile ICTR-046 46
1-[2-(Aminomethyl)phenyl]-*N*,*N*-dimethylmethanamin ICTR-062 46
(*Z*)-4-[({2-[(Dimethylamino)methyl)]benzyl}amino)methylene]isoquinoline-1,3(2*H*,4*H*)-dione ICTR-063 47
Synthesis of ICTR-119 47
2-[(Diethylamino)methyl]benzonitril ICTR-106 47
N-[2-(Aminomethyl)benzyl]-*N*-ethylethanamine ICTR-117 47
(*Z*)-4-[({2-[(Diethylamino)methyl]benzyl}amino)methylene]isoquinoline-1,3(2*H*,4*H*)-dione ICTR-119 48
Synthesis of ICTR-120 48
3-[(Diethylamino)methyl]benzonitrile ICTR-107 48
N-[3-(Aminomethyl)benzyl]-*N*-ethylethanamine ICTR-118 48
(*Z*)-4-[({3-[(Diethylamino)methyl]benzyl}amino)methylene]isoquinoline-1,3(2*H*,4*H*)-dione ICTR-120 49
(*Z*)-4-[({4-[(Dimethylamino)methyl]benzyl}amino)methylene]isoquinoline-1,3(2*H*,4*H*)-dione ICTR-078 49
Series III$_A$: Aniline Derivatives 50
(*Z*)-4-[({2-[(Dimethylamino)methyl]phenyl}amino)methylene]isoquinoline-1,3(2*H*,4*H*)-dione TR-022 50
(*Z*)-4-[({3-[(Dimethylamino)methyl]phenyl}amino)methylene]isoquinoline-1,3(2*H*,4*H*)-dione TR-010 50
(Z)-4-[({4-[(Dimethylamino)methyl]phenyl}amino)methylene]isoquinoline-1,3(2*H*,4*H*)-dione TR-021 51
(*Z*)-4-{[(4-Isobutylphenyl)amino]methylen}isoquinoline-1,3(2*H*,4*H*)-dione ICTR-076 51
Synthesis of TR-036*HCl 51
N-Ethyl-*N*-(3-nitrobenzyl)ethanamine ICTR-121 52
3-[(Diethylammonio)methyl]benzenaminiumdichloride ICTR-122*2HCl 52
(*Z*)-*N*-(3-{[(1,3-Dioxo-2,3-dihydroisoquinoline-4(1*H*)-ylidene)methyl]amino}benzyl)-*N*-ethylethanaminiumchloride TR-036*HCl 53
Synthesis of TR-038 54
4-(3-Nitrobenzyl)morpholine TR-029 54
3-(Morpholinomethyl)aniline TR-037 54
(*Z*)-4-({[3-(Morpholinomethyl)phenyl]amino}methylene)isoquinoline-1,3(2*H*,4*H*)-dione TR-038 54
Series III$_B$: Ortho substituted Aniline Derivatives 55

Synthesis of ICTR-022 55
N-Isopropyl-*N*-(2-nitrobenzyl)propan-2-amine ICTR-001 55
2-[(Diisopropylamino)methyl]aniline ICTR-017 55
(*Z*)-4-[({2-[(Diisopropylamino)methyl]phenyl}amino)methylene]isoquinoline-1,3(2*H*,4*H*)-dione ICTR-022 55
Synthesis of ICTR-023 56
1-(2-Nitrobenzyl)pyrrolidine ICTR-002 56
2-(Pyrrolidin-1-ylmethyl)aniline ICTR-021 56
(*Z*)-4-({[2-(Pyrrolidin-1-ylmethyl)phenyl]amino}methylene)isoquinoline-1,3(2*H*,4*H*)-dione ICTR-023 56
Synthesis of ICTR-028 57
1-(2-Nitrobenzyl)piperidin ICTR-003 57
2-(Piperidin-1-ylmethyl)aniline ICTR-024 57
(*Z*)-4-({[2-(Piperidin-1-ylmethyl)phenyl]amino}methylene)isoquinoline-1,3(2*H*,4*H*)-dione ICTR-028 57
Synthesis of ICTR-036 58
1-(2-Nitrobenzyl)-1*H*-pyrrole ICTR-008 58
2-[(1*H*-Pyrrol-1-yl)methyl]aniline ICTR-054 58
(*Z*)-4-[({2-[(1H-Pyrrol-1-yl)methyl]phenyl}amino)methylene]isoquinoline-1,3(2*H*,4*H*)-dione ICTR-036 58
Synthesis of ICTR-042 59
1-(2-Nitrobenzyl)-1*H*-1,2,4-triazole ICTR-011 59
2-[(1*H*-1,2,4-Triazol-1-yl)methyl]aniline ICTR-041 59
(*Z*)-4-[({2-[(1*H*-1,2,4-Triazol-1-yl)methyl]phenyl}amino)methylene]isoquinoline-1,3(2*H*,4*H*)-dione ICTR-042 59
Synthesis of ICTR-058*HCl 60
tert-Butyl 4-(2-nitrobenzyl)piperazine-1-carboxylate ICTR-004 60
tert-Butyl 4-(2-aminobenzyl)piperazine-1-carboxylate ICTR-029 60
tert-Butyl (*Z*)-4-(2-{[(1,3-dioxo-2,3-dihydroisoquinolin-4(1*H*)-ylidene)methyl]amino}benzyl)piperazine-1-carboxylate ICTR-034 60
(*Z*)-4-(2-{[(1,3-Dioxo-2,3-dihydroisoquinolin-4(1*H*)-ylidene)methyl]amino}benzyl)piperazin-1-ium chloride ICTR-058*HCl 61
Synthesis of ICTR-035 61
1-Methyl-4-(2-nitrobenzyl)piperazine ICTR-006 61
2-[(4-Methylpiperazin-1-yl)methyl]aniline ICTR-027 61
(*Z*)-4-[({2-[(4-Methylpiperazin-1-yl)methyl]phenyl}amino)methylene]isoquinoline-1,3(2*H*,4*H*)-dione ICTR-035 62
Synthesis of ICTR-040 62
4-Methyl-1-(2-nitrobenzyl)piperidine ICTR-005 62
2-[(4-Methylpiperidin-1-yl)methyl]aniline ICTR-026 62
(*Z*)-4-[({2-[(4-Methylpiperidin-1-yl)methyl]phenyl}amino)methylene]isoquinoline-1,3(2*H*,4*H*)-dione ICTR-040 63
Synthesis of ICTR-050 63
1-Ethyl-4-(2-nitrobenzyl)piperazine ICTR-039 63
2-[(4-Ethylpiperazin-1-yl)methyl]aniline ICTR-047 64
(*Z*)-4-[({2-[(4-Ethylpiperazin-1-yl)methyl]phenyl}amino)methylene]isoquinoline-1,3(2*H*,4*H*)-dione ICTR-050 64

Synthesis of ICTR-086*HCl 64
N-Ethyl-*N*-(2-nitrobenzyl)ethanaminium chloride ICTR-084*HCl 64
2-([Diethylamonio]methyl)benzenaminium dichloride ICTR-085*2HCl 65
(*Z*)-N-(2-{[(1,3-Dioxo-2,3-dihydroisoquinolin-4(1*H*)-ylidene)methyl]amino}benzyl)-*N*-ethyl-ethanaminium chloride ICTR-086*HCl 65
Synthesis of ICTR-060 66
N,*N*-Diethyl-2-nitroaniline ICTR-048 66
N,*N*-Diethylbenzen-1,2-diamin ICTR-057 66
(*Z*)-4-({[2-(Diethylamino)phenyl]amino}methylene)isoquinoline-1,3(2*H*,4*H*)-dione ICTR-060 66
Synthesis of ICTR-059*HCl 67
N,*N*-Dimethyl-2-(2-nitrophenyl)ethan-1-amine ICTR-044 67
2-[2-(Dimethylamino)ethyl]aniline ICTR-056 67
(*Z*)-2-(2-{[(1,3-Dioxo-2,3-dihydroisoquinolin-4(1H)-ylidene)methyl]amino}phenyl)-*N*,*N*-dimethylethan-1-aminium chloride ICTR-059*HCl 67
(*Z*)-4-{[(2-Aminophenyl)amino]methylene}isoquinoline-1,3(2*H*,4*H*)-dione ICTR-019 68
(*Z*)-4-({[2-(Dimethylamino)phenyl]amino}methylene)isoquinoline-1,3(2*H*,4*H*)-dione ICTR-015 68
(*Z*)-4-({[2-(2-Hydroxyethyl)phenyl]amino}methylene)isoquinoline-1,3(2*H*,4*H*)-dione ICTR-053 68
Series IV: Planar, rigid Ring System 70
2-Bromphenanthridin-6(5*H*)-one TR-027 70
2-{3-[(Dimethylamino)methyl]phenyl}phenanthridin-6(5*H*)-one TR-033 70
2-{3-[(Diethylamino)methyl]phenyl}phenanthridin-6(5*H*)-one ICTR-096 71
2-{2-[(Diethylamino)methyl]phenyl}phenanthridin-6(5*H*)-one ICTR-094 71
References 72
HPLC traces of compounds TR-07*HCl, TR-033, TR-036*HCl, and ICTR-059*HCl 73
Figure S 14: HPLC trace of TR-07*HCl 73
Figure S 15: HPLC trace of TR-033 73
Figure S 16: HPLC trace of TR-036*HCl 74
Figure S 17: HPLC trace of ICTR-059*HCl 74
Figure S 18: ^{1}H-NMR spectra (500 MHz) of TR-01 in DMSO-d_6 75
Figure S 19: ^{13}C-NMR (APT) spectra (125 MHz) of TR-01 in DMSO-d_6 77
Figure S 20: ^{1}H-NMR spectra (500 MHz) of TR-02 in DMSO-d_6 78
Figure S 21: ^{13}C-NMR (APT) spectra (125 MHz) of TR-02 in DMSO-d_6 80
Figure S 22: ^{1}H-NMR spectra (500 MHz) of TR-03 in DMSO-d_6 81
Figure S 23: ^{13}C-NMR (APT) spectra (125 MHz) of TR-03 in DMSO-d_6 83
Figure S 24: ^{1}H-NMR spectra (500 MHz) of TR-07*HCl in DMSO-d_6 84
Figure S 25: ^{13}C-NMR (APT) spectra (125 MHz) of TR-07*HCl in DMSO-d_6 86
Figure S 26: HMBC spectra of TR-07*HCl in DMSO-d_6 87
Figure S 27: ^{1}H-NMR spectra (500 MHz) of TR-010 in DMSO-d_6 88
Figure S 28: ^{13}C-NMR (APT) spectra (100 MHz) of TR-010 in DMSO-d_6 89
Figure S 29: ^{1}H-NMR spectra (500 MHz) of TR-014 in DMSO-d_6 90
Figure S 30: ^{13}C-NMR (APT) spectra (100 MHz) of TR-014 in DMSO-d_6 91
Figure S 31: 1D-NOESY-NMR spectra (500 MHz) of TR-014 in DMSO-d_6 92
Figure S 32: ^{1}H-NMR spectra (500 MHz) of TR-018 in DMSO-d_6 93
Figure S 33: ^{13}C-NMR (APT) spectra (125 MHz) of TR-018 in DMSO-d_6 94
Figure S 34: ^{1}H-NMR spectra (500 MHz) of TR-021 in DMSO-d_6 95
Figure S 35: ^{13}C-NMR (APT) spectra (125 MHz) of TR-021 in DMSO-d_6 96
Figure S 36: ^{1}H-NMR spectra (500 MHz) of TR-022 in DMSO-d_6 97

Figure S 37: ^{13}C-NMR (APT) spectra (125 MHz) of TR-022 in DMSO-d_6 98
Figure S 38: ^{1}H-NMR spectra (500 MHz) of TR-030 in DMSO-d_6 99
Figure S 39: ^{13}C-NMR (APT) spectra (125 MHz) of TR-030 in DMSO-d_6 101
Figure S 40: ^{1}H-NMR spectra (500 MHz) of TR-031 in DMSO-d_6 102
Figure S 41: ^{13}C-NMR (APT) spectra (100 MHz) of TR-031 in DMSO-d_6 104
Figure S 42: ^{1}H-NMR spectra (500 MHz) of TR-032 in DMSO-d_6 105
Figure S 43: ^{13}C-NMR (APT) spectra (125 MHz) of TR-032 in DMSO-d_6 106
Figure S 44: ^{1}H-NMR spectra (400 MHz) of TR-033 in DMSO-d_6 107
Figure S 45: ^{13}C-NMR (APT) spectra (100 MHz) of TR-033 in DMSO-d_6 108
Figure S 46: ^{1}H-NMR spectra (500 MHz) of TR-036*HCl in DMSO-d_6 109
Figure S 47: ^{13}C-NMR (APT) spectra (125 MHz) of TR-036*HCl in DMSO-d_6 110
Figure S 48: ^{1}H-NMR spectra (500 MHz) of TR-038 in DMSO-d_6 111
Figure S 49: ^{13}C-NMR (APT) spectra (125 MHz) of TR-038 in DMSO-d_6 112
Figure S 50: ^{1}H-NMR spectra (500 MHz) of ICTR-015 in DMSO-d_6 113
Figure S 51: ^{13}C-NMR (APT) spectra (100 MHz) of ICTR-015 in DMSO-d_6 114
Figure S 52: ^{1}H-NMR spectra (500 MHz) of ICTR-019 in DMSO-d_6 115
Figure S 53: ^{13}C-NMR (APT) spectra (100 MHz) of ICTR-019 in DMSO-d_6 116
Figure S 54: ^{1}H-NMR spectra (500 MHz) of ICTR-022 in DMSO-d_6 117
Figure S 55: ^{13}C-NMR (APT) spectra (100 MHz) of ICTR-022 in DMSO-d_6 118
Figure S 56: ^{1}H-NMR spectra (500 MHz) of ICTR-023 in DMSO-d_6 119
Figure S 57: ^{13}C-NMR (APT) spectra (100 MHz) of ICTR-023 in DMSO-d_6 120
Figure S 58: ^{1}H-NMR spectra (500 MHz) of ICTR-028 in DMSO-d_6 121
Figure S 59: ^{13}C-NMR (APT) spectra (125 MHz) of ICTR-028 in DMSO-d_6 122
Figure S 60: ^{1}H-NMR spectra (500 MHz) of ICTR-035 in DMSO-d_6 123
Figure S 61: ^{13}C-NMR (APT) spectra (100 MHz) of ICTR-035 in DMSO-d_6 124
Figure S 62: ^{1}H-NMR spectra (500 MHz) of ICTR-036 in DMSO-d_6 125
Figure S 63: ^{13}C-NMR (APT) spectra (100 MHz) of ICTR-036 in DMSO-d_6 126
Figure S 64: ^{1}H-NMR spectra (500 MHz) of ICTR-040 in DMSO-d_6 127
Figure S 65: ^{13}C-NMR (APT) spectra (100 MHz) of ICTR-040 in DMSO-d_6 128
Figure S 66: ^{1}H-NMR spectra (500 MHz) of ICTR-042 in DMSO-d_6 129
Figure S 67: ^{13}C-NMR (APT) spectra (100 MHz) of ICTR-042 in DMSO-d_6 130
Figure S 68: ^{1}H-NMR spectra (500 MHz) of ICTR-050 in DMSO-d_6 131
Figure S 69: ^{13}C-NMR (APT) spectra (125 MHz) of ICTR-050 in DMSO-d_6 132
Figure S 70: ^{1}H-NMR spectra (500 MHz) of ICTR-052 in DMSO-d_6 133
Figure S 71: ^{13}C-NMR (APT) spectra (100 MHz) of ICTR-052 in DMSO-d_6 134
Figure S 72: ^{1}H-NMR spectra (500 MHz) of ICTR-053 in DMSO-d_6 135
Figure S 73: ^{13}C-NMR (APT) spectra (100 MHz) of ICTR-053 in DMSO-d_6 136
Figure S 74: ^{1}H-NMR spectra (500 MHz) of ICTR-058*HCl in DMSO-d_6 137
Figure S 75: ^{13}C-NMR (APT) spectra (125 MHz) of ICTR-058*vHCl in DMSO-d_6 139
Figure S 76: ^{1}H-NMR spectra (500 MHz) of ICTR-059*HCl in DMSO-d_6 140
Figure S 77: ^{13}C-NMR (APT) spectra (125 MHz) of ICTR-059*HCl in DMSO-d_6 141
Figure S 78: ^{1}H-NMR spectra (500 MHz) of ICTR-060 in DMSO-d_6 142
Figure S 79: ^{13}C-NMR (APT) spectra (100 MHz) of ICTR-060 in DMSO-d_6 143
Figure S 80: ^{1}H-NMR spectra (500 MHz) of ICTR-063 in DMSO-d_6 144
Figure S 81: ^{13}C-NMR (APT) spectra (100 MHz) of ICTR-063 in DMSO-d_6 145
Figure S 82: ^{1}H-NMR spectra (500 MHz) of ICTR-076 in DMSO-d_6 146
Figure S 83: ^{13}C-NMR (APT) spectra (100 MHz) of ICTR-076 in DMSO-d_6 147

Figure S 84: ^{1}H-NMR spectra (500 MHz) of ICTR-078 in DMSO-d_6 148
Figure S 85: ^{13}C-NMR (APT) spectra (100 MHz) of ICTR-078 in DMSO-d_6 149
Figure S 86: ^{1}H-NMR spectra (500 MHz) of ICTR-086*HCl in DMSO-d_6 150
Figure S 87: ^{13}C-NMR (APT) spectra (125 MHz) of ICTR-086*HCl in DMSO-d_6 151
Figure S 88: ^{1}H-NMR spectra (500 MHz) of ICTR-094 in DMSO-d_6 152
Figure S 89: ^{13}C-NMR (APT) spectra (125 MHz) of ICTR-094 in DMSO-d_6 154
Figure S 90: ^{1}H-NMR spectra (500 MHz) of ICTR-096 in DMSO-d_6 155
Figure S 91: ^{13}C-NMR (APT) spectra (125 MHz) of ICTR-096 in DMSO-d_6 156
Figure S 92: ^{1}H-NMR spectra (500 MHz) of ICTR-108 in DMSO-d_6 157
Figure S 93: ^{13}C-NMR (APT) spectra (125 MHz) of ICTR-108 in DMSO-d_6 158
Figure S 94: ^{1}H-NMR spectra (500 MHz) of ICTR-119 in DMSO-d_6 159
Figure S 95: ^{13}C-NMR (APT) spectra (125 MHz) of ICTR-119 in DMSO-d_6 160
Figure S 96: ^{1}H-NMR spectra (500 MHz) of ICTR-120 in DMSO-d_6 161
Figure S 97: ^{13}C-NMR (APT) spectra (125 MHz) of ICTR-120 in DMSO-d_6 162

Figure S 1: Validation of the catalytic activity of recombinant PRMT enzymes.

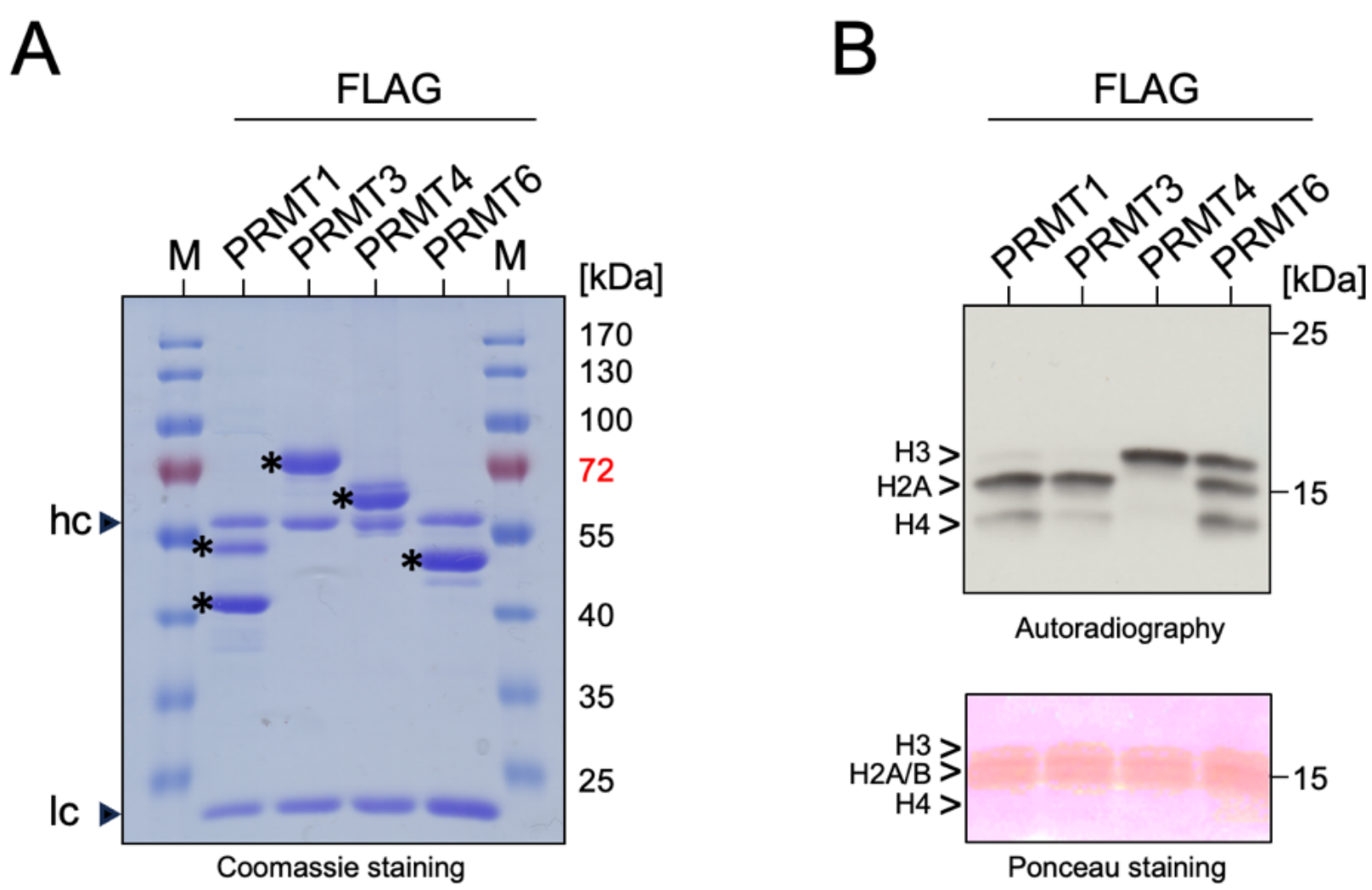

A: Coomassie blue-stained SDS-PAGE gel visualizes the recombinant FLAG-tagged PRMT1, PRMT3, PRMT4, and PRMT6 enzymes purified from baculovirus-infected Sf9 cells. Molecular weights (kDa) of the protein marker bands are indicated. Arrowheads mark the heavy chain (hc) and light chain (lc) of the resin-derived anti-FLAG antibodies. Full-length FLAG-tagged PRMT protein bands are marked with asterisks. FLAG-PRMT1 appears as two bands: a lower migrating band with the expected molecular weight of 42 kDa and the higher migrating band, post-translationally modified, with a molecular weight of approximately 50 kDa.

B: Recombinant FLAG-tagged PRMTs (from A) were subjected to *in vitro* methyltransferase assays in the presence of core histones and [^{14}C-methyl]-SAM. Reactions were separated by SDS-PAGE, blotted and analyzed by autoradiography. Methylation of the histone proteins served as positive control for both the catalytic activity and specificity of the different PRMTs as well as the quality of the enzyme preparations. Arrowheads indicate the protein bands of histones H3, H2A, and H4, which are substrates of PRMT1/PRMT3 (H2A and H4), PRMT4 (H3) and PRMT6 (H3, H2A, and H4). Ponceau S staining of the blot serves as loading control.

Figure S 2: Establishment of the F-PRMT assay.

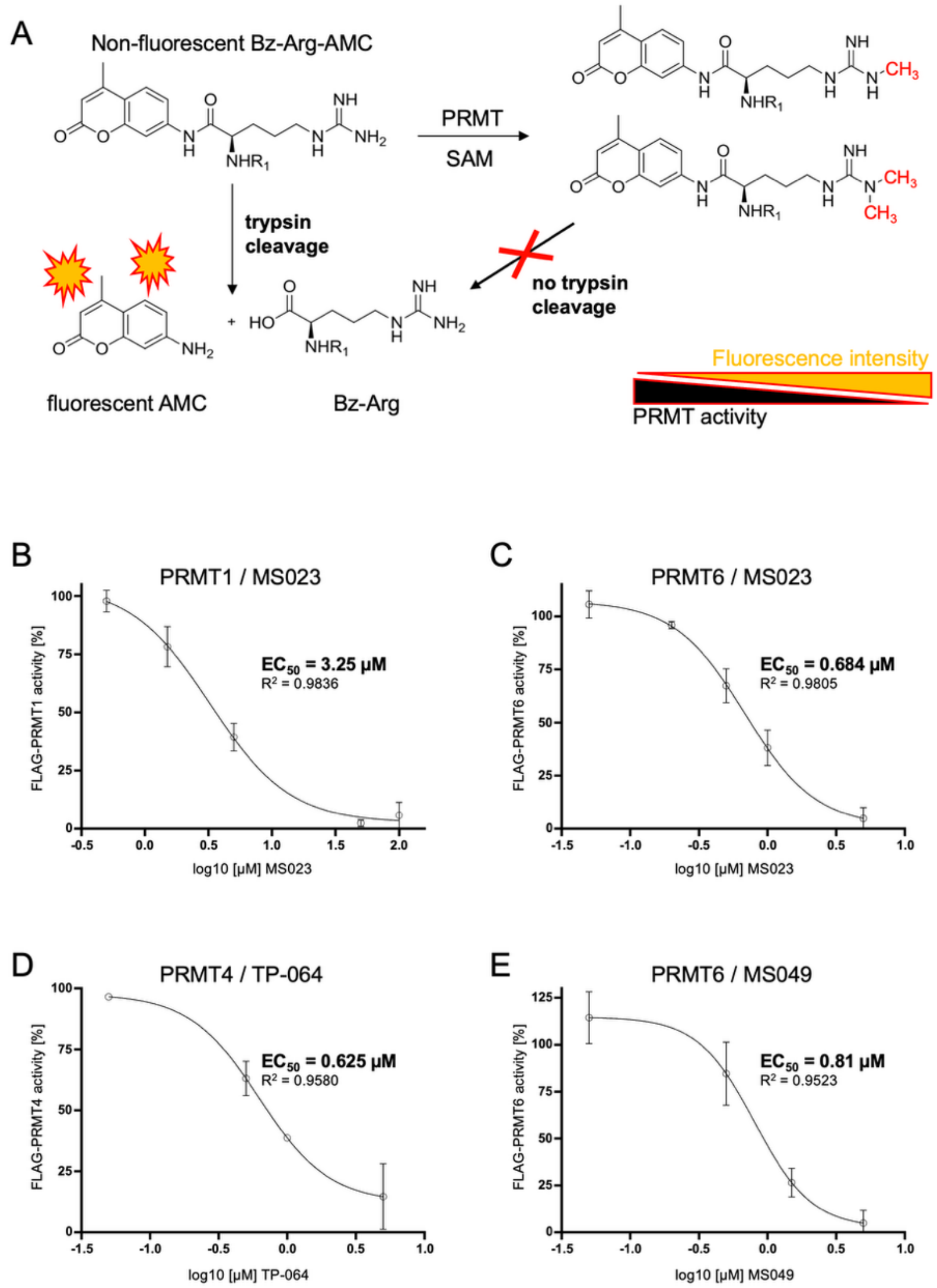

A: Reaction scheme of the *in vitro* fluorescence-based PRMT assay (F-PRMT assay). The fluorogenic substrate Bz-Arg-AMC undergoes methylation by PRMTs upon the addition of the cofactor SAM. Subsequently, trypsin cleaves the non-methylated substrate, leading to the generation of the fluorescent product AMC. In contrast, methylated substrate molecules are resistant to trypsin cleavage. Therefore, the fluorescence intensity is inversely correlated to the PRMT enzymatic activity (Kramer et al., 2018). B-E: The half-maximal effective concentration (EC_{50}) of commercially available PRMT inhibitors using the F-PRMT assay was determined by applying 4-5 concentrations of either MS023, TP-064, or MS049 to FLAG-tagged PRMT1, PRMT4, or PRMT6 (2 µM). The enzyme activities were calculated based on the mean fluorescence intensities. Activities of compound-treated enzymes are shown in % relative to the solvent-treated enzyme activity, which was set to 100% (mean ± SD, n=3), and then plotted against the logarithm of the compound concentrations. Concentration-activity curves were fitted by nonlinear regression, with the EC_{50} values and the coefficients of determination (R^2) indicated.

Figure S 3: Structure revision of K002FT017 and structure elucidation of TR-07.

A

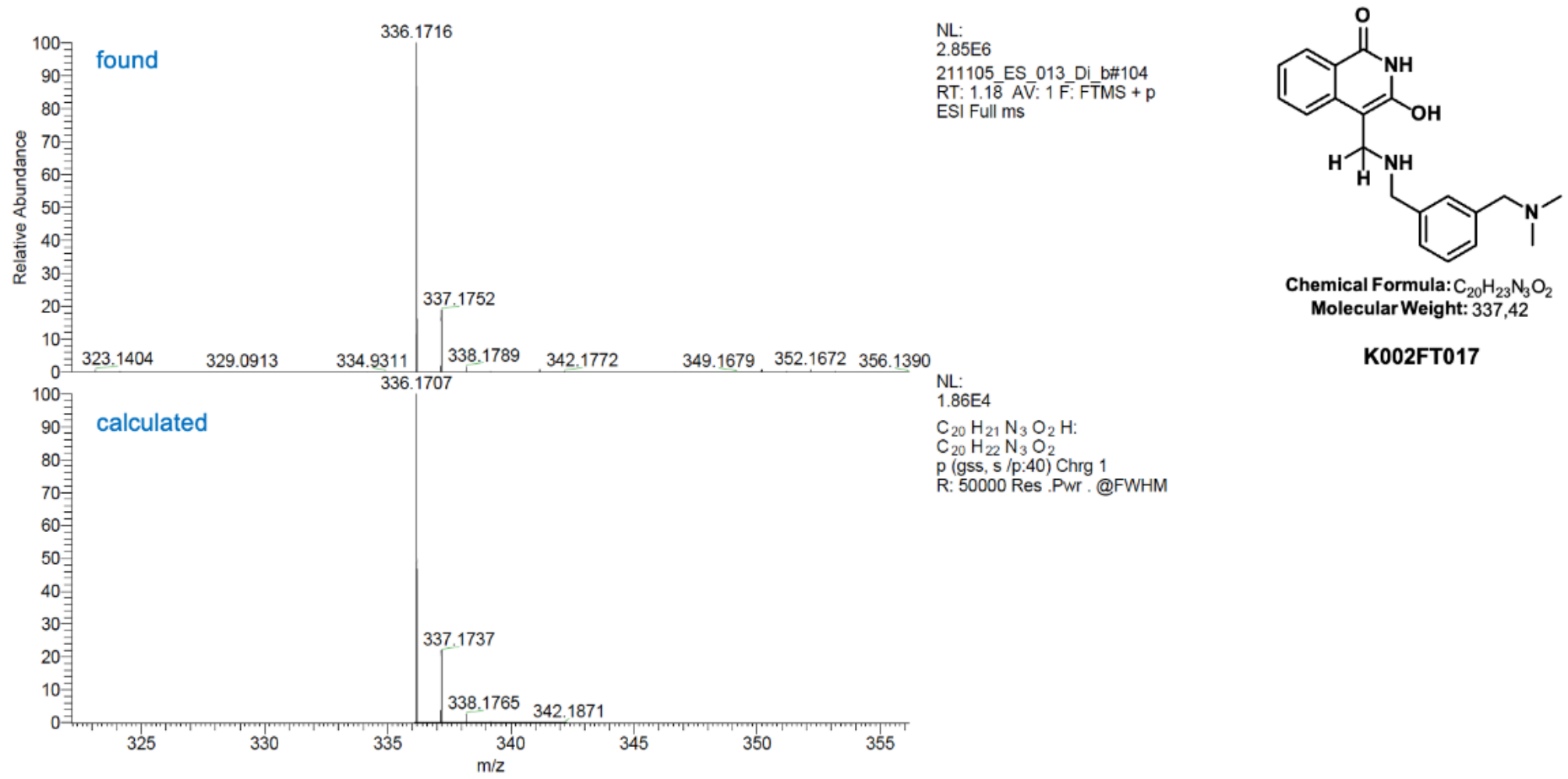

B

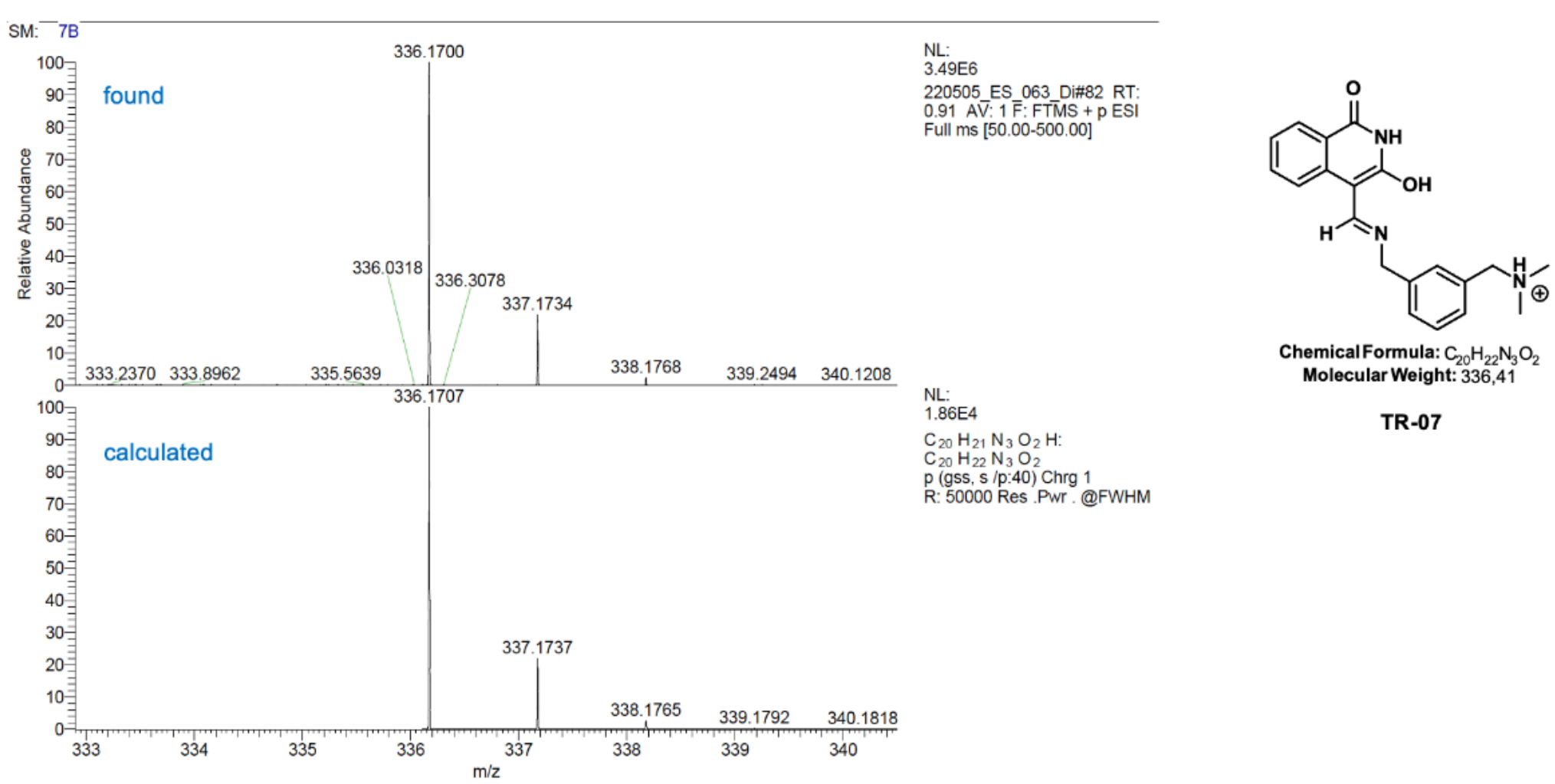

HR-MS: calcd for $C_{20}H_{22}N_3O_2$ $[M]^+$: 336.1707; found: 336.1700

A: High-resolution mass spectrum of K002FT017 (ESI, *m/z*): The found mass of 336.1716 does not match the expected mass for either $[(M+H)^+]$ or $[M]^+$ of compound K002FT017, indicating that the proposed molecular formula of K002FT017 is not correct.
B: High resolution mass spectrum of TR-07 (ESI, *m/z*). The calculated and found masses fit the chemical formula of TR-07*HCl.

Figure S 3 (continued)

C

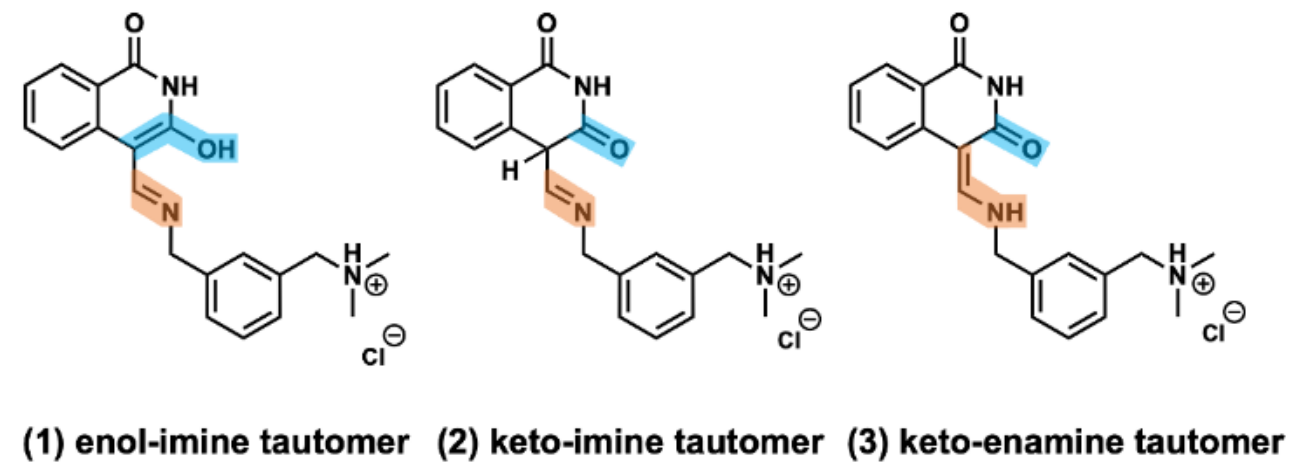

D

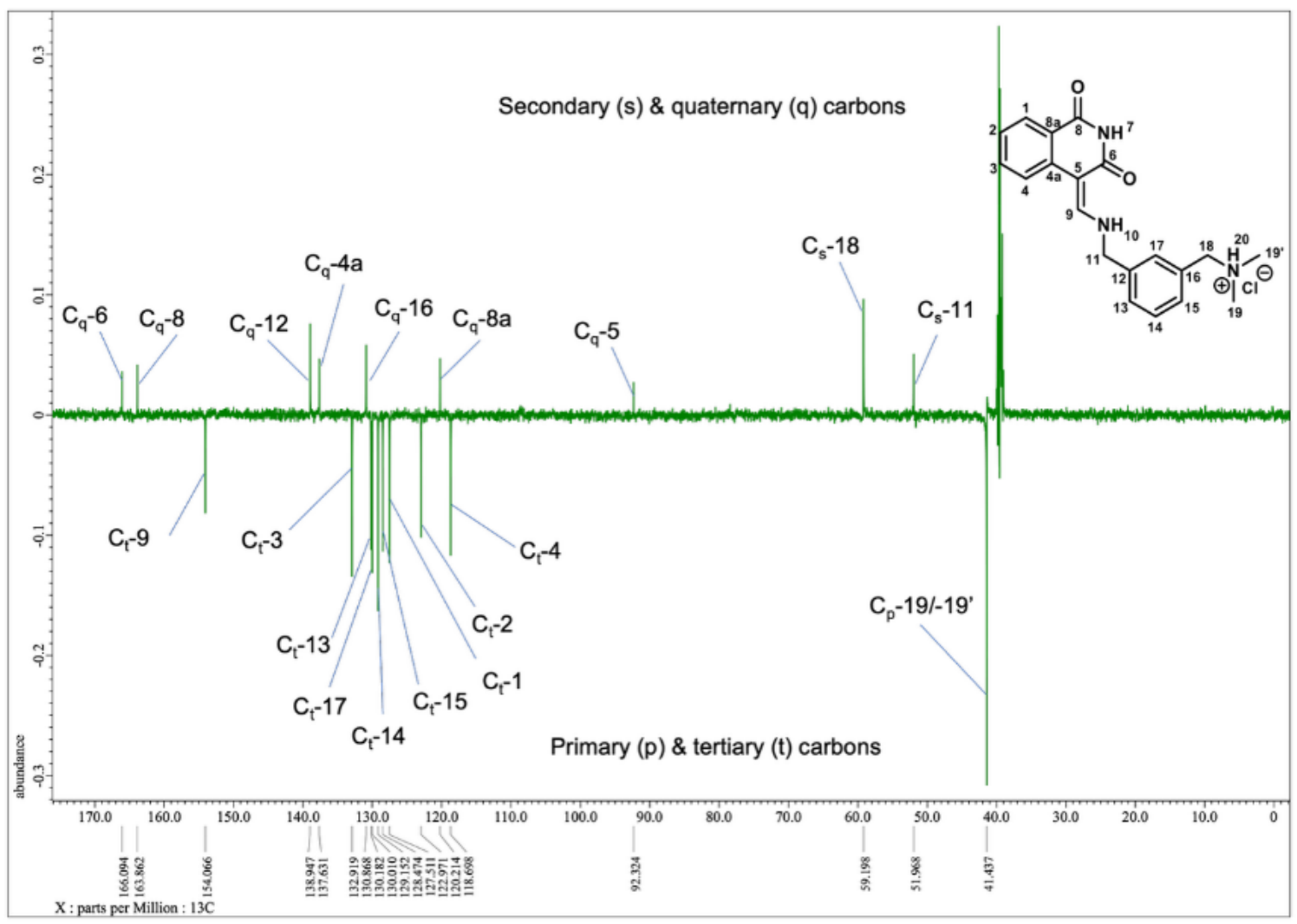

^{13}C-NMR (125 MHz, DMSO-d_6, 300 K) APT (attached proton test) spectrum of TR-07

E

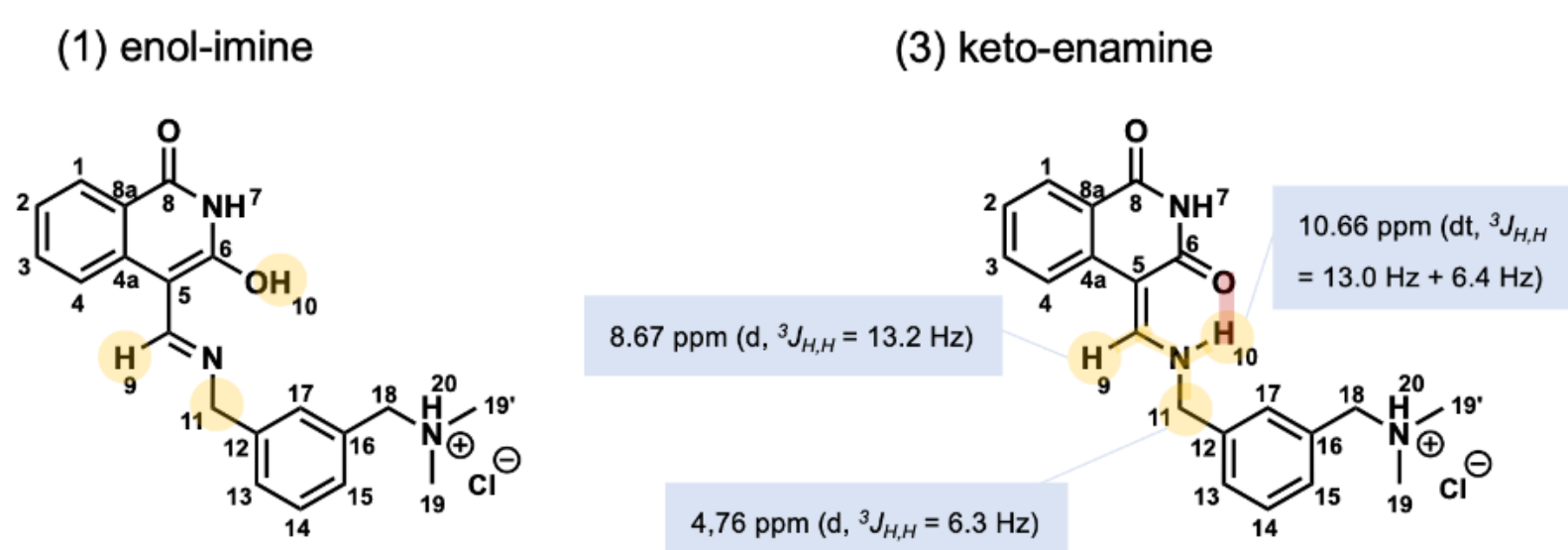

C: Structural representation of the three plausible tautomeric forms of TR-07, (1) enol-imine tautomer, (2) keto-imine tautomer, and (3) keto-enamine tautomer; structural differences are highlighted.
D: ^{13}C-NMR (125 MHz, DMSO-d_6, 300 K) APT (attached proton test) spectrum of TR-07. Upper part of the spectrum: signals of secondary and quarternary carbon atoms; lower part: signals of primary and tertiary carbon atoms. Based on this spectrum, the keto-imin tautomer could be excluded.
E: Based on the ^{1}H-NMR and HMBC spectra and the observed coupling constants, as well as the work of Albrecht & Fischer (1970)[6], the enol-imine tautomer was excluded. For a better illustration, only the two remaining tautomers and the assigned coupling constants are shown: the enamine proton (H-10) on the one hand couples with the allylic proton (H-9): d ($^3J_{H,H}$ = 13.2 Hz); on the other hand, it interacts with the methylene group resulting in a dt ($^3J_{H,H}$ = 13.0, 6.4 Hz).

Figure S 3 (continued)

F

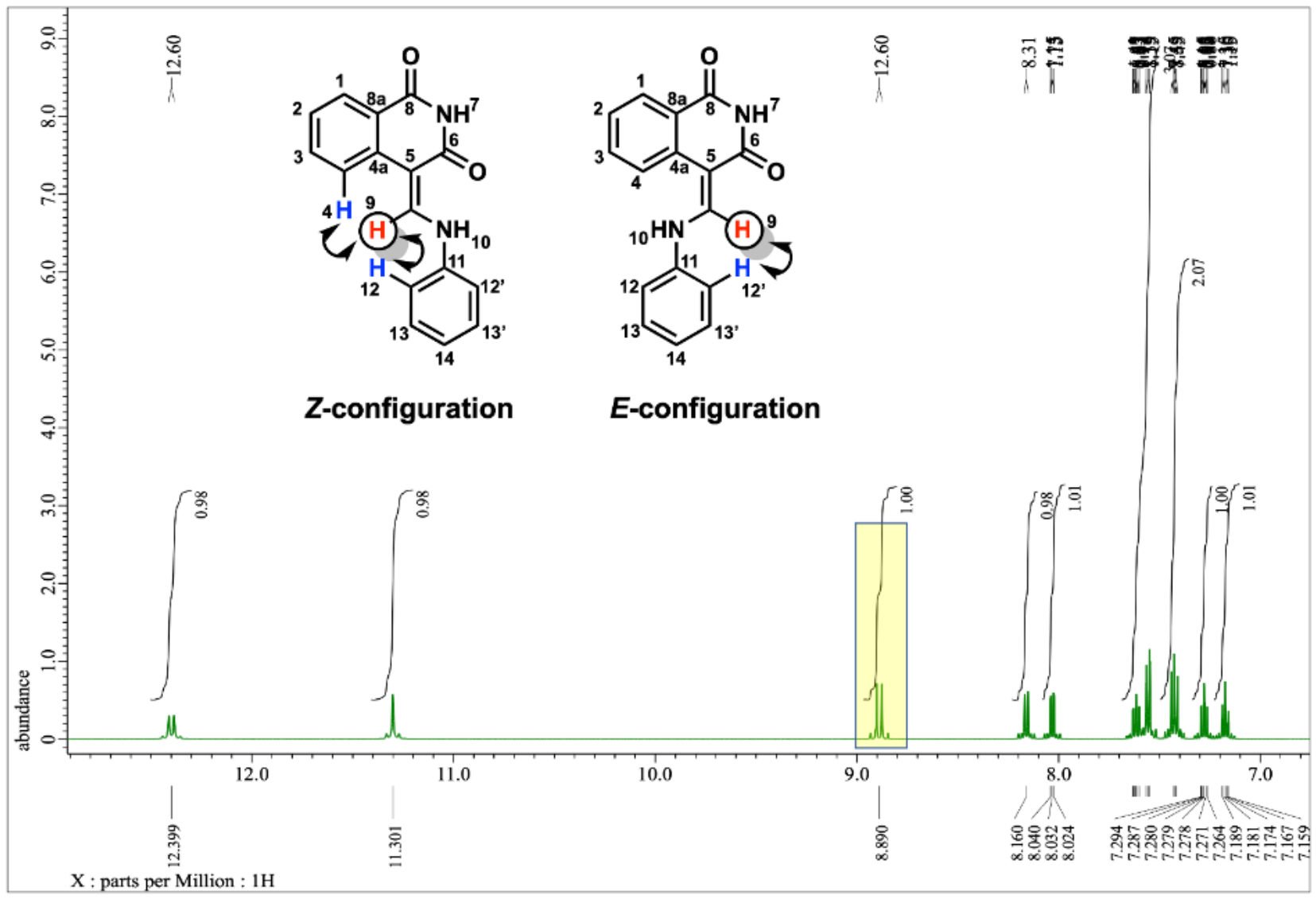

^{1}H-NMR (500 MHz, DMSO-d_6, 300 K) spectrum of TR-014

G

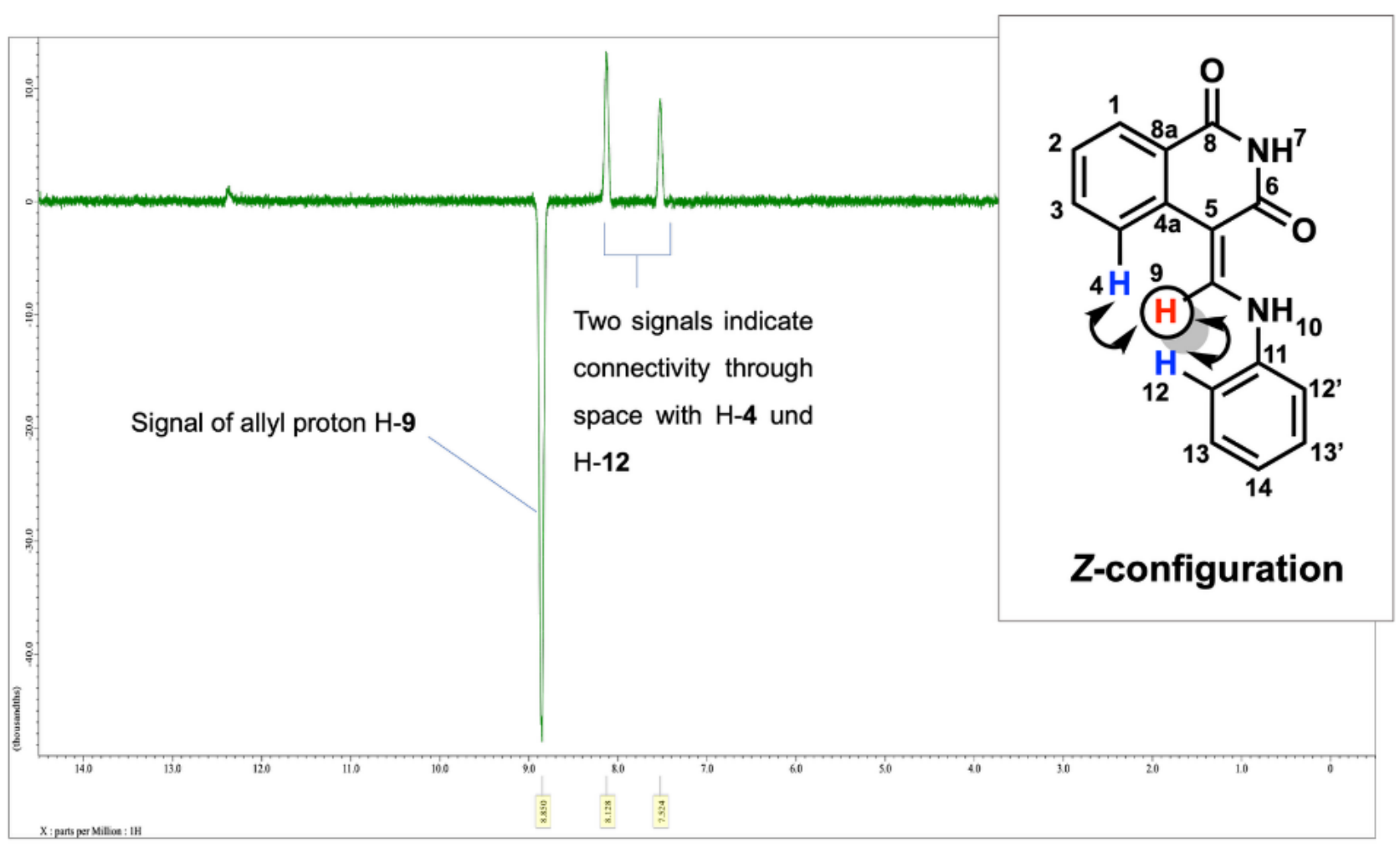

1D NOESY NMR (500 MHz, DMSO-d_6, 300 K) spectrum of TR-014

F: ^{1}H-NMR (500 MHz, DMSO-d_6, 300 K) spectrum of TR-014, only downfield region (13-7 ppm) is shown. Structural representation of the *Z*- and *E*-configured isomers, in which important coupling partners of the allyl proton (H-9) required for structure elucidation are highlighted.

G: Spectrum of the 1D NOESY experiment (500 MHz, DMSO-d_6, 300 K) of TR-014, only downfield region (13 - 7 ppm) is shown. In addition to the signal of the allylic proton at 8.85 ppm, two further signals occur at 8.13 ppm and 7.52 ppm. This proves the spatial coupling of H-9 to the two protons H-4 and H-12 and, accordingly, the *Z*-configuration of this compound.

Figure S 4: Unbiased docking calculations of a TR-07 subfragment to human PRMT1.

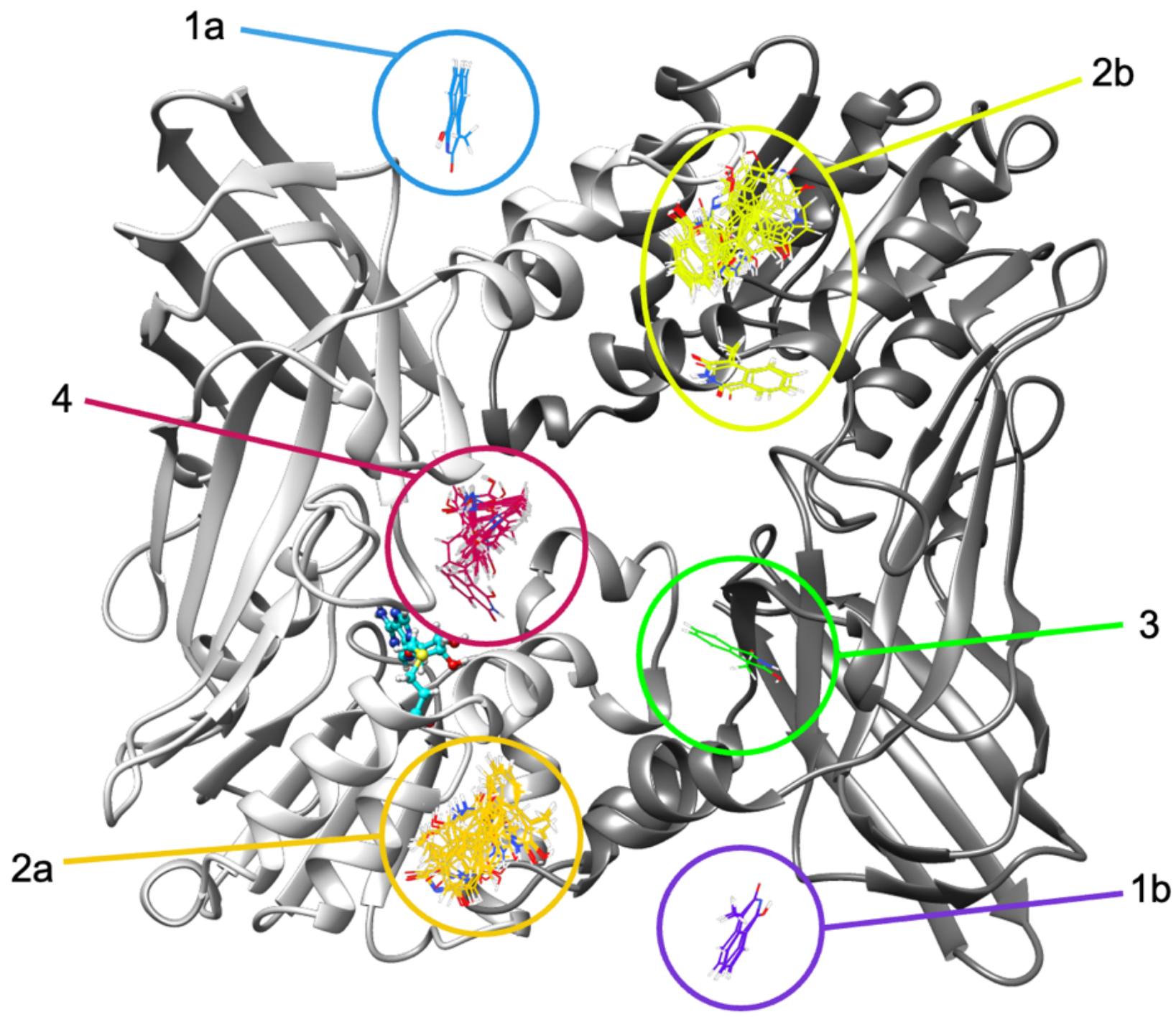

Depiction of possible binding poses of 3-hydroxy-4-methyl-1(2H)-isoquinolinone in human PRMT1 (PDB: 6NT2) generated with the SwissDock web-service. The input used for this unbiased docking was a homodimer of human PRMT1, and due to symmetry, two of the potential pose clusters (no. 1 and 2) are depicted twice (denoted as a and b for the same number). For the sake of orientation, the co-crystallized SAH (in cyan) is added in one monomer. The SAH molecule in the second monomer partially overlaps with the yellow pose (no. 2b) and is therefore not depicted. Four potential binding sites have been identified. No. 1a/b: blue (light blue/indigo) – between the outer side of the dimerization arm and the β-barrel. No. 2a/b: yellow (lemon/mango) – around the αY-helix, extending from the SAM binding pocket to the interface between the dimerization arm and the Rossman fold. No. 3: green – between the C-terminus and the dimerization arm. No. 4: red – close to the arginine binding site.

Figure S 5: Comparison of the docking pose of TR-036 in human PRMT1, focusing on the region encompassing E216, with the corresponding regions in PRMT3, PRMT4, and PRMT6.

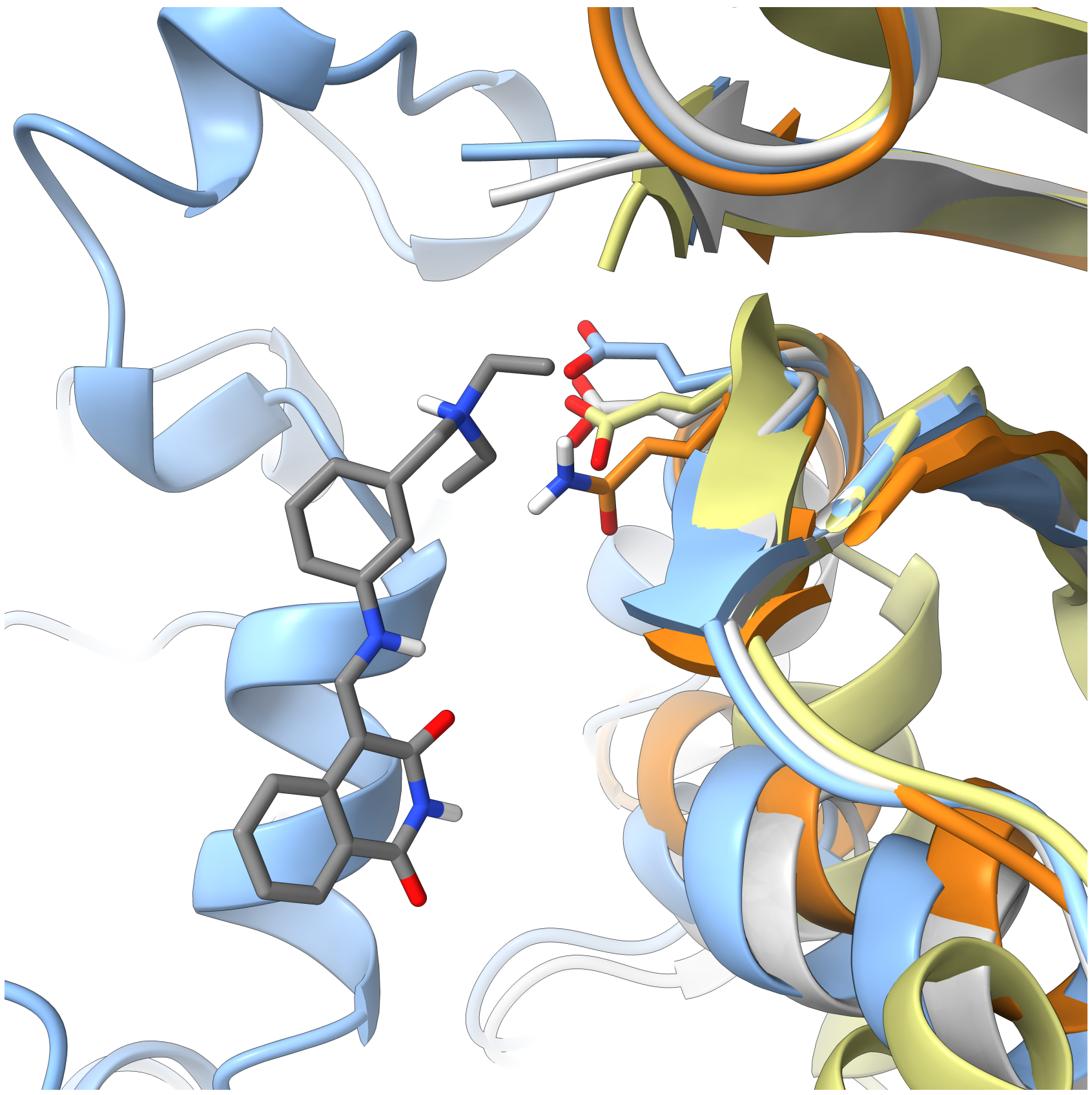

The docking pose of TR-036 is depicted in dark grey. PRMT1 is depicted in blue, PRMT3 in grey, PRMT4 in orange, and PRMT6 in yellow. The only side chains shown are E216 (human PRMT1), E418 (human PRMT3), Q337 (human PRMT4), and E235 (human PRMT6).

Figure S 6: HDX-MS analysis of TR-07 binding to PRMT1.

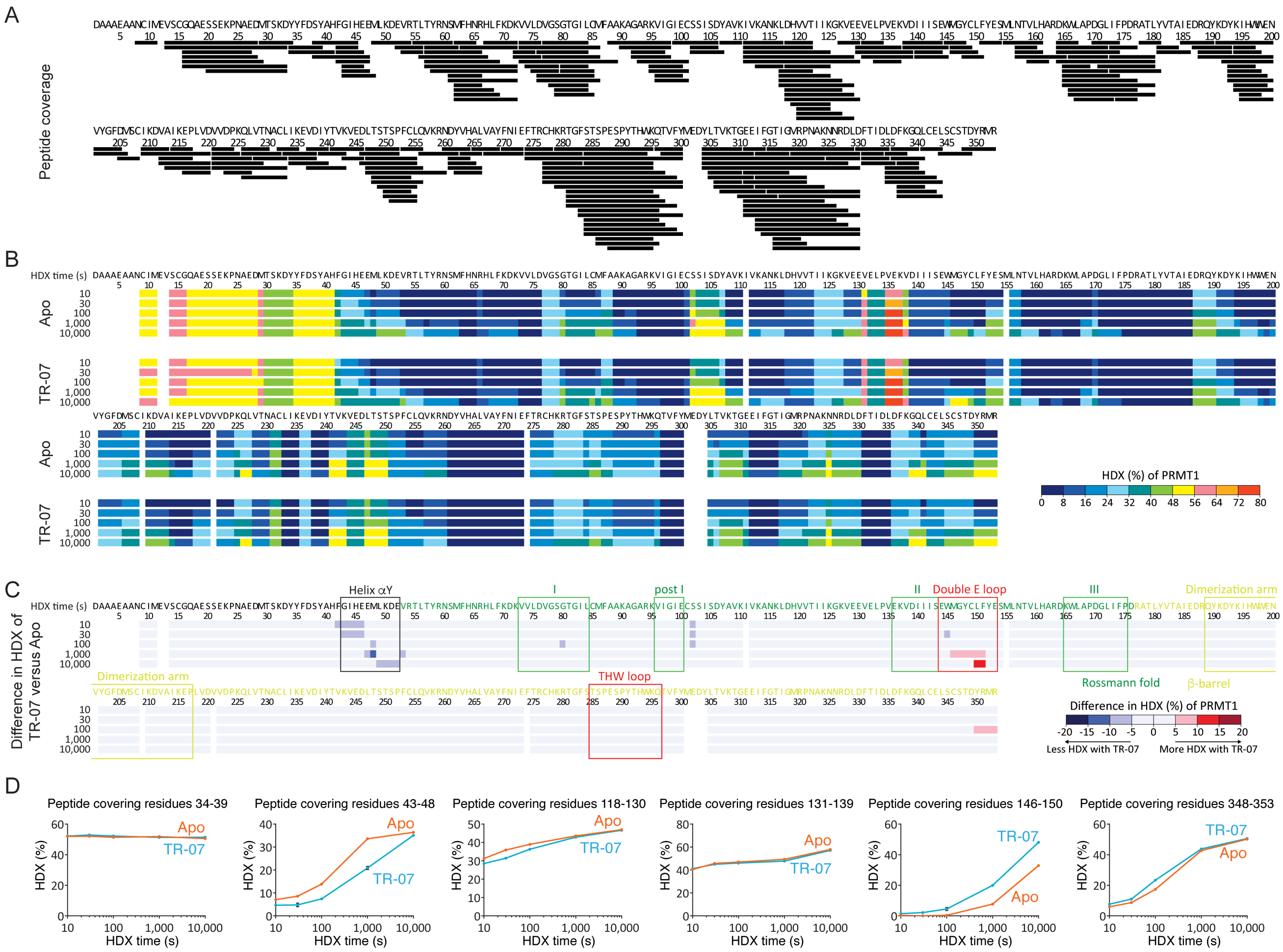

A: Each black bar projected on the amino acid sequence of FLAG-tagged rat PRMT1 (Uniprot-ID Q63009) represents a peptide identified in HDX-MS for the holo state (TR-07-bound) and the corresponding apo (ligand-free) control.
B: The deuterium incorporation of FLAG-tagged PRMT1 in the apo or TR-07-bound states over time is displayed in rainbow colors from blue (0% deuterium uptake) to red (80% deuterium uptake).
C: The differences in deuterium incorporation between the TR-07-bound FLAG-tagged PRMT1 and the apo control are displayed on the amino acid sequence. Blue and red color denote regions of PRMT1 with reduced ("protection") and increased ("deprotection") HDX in presence of TR-07, respectively. The *N*-terminal helix aY (black) and the conserved regions of the catalytic core are indicated: Rossman fold (green) with the SAM-binding motifs (I, post I, II, and III), the b-barrel and dimerization arm (yellow), and the two loops (Double E loop and the THW loop, red) that form the active site.
D: Deuterium incorporation of selected representative PRMT1 peptides. Data represent mean ± SD of n=3 technical replicates.

Figure S 7: HDX-MS analysis of TR-036 binding to PRMT1.

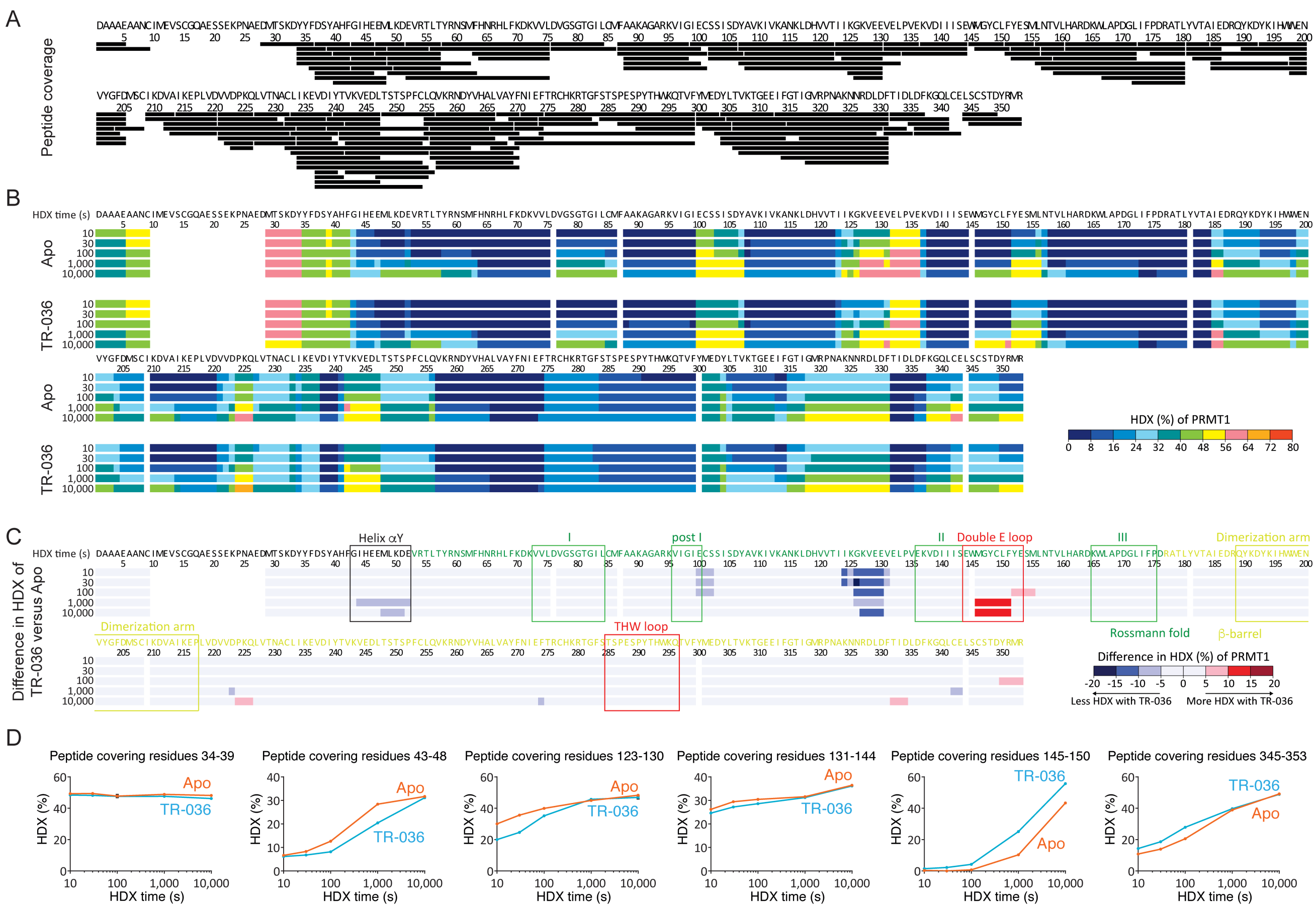

A: Each black bar projected on the amino acid sequence of FLAG-tagged rat PRMT1 (Uniprot-ID Q63009) represents a peptide identified in HDX-MS for the holo state (TR-036-bound) and the corresponding apo (ligand-free) control.
B: The deuterium incorporation of FLAG-tagged PRMT1 in the apo or TR-036-bound state over time is displayed in rainbow colors from blue (0% deuterium uptake) to red (80% deuterium uptake).
C: The differences in deuterium incorporation between the TR-036-bound FLAG-tagged PRMT1 and the apo control are displayed on the amino acid sequence. Blue and red color denote regions in PRMT1 with reduced ("protection") and increased ("deprotection") HDX in presence of TR-036, respectively. The N-terminal helix aY (black) and the conserved regions of the catalytic core are indicated: Rossman fold (green) with the SAM-binding motifs (I, post I, II, and III), the b-barrel and dimerization arm (yellow), and the two loops (Double E loop and the THW loop, red) that form the active site.
D: Deuterium incorporation of selected representative PRMT1 peptides. Data represent mean ± SD of n=3 technical replicates.

Figure S 8: Impact of TR-033 and TR-036 on homodimerization of PRMT1.

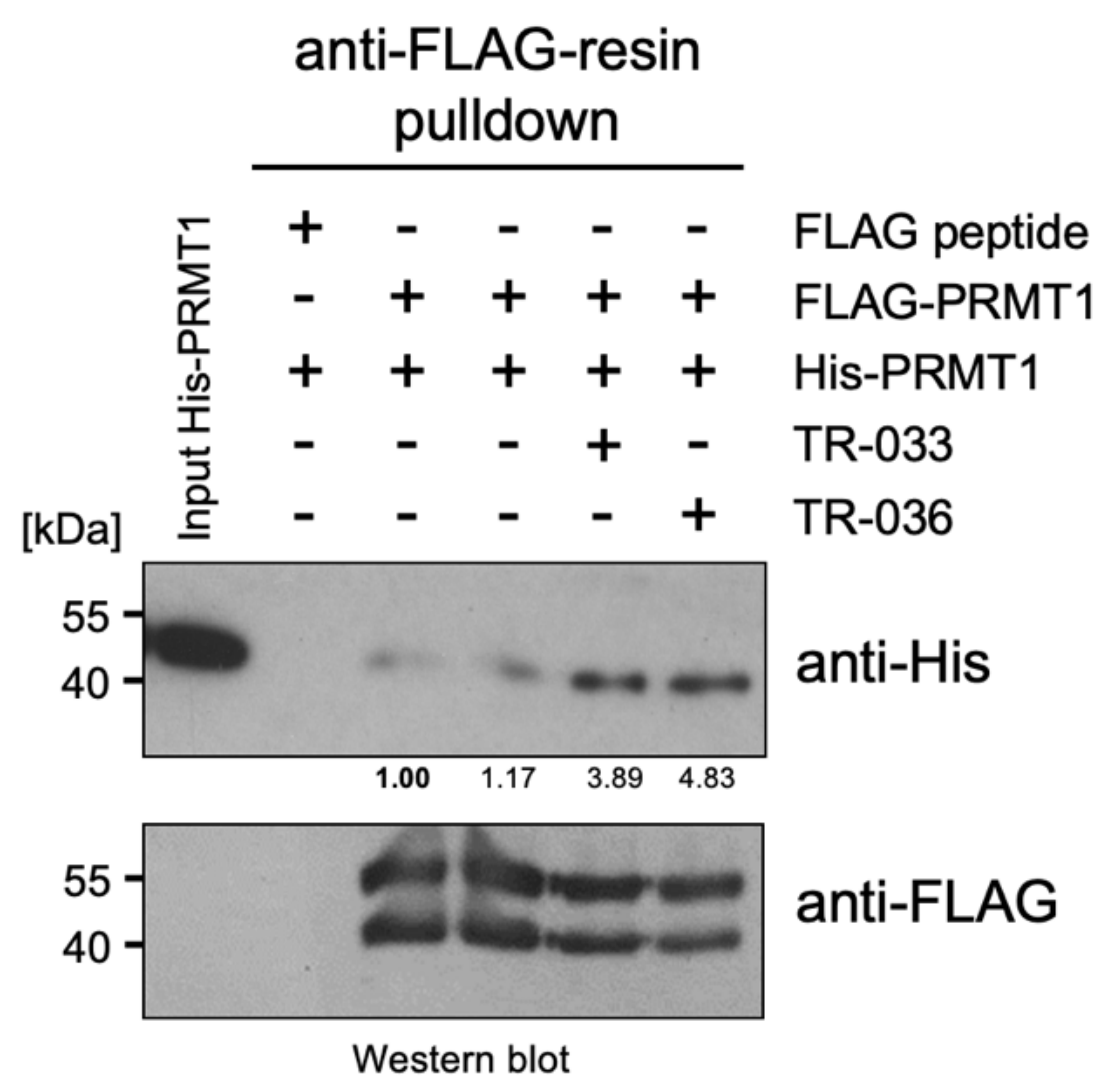

FLAG-tagged PRMT1 immobilized to anti-FLAG resin and eluted His-tagged PRMT1 (mobile phase) were incubated in the presence of TR-033 or TR-036 (200 µM, +), or in the presence of DMSO (solvent of the compounds, -). Subsequently, FLAG-beads were washed and bead-bound proteins were analyzed together with an His-tagged PRMT1 input (20 ng) by Western blot using antibodies against the His- and FLAG-tags. As negative control, anti-FLAG resin saturated with FLAG peptide was incubated with His-tagged PRMT1. The His-PRMT1 signals were densitometrically quantified, corrected to the FLAG-PRMT1 signals, and expressed relative to the solvent control, as specified by the numbers below the immunostainings.

Figure S 9: Quantification of the effect of TR-07 on the homooligomeric interaction of PRMT1 from biological replicates.

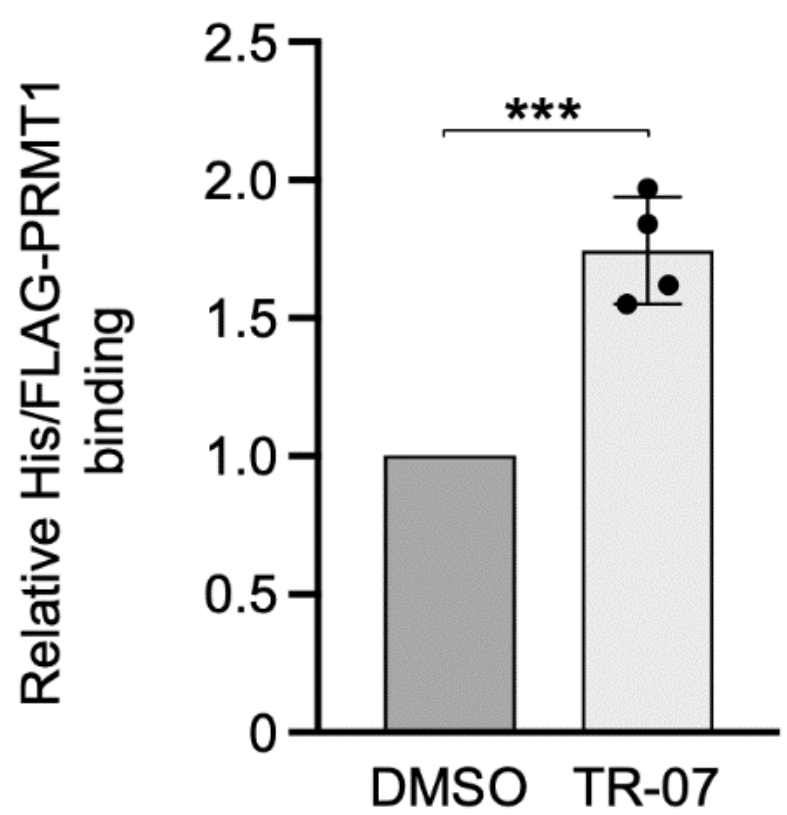

Pulldown assays using immobilized FLAG-tagged PRMT1 and eluted His-tagged PRMT1 in the presence of 200 µM TR-07 or DMSO (solvent control) were performed and densitometrically quantified, as described in Fig. 5C. The quantification graph summarizes the results of four biological replicates (including the representative result shown in Fig. 5C). The signal of His-PRMT1 binding to FLAG-PRMT1 in the presence of TR-07 was expressed relative to the solvent control, which was set to 1 (mean ± SD, n=4; ***: $p \leq 0.001$, using the student *t*-test).

Figure S 10: Effect of PRMT1 activators TR-033, TR-036, and ICTR-059 on gemcitabine-induced apoptosis in PDAC cell lines.

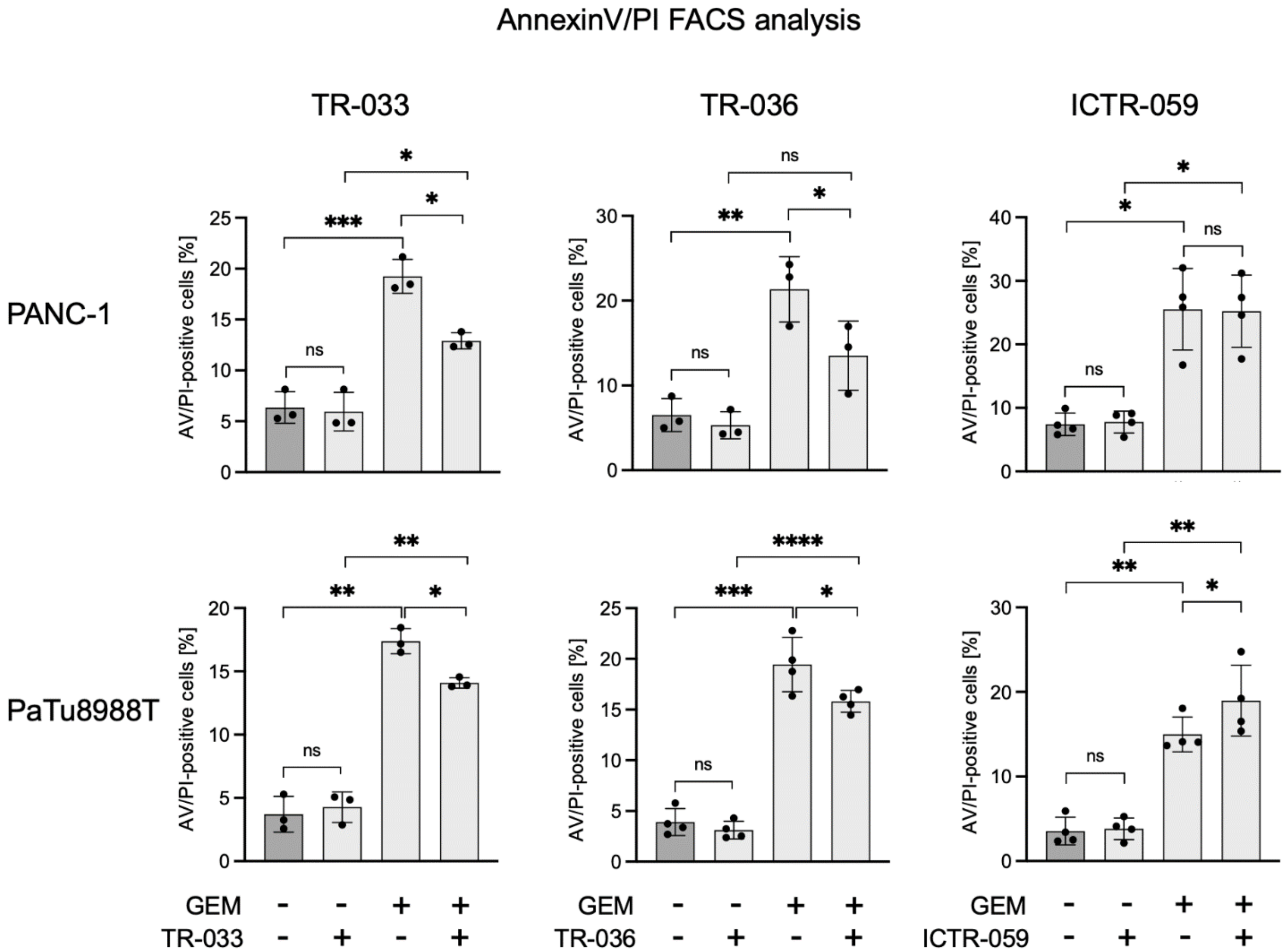

PANC-1 and PaTu8988T cells were cultured in the absence (-) or presence (+) of TR-033 (5 µM), TR-036 (10 µM), or ICTR-059 (5 µM). After 24 hours, gemcitabine (GEM, 1 µM) was added (+) or not (-) for another incubation of 48 hours. Subsequently, the apoptotic cell fraction was assessed by flow cytometry using FITC-labeled Annexin V (AV) and propidium iodide (PI) staining and quantified in % (mean ± SD, n=3-4; ns: not significant, *: p ≤0.05, **: p ≤0.01, ***: p ≤0.001, ****: p ≤0.0001, using the student *t*-test).

Figure S 11: Effect of PRMT1 activators TR-07, TR-033, TR-036, and ICTR-059 on the viability of PaTu8898T cells.

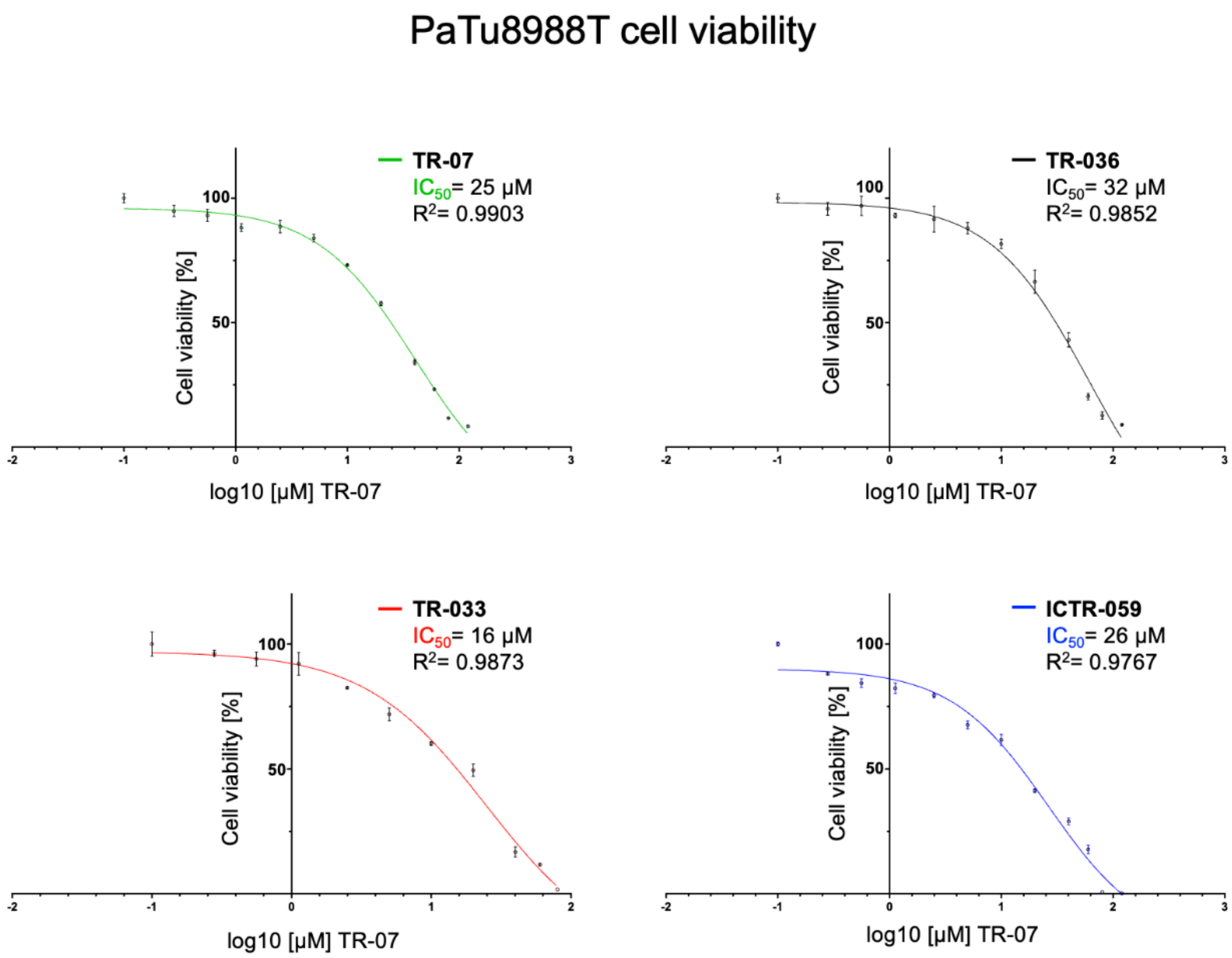

PaTu8988T cells were cultured for 96 hours in the presence of DMSO (solvent control) or activator compounds, each at 11-12 different concentrations. Cell viability was quantified using the CellTiter-Glo assay. Viability of compound-treated cells is shown in % relative to the viability of solvent-treated cells, which was set to 100% (mean ± SD, n=3), and then plotted against the logarithm of the compound concentrations. Concentration-viability curves were fitted by nonlinear regression, with the half-maximal inhibitory concentration (IC_{50}) of the compounds for PaTu8898T cell viability and the coefficients of determination (R^2) indicated.

Figure S 12: Monitoring of the body weight of mice bearing PANC-1 xenografts

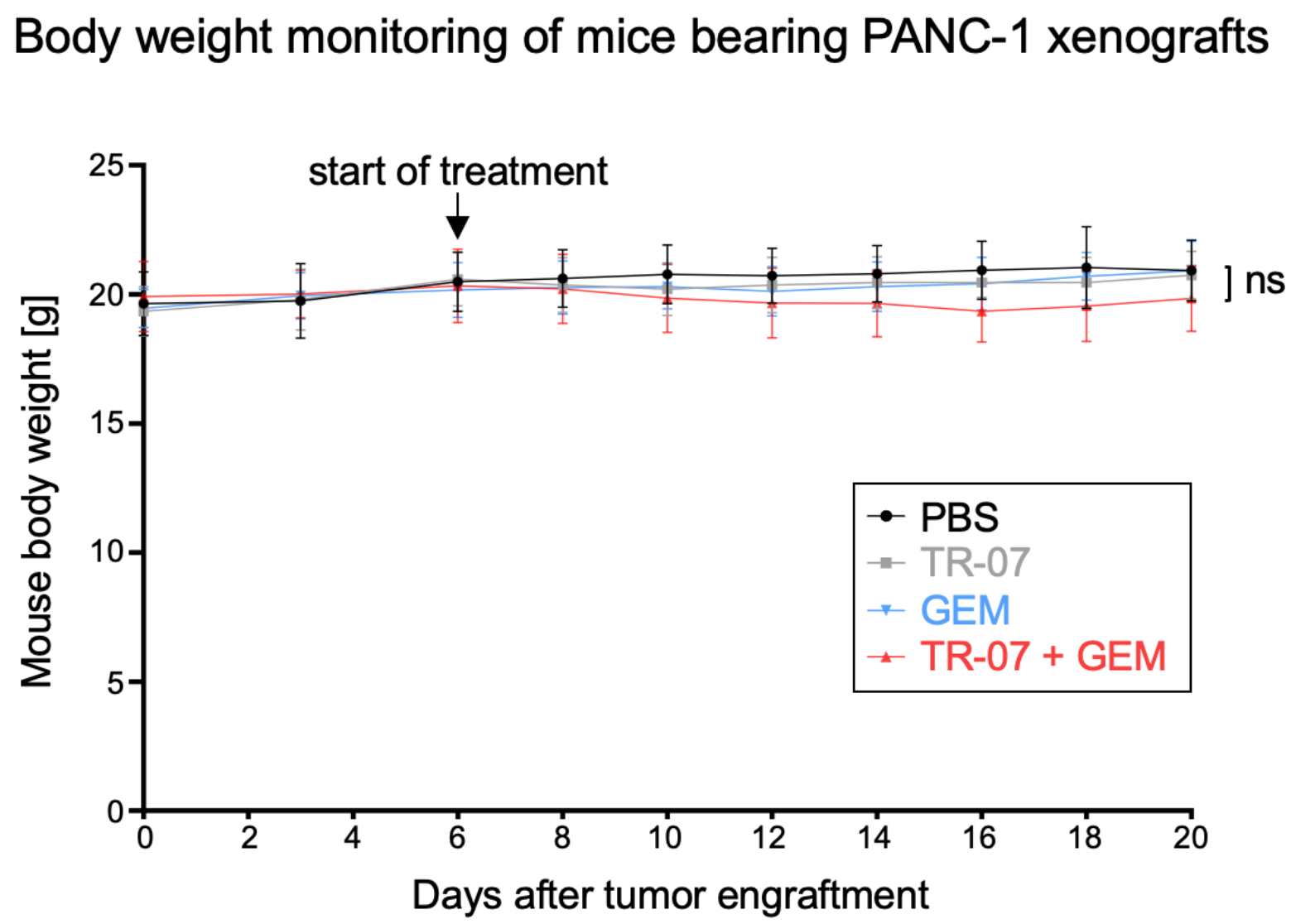

In the PDAC xenograft experiments of Fig. 6D, PANC-1 cells were injected into athymic nude mice. Six days post-engraftment, the mice were divided into four cohorts that received either PBS, TR-07, gemcitabine (GEM), or a combination of TR-07 and GEM (TR-07 + GEM) for 2 weeks. The body weight of the mice was measured every 2-3 days throughout the experiment. The mean body weights of each treatment cohort are displayed (mean ± SD, n=5 for each cohort; ns: not significant, using the student *t*-test).

Figure S 13: Effect of TR-07 on the cellular ADMA levels in PANC-1 tumor xenografts.

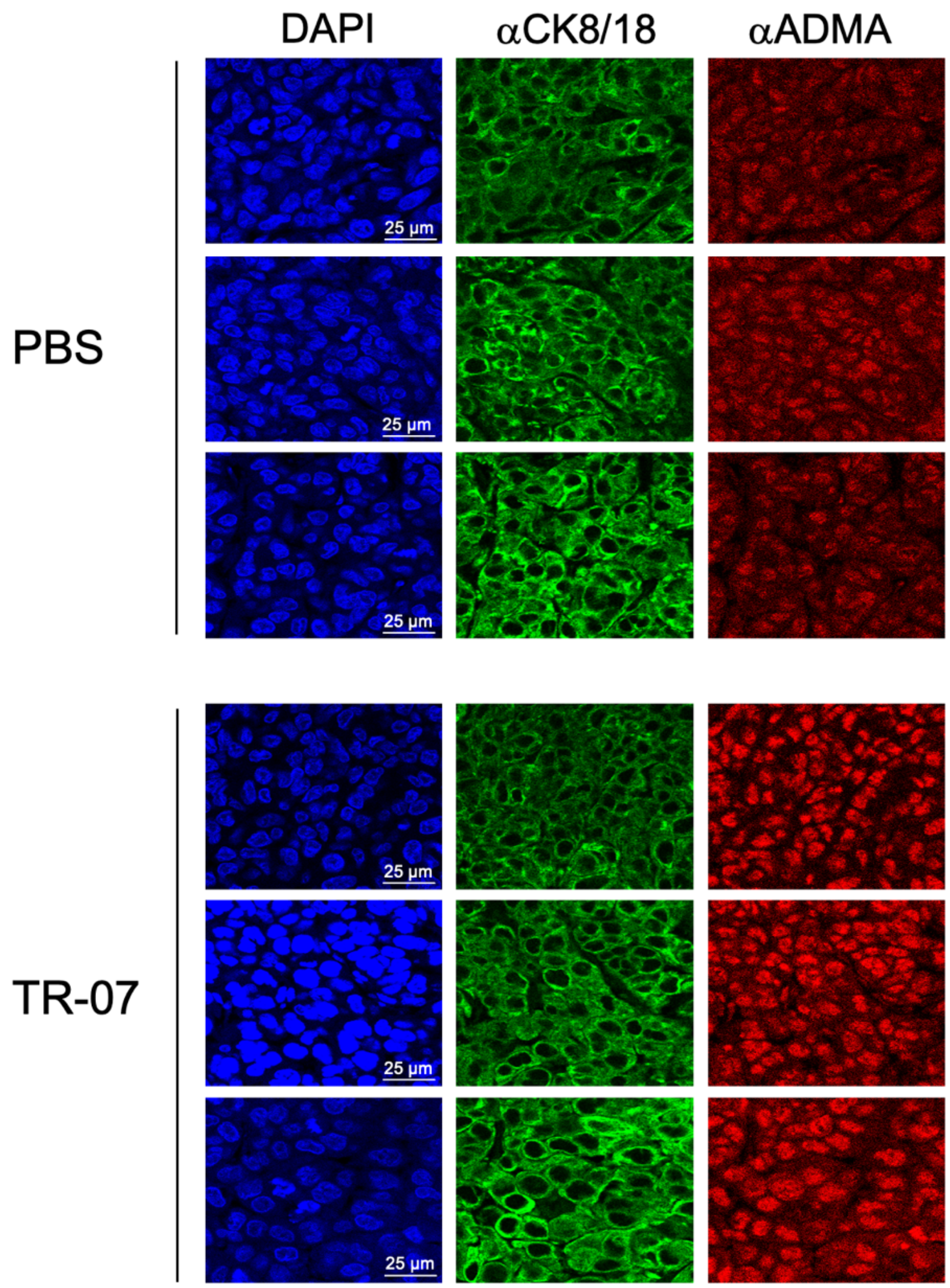

Post-engraftment of PANC-1 cells in PDAC xenograft experiments (Fig. 6D), the mice were treated with PBS or TR-07 for two weeks. Then mice were sacrificed and the tumors were dissected (Fig. 6E). Immunofluorescence (IF) staining of the PANC-1 xenograft tissues was performed using DAPI (blue, nuclei/DNA), antibodies against CK8/18 (green, staining PANC-1 cells of the tumor xenograft tissue) and ADMA (red). Representative IF images of 3 tumors each from PBS- and TR-07-treated mice are shown. Scale bars: 25 µm.

Table S1: Overview of docking setups.

Docking series, intended target PRMT, used PDB structures, docking programs, and used libraries are summarized. [a] LL = lead-like subset (clogP < 4 & 250 g/mol < MW < 350 g/mol). [b] The custom subsets were built by similarity and substructure searches further explained in the Methods.

Series	Intended target	Used PDB structure(s)	Docking program	Library used
K001FTxxx	PRMT4	5DXA	DOCK3.6	ZINC12 LL[a]
K001JSxxx	PRMT4	6ARV, 6DVR, 5DWQ & 5DX0	DOCK3.6 & DOCK3.7	ZINC12 & ZINC15 LL[a]
K400JKxxx	PRMT4	2Y1X	DOCK3.7	ZINC20 custom set[b]
K002FTxxx	PRMT6	4Y30	DOCK3.6	ZINC12 & ZINC15 LL[a]
K002JSxxx	PRMT6	4Y30	DOCK3.6 & DOCK3.7	ZINC15 LL[a]
K600JKxxx	PRMT6	4Y2H, 4Y30, 5E8R, 5EGS, 6P7I & 7NR4	OpenEye HYBRID & FRED	ZINC20 custom set[b]
K500JKxxx	PRMT1	6NT2	OpenEye HYBRID & FRED	ZINC20 custom set[b]

Table S2: Summary of the remaining 59 compounds of the original docking screens with respect to their effects on the catalytic activity of the intended target PRMT, either PRMT4 or PRMT6.

Compound names, intended PRMT to inhibit (i), 2D structure, experimental result of the F-PRMT assays, the compounds' efficacy in altering PRMT activity (±Δ%), and the corresponding p-values of efficacy are summarized. Two-dimensional structures depict the enantiomer that emerged from the docking calculations, whereas racemates were used in all experiments with docking-derived compounds. The PRMT activity upon compound treatment (100 µM) was calculated as % relative to the activity in the presence of solvent control (untreated), for which the activity was set to 100% (n=3). Changes upon compound treatment (=efficacy) were expressed as mean % differences of inhibition (-Δ%) or activation of catalysis (+Δ%). In case of an inhibitory (**i**) effect for the experimental result, the compound-treated activity was lower by -Δ% than untreated, whereas in case of an activatory (**a**) effect, the compound-treated activity was higher by +Δ% than untreated. Compounds, which did not significantly affect the catalytic activity of the intended PRMT (less than ±6% change or not significant p-value), are marked with **n.e.** (no effect) for the experimental result and with − for the efficacy. P-values of efficacy were calculated using the student *t*-test (n.s.: not significant; *: p ≤0.05,**: p ≤0.01, ***: p ≤0.001, ****: p ≤0.0001).

Compound	Intended target	2D structure	Experimental result	Efficacy ±Δ%	P-value
**K001FT001**	PRMT4 **i**	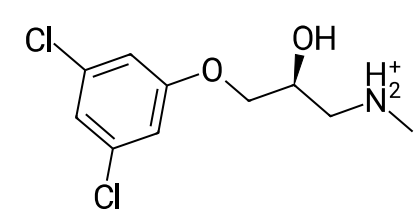	PRMT4 **i**	-25%	***
**K001FT002**	PRMT4 **i**	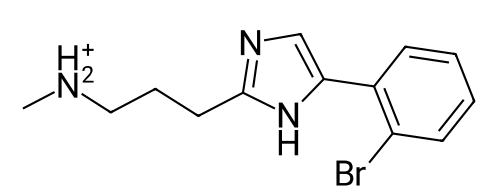	PRMT4 **n.e.**	-	n.s.
**K001FT003**	PRMT4 **i**	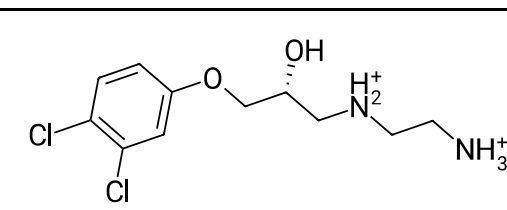	PRMT4 **i**	-50%	****
**K001FT004**	PRMT4 **i**	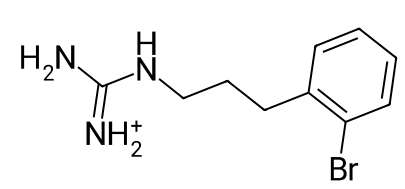	PRMT4 **n.e.**	-	n.s.
**K001FT005**	PRMT4 **i**	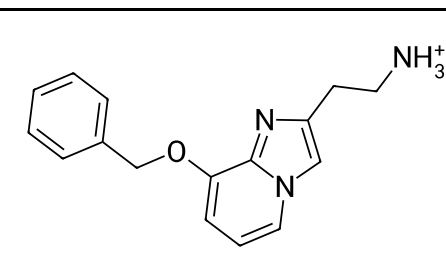	PRMT4 **n.e.**	-	n.s.
**K001FT006**	PRMT4 **i**	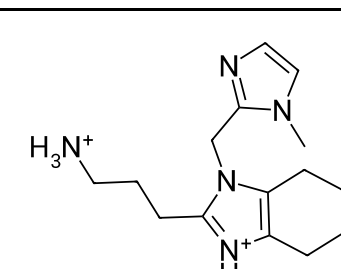	PRMT4 **n.e.**	-	n.s.
**K001FT007**	PRMT4 **i**	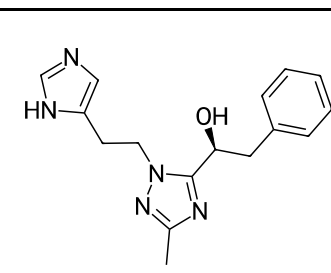	PRMT4 **n.e.**	-	n.s.

Table S2 continued

Compound	Intended target	2D structure	Experimental result	Efficacy ±∆%	P-value
**K001FT008**	PRMT4 i	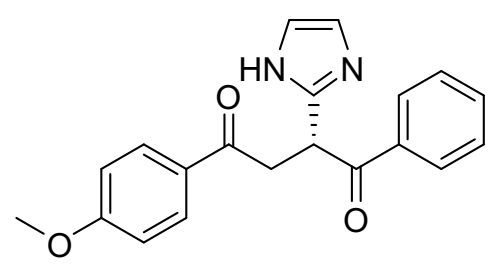	PRMT4 n.e.	-	n.s.
**K001FT009**	PRMT4 i	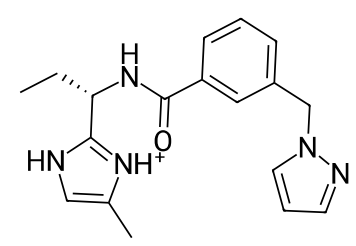	PRMT4 n.e.	-	n.s.
**K001FT010**	PRMT4 i	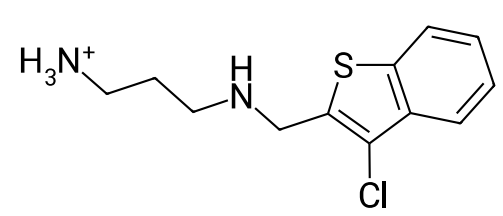	PRMT4 n.e.	-	n.s.
**K001FT011**	PRMT4 i	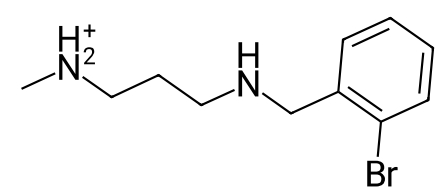	PRMT4 n.e.	-	n.s.
**K001JS001**	PRMT4 i	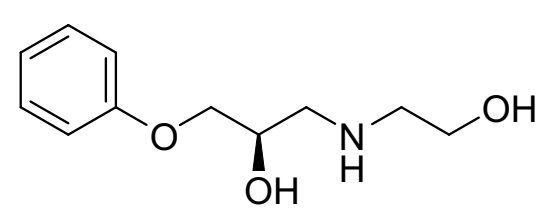	PRMT4 n.e.	-	n.s.
**K001JS002**	PRMT4 i	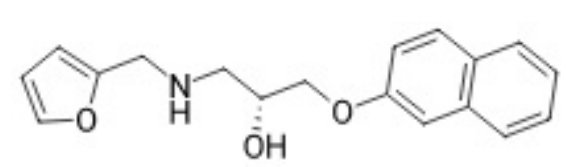	PRMT4 n.e.	-	n.s.
**K001JS005**	PRMT4 i	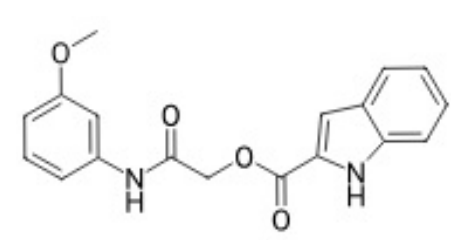	PRMT4 n.e.	-	n.s.
**K001JS008**	PRMT4 i	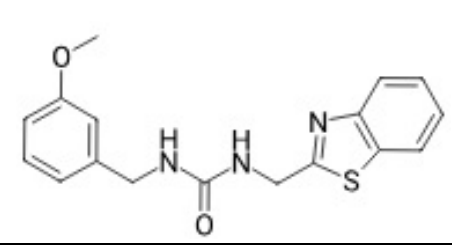	PRMT4 n.e.	-	n.s.
**K001JS009**	PRMT4 i	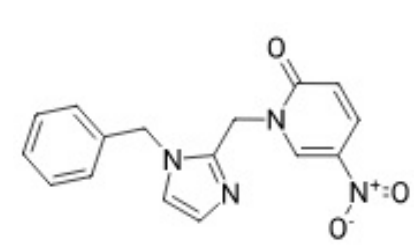	PRMT4 n.e.	-	n.s.
**K001JS011**	PRMT4 i	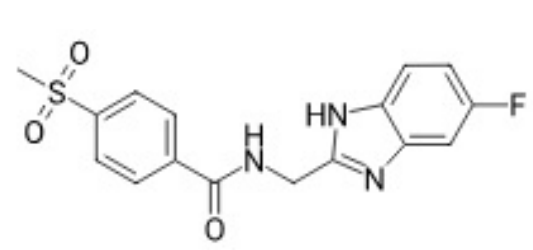	PRMT4 n.e.	-	n.s.
**K001JS012**	PRMT4 i	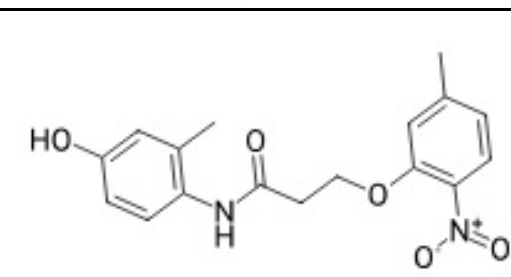	PRMT4 n.e.	-	n.s.

Table S2 continued

Compound	Intended target	2D structure	Experimental result	Efficacy ±Δ%	P-value
**K400JK003**	PRMT4 i	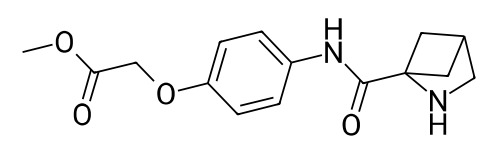	PRMT4 n.e.	-	n.s.
**K400JK004**	PRMT4 i	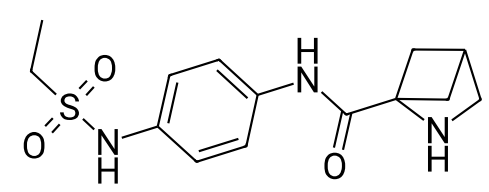	PRMT4 a	+8%	*
**K400JK006**	PRMT4 i	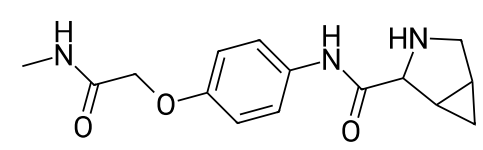	PRMT4 n.e.	-	n.s.
**K400JK009**	PRMT4 i	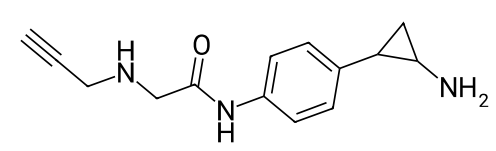	PRMT4 n.e.	-	n.s.
**K400JK010**	PRMT4 i	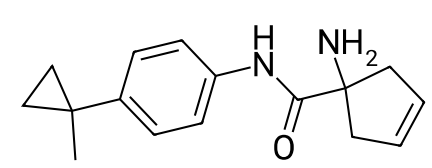	PRMT4 i	-13%	*
**K400JK011**	PRMT4 i	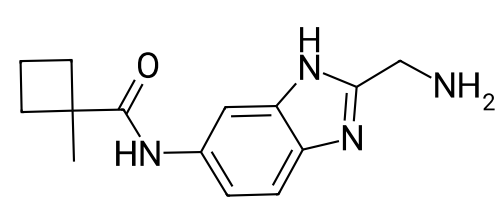	PRMT4 i	-10%	**
**K400JK012**	PRMT4 i	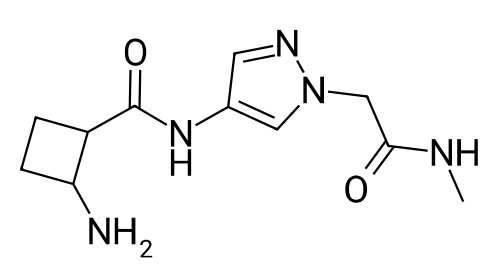	PRMT4 n.e.	-	n.s.
**K400JK015**	PRMT4 i	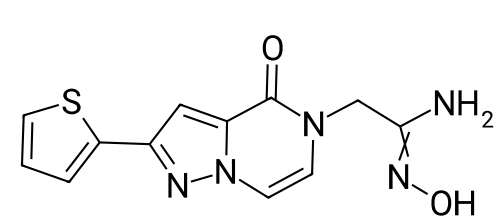	PRMT4 n.e.	-	n.s.
**K400JK016**	PRMT4 i	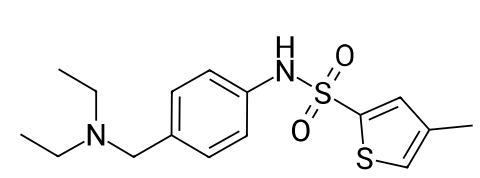	PRMT4 n.e.	-	n.s.
**K002FT001**	PRMT6 i	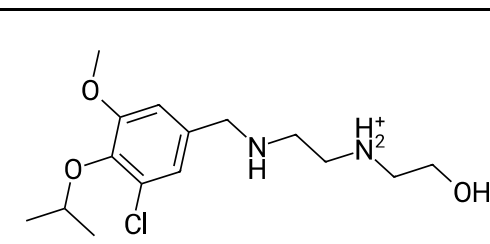	PRMT6 n.e.	-	n.s.
**K002FT002**	PRMT6 i	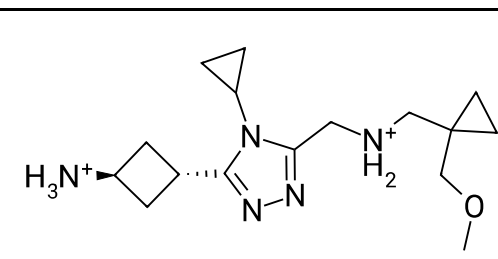	PRMT6 n.e..	-	n.s.
**K002FT003**	PRMT6 i	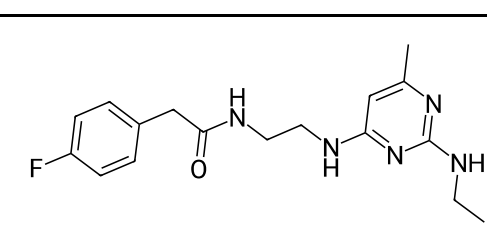	PRMT6 n.e.	-	n.s.

Table S2 continued

Compound	Intended target	2D structure	Experimental result	Efficacy ±∆%	P-value
**K002FT004**	PRMT6 i	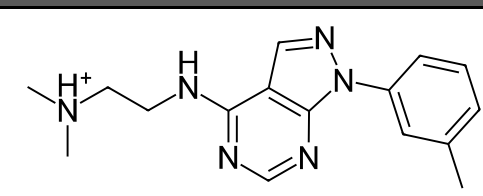	PRMT6 n.e.	-	n.s.
**K002FT005**	PRMT6 i	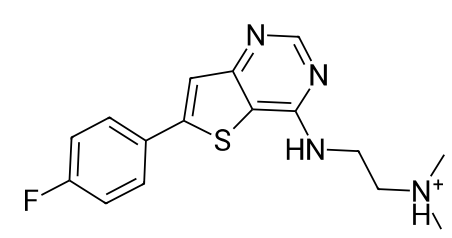	PRMT6 n.e.	-	n.s.
**K002FT006**	PRMT6 i	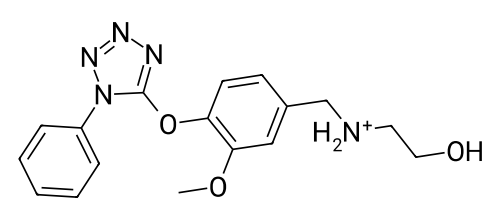	PRMT6 n.e.	-	n.s.
**K002FT007**	PRMT6 i	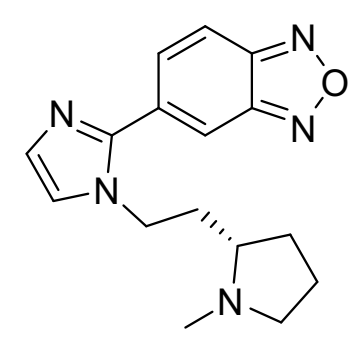	PRMT6 n.e.	-	n.s.
**K002FT008**	PRMT6 i	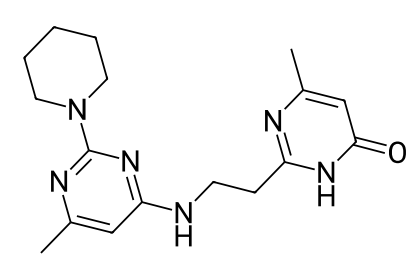	PRMT6 i	-10%	*
**K002FT009**	PRMT6 i	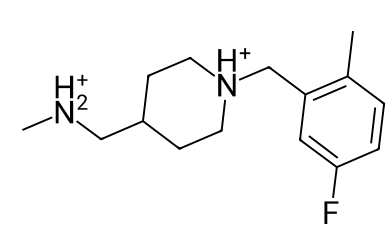	PRMT6 n.e.	-	n.s.
**K002FT010**	PRMT6 i	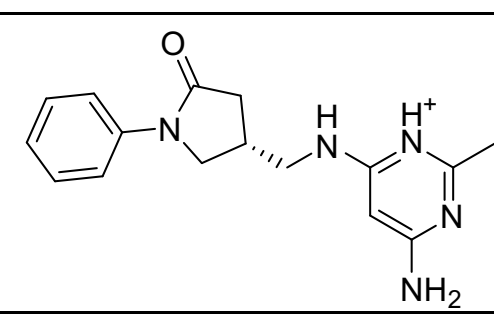	PRMT6 n.e.	-	n.s.
**K002FT011**	PRMT6 i	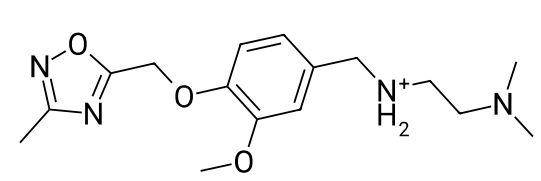	PRMT6 n.e.	-	n.s.
**K002FT012**	PRMT6 i	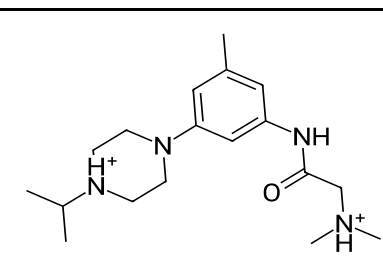	PRMT6 n.e.	-	n.s.
**K002FT013**	PRMT6 i	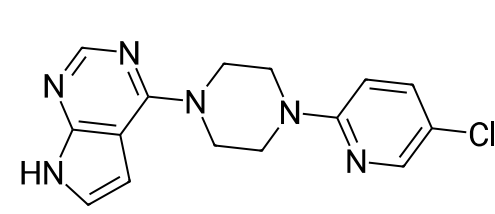	PRMT6 n.e.	-	n.s.

Table S2 continued

Compound	Intended target	2D structure	Experimental result	Efficacy ±∆%	P-value
**K002FT014**	PRMT6 **i**	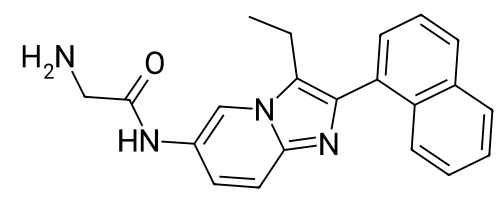	PRMT6 **n.e.**	-	n.s.
**K002FT015**	PRMT6 **i**	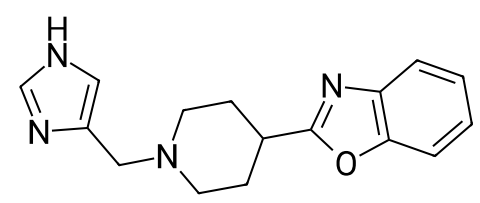	PRMT6 **n.e.**	-	n.s.
**K002FT016**	PRMT6 **i**	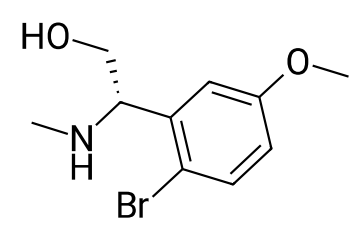	PRMT6 **n.e.**	-	n.s.
**K002FT018**	PRMT6 **i**	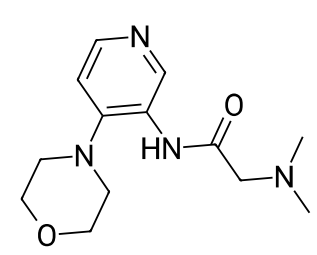	PRMT6 **n.e.**	-	n.s.
**K002JS001**	PRMT6 **i**	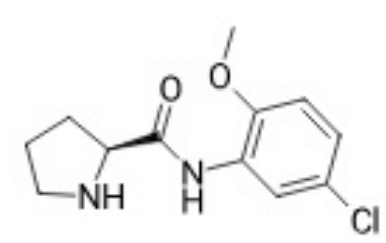	PRMT6 **n.e.**	-	n.s.
**K002JS002**	PRMT6 **i**	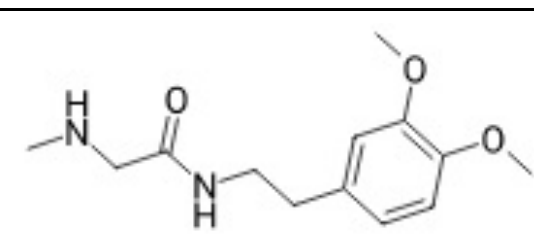	PRMT6 **n.e.**	-	n.s.
**K002JS005**	PRMT6 **i**	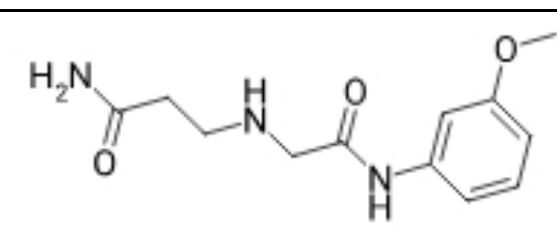	PRMT6 **n.e.**	-	n.s.
**K002JS009**	PRMT6 **i**	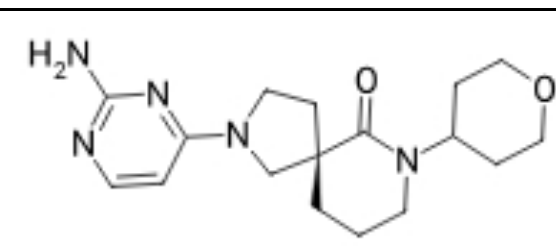	PRMT6 **n.e.**	-	n.s.
**K002JS010**	PRMT6 **i**	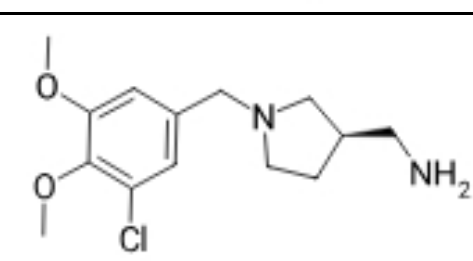	PRMT6 **n.e.**	-	n.s.
**K002JS012**	PRMT6 **i**	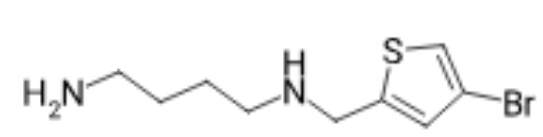	PRMT6 **n.e.**	-	n.s.
**K002JS015**	PRMT6 **i**	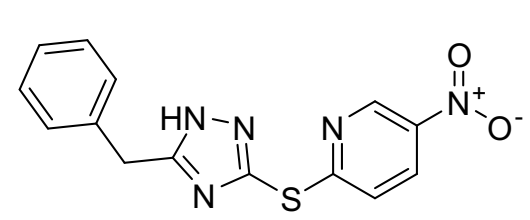	PRMT6 **n.e.**	-	n.s.
**K002JS017**	PRMT6 **i**	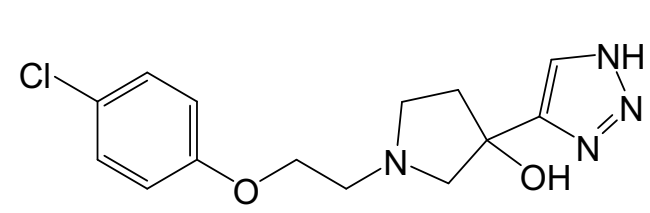	PRMT6 **n.e.**	-	n.s.

Table S2 continued

Compound	Intended target	2D structure	Experimental result	Efficacy ±Δ%	P-value
**K002JS018**	PRMT6 **i**	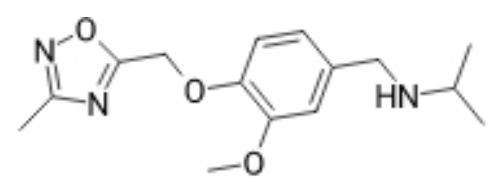	PRMT6 **n.e.**	-	n.s.
**K600JK005**	PRMT6 **i**	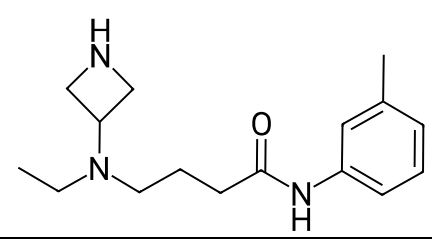	PRMT6 **n.e.**	-	n.s.
**K600JK006**	PRMT6 **i**	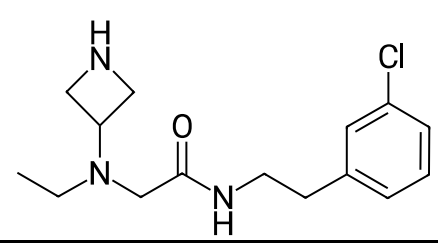	PRMT6 **n.e.**	-	n.s.
**K600JK007**	PRMT6 **i**	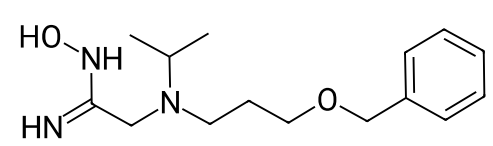	PRMT6 **n.e.**	-	n.s.
**K600JK008**	PRMT6 **i**	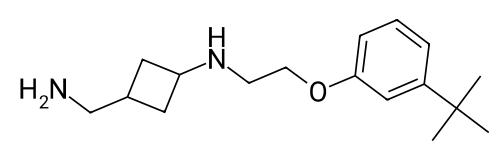	PRMT6 **n.e.**	-	n.s.
**K600JK012**	PRMT6 **i**	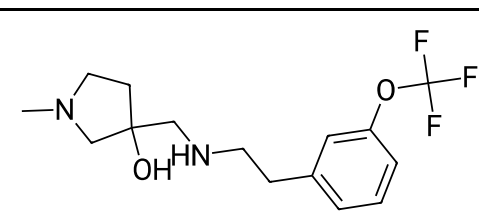	PRMT6 **n.e.**	-	n.s.
**K600JK013**	PRMT6 **i**	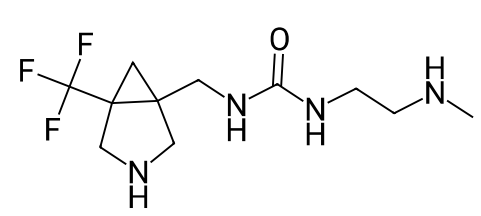	PRMT6 **n.e.**	-	n.s.

Table S3: Summary of TR-07 and 45 derivates as well as new molecules, with respect to their effects on the catalytic activity of PRMT1 and other type I PRMTs.

Compound names, intended PRMT to inhibit (**i**) or activate (**a**), SAR series, 2D structure, experimental result of the F-PRMT assays, the compounds' efficacy in altering PRMT activity (±Δ%), and the corresponding p-values of efficacy are summarized. The PRMT activity upon compound treatment (100 µM) was calculated as % relative to the activity in the presence of solvent control (untreated), for which the activity was set to 100% (n≥3). Changes upon compound treatment (=efficacy) were expressed as mean % differences of activation (+Δ%) or inhibition of catalysis (-Δ%). In case of an activatory (**a**) effect for the experimental result, the compound-treated activity was higher by +Δ% than untreated, whereas in case of an inhibitory (**i**) effect, the compound-treated activity was lower by -Δ% than untreated. Compounds, which did not significantly affect the catalytic activity (less than ±6% change or not significant p-value), are marked with **n.e.** (no effect) for the experimental result and with − for the efficacy. The label **n.t.** indicates that the compound was not tested for its influence on the respective PRMT activity. P-values of efficacy were calculated using the student *t*-test (n.s.: not significant; *: p ≤0.05,**: p ≤0.01, ***: p ≤0.001, ****: p ≤0.0001).

	Name	Intended target	SAR series	2D structure	Experimental result	Efficacy ±Δ%	P-value
	**TR-07**	PRMT6 **i**		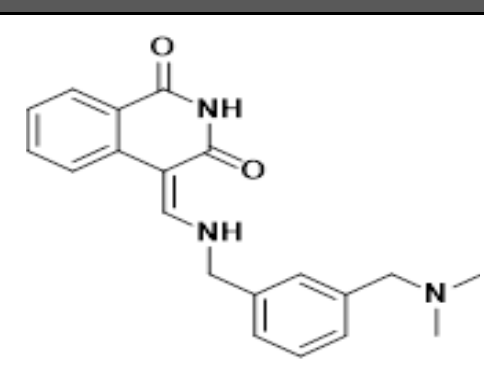	PRMT1 **a**<br>PRMT3 **n.e.**<br>PRMT4 **n.e.**<br>PRMT6 **n.e.**	+45%<br>-<br>-<br>-	****<br>n.s.<br>n.s.<br>n.s.
1	**TR-02**	PRMT1 **a**	I	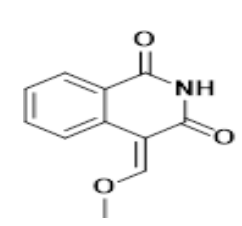	PRMT1 **n.e.**<br>PRMT3 **i**<br>PRMT4 **n.e.**<br>PRMT6 **i**	-<br>-22%<br>-<br>-12%	n.s.<br>***<br>n.s.<br>****
2	**TR-03**	PRMT1 **a**	I	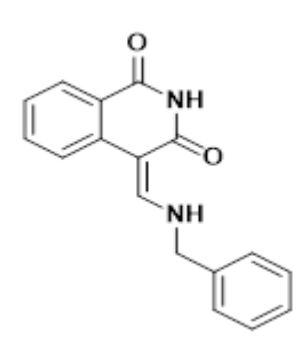	PRMT1 **i**<br>PRMT3 **n.e.**<br>PRMT4 **n.e.**<br>PRMT6 **n.e.**	-13%<br>-<br>-<br>-	**<br>n.s.<br>n.s.<br>n.s.
3	**TR-010**	PRMT1 **a**	IIIA	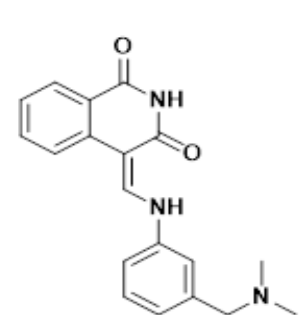	PRMT1 **a**<br>PRMT3 **a**<br>PRMT4 **n.e.**<br>PRMT6 **n.e.**	+75%<br>+45%<br>-<br>-	****<br>****<br>n.s.<br>n.s.
4	**TR-014**	PRMT1 **a**	I	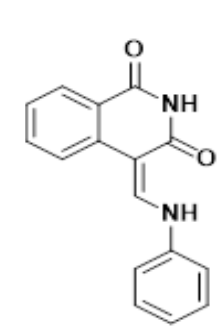	PRMT1 **n.e.**<br>PRMT3 **n.e.**<br>PRMT4 **n.e.**<br>PRMT6 **n.e.**	-<br>-<br>-<br>-	n.s.<br>n.s.<br>n.s.<br>n.s.

Table S3 continued

	Name	Intended target	SAR series	2D structure	Experimental result	Efficacy ±Δ%	P-value
5	**TR-018**	PRMT1 a	I	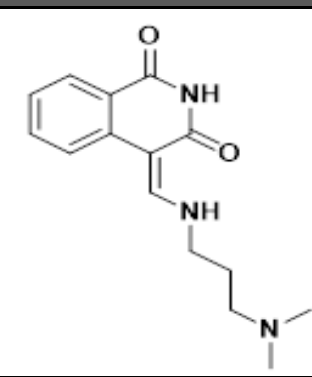	PRMT1 a<br>PRMT3 n.e.<br>PRMT4 n.e.<br>PRMT6 n.e.	+10%<br>-<br>-<br>-	**<br>n.s.<br>n.s.<br>n.s.
6	**TR-021**	PRMT1 a	IIIA	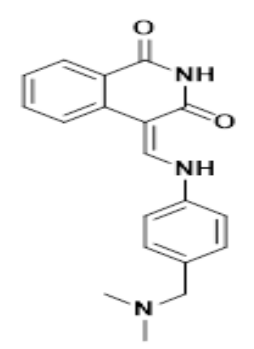	PRMT1 a<br>PRMT3 a<br>PRMT4 n.e.<br>PRMT6 n.e.	+70%<br>+50%<br>-<br>-	****<br>****<br>n.s.<br>n.s.
7	**TR-022**	PRMT1 a	IIIA	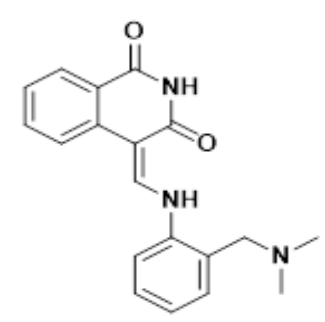	PRMT1 a<br>PRMT3 n.e.<br>PRMT4 n.e.<br>PRMT6 n.e.	+40%<br>-<br>-<br>-	****<br>n.s.<br>n.s.<br>n.s.
8	**TR-030**	PRMT1 a	I	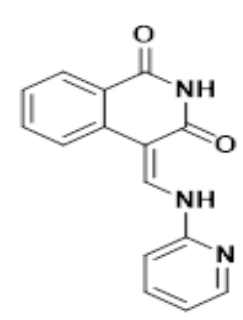	PRMT1 n.e.<br>PRMT3 n.e.<br>PRMT4 n.e.<br>PRMT6 n.e.	-<br>-<br>-<br>-	n.s.<br>n.s.<br>n.s.<br>n.s.
9	**TR-031**	PRMT1 a	I	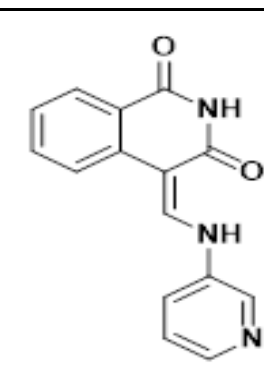	PRMT1 n.e.<br>PRMT3 i<br>PRMT4 i<br>PRMT6 i	-<br>-20%<br>-6%<br>-10%	n.s.<br>***<br>**<br>*
10	**TR-032**	PRMT1 a	I	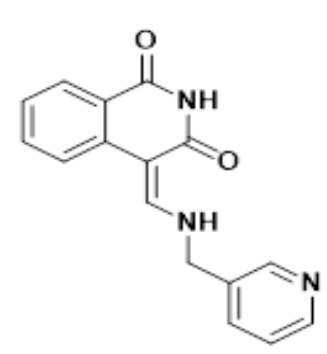	PRMT1 a<br>PRMT3 n.e.<br>PRMT4 n.e.<br>PRMT6 a	+14%<br>-<br>-<br>+14%	*<br>-<br>-<br>*
11	**TR-033**	PRMT1 a	IV	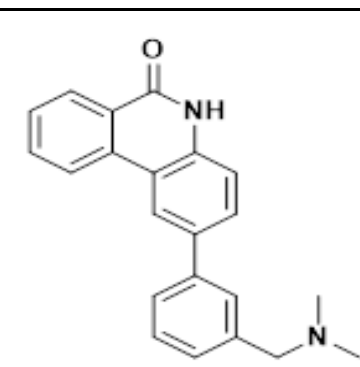	PRMT1 a<br>PRMT3 a<br>PRMT4 n.e.<br>PRMT6 a	+125%<br>+90%<br>-<br>+45%	****<br>****<br>n.s.<br>****
12	**TR-036**	PRMT1 a	IIIA	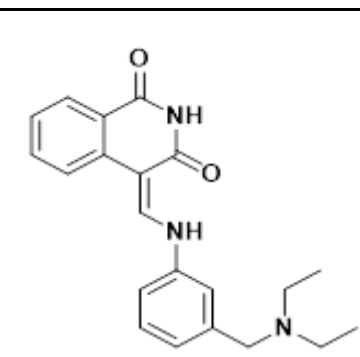	PRMT1 a<br>PRMT3 a<br>PRMT4 n.e.<br>PRMT6 a	+115%<br>+90%<br>-<br>+15%	****<br>****<br>n.s.<br>**

Table S3 continued

	Name	Intended target	SAR series	2D structure	Experimental result	Efficacy ±∆%	P-value
13	**TR-038**	PRMT1 **a**	III$_A$	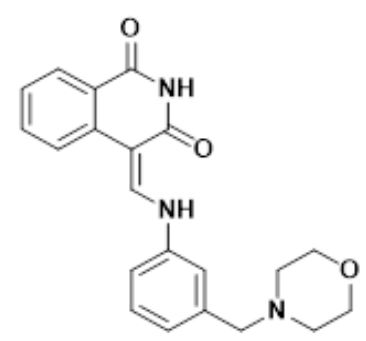	PRMT1 **i**<br>PRMT3 **i**<br>PRMT4 **i**<br>PRMT6 **i**	-35%<br>-20%<br>-10%<br>-22%	****<br>**<br>**<br>***
14	**ICTR-015**	PRMT1 **a**	III$_B$	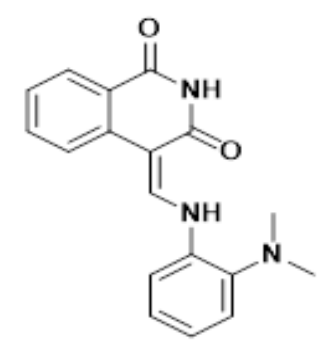	PRMT1 **n.e.**<br>PRMT3 **n.e.**<br>PRMT4 **n.e.**<br>PRMT6 **n.t.**	-<br>-<br>-	n.s.<br>n.s.<br>n.s.
15	**ICTR-019**	PRMT1 **a**	III$_B$	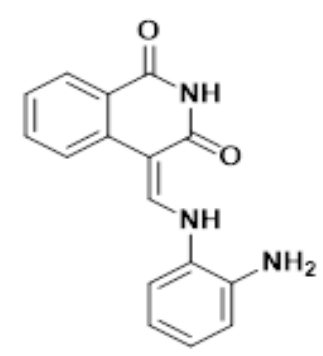	PRMT1 **n.e.**<br>PRMT3 **n.e.**<br>PRMT4 **n.e.**<br>PRMT6 **n.t.**	-<br>-<br>-	n.s.<br>n.s.<br>n.s.
16	**ICTR-022**	PRMT1 **a**	III$_B$	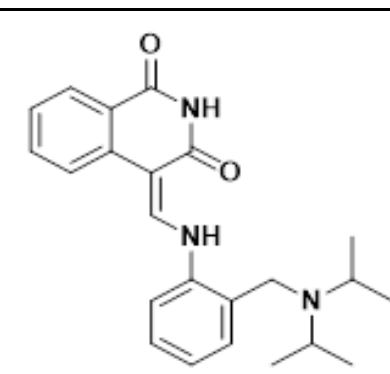	PRMT1 **a**<br>PRMT3 **n.e.**<br>PRMT4 **n.e.**<br>PRMT6 **n.t.**	+13%<br>-<br>-	**<br>n.s.<br>n.s.
17	**ICTR-023**	PRMT1 **a**	III$_B$	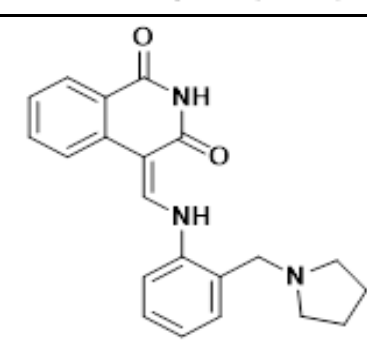	PRMT1 **a**<br>PRMT3 **a**<br>PRMT4 **n.e.**<br>PRMT6 **n.t.**	+16%<br>+30%<br>-	***<br>**<br>n.s.
18	**ICTR-028**	PRMT1 **a**	III$_B$	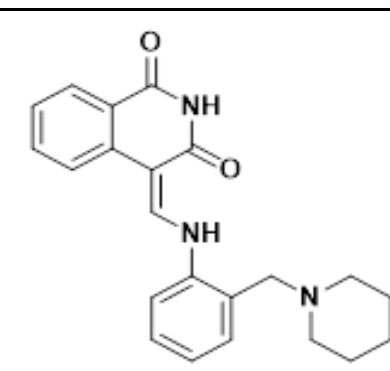	PRMT1 **i**<br>PRMT3 **n.t.**<br>PRMT4 **n.t.**<br>PRMT6 **n.t.**	-15%	**
19	**ICTR-035**	PRMT1 **a**	III$_B$	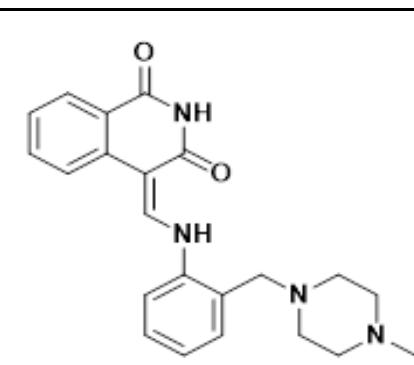	PRMT1 **a**<br>PRMT3 **a**<br>PRMT4 **a**<br>PRMT6 **a**	+95%<br>+85%<br>+15%<br>+35%	****<br>***<br>**<br>***
20	**ICTR-036**	PRMT1 **a**	III$_B$	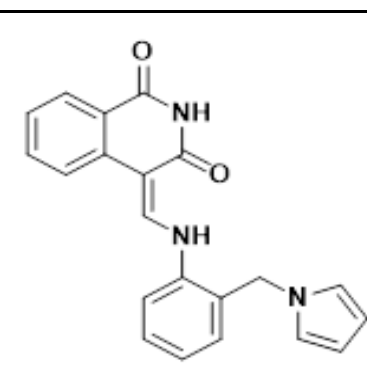	PRMT1 **i**<br>PRMT3 **n.e.**<br>PRMT4 **n.e.**<br>PRMT6 **n.t.**	-7%<br>-<br>-	*<br>n.s.<br>n.s.

Table S3 continued

	Name	Intended target	SAR series	2D structure	Experimental result	Efficacy ±Δ%	P-value
21	**ICTR-040**	PRMT1 **a**	III$_B$	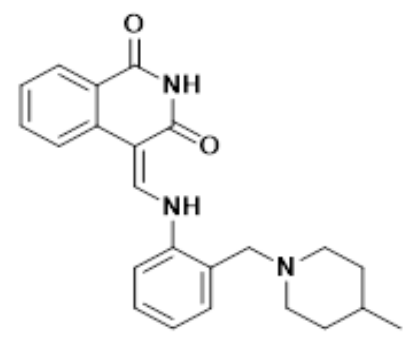	PRMT1 **n.e.**<br>PRMT3 **n.t.**<br>PRMT4 **n.t.**<br>PRMT6 **n.t.**	-	n.s.
22	**ICTR-042**	PRMT1 **a**	III$_B$	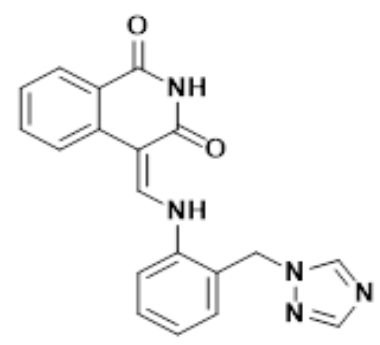	PRMT1 **n.e.**<br>PRMT3 **n.e.**<br>PRMT4 **n.e.**<br>PRMT6 **n.t.**	-<br>-<br>-	n.s.<br>n.s.<br>n.s.
23	**ICTR-050**	PRMT1 **a**	III$_B$	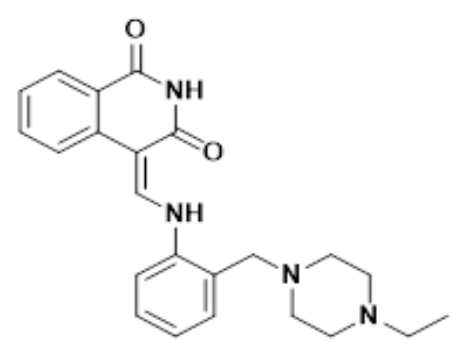	PRMT1 **a**<br>PRMT3 **a**<br>PRMT4 **a**<br>PRMT6 **a**	+80%<br>+94%<br>+30%<br>+12%	****<br>****<br>**<br>*
24	**ICTR-052**	PRMT1 **a**	II	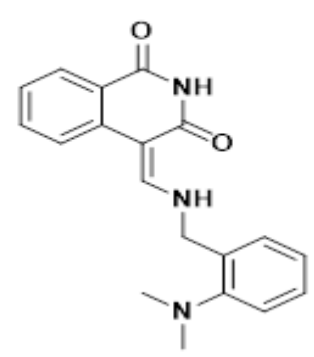	PRMT1 **n.e.**<br>PRMT3 **n.e.**<br>PRMT4 **n.e.**<br>PRMT6 **n.t.**	-<br>-<br>-	n.s.<br>n.s.<br>n.s.
25	**ICTR-053**	PRMT1 **a**	III$_B$	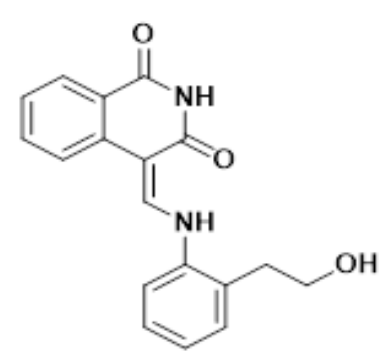	PRMT1 **n.e.**<br>PRMT3 **a**<br>PRMT4 **n.e.**<br>PRMT6 **n.t.**	-<br>+30%<br>-	n.s.<br>**<br>n.s.
26	**ICTR-058**	PRMT1 **a**	III$_B$	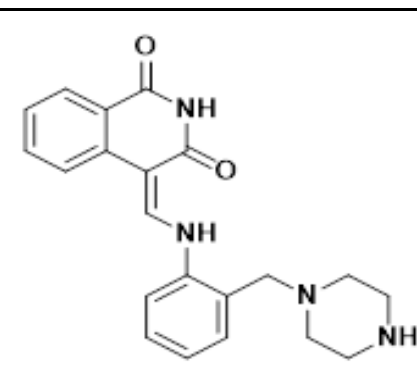	PRMT1 **a**<br>PRMT3 **a**<br>PRMT4 **n.e.**<br>PRMT6 **a**	+90%<br>+100%<br>-<br>+40%	****<br>***<br>n.s.<br>**
27	**ICTR-059**	PRMT1 **a**	III$_B$	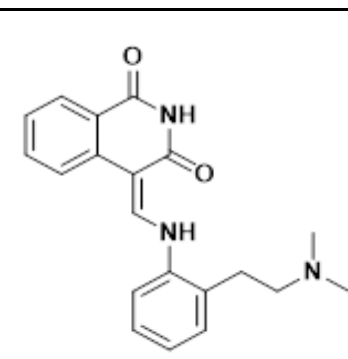	PRMT1 **a**<br>PRMT3 **a**<br>PRMT4 **n.e.**<br>PRMT6 **n.e.**	+100%<br>+45%<br>-<br>-	****<br>****<br>n.s.<br>n.s.
28	**ICTR-060**	PRMT1 **a**	III$_B$	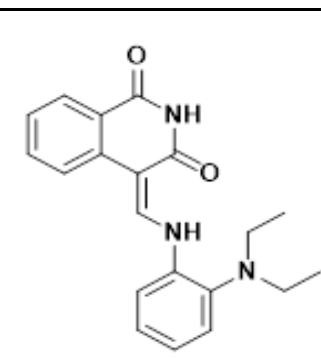	PRMT1 **n.e.**<br>PRMT3 **n.t.**<br>PRMT4 **n.t.**<br>PRMT6 **n.t.**	-	n.s.

Table S3 continued

	Name	Intended target	SAR series	2D structure	Experimental result	Efficacy ±Δ%	P-value
29	**ICTR-063**	PRMT1 a	II	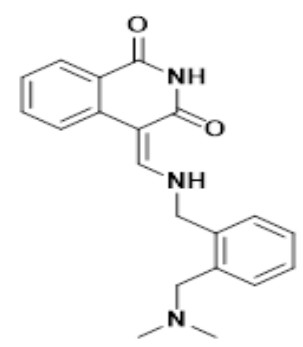	PRMT1 a<br>PRMT3 n.t.<br>PRMT4 n.t.<br>PRMT6 n.t.	+20%	***
30	**ICTR-076**	PRMT1 a	IIIA	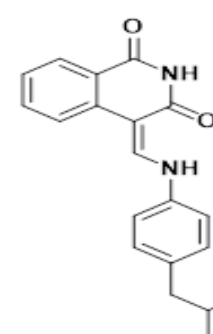	PRMT1 n.e.<br>PRMT3 n.t.<br>PRMT4 n.t.<br>PRMT6 n.t.	-	n.s.
31	**ICTR-078**	PRMT1 a	II	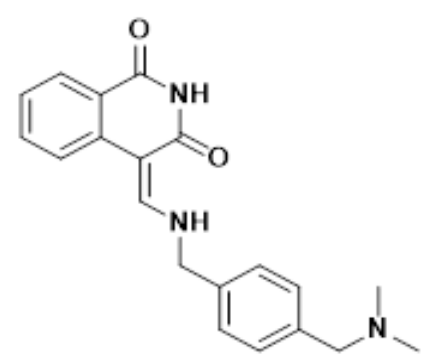	PRMT1 a<br>PRMT3 n.e.<br>PRMT4 n.e.<br>PRMT6 n.e.	+40%<br>-<br>-<br>-	****<br>n.s.<br>n.s.<br>n.s.
32	**ICTR-086**	PRMT1 a	IIIB	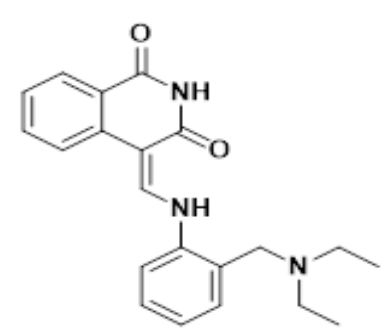	PRMT1 a<br>PRMT3 n.t.<br>PRMT4 n.t.<br>PRMT6 n.t.	+11%	***
33	**ICTR-094**	PRMT1 a	IV	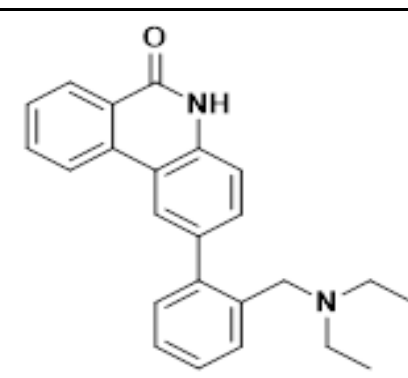	PRMT1 a<br>PRMT3 n.t.<br>PRMT4 n.t.<br>PRMT6 n.t.	+20%	**
34	**ICTR-096**	PRMT1 a	IV	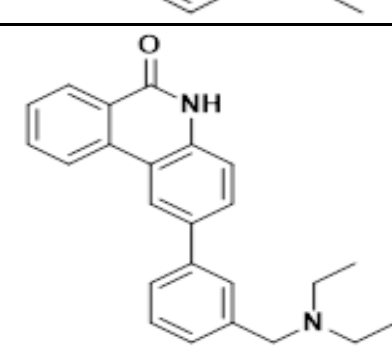	PRMT1 a<br>PRMT3 a<br>PRMT4 n.e.<br>PRMT6 a	+90%<br>+70%<br>-<br>+50%	****<br>****<br>n.s.<br>****
35	**ICTR-108**	PRMT1 a	II	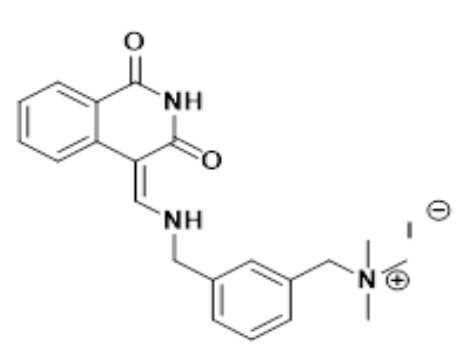	PRMT1 a<br>PRMT3 n.t.<br>PRMT4 n.t.<br>PRMT6 n.t.	+15%	*
36	**ICTR-119**	PRMT1 a	II	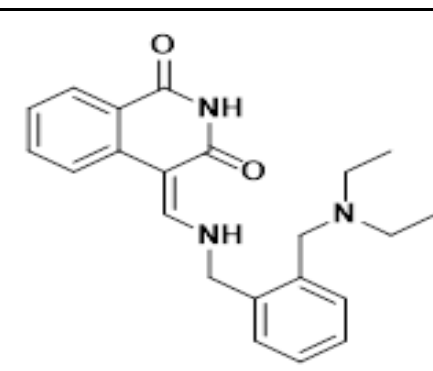	PRMT1 n.e.<br>PRMT3 n.t.<br>PRMT4 n.t.<br>PRMT6 n.t.	-	n.s.

Table S3 continued

	Name	Intended target	SAR series	2D structure	Experimental result	Efficacy ±Δ%	P-value
37	**ICTR-120**	PRMT1 **a**	II	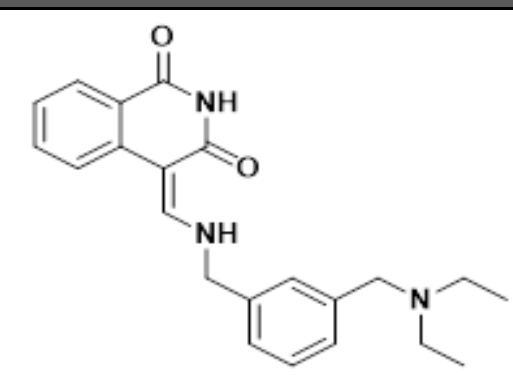	PRMT1 **a**<br>PRMT3 **a**<br>PRMT4 **n.e.**<br>PRMT6 **n.e.**	+60%<br>+45%<br>-<br>-	****<br>**<br>n.s.<br>n.s.
38	**K500JK001**	PRMT1 **a**	V	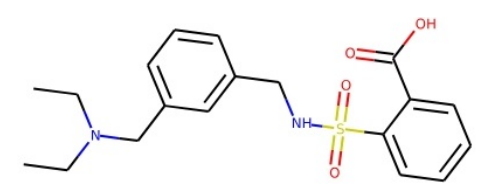	PRMT1 **a**<br>PRMT3 **n.t.**<br>PRMT4 **n.t.**<br>PRMT6 **n.t.**	+15%	**
39	**K500JK003**	PRMT1 **a**	V	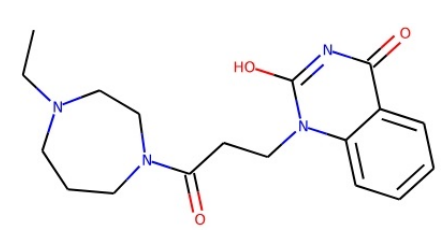	PRMT1 **n.e.**<br>PRMT3 **n.t.**<br>PRMT4 **n.t.**<br>PRMT6 **n.t.**	-	n.s.
40	**K500JK004**	PRMT1 **a**	V	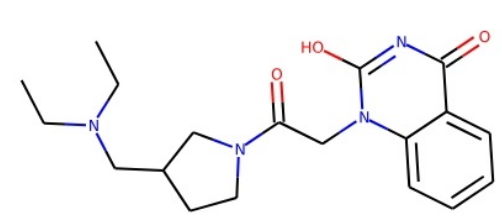	PRMT1 **n.e.**<br>PRMT3 **n.t.**<br>PRMT4 **n.t.**<br>PRMT6 **n.t.**	-	n.s.
41	**K500JK005**	PRMT1 **a**	V	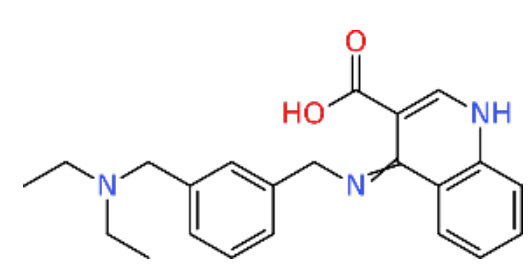	PRMT1 **n.e.**<br>PRMT3 **n.t.**<br>PRMT4 **n.t.**<br>PRMT6 **n.t.**	-	n.s.
42	**K500JK006**	PRMT1 **a**	V	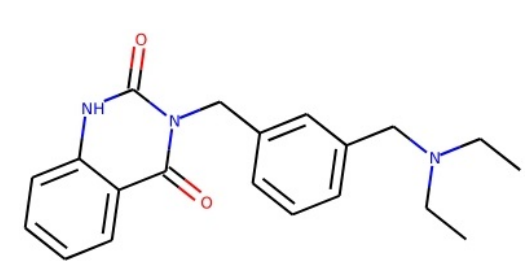	PRMT1 **n.e.**<br>PRMT3 **n.t.**<br>PRMT4 **n.t.**<br>PRMT6 **n.t.**	-	n.s.
43	**K500JK007**	PRMT1 **a**	V	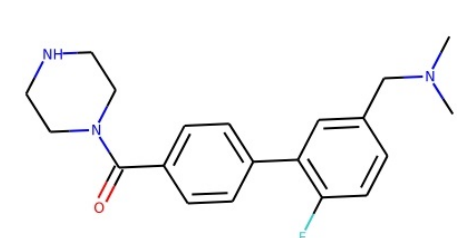	PRMT1 **a**<br>PRMT3 **n.t.**<br>PRMT4 **n.t.**<br>PRMT6 **n.t.**	+15%	**
44	**K500JK008**	PRMT1 **a**	V	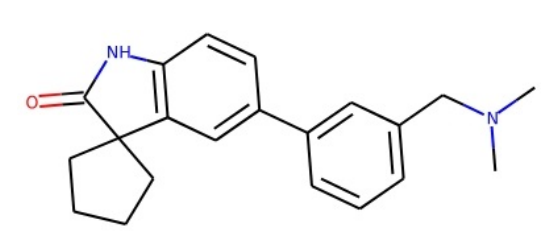	PRMT1 **a**<br>PRMT3 **n.t.**<br>PRMT4 **n.t.**<br>PRMT6 **n.t.**	+17%	*

Table S3 continued

	Name	Intended target	SAR series	2D structure	Experimental result	Efficacy ±∆%	P-value
45	**K500JK010**	PRMT1 **a**	V	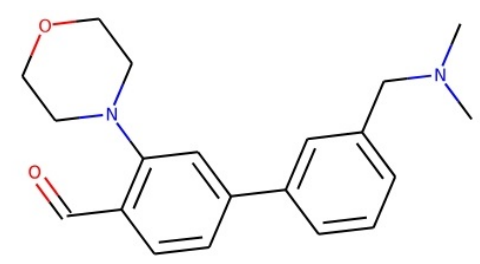	PRMT1 **a**<br>PRMT3 **n.t.**<br>PRMT4 **n.t.**<br>PRMT6 **n.t.**	+20%	**

General Working Procedure (GWP)

GWP 1: Nucleophilic Substitution under Microwave Irradiation

In a microwave vessel (20.0 mL), the corresponding bromomethyl nitrobenzene or bromomethyl benzonitrile (1.00 equiv), the corresponding amine (1.10 equiv), and K_2CO_3 (1.00 equiv) were dissolved in MeCN (3 mL/mmol) and stirred under microwave conditions (120 °C, 150 W) for 1-2 hours. After complete conversion (as determined by TLC) and cooling to room temperature, the mixture was dissolved in H_2O and extracted several times with DCM. The combined organic phase was washed with brine, dried over $MgSO_4$, filtered, and the solvent was removed under reduced pressure. If necessary, the crude product was purified according to the specified method.

GWP 2: Iron Reduction of 3/4-Substituted Nitrobenzylamines

A suspension of NH_4Cl (0.50 equiv) and iron (10.0 equiv) in H_2O (7.5 mL/mmol) was stirred at 50 °C for 1 hour and then mixed with a solution of the corresponding nitro compound (1.00 equiv) dissolved in MeOH (7.5 mL/mmol) and stirred for an additional 3 hours at 50 °C after the dropwise addition of HCl (conc.) (0.5 mL/mmol). After the reaction was complete (as determined by TLC and staining with Ehrlich's reagent) and cooling to room temperature, a basic pH value (8–14) was adjusted with K_2CO_3. or NaOH. The resulting mixture was filtered over Celite, and the pad was washed with MeOH. After removal of MeOH under reduced pressure, DCM was added and the organic phase was washed with brine, dried over $MgSO_4$, filtered, and the solvent was removed under reduced pressure. The crude product was immediately further processed in the next step without any further purification.

GWP 3: Iron Reduction of 2-Substituted Nitrobenzylamines

A suspension of NH_4Cl (0.50 equiv) and iron (10.0 equiv) in AcOH (20.0 mL) was stirred at 60 °C for 1 hour and then mixed with a solution of the corresponding nitro compound (1.00 equiv) in MeOH (13.5 mL/mmol) and stirred for another 3 hours at 60 °C. After the reaction was complete (as determined by TLC and staining with Ehrlich's reagent), either a basic pH value (8–14) was adjusted with K_2CO_3 or NaOH and the resulting mixture was filtered over Celite, or directly acid-filtered with washing of the pad with MeOH. MeOH was then removed under reduced pressure, DCM was added and subsequently the combined organic phase was washed with brine, dried over $MgSO_4$, filtered, and the solvent was removed under reduced pressure. The crude product was immediately further processed in the next step without any further purification.

GWP 4: Reduction of the Nitro Group with Ammonium Formate

To a solution of the corresponding nitro compound (1.00 equiv) in EtOH (20 mL/mmol), Pd/C (0.075 equiv, 10% Pd on activated carbon) and Ammonium formate (7.0 equiv) were added and stirred for 1 hour. A hydrogen atmosphere was created using a balloon, and the mixture was stirred for an additional 10 minutes to 2 hours at

room temperature. After the reaction was complete (as determined by TLC and staining with Ehrlich's reagent), the mixture was filtered over a Celite pad, washed with MeOH, and the solvent was removed under reduced pressure. The residue was dissolved in DCM, washed with H_2O, dried over $MgSO_4$, filtered, and the solvent was removed under reduced pressure. The crude product was immediately further processed in the next step without any further purification.

GWP 5: Reduction of the Nitrile Group with $LiAlH_4$

In a preheated flask the corresponding nitrile (1.00 equiv) was dissolved in dry THF (10 mL/mmol) under argon atmosphere, cooled to 0 °C in an ice bath, then a 2.4 M $LiAlH_4$ solution in THF (1.50 equiv) was added dropwise and stirred for 3 hours at 0 °C. After the reaction was complete, sodium sulfate decahydrate was added and stirred for an additional 30 minutes at 0 °C. After warming to room temperature and stirring for another hour, the reaction mixture was filtered over Celite and the solvent was removed using a rotary evaporator. The crude product was immediately further processed in the next reaction step without further purification.

GWP 6: Coupling with Precursor TR-02

A suspension of **TR-02** (250 mg, 1.23 mmol, 1.00 equiv) and the corresponding amine (1.30 equiv; if the corresponding hydrochlorides are used, triethylamine 1.30 equiv of Et_3N is also added) in MeOH (4.0 mL/mmol) was stirred under microwave conditions (125 °C, 300 W) for 30 minutes. After cooling to room temperature, the reaction mixture was filtered, the precipitate was washed with MeOH and Et_2O, and dried under vacuum (10^{-3} mbar, 50 °C). If no precipitate formed, the solvent was removed under reduced pressure, the crude product was adsorbed onto silica gel, and purified by MPLC according to the specified method.

Synthesis of K002FT017, alias TR-07[a]

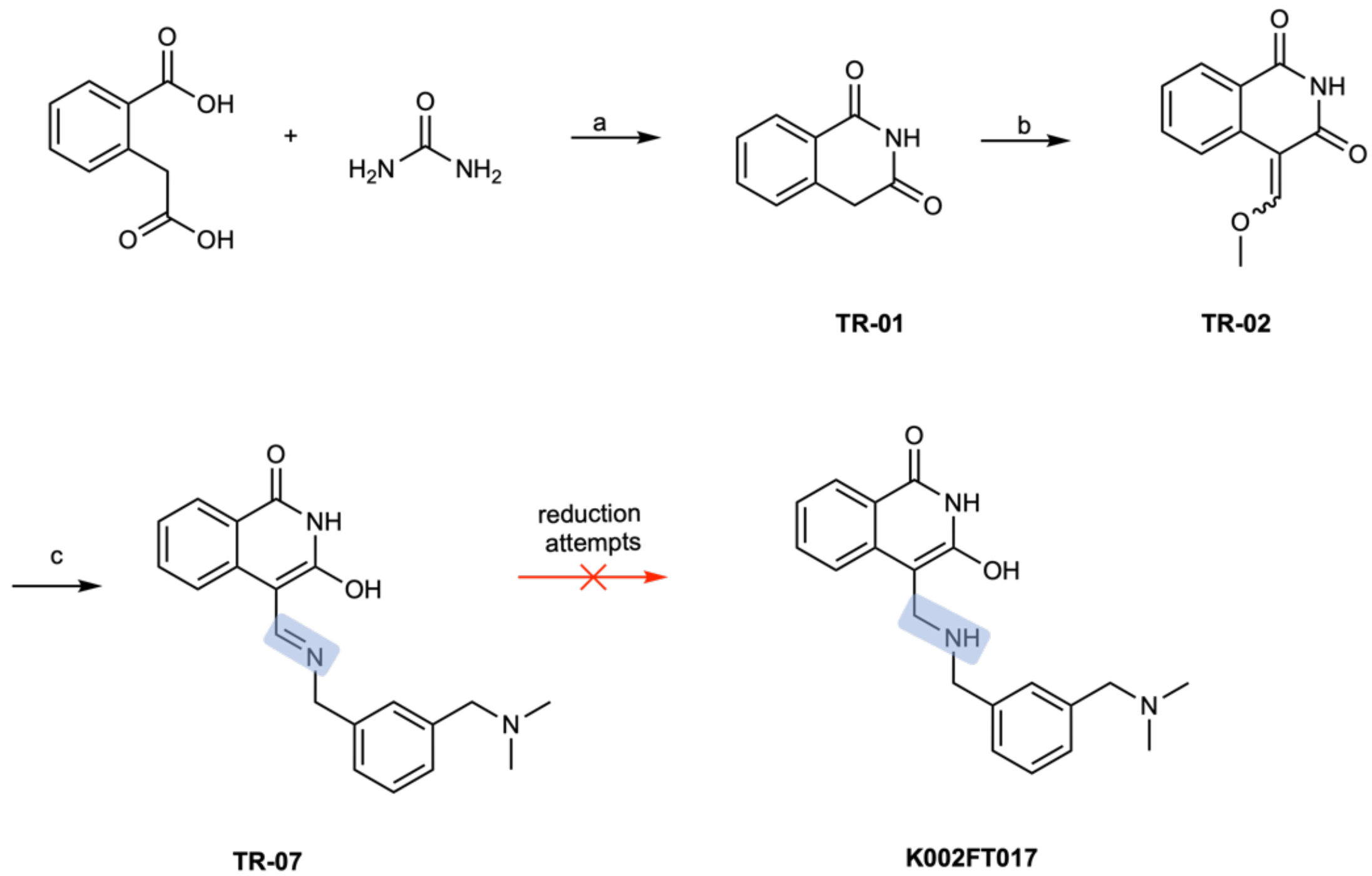

[a] Reagents and conditions: a) AcOH, 110 °C, 3 h, 76%; b) trimethyl orthoformate, Ac_2O, DMF, @MW 125 °C, 30 min, 84%; c) 1-[3-(aminomethyl)phenyl]-N,N-dimethylmethaneamine *2HCl, Et_3N, MeOH, @MW 125 °C, 30 min, 44%.

Isoquinoline-1,3(2*H*,4*H*)-dione (TR-01)

Homophthalic acid (5.00 g, 27.80 mmol, 1.00 equiv) was suspended in AcOH and heated to 110 °C. Subsequently, urea (11.90 g, 197.40 mmol, 7.10 equiv) was added in portions over 2 minutes and the reaction mixture was stirred at 110 °C for 3 h. After cooling to room temperature, the mixture was poured into 300 mL of ice water and stirred for an additional 30 minutes. The resulting precipitate was filtered, washed with H_2O (2 x 50.00 mL) and MeOH (3 x 50.00 mL) and then dried under high vacuum. Compound **TR-01** (3.40 g, 21.10 mmol) was obtained as a colorless to pale yellow solid with a yield of 76%. mp 230.2°C.[1] ^{1}H-NMR (500 MHz, DMSO-d_6, 300 K): δ_H (ppm) = 11.26 (s, 1H), 8.01 (ddd, ^3J = 7.7 Hz, ^4J = 1.4 Hz, ^5J = 0.6 Hz, 1H), 7.64 (td, ^3J = 7.5 Hz, ^4J = 1.4 Hz, 1H), 7.45 (td, ^3J = 7.6 Hz, ^4J = 1.2 Hz, 1H), 7.38 (ddd, ^3J = 7.7 Hz, ^4J = 1.2 Hz, ^5J = 0.6 Hz, 1H), 4.03 (s, 2H,) ppm.[2] ^{13}C-NMR (125 MHz, DMSO-d_6, 300 K): δ_C (ppm) = 170.9, 165.3, 136.6, 133.4, 127.8, 127.4, 127.1, 125.0, 35.9.[2] HR-MS: calcd for $C_9H_6N_1O_2$ [M-H]$^-$ 160.0404; found: 160.0403.[2] Purity was determined by qNMR using maleic acid as an internal standard: 94.6%.

(E/Z)-4-(Methoxymethylene)isoquinoline-1,3(2H,4H)-dione (TR-02)

To a suspension of **TR-01** (3.00 g, 18.6 mmol, 1.00 equiv) in DMF (8.00 mL), trimethyl orthoformate (8.14 mL, 74.4 mmol, 4.00 equiv) and Ac_2O (12.0 mL, 0.65 mL/mmol) were added and stirred for 30 minutes under microwave conditions (125 °C, 300 W). After cooling to room temperature, Et_2O (300 mL) was added and the mixture was stirred for an additional 5 minutes at room temperature. The resulting precipitate was filtered, washed with Et_2O (3 x 50.0 mL) and dried under high vacuum (10^{-3} mbar) to obtain **TR-02** (3.19 g, 15.7 mmol) as an orange to yellow solid with a yield of 84%. mp decomposition at > 260 °C.[1] ^{1}H-NMR (500 MHz, DMSO-d_6, 300 K): δ_H (ppm) = 11.24 (s, 1H), 8.25 (d, ^3J = 8.0 Hz, 1H), 8.07 (ddd, ^3J = 7.8 Hz, ^4J = 1.1 Hz, ^5J = 0.5 Hz, 1H), 8.03 (s, 1H), 7.68 (td, ^3J = 7.8 Hz, ^4J = 1.4 Hz, 1H), 7.41 (td, ^3J = 7.6 Hz, ^4J = 1.1 Hz, 1H), 4.22 (s, 3H).[1] ^{13}C-NMR (125 MHz, DMSO-d_6, 300 K): δ_C (ppm) = 165.8, 165.5, 164.2, 133.7, 133.1, 127.6, 126.6, 125.8, 122.8, 103.5, 64.4. HR-MS: calcd for $C_{11}H_8N_1O_3$ [M-H]$^-$: 202.0510; found: 202.0510. Purity was determined by qNMR using maleic acid as an internal standard: 94.6%.

(Z)-1-[3-({[(1,3-Dioxo-2,3-dihydroisoquinolin-4(1H)-lidene)methyl] amino}methyl) phenyl]-N,N-dimethylmethanaminiumchloride TR-07*HCl

To a solution of *N*-[3-(aminomethyl)benzyl]-*N*,*N*-dimethylaminedihydrochloride (700 mg, 2.95 mmol, 1.20 equiv) in MeOH (7.0 mL), Et_3N (1.02 mL, 3.00 equiv) was added dropwise, stirred for 10 minutes, and then **TR-02** (500 mg, 2.46 mmol, 1.0 eq.) was added and stirred for 1 hour under microwave conditions (20 mL vessel, 110 °C, 300 W). Three separate batches were combined and the solvent was removed under reduced pressure. The remaining residue was resuspended in EtOAc (50.0 mL), treated with 4 *M* HCl in dioxane, and stirred overnight at room temperature to obtain the corresponding hydrochloride. The solvent was removed under reduced pressure and the crude product (max. 400 mg) was dissolved in 8.0 mL of 5% MeCN in H_2O and purified by preparative HPLC through several runs, yielding altogether 1.629 g (4.38 mmol, 59%) of product **TR-07*HCl** as a white to pale beige solid. mp 256.3 °C. ^{1}H-NMR (500 MHz, DMSO-d_6, 300 K): δ_H (ppm) = 10.97 (br s, 2H), 10.66 (dt, ^3J = 13.0 Hz, ^3J = 6.4 Hz, 1H), 8.67 (d, ^3J = 13.2 Hz, 1H), 7.98 (dd, ^3J = 8.0 Hz, ^4J = 1.4 Hz, 1H), 7.86 (d, ^3J = 8.3 Hz, 1H), 7.59-7.56 (m, 2H), 7.55 (td, ^3J = 8.2 Hz, 7.7 Hz, ^4J = 1.4 Hz, 1H), 7.48 (t, ^3J = 7.7 Hz, 1H), 7.47 (dd, ^4J = 2.2 Hz, 0.9 Hz, 1H), 7.17 (td, ^3J = 7.6 Hz, ^4J = 0.9 Hz, 1H), 4.76 (d, ^3J = 6.3 Hz, 2H), 4.27 (s, 2H), 2.67 (s, 3H). ^{13}C-NMR (125 MHz, DMSO-d_6, 300 K): δ_C (ppm) = 166.1, 163.9, 154.1, 139.0, 137.6, 132.9, 130.9, 130.2, 130.0, 129.2, 128.5, 127.5, 123.0, 120.2, 118.7, 92.3, 59.2, 52.0, 41.4 (2C). HR-MS: calcd for $C_{20}H_{22}N_3O_2$ [M]$^+$: 336.1707; found: 336.1700. Elemental analysis calcd (%) for $C_{20}H_{22}Cl_1N_3O_2$ x 0.75 H_2O: C: 62.33, H: 6.15, N: 10.90; found: C: 62.31, H: 6.36, N: 10.89. Purity was determined by qNMR using maleic acid as an internal standard: 100.5%.

Structure-activity-relationship (SAR) analyses of TR-07.

Synthesis of the benzylic amine precursors and aniline derivatives

All derivatives were synthesized from the central intermediate **TR-02**, which was reacted with the corresponding primary amine, following the synthetic sequences already described for the generation of **TR-07**. Amines that were not commercially available were synthesized in-house. In brief, the appropriate bromoalkylbenzonitrile derivatives were utilized for the synthesis of the corresponding substituted benzylamines, while the respective nitro derivatives were used for the generation of the aniline derivatives, as shown in Error! Reference source not found.. The primary amines were obtained by nucleophilic substitution under microwave irradiation conditions and subsequent reduction of either the nitrile functionality with $LiAlH_4$ or the aromatic nitro group with elemental iron.

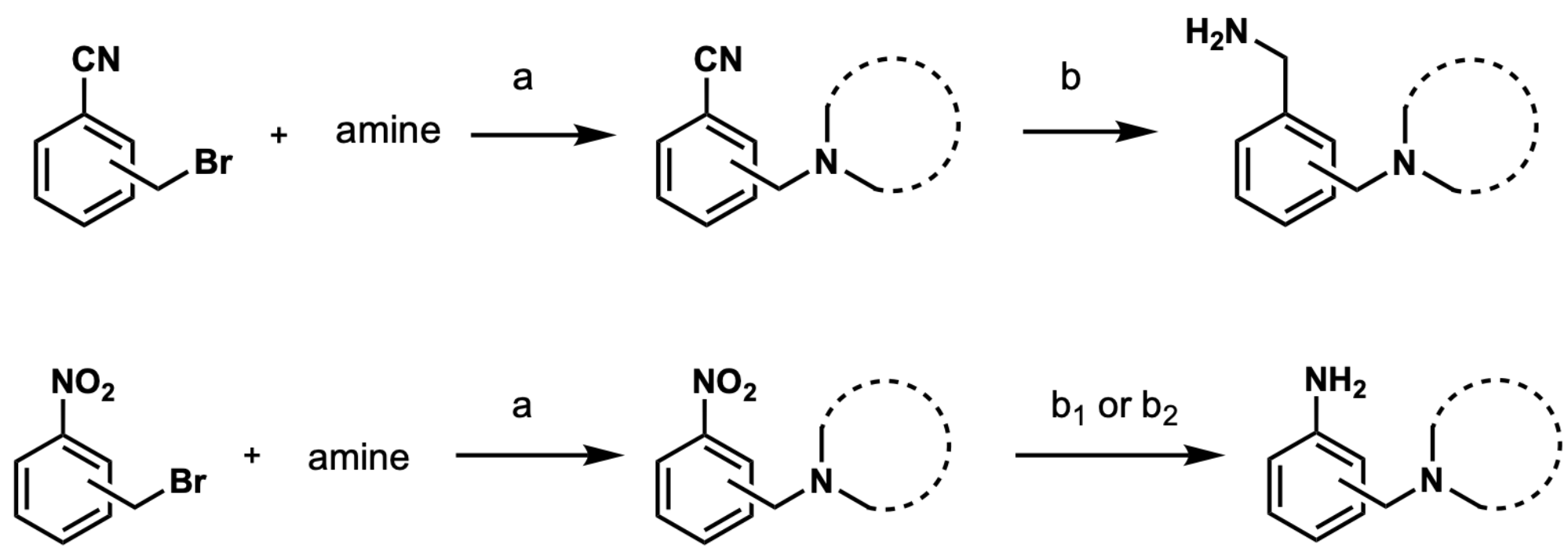

Reagents and conditions: a) K_2CO_3, MeCN, @MW: 120 °C, 150 W, 1 h; b) $LiAlH_4$, $THF_{(dry)}$, 0 °C, 3 h; b1) Fe, NH_4Cl, $HCl_{(conc.)}$, H_2O/MeOH (1:1), 50 °C, 4 h or b2) Fe, NH_4Cl, AcOH, 60 °C, 4 h.

Series I: Reduction of molecular complexity

(*Z*)-4-[(Benzylamino)methylene]isoquinoline-1,3(2*H*,4*H*)-dione TR-03

In a microwave vessel (20 mL), **TR-02** (2.00 g, 9.84 mmol, 1.00 equiv) and benzylamine (1.62 mL, 14.78 mmol, 1.50 equiv) were suspended in MeOH (9.84 mL) and stirred in the microwave reactor (110 °C, 300 W) for 1 h. After cooling to room temperature, the precipitate was filtered, washed with MeOH (15 mL) and Et_2O (10 mL), and finally dried under high vacuum at 50 °C to yield **TR-03** (2.60 g, 9.34 mmol) as a yellow solid with a yield of 95%. mp 183.8 °C. ^{1}H-NMR (500 MHz, DMSO-d_6, 300 K): δ_H (ppm) = 10.96 (s, 1H), 10.63 (dt, ^3J = 12.9 Hz, 6.3 Hz, 1H), 8.64 (d, ^3J = 13.2 Hz, 1H), 7.98 (dd, ^3J = 7.9 Hz, ^4J = 1.6 Hz, 1H), 7.83 (d, ^3J = 8.3 Hz, 1H), 7.55 (ddd, ^3J = 8.3 Hz, 6.9 Hz, ^4J = 1.4 Hz, 1H), 7.39-7.38 (m, 4H), 7.33-7.30 (m, 1H), 7.17 (td, ^3J = 7.5 Hz, ^4J = 1.2 Hz, 1H), 4.71 (d, ^3J = 6.3 Hz, 2H). ^{13}C-NMR (125 MHz, DMSO-d_6, 300 K): δ_C (ppm) = 166.1, 163.9, 154.0, 138.3, 137.7, 132.9, 128.7 (2C), 127.6 (2C), 127.5, 127.5, 122.9, 120.2, 118.6, 92.2, 52.2. HR-MS: calcd for $C_{17}H_{15}N_2O_2$

$[M+H]^+$ 279.1128; found: 279.1123. Elemental analysis calcd (%) for $C_{17}H_{14}N_2O_2$: C: 73.37, H: 5.07, N: 10.07; found: C: 73.37, H: 5.19, N: 10.04.

(*Z*)-4-[(Phenylamino)methylene]isoquinoline-1,3(2*H*,4*H*)-dione TR-014

In a microwave vessel (20 mL), **TR-02** (500 mg, 2.46 mmol, 1.00 equiv) and aniline (337 µL, 3.69 mmol, 1.50 equiv) were suspended in MeOH (5.00 mL) and stirred in the microwave reactor (125 °C, 300 W) for 30 minutes. After cooling to room temperature, the precipitate was filtered, washed with MeOH (15 mL) and Et_2O (10 mL), and dried under high vacuum at 50 °C to give **TR-014** (364 mg, 1.38 mmol) as a yellow solid with a yield of 56%. mp 241.6 °C. ^{1}H-NMR (500 MHz, DMSO-*d*$_6$, 300 K): δ_H (ppm) = 12.40 (d, ^3J = 12.6 Hz, 1H), 11.30 (s, 1H), 8.89 (d, ^3J = 12.6 Hz, 1H), 8.16 (d, ^3J = 8.3 Hz, 1H), 8.03 (dd, ^3J = 7.7 Hz, ^4J = 1.2 Hz, 1H), 7.62 (ddd, ^3J = 8.3 Hz, 6.9 Hz, ^4J = 1.4 Hz, 1H), 7.56 (dd, ^3J = 8.6 Hz, ^4J = 1.2 Hz, 2H), 7.43 (dd, ^3J = 8.6 Hz, 7.5 Hz, 2H), 7.28 (td, ^3J = 7.5 Hz, ^4J = 1.1 Hz, 1H), 7.17 (td, ^3J = 7.3 Hz, ^4J = 1.2 Hz, 1H). ^{13}C-NMR (100 MHz, DMSO-*d*$_6$, 300 K): δ_C (ppm) = 166.8, 163.8, 144.7, 139.2, 136.5, 133.1, 129.6 (2C), 127.5, 124.5, 124.3, 121.0, 120.0, 117.5 (2C), 95.6. HR-MS: calcd for $C_{16}H_{13}N_2O_2$ $[M+H]^+$ 265.0972; found: 265.0973. Elemental analysis calcd (%) for $C_{16}H_{12}N_2O_2$ x 0.25 H_2O: C: 71.50, H: 4.69, N: 10.42; found: C: 71.28, H: 4.57, N: 10.35. Purity was determined by qNMR using maleic acid as an internal standard: 95.1%.

(*Z*)-4-({[3-(Dimethylamino)propyl]amino}methylene)isoquinoline-1,3(2*H*,4*H*)-dione TR-018

In a microwave vessel (20 mL), **TR-02** (500 mg, 2.46 mmol, 1.00 equiv) and *N*,*N*-dimethylpropane-1,3-diamine (341 µL, 2.71 mmol, 1.10 equiv) were suspended in MeOH (5.00 mL) and stirred in the microwave reactor (125 °C, 300 W) for 30 minutes. After cooling to room temperature, the precipitate was filtered, washed with MeOH (15 mL) and Et_2O (10 mL), and dried under high vacuum at 50 °C to obtain **TR-018** (453 mg, 1.66 mmol) as a beige solid with a yield of 67%. mp 186.7 °C. ^{1}H-NMR (500 MHz, DMSO-*d*$_6$, 300 K): δ_H (ppm) = 10.89 (s, 1H), 10.52 (dt, ^3J = 13.3 Hz, 6.5 Hz, 1H), 8.45 (d, ^3J = 13.5 Hz, 1H), 7.97 (dd, ^3J = 7.7 Hz, ^4J = 1.4 Hz, 1H), 7.78 (d, ^3J = 8.3 Hz, 1H), 7.52 (ddd, ^3J = 8.4 Hz, 7.0 Hz, ^4J = 1.4 Hz, 1H), 7.14 (td, ^3J = 7.6 Hz, ^4J = 1.2 Hz, 1H), 3.52 (q, ^3J = 6.6 Hz, 2H), 2.27 (t, ^3J = 6.7 Hz, 2H), 2.14 (s, 6H), 1,73 (t, ^3J = 6.7 Hz, 2H). ^{13}C-NMR (125 MHz, DMSO-*d*$_6$, 300 K): δ_C (ppm) = 165.9, 163.9, 154.1, 137.9, 132.8, 127.5, 122.6, 120.0, 118.4, 91.5, 56.2, 47.5, 45.1 (2C), 27.9. HR-MS: calcd for $C_{15}H_{20}N_3O_2$ $[M+H]^+$ 274.1550; found: 274.1553. Elemental analysis calcd (%) for $C_{15}H_{19}N_3O_2$: C: 65.91, H: 7.01, N: 15.37; found: C: 65.84, H: 6.83, N: 15.36.

(*Z*)-4-[(Pyridin-2-ylamino)methylene]isoquinoline-1,3(2*H*,4*H*)-dione TR-030

In a microwave vessel (20 mL), **TR-02** (406 mg, 2.00 mmol, 1.00 equiv) and 2-aminopyridine (207 mg, 2.20 mmol, 1.10 equiv) were suspended in MeOH (4.00 mL) and stirred in the microwave reactor (125 °C, 300 W) for 30 minutes. After cooling to room temperature, the precipitate was filtered, washed with MeOH (15 mL) and Et_2O (10 mL), and dried under high vacuum at 50 °C to afford product **TR-030** (493 mg, 1.86 mmol) as a yellow solid with a yield of 93%. mp 291.5 °C. ^{1}H-NMR (500 MHz, DMSO-*d*$_6$, 300 K): δ_H (ppm) = 12.22 (d, ^3J = 12.3

Hz, 1H), 11.38 (s, 1H), 9.17 (d, ^3J = 12.3 Hz, 1H), 8.39 (dd, ^3J = 4.9 Hz, ^4J = 1.7 Hz, 1H), 8.04 (dd, ^3J = 8.0 Hz, ^4J = 1.4 Hz, 1H), 7.97 (d, ^3J = 8.0 Hz, 1H), 7.84 (ddd, ^3J = 8.2 Hz, 7.3 Hz, ^4J = 1.7 Hz, 1H), 7.65 (ddd, ^3J = 8.4 Hz, 7.1 Hz, ^4J = 1.4 Hz, 1H), 7.53 (d, ^3J = 8.3 Hz, 1H), 7.32 (td, ^3J = 7.6 Hz, ^4J = 1.2 Hz, 1H), 7.16 (ddd, ^3J = 7.3 Hz, 4.9 Hz, ^4J = 0.9 Hz, 1H) ppm. ^{13}C-NMR (125 MHz, DMSO-d_6, 300 K): δ_C (ppm) = 166.8, 163.8, 150.9, 148.4, 141.5, 139.0, 136.1, 133.5, 127.7, 124.9, 121.5, 119.8, 119.7, 112.1, 97.1. HR-MS: calcd for $C_{15}H_{12}N_3O_2$ [M+H]$^+$ 266.0924; found: 266.0927. Elemental analysis calcd (%) for $C_{15}H_{11}N_3O_2$: C: 67.92, H: 4.18, N: 15.84; found: C: 67.56, H: 4.25, N: 15.73.

(*Z*)-4-[(Pyridin-3-ylamino)methylene]isoquinoline-1,3(2*H*,4*H*)-dione TR-031

In a microwave vessel (20 mL), **TR-02** (406 mg, 2.00 mmol, 1.00 equiv) and 3-aminopyridine (207 mg, 2.20 mmol, 1.10 equiv) were suspended in MeOH (4.00 mL) and stirred in the microwave reactor (125 °C, 300 W) for 30 minutes. After cooling to room temperature, the precipitate was filtered, washed with MeOH (15 mL) and Et_2O (10 mL), and dried under high vacuum at 50 °C to provide **TR-031** (510 mg, 1.92 mmol) as a yellow solid with a yield of 96%. mp 253.4 °C. ^{1}H-NMR (500 MHz, DMSO-d_6, 300 K): δ_H (ppm) = 12.29 (d, ^3J = 12.3 Hz, 1H), 11.35 (s, 1H), 8.89 (d, ^3J = 12.3 Hz, 1H), 8.79 (d, ^4J = 2.6 Hz, 1H), 8.36 (dd, ^3J = 4.6 Hz, ^4J = 1.4 Hz, 1H), 8.17 (d, ^3J = 8.3 Hz, 1H), 8.06 (ddd, ^3J = 8.3 Hz, ^4J = 2.9 Hz, 1.4 Hz, 1H), 8.03 (dd, ^3J = 8.0 Hz, ^4J = 1.2 Hz, 1H), 7.63 (ddd, ^3J = 8.3 Hz, 7.0 Hz, ^4J = 1.4 Hz, 1H), 7.45 (dd, ^3J = 8.3 Hz, ^4J = 4.6 Hz, 1H), 7.30 (td, ^3J = 7.6 Hz, ^4J = 1.2 Hz, 1H). ^{13}C-NMR (100 MHz, DMSO-d_6, 300 K): δ_C (ppm) = 166.9, 163.8, 145.2, 144.8, 140.1, 136.3, 136.1, 133.2, 127.5, 124.6, 124.5, 124.0, 121.2, 120.3, 96.7. HR-MS: calcd for $C_{15}H_{11}N_3O_2Na_1$ [M+H]$^+$ 288.0743; found: 288.0752. Elemental analysis calcd (%) for $C_{15}H_{11}N_3O_2$: C: 67.92, H: 4.18, N: 15.84; found: C: 67.52, H: 4.25, N: 15.80.

(*Z*)-4-{[(Pyridin-3-ylmethyl)amino]methylene}isoquinoline-1,3(2*H*,4*H*)-dione TR-032

In a microwave vessel (20 mL), **TR-02** (406 mg, 2.00 mmol, 1.00 equiv) and pyridin-3-ylmethanamine (222 µL, 2.20 mmol, 1.10 equiv) were suspended in MeOH (4.00 mL) and stirred in the microwave reactor (125 °C, 300 W) for 30 minutes. After cooling to room temperature, the precipitate was filtered, washed with MeOH (15 mL) and Et_2O (10 mL), and dried under high vacuum at 50 °C to give **TR-032** (520 mg, 1.86 mmol) as a beige solid with a yield of 93%. mp 231.4 °C. ^{1}H-NMR (500 MHz, DMSO-d_6, 300 K): δ_H (ppm) = 10.96 (s, 1H), 10.63 (dt, ^3J = 13.2 Hz, 6.3 Hz, 1H), 8.65 (d, ^3J = 13.2 Hz, 1H), 8.63 (d, ^4J = 2.3 Hz, 1H), 8.52 (dd, ^3J = 4.7 Hz, ^4J = 1.4 Hz, 1H), 7.98 (d, ^3J = 7.9 Hz, ^4J = 1.6 Hz, 1H), 7.84 (d, ^3J = 8.0 Hz, 1H), 7.82 (ddd, ^3J = 7.9 Hz, ^4J = 2.3 Hz, 1.4 Hz, 1H), 7.55 (ddd, ^3J = 8.3 Hz, 6.9 Hz, ^4J = 1.4 Hz, 1H), 7.41 (ddd, ^3J = 7.8 Hz, 4.8 Hz, ^5J = 0.9 Hz, 1H), 7.17 (ddd, ^3J = 7.9 Hz, 7.0 Hz, ^4J = 1.2 Hz, 1H), 4.74 (d, ^3J = 6.3 Hz, 2H). ^{13}C-NMR (125 MHz, DMSO-d_6, 300 K): δ_C (ppm) = 166.0, 163.9, 153.9, 149.0, 148.8, 137.6, 135.4, 134.1, 132.9, 127.5, 123.7, 123.0, 120.3, 118.7, 92.5, 49.8. HR-MS: calcd for $C_{16}H_{13}N_3O_2Na_1$ [M+Na]$^+$ 302.0900; found: 302.0910. Elemental analysis calcd (%) for $C_{16}H_{13}N_3O_2$: C: 68.81, H: 4.69, N: 15.04; found: C: 68.50, H: 4.67, N: 15.02.

Series II: Modifications in the Southern Region - benzylamine derivatives

Synthesis of ICTR-108

The synthesis of the compounds in this series was analogous to the previously used synthesis sequence. For the synthesis of the alkylated derivative **ICTR-108**, the amine precursor was prepared according to a published procedure.[3] In brief, 1,3-phenylenedimethanamine was at first mono Boc-protected and subsequently methylated utilizing methyl iodide resulting in **ICTR-104**. Deprotection of **ICTR-104** with aqueous hydrogen iodide gave **ICTR105**, which was then reacted with precursor **TR-02** under microwave irradiation conditions to afford **ICTR-108** in good overall yield.

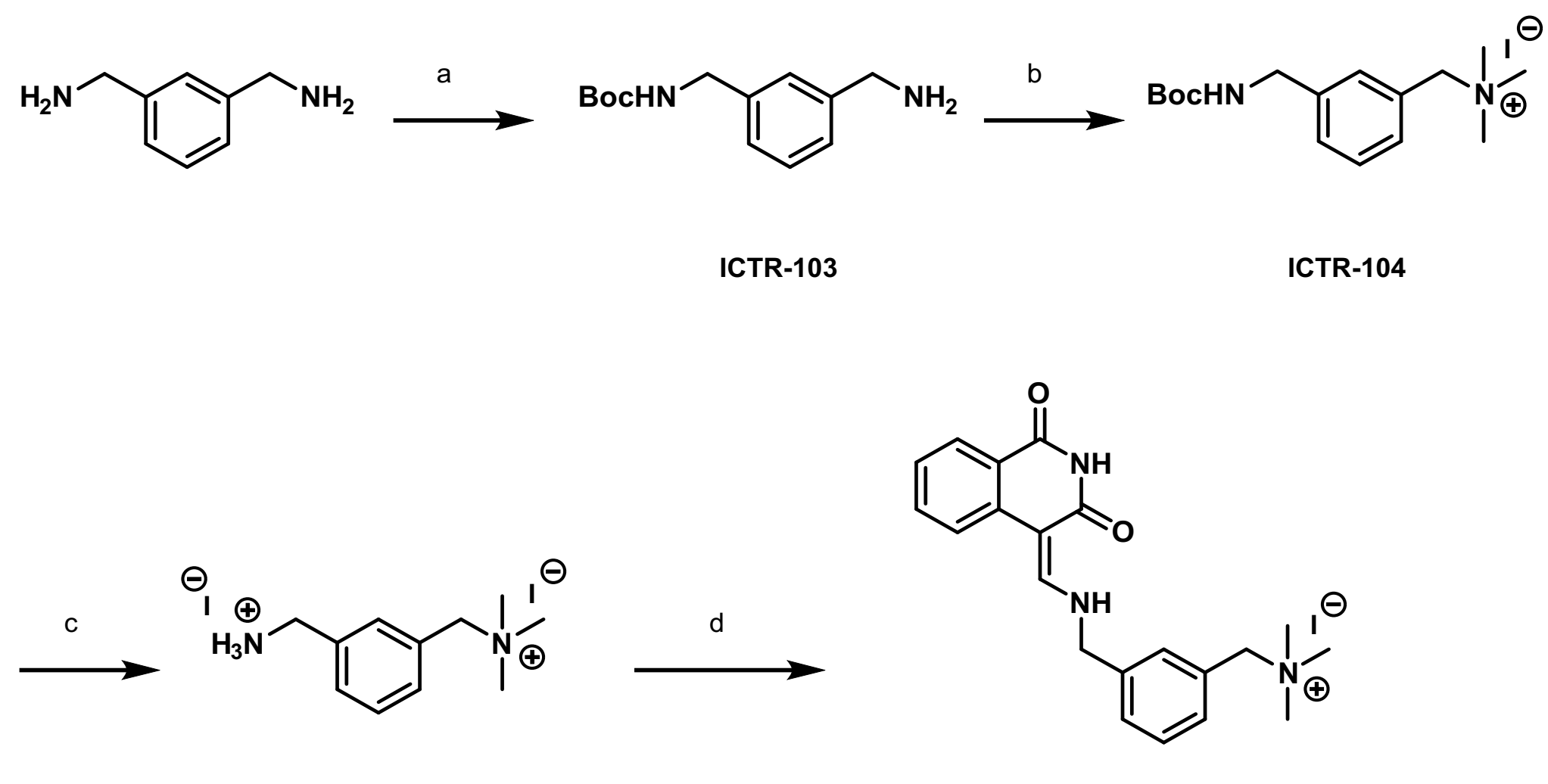

a Reagents and conditions: a) Boc-Anhydride, Et_3N, DCM, 0 °C – rt, o.n., 56%; b) MeI, $NaHCO_3$, MeOH, rt, 2 d; c) HI (aq., 57%), H_2O, rt, o.n., 29% (o2s); d) Et_3N, MeOH, @MW: 125 °C, 30 min, 83%.

tert-Butyl [3-(aminomethyl)benzyl]carbamate ICTR-103

A 0.5 M solution of Boc_2O (1091 mg, 5.00 mmol, 1.00 equiv) in $CDCl_3$ (10.0 mL) was added dropwise over a period of 2 hours to a 0.25 M solution of 1,3-phenylenedimethylamine (3405 mg, 25.0 mmol, 5.00 equiv) in $CDCl_3$ (100 mL) at 0 °C. The ice bath was removed and the mixture was stirred overnight at room temperature. After the reaction was complete (according to TLC), the mixture was filtered and the filtrate was concentrated under reduced pressure. The oily residue was dissolved in EtOAc, washed with brine (3x), dried over MgSO4, filtered, and the solvent was removed under reduced pressure. Purification by MPLC (0-10% MeOH in DCM over 20 min) yielded **ICTR-103** (649 mg, 2.79 mmol) as a colorless to pale yellow oil with a yield of 56%.[5] ^{1}H-NMR (300 MHz, DMSO-d_6, 300 K): δ_H = 7.36–7.19 (m, 4H), 4.90 (br s, 1H), 4.34 (d, ^3J = 5,5 Hz, 2H), 3.89 (s, 2H), 1.63 (br s, 2H), 1.50 (s, 9H). HR-MS: calcd for $C_{13}H_{20}N_2O_2Na$ [M+Na]$^+$ 259.1417; found: 259.1416.

1-(3-{[(*tert*-Butoxycarbonyl)amino]methyl}phenyl)-*N*,*N*,*N*-trimethylmethanaminiumiodide ICTR-104

To a solution of **ICTR-103** (500 mg, 2.12 mmol, 1.00 equiv) and $NaHCO_3$ (1781 mg, 21.2 mmol, 10.0 equiv) in MeOH (14 mL), MeI (1.32 mL, 21.2 mmol, 10.0 equiv) was added, and the mixture was stirred for 2 days at room temperature. After the reaction was complete, the mixture was filtered, and the solvent was removed under reduced pressure to obtain product **ICTR-104** as a white solid.[4] The crude product was used in the next reaction step without any further purification. ^{1}H-NMR (300 MHz, CD_3OD, 300 K): δ_H (ppm) = 7.48 (m, 4H), 4.57 (s, 2H), 4.23 (s, 2H), 3.13 (s, 9H), 1.45 (s, 9H). HR-MS: calcd for $C_{16}H_{27}N_2O_2$ [M+H]$^+$ 279.2067; found: 279.2066.

1-[3-(Ammoniomethyl)phenyl]-*N*,*N*,*N*-trimethylmethanaminiumdiiodide ICTR-105

To a solution of crude **ICTR-104** (1.0 g, 2.46 mmol, 1.00 equiv) in H_2O (21.0 mL), 57% HI (aq., 1.62 mL, 5.00 equiv) was added, and the mixture was stirred overnight at room temperature. After the reaction was complete, the solution was washed with DCM (4 x 10.0 mL), the organic layer was discarded and the H_2O was removed under reduced pressure. The remaining residue was dissolved in MeOH (18.0 mL) and a precipitate formed upon the addition of EtOAc (36.0 mL). The precipitate was filtered, washed with EtOAc and MeOH, and dried in a high vacuum at 50 °C to yield **ICTR-105** (302 mg, 0.70 mmol) as a pale yellow solid with a yield of 29% over two steps. ^{1}H-NMR (300 MHz, CD_3OD, 300 K): δ_H (ppm) = 7.77 (s, 1H), 7.71-7.61 (m, 3H), 4.67 (s, 2H), 4.25 (s, 2H), 3.18 (s, 9H). HR-MS: calcd for $C_{11}H_{19}N_2$ [M+H]$^+$ 179.1543; found: 179.1541.

(*Z*)-1-[3-({[(1,3-Dioxo-2,3-dihydroisoquinoline-4(1*H*)-ylidene)methyl]amino} methyl)phenyl]-*N*,*N*,*N*-trimethylmethanaminiumiodide ICTR-108

In a microwave vessel (15 mL), **TR-02** (91 mg, 0.45 mmol, 1.00 equiv), **ICTR-105** (250 mg, 0.58 mmol, 1.30 equiv), and Et_3N (80 µL, 1.30 equiv) were suspended in MeOH (3.00 mL) and stirred in a microwave reactor (125 °C, 300 W) for 30 minutes. After cooling to room temperature, the precipitate was filtered, washed with MeOH (5.00 mL), and dried in a high vacuum to obtain **ICTR-108** (179 mg, 0.38 mmol) as a pale yellow solid with a yield of 83%. mp 222.4 °C. ^{1}H-NMR (500 MHz, DMSO-d_6, 300 K): δ_H = 10.97 (s, 1H), 10.66 (dt, ^3J = 13.0 Hz, 6.4 Hz, 1H), 8.68 (d, ^3J = 13.2 Hz, 1H), 7.98 (dd, ^3J = 8.0 Hz, ^4J = 1.4 Hz, 1H), 7.85 (d, ^3J = 8.0 Hz, 1H), 7.60-7.54 (m, 3H), 7.53 (br s, 1H), 7.49 (d, ^3J = 7.3 Hz, 1H), 7.18 (t, ^3J = 7.5 Hz, 1H), 4.80 (d, ^3J = 6.3 Hz, 2H), 4.55 (s, 2H), 3.04 (s, 9H). ^{13}C-NMR (125 MHz, DMSO-d_6, 300 K): δ_C (ppm) = 166.1, 163.8, 154.0, 139.2, 137.6, 132.9, 132.0, 131.7, 129.4, 129.3, 128.7, 127.5, 123.0, 120.2, 118.6, 92.3, 67.6, 51.9, 51.8 (3C). HR-MS: calcd for $C_{21}H_{24}N_3O_2$ [M]$^+$ 350.1863; found: 350.1861. Elemental analysis calcd (%) for $C_{21}H_{24}I_1N_3O_2$ x 1.0 H_2O: C: 50.92, H: 5.29, N: 8.48; found: C: 50.95, H: 5.42, N: 8.50.

(*Z*)-4-({[2-(Dimethylamino)benzyl]amino}methylene)isoquinolin-1,3(2*H*,4*H*)-dione ICTR-052

In a microwave vessel (20 mL), **TR-02** (313 mg, 1.54 mmol, 1.00 equiv) and 2-(aminomethyl)-*N*,*N*-dimethylaniline (0.29 mL, 2.00 mmol, 1.30 equiv) were suspended in MeOH (5.00 mL) and stirred in the

microwave reactor (125 °C, 300 W) for 30 minutes. After cooling to room temperature, the precipitate formed was filtered, washed with MeOH (10 mL) and Et_2O (10 mL), and finally dried under high vacuum at 50 °C to afford **ICTR-052** (440 mg, 1.37 mmol) as a pale yellow solid with a yield of 89%. mp 200 °C. ^{1}H-NMR (500 MHz, DMSO-d_6, 300 K): δ_H (ppm) = 10.94 (br s, 1H), 10.84 (dt, ^3J = 13.0 Hz, 6.4 Hz, 1H), 8.63 (d, ^3J = 13.0 Hz, 1H), 7.98 (dd, ^3J = 8.0 Hz, ^4J = 1.4 Hz, 1H), 7.81 (d, ^3J = 8.0 Hz, 1H), 7.54 (ddd, ^3J = 8.5 Hz, 7.1 Hz, ^4J = 1.4 Hz, 1H), 7.32-7.26 (m, 2H), 7.23 (dd, ^3J = 8.0 Hz, ^4J = 1.1 Hz, 1H), 7.15 (td, ^3J = 7.4 Hz, ^4J = 1.0 Hz, 1H), 7.09 (td, ^3J = 7.3 Hz, 7.1 Hz, ^4J = 1.1 Hz, 1H), 4.78 (d, ^3J = 6.4 Hz, 2H), 2.67 (s, 6H). ^{13}C-NMR (125 MHz, DMSO-d_6, 300 K): δ_C (ppm) = 166.1, 163.9, 154.0, 152.3, 137.8, 132.9, 132.6, 128.6, 128.4, 127.5, 123.7, 122.8, 120.2, 120.1, 118.4, 91.9, 49.7, 44.7 (2C). HR-MS: calcd for $C_{19}H_{19}N_3O_2Na_1$ $[M+Na]^+$ 344.1369; found: 344.1379. Elemental analysis calcd (%) for $C_{19}H_{19}N_3O_2$: C: 71.01, H: 5.96, N: 13.07; found: C: 71.15, H: 5.86, N: 13.17.

Synthesis of ICTR-063

2-[(Dimethylamino)methyl]benzonitrile ICTR-046

ICTR-046 was synthesized using a slightly modified version of GWP 1 (120 °C, 150 W, 1 h) employing 2-(bromomethyl)benzonitrile (673 mg, 3.43 mmol, 1.00 equiv), dimethylamino hydrochloride (307 mg, 3.77 mmol, 1.10 equiv), Et_3N (0.71 mL, 1.50 equiv), and potassium carbonate (474 mg, 3.43 mmol, 1.00 equiv). After washing with brine (2 x 25 mL), drying over $MgSO_4$, filtering, and removing the solvent under reduced pressure, **ICTR-46** (313 mg, 1.95 mmol) was obtained as an orange-brown oil with a yield of 57%. ^{1}H-NMR (500 MHz, $CDCl_3$, 300 K): δ_H (ppm) = 7.64 (dd, ^3J = 8.0 Hz, ^4J = 1.2 Hz, 1H), 7.56 (ddd, ^3J = 7.7 Hz, 6.9 Hz, ^4J = 1.2 Hz, 2H), 7.55 (dd, ^3J = 8.6 Hz, ^4J = 2,0 Hz, 1H), 7.35 (ddd, ^3J = 8.3 Hz, 6.3 Hz, ^4J = 2.0 Hz, 1H), 3.63 (s, 2H), 2.29 (s, 6H). ^{13}C-NMR (125 MHz, $CDCl_3$, 300 K): δ_C (ppm) = 143.1, 132.9, 132.8, 130.2, 127.7, 118.0, 113.0, 61.9, 45.5 (2C). HR-MS: calcd for $C_{10}H_{13}N_2$ $[M+H]^+$ 161.1073; found: 161.1071. Purity was determined by qNMR using dimethyl terephthalate as an internal standard: 87.7%.

1-[2-(Aminomethyl)phenyl]-*N*,*N*-dimethylmethanamin ICTR-062

ICTR-062 was obtained according to GWP 5 (Reduction of nitrile group with $LiAlH_4$) using **ICTR-046** (200 mg, 1.25 mmol, 1.00 equiv) in dry THF (12.5 mL) and 2.4 M $LiAlH_4$ solution in THF (0.78 mL, 1.87 mmol, 1.50 equiv). After the reaction was complete, sodium sulfate decahydrate ($Na_2SO_4 \cdot$x 10 H_2O) (2.0 g, 6.25 mmol, 5.00 equiv) was added and the reaction mixture stirred for an additional 30 minutes at 0 °C. After removal of the ice bath, the reaction mixture was stirred for another hour at room temperature, then filtered over Celite and the solvent was removed under reduced pressure, yielding 205 mg (1.25 mmol, quant.) of **ICTR-062** as a yellow oil, which was immediately further processed without additional purification.

(*Z*)-4-[({2-[(Dimethylamino)methyl)]benzyl}amino)methylene]isoquinoline-1,3(2*H*,4*H*)-dione ICTR-063

In a microwave vessel (20 mL), **TR-02** (191 mg, 0.94 mmol, 1.00 equiv) and **ICTR-062** (200 mg, 1.22 mmol, 1.30 equiv) were suspended in MeOH (5.00 mL) and the mixture was stirred in a microwave reactor at 125 °C and 300 W for 30 minutes. After cooling to room temperature, the solvent was removed under reduced pressure and the crude product was adsorbed on silica gel. Purification was carried out via MPLC with a gradient of 0-5% MeOH in DCM over 30 minutes, yielding the slightly impure product (145 mg, 0.43 mmol) as a yellow solid. After recrystallization from MeOH, 84 mg (0.25 mmol, 27%) of **ICTR-063** were obtained. mp 220.6 °C. ^{1}H-NMR (500 MHz, DMSO-*d*$_6$, 300 K): δ_H (ppm) = 10.87 (dt, ^3J = 13.3 Hz, ^3J = 6.5 Hz, 1H), 10.83 (br s, 1H), 8.64 (d, ^3J = 13.5 Hz, 1H), 7.96 (dd, ^3J = 8.0 Hz, ^4J = 1.4 Hz, 1H), 7.86 (d, ^3J = 8.2 Hz, 1H), 7.54 (td, ^3J = 7.7 Hz, ^4J = 1.4 Hz, 1H), 7.50 (dd, ^3J = 7.3 Hz, ^4J = 1.1 Hz, 1H), 7.33 (ddd, ^3J = 7.3 Hz, 7.1 Hz, ^4J = 2.1 Hz, 1H), 7.28 (ddd, ^3J = 7.3 Hz, 7.1 Hz, ^4J = 1.4 Hz, 1H), 7.25 (dd, ^3J = 7.3 Hz, ^4J = 1.8 Hz, 1H), 7.14 (ddd, ^3J = 7.6 Hz, 7.4 Hz, ^4J = 1.0 Hz, 1H), 4.71 (d, ^3J = 6.4 Hz, 2H), 3.51 (s, 2H), 2.20 (s, 6H). ^{13}C-NMR (125 MHz, DMSO-*d*$_6$, 300 K): δ_C (ppm) = 165.6, 163.9, 152.9, 138.1, 137.5, 137.5, 132.8, 130.5, 129.5, 128.1, 127.7, 127.5, 122.5, 120.0, 118.4, 91.7, 62.1, 51.3, 44.6 (2C). HR-MS: calcd for $C_{20}H_{22}N_3O_2$ [M+H]$^+$ 336.1707; found: 336.1712. Elemental analysis calcd (%) for $C_{20}H_{21}N_3O_2$: C: 71.62, H: 6.31, N: 12.53; found: C: 71.39, H: 6.41, N: 12.43.

Synthesis of ICTR-119

2-[(Diethylamino)methyl]benzonitril ICTR-106

According to a slightly modified GWP 1 (120 °C, 150 W, 2 h), 2-(bromomethyl)benzonitrile (1.0 g, 5.12 mmol, 1.00 equiv), diethylamine (0.6 mL, 5.64 mmol, 1.10 equiv) and K_2CO_3 (708 mg, 5.12 mmol, 1.00 equiv) in MeCN (14 mL) were used. The reaction mixture was filtered over a Celite pad, the solvent was removed under reduced pressure, and the crude product was adsorbed on silica gel. After purification by MPLC (0-10% EtOAc in CH over 20 min), 794 mg (4.22 mmol, 82%) of **ICTR-106** were obtained as a yellow oil. ^{1}H-NMR (500 MHz, $CDCl_3$, 300 K): δ_H (ppm) = 7.65 (dd, ^3J = 7.7 Hz, ^4J = 1.2 Hz, 1H), 7.61 (dd, ^3J = 7.7 Hz, ^4J = 1.2 Hz, 1H), 7.55 (t, ^3J = 7.7 Hz, 1H), 7.32 (t, ^3J = 7.7 Hz, 1H), 3.77 (s, 2H), 2.58 (q, ^3J = 7.1 Hz, 4H), 1.06 (t, ^3J = 7.2 Hz, 6H). ^{13}C-NMR (125 MHz, $CDCl_3$): δ_C (ppm) = 144.8, 132.9, 132.7, 129.9, 127.2, 118.1, 112.5, 55.8, 47.2 (2C), 12.0 (2C). HR-MS: calcd for $C_{12}H_{17}N_2$ [M+H]$^+$ 189.1386; found: 189.1384. Purity was determined by qNMR using maleic acid as an internal standard: 92.6%.

N-[2-(Aminomethyl)benzyl]-*N*-ethylethanamine ICTR-117

ICTR-117 was prepared according to GWP 5 using **ICTR-106** in THF (anhydrous) and 2.4 M $LiAlH_4$ solution in THF. After the reaction was complete, sodium sulfate decahydrate was added and stirred for an additional 30 minutes at 0 °C. After removal of the ice bath, the reaction mixture was stirred for another hour at room temperature, then filtered over Celite, and the solvent was removed under reduced pressure to furnish 349 mg of **ICTR-117** as a yellow oil, which was used immediately without further purification.

(*Z*)-4-[({2-[(Diethylamino)methyl]benzyl}amino)methylene]isoquinoline-1,3(2*H*,4*H*)-dione ICTR-119

In a microwave vessel (20 mL), **TR-02** and crude **ICTR-117** were suspended in MeOH (5.00 mL) and stirred in the microwave reactor (125 °C, 300 W) for 30 minutes. After cooling to room temperature, the solvent was removed under reduced pressure, the crude product was adsorbed on silica gel and purified by MPLC (0-10% MeOH in DCM over 20 minutes), resulting in 183 mg of **ICTR-119** as a beige solid (0.50 mmol, 63% yield). mp 167.8 °C. ^{1}H-NMR (500 MHz, DMSO-*d*$_6$, 300 K): δ_H (ppm) = 10.86 (s, 1H), 10.68 (dt, ^3J = 13.0 Hz, 6.4 Hz, 1H), 8.64 (d, ^3J = 13.5 Hz, 1H), 7.96 (dd, ^3J = 8.0 Hz, ^4J = 1.4 Hz, 1H), 7.85 (d, ^3J = 8.0 Hz, 1H), 7.55 (td, ^3J = 7.6 Hz, ^4J = 1.4 Hz, 1H), 7.47 (d, ^3J = 7.2 Hz, 1H), 7.32-7.25 (m, 3H), 7.15 (dd, ^3J = 7.6 Hz, 7.3 Hz, 1H), 4.74 (d, ^3J = 6.3 Hz, 2H), 3.64 (s, 2H), 2.52 (q, ^3J = 7.2 Hz, 4H), 1.01 (t, ^3J = 7.2 Hz, 6H). ^{13}C-NMR (125 MHz, DMSO-*d*$_6$, 300 K): δ_C (ppm) = 165.7, 163.9, 153.2, 137.9, 137.8, 137.2, 132.8, 130.4, 129.2, 127.7 (2C), 127.5, 122.7, 120.1, 118.5, 91.8, 55.2, 50.8, 45.8 (2C), 10.4 (2C). HR-MS: calcd for $C_{22}H_{25}N_3O_2Na$ [M+Na]$^+$ 386.1839; found: 386.1835. Elemental analysis calcd (%) for $C_{22}H_{25}N_3O_2$: C: 72.70, H: 6.93, N: 11.56; found: C: 72.44, H: 6.71, N: 11.48.

Synthesis of ICTR-120

3-[(Diethylamino)methyl]benzonitrile ICTR-107

Following a slightly modified GWP 1 (120 °C, 150 W, 2 h), 3-(bromomethyl)benzonitrile (1.0 g, 5.12 mmol, 1.00 equiv), diethylamine (0.6 mL, 5.64 mmol, 1.10 equiv), and K_2CO_3 (708 mg, 5.12 mmol, 1.00 equiv) were employed in MeCN (14 mL). The reaction mixture was filtered over a Celite pad, the solvent was removed under reduced pressure, and the crude product was adsorbed on silica gel. After purification by MPLC (0-10% EtOAc in CH over 20 min), 450 mg (2.39 mmol, 47%) of **ICTR-107** were obtained as a pale yellow oil. ^{1}H-NMR (500 MHz, $CDCl_3$, 300 K): δ_H (ppm) = 7.66 (dd, ^4J = 1.9 Hz, 1.4 Hz, 1H), 7,57 (ddd, ^3J = 7.7 Hz, ^4J = 2.0 Hz, 1.4 Hz, 1H), 7,52 (dt, ^3J = 7.7 Hz, ^4J = 1.4 Hz, 1H), 7.40 (t, ^3J = 7.7 Hz, 1H), 3.57 (s, 2H), 2.51 (q, ^3J = 7.2 Hz, 4H), 1.03 (t, ^3J = 7.2 Hz, 6H). ^{13}C-NMR (125 MHz, $CDCl_3$): δ_C (ppm) = 142.1, 133.2, 132.3, 130.6, 129.0, 119.3, 112.3, 57.0, 47.0 (2C), 11.9 (2C). HR-MS: calcd for $C_{12}H_{17}N_2$ [M+H]$^+$ 189.1386; found: 189.1383. Purity was determined by qNMR using dimethyl terephthalate as an internal standard: 86.9%.

N-[3-(Aminomethyl)benzyl]-*N*-ethylethanamine ICTR-118

According to GWP 5, **ICTR-107** (350 mg, 1.86 mmol, 1.00 equiv) was used in anhydrous THF (18.6 mL) and 2.4 M $LiAlH_4$ solution in THF (1.2 mL, 2.88 mmol, 1.50 equiv). After the reaction was complete, sodium sulfate decahydrate (3.0 g, 9.30 mmol, 5.00 equiv) was added and stirred for an additional 30 minutes at 0 °C. After removal of the ice bath, the reaction mixture was stirred for another hour at room temperature, then filtered over Celite, and the solvent was removed under reduced pressure to give 330 mg (1.72 mmol, 92%) of **ICTR-118** as a pale yellow oil, which was used immediately without further purification.

(*Z*)-4-[({3-[(Diethylamino)methyl]benzyl}amino)methylene]isoquinoline-1,3(2*H*,4*H*)-dione ICTR-120

In a microwave vessel (20 mL), **TR-02** (163 mg, 0.80 mmol, 1.00 equiv) and crude **ICTR-118** (200 mg, 1.04 mmol, 1.30 equiv) were suspended in MeOH (7.00 mL) and stirred in a microwave reactor (125 °C, 300 W) for 30 minutes. After cooling to room temperature, the solvent was removed under reduced pressure, the crude product was adsorbed on silica gel and purified by MPLC (0-10% MeOH in DCM over 20 min), yielding 219 mg (0.60 mmol, 75%) of **ICTR-120** as a brownish solid. mp 146.8 °C. ^{1}H-NMR (500 MHz, DMSO-*d*$_6$, 300 K): δ_H (ppm) = 10.95 (s, 1H), 10.66 (dt, ^{3}J = 13.2 Hz, 6.6 Hz, 1H), 8.64 (d, ^{3}J = 13.2 Hz, 1H), 7.98 (dd, ^{3}J = 8.0 Hz, ^{4}J = 1.4 Hz, 1H), 7.82 (d, ^{3}J = 8.0 Hz, 1H), 7.55 (ddd, ^{3}J = 8.3 Hz, 6.9 Hz, ^{4}J = 1.4 Hz, 1H), 7.34 (s, 1H), 7.32 (t, ^{3}J = 7.5 Hz, 1H), 7.25 (d, ^{3}J = 7.2 Hz, 2H), 7.17 (t, ^{3}J = 7.5 Hz, 1H), 4.71 (d, ^{3}J = 6.5 Hz, 2H), 3.53 (s, 2H), 2.44 (q, ^{3}J = 6.9 Hz, 4H), 0.95 (t, ^{3}J = 7.2 Hz, 6H). ^{13}C-NMR (125 MHz, DMSO-*d*$_6$, 300 K): δ_C (ppm) = 166.1, 163.8, 153.9, 138.1, 137.7, 137.7, 132.9, 128.4, 127.7, 127.5, 125.8, 122.9, 120.2, 118.5, 92.1, 56.8, 52.2, 46.1(2C), 11.5(2C). HR-MS: calcd for $C_{22}H_{26}N_3O_2$ [M+H]$^+$ 364.2020; found: 364.2014. Elemental analysis calcd (%) for $C_{22}H_{25}N_3O_2$ x 0.5 H_2O: C: 70.94, H: 7.04, N: 11.28; found: C: 70.80, H: 6.82, N: 11.15.

(*Z*)-4-[({4-[(Dimethylamino)methyl]benzyl}amino)methylene]isoquinoline-1,3(2*H*,4*H*)-dione ICTR-078

In a microwave vessel (20 mL), **TR-02** (242 mg, 1.19 mmol, 1.00 equiv), 1-[4-(aminomethyl)phenyl]-*N*,*N*-dimethylmethanaminiumchloride (311 mg, 1.55 mmol, 1.30 equiv), and Et_3N (0.22 mL, 1.55 mmol, 1.30 equiv) were suspended in MeOH (5.00 mL) and stirred in a microwave reactor (125 °C, 300 W) for 30 minutes. After cooling to room temperature, the precipitate was filtered, washed with MeOH (10 mL) and Et_2O (10 mL), and dried in a high vacuum at 50 °C to obtain **ICTR-078** (287 mg, 0.86 mmol) as a beige to yellow solid with a yield of 72%. mp 194.3 °C. ^{1}H-NMR (500 MHz, DMSO-*d*$_6$, 300 K): δ_H (ppm) = 10.96 (s, 1H), 10.66 (dt, ^{3}J = 13.0 Hz, 6.4 Hz, 1H), 8.64 (d, ^{3}J = 13.0 Hz, 1H), 7.98 (dd, ^{3}J = 7.8 Hz, ^{4}J = 1.4 Hz, 1H), 7.83 (d, ^{3}J = 8.2 Hz, 1H), 7.55 (ddd, ^{3}J = 8.3 Hz, 7.0 Hz, ^{4}J = 1.4 Hz, 1H), 7.33 (d, ^{3+5}J = 8.2 Hz, 1H), 7.29 (d, ^{3+5}J = 8.2 Hz, 1H), 7.17 (dd, ^{3}J = 7.6 Hz, 7.4 Hz, 1H), 4.70 (d, ^{3}J = 6.4 Hz, 2H), 3.36 (s, 2H), 2.12 (s, 6H). ^{13}C-NMR (125 MHz, DMSO-*d*$_6$, 300 K): δ_C = 166.1, 163.9, 153.9, 138.3, 137.7, 136.9, 132.9, 129.0, 127.5, 127.3, 122.9, 120.2, 118.6, 92.1, 63.0, 52.0, 44.9(2C). HR-MS: calcd for $C_{20}H_{22}N_3O_2$ [M+H]$^+$ 336.1707; found: 336.1710. Elemental analysis calcd (%) for $C_{20}H_{21}N_3O_2$ x 0.25 H_2O: C: 70.67, H: 6.38, N: 12.36; found: C: 70.74, H: 6.29, N: 12.32.

Series III_A: Aniline Derivatives

(*Z*)-4-[({2-[(Dimethylamino)methyl]phenyl}amino)methylene]isoquinoline-1,3(2*H*,4*H*)-dione TR-022

In a microwave vessel (20 mL), **TR-02** (250 mg, 1.23 mmol, 1.00 equiv) and 2-[(dimethylamino)methyl]aniline (203 mg, 1.35 mmol, 1.10 equiv) were suspended in MeOH (2.50 mL) and stirred in the microwave reactor (125 °C, 300 W) for 30 minutes. After cooling to room temperature, the solvent was removed under reduced pressure, the crude product was adsorbed on silica gel and purified by MPLC (0-10% MeOH in DCM over 10 minutes), yielding 325 mg (1.01 mmol, 82%) of **TR-022** as a yellow-brown solid. mp 172 °C. ^{1}H-NMR (500 MHz, DMSO-d_6, 300 K): δ_H (ppm) = 12.78 (d, ^3J = 13.2 Hz, 1H), 11.18 (s, 1H), 8.75 (d, ^3J = 13.2 Hz, 1H), 8.13 (d, ^3J = 8.3 Hz, 1H), 8.02 (dd, ^3J = 8.0 Hz, ^4J = 1.4 Hz, 1H), 7.81 (d, ^3J = 8.0 Hz, 1H), 7.60 (ddd, ^3J = 8.3 Hz, 6.9 Hz, ^4J= 1.4 Hz, 1H), 7.41 (td, ^3J = 7.7 Hz, ^4J = 1.4 Hz, 1H), 7.26 (td, ^3J = 7.5 Hz, ^4J = 1.0 Hz, 1H), 7.26 (dd, ^3J = 7.5 Hz, ^4J = 1.4 Hz, 1H), 7.13 (td, ^3J = 7.5 Hz, ^4J = 1.2 Hz, 1H), 3.45 (s, 2H), 2.22 (s, 6H). ^{13}C-NMR (125 MHz, DMSO-d_6, 300 K): δ_C (ppm) = 166.1, 163.9, 144.3, 139.5, 136.9, 133.1, 130.6, 128.8, 128.0, 127.5, 124.0 (2C), 121.0, 119.9, 116.9, 96.1, 61.2, 44.7 (2C). HR-MS: calcd for $C_{19}H_{20}N_3O_2$ [M+H]$^+$ 322.1550; found: 322.1556. Elemental analysis calcd (%) for $C_{19}H_{19}N_3O_2$ x 0.25 H_2O: C: 70.03, H: 6.03, N: 12.89; found: C: 70.04, H: 5,86, N: 12.81. Purity was determined by qNMR using maleic acid as an internal standard: 91.7%.

(*Z*)-4-[({3-[(Dimethylamino)methyl]phenyl}amino)methylene]isoquinoline-1,3(2*H*,4*H*)-dione TR-010

In a microwave vessel (20 mL), **TR-02** (200 mg, 0.98 mmol, 1.00 equiv) and 3-[(dimethylamino)methyl]aniline (177 mg, 1.18 mmol, 1.20 equiv) were suspended in MeOH (2.00 mL) and stirred in the microwave reactor (125 °C, 300 W) for 30 minutes. After cooling to room temperature, the precipitate was filtered, washed with MeOH (15 mL) and Et_2O (10 mL) and dried in high vacuum at 50 °C to obtain **TR-010** (213 mg, 0.66 mmol) as a yellow-orange solid with a yield of 68%. mp 145.8 °C. ^{1}H-NMR (500 MHz, DMSO-d_6, 300 K): δ_H (ppm) = 12.43 (d, ^3J = 12.6 Hz, 1H), 11.31 (s, 1H), 8.89 (d, ^3J = 12.6 Hz, 1H), 8.17 (d, ^3J = 8.3 Hz, 1H), 8.03 (dd, ^3J = 8.0 Hz, ^4J = 1.2 Hz, 1H), 7.62 (ddd, ^3J = 8.3 Hz, 6.9 Hz, ^4J = 1.4 Hz, 1H), 7.46 (ddd, ^3J = 7.7 Hz, ^4J = 2.3 Hz, 1.2 Hz, 1H), 7.43 (dd, ^4J = 1.7 Hz, 1.2 Hz, 1H), 7.37 (t, ^3J = 7.7 Hz, 1H), 7.28 (td, ^3J = 7.5 Hz, ^4J = 1.2 Hz, 1H), 7.11 (d, ^3J = 7.5 Hz, 1H), 3.43 (s, 2H), 2.18 (s, 6H). ^{13}C-NMR (100 MHz, DMSO-d_6, 300 K): δ_C (ppm) = 166.9, 163.8, 144.7, 140.8, 139.1, 136.5, 133.1, 129.4, 127.5, 125.0, 124.3, 121.0, 120.1, 117.8, 116.0, 95.6, 63.0, 45.0(2C). HR-MS: calcd for $C_{19}H_{20}N_3O_2$ [M+H]$^+$ 322.1550; found: 322.1542. Elemental analysis calcd (%) for $C_{19}H_{19}N_3O_2$ x 0.25 H_2O: C: 70.03, H: 6.03, N: 12.89; found: C: 70.12, H: 6.02, N: 12.77. Purity was determined by qNMR using maleic acid as an internal standard: 92.1%.

(Z)-4-[({4-[(Dimethylamino)methyl]phenyl}amino)methylene]isoquinoline-1,3(2*H*,4*H*)-dione TR-021

In a microwave vessel (20 mL), **TR-02** (250 mg, 1.23 mmol, 1.00 equiv) and 4-[(dimethylamino)methyl]aniline (203 mg, 1.35 mmol, 1.10 equiv) were suspended in MeOH (2.50 mL) and stirred in the microwave reactor (125 °C, 300 W) for 30 minutes. After cooling to room temperature, the solvent was removed under reduced pressure, the crude product was adsorbed on silica gel and purified by MPLC (0-10% MeOH in DCM over 10 min), yielding 342 mg (1.06 mmol, 87%) of **TR-021** as a yellow solid. mp 163.4 °C. ^{1}H-NMR (500 MHz, DMSO-*d*$_6$, 300 K): δ_H (ppm) = 12.41 (d, ^{3}J = 12.6 Hz, 1H), 11.29 (s, 1H), 8.88 (d, ^{3}J = 12.6 Hz, 1H), 8.15 (d, ^{3}J = 8.3 Hz, 1H), 8.03 (dd, ^{3}J = 8.0 Hz, ^{4}J = 1.2 Hz, 1H), 7.61 (ddd, ^{3}J = 8.4 Hz, 7.0 Hz, ^{4}J = 1.4 Hz, 1H), 7.50 (d, ^{3+5}J = 8.6 Hz, 1H), 7.32 (d, ^{3+5}J = 8.6 Hz, 1H), 7.27 (td, ^{3}J = 7.5 Hz, ^{4}J = 1.2 Hz, 1H), 3.38 (s, 2H), 2.15 (s, 6H). ^{13}C-NMR (125 MHz, DMSO-*d*$_6$, 300 K): δ_C (ppm) = 166.8, 163.8, 144.8, 138.0, 136.5, 135.2, 133.1, 130.0(2C), 127.5, 124.2, 121.0, 120.0, 117.3(2C), 95.4, 62.7, 44.8(2C). HR-MS: calcd for $C_{19}H_{20}N_3O_2$ [M+H]$^+$ 322.1550; found: 322.1561. Elemental analysis calcd (%) for $C_{19}H_{19}N_3O_2$ x 0.75 H_2O: C: 68.14, H: 6.17, N: 12.55; found: C: 68.32, H: 5.93, N: 12.35. Purity was determined by qNMR using maleic acid as an internal standard: 91.6%.

(*Z*)-4-{[(4-Isobutylphenyl)amino]methylen}isoquinoline-1,3(2*H*,4*H*)-dione ICTR-076

In a microwave vessel (20 mL), **TR-02** (262 mg, 1.29 mmol, 1.00 equiv) and 4-isobutylaniline 135 (0.27 mL, 1.68 mmol, 1.30 equiv) were suspended in MeOH (7.00 mL) and stirred in the microwave reactor (125 °C, 300 W) for 30 minutes. After cooling to room temperature, the precipitate was filtered, washed with MeOH (10 mL) and Et_2O (10 mL), and dried in high vacuum at 50 °C to afford **ICTR-076** (370 mg, 0.90 mmol) as a light yellow solid with a yield of 90%. mp 172.3 °C. ^{1}H-NMR (500 MHz, DMSO-*d*$_6$, 300 K): δ_H (ppm) = 12.40 (d, ^{3}J = 12.6 Hz, 1H), 11.27 (s, 1H), 8.87 (d, ^{3}J = 12.6 Hz, 1H), 8.15 (d, ^{3}J = 8.2 Hz, 1H), 8.03 (dd, ^{3}J = 8.0 Hz, ^{4}J = 1.2 Hz, 1H), 7.61 (ddd, ^{3}J = 8.4 Hz, 7.0 Hz, ^{4}J = 1.4 Hz, 1H), 7.46 (d, ^{3+5}J = 8.5 Hz, 2H), 7.27 (dd, ^{3}J = 7.7 Hz, 7.3 Hz, 1H), 7.20 (d, ^{3+5}J = 8.5 Hz, 2H), 2.45 (d, ^{3}J = 7.1 Hz, 2H), 1.83 (sm, 1H), 0.87 (d, ^{3}J = 6.6 Hz, 6H). ^{13}C-NMR (125 MHz, DMSO-*d*$_6$, 300 K): δ_C (ppm) = 166.7, 163.8, 144.9, 137.7, 137.1, 136.6, 133.1, 130.0(2C), 127.5, 124.1, 120.9, 119.9, 117.4(2C), 95.2, 43.9, 29.6, 22.0(2C). HR-MS: calcd for $C_{20}H_{20}N_2O_2Na_1$ [M+Na]$^+$ 343.1417; found: 343.1418. Elemental analysis calcd (%) for $C_{20}H_{20}N_2O_2$: C: 74.98, H: 6.29, N: 8.74; found: C: 74.73, H: 6.39, N: 8.75.

Synthesis of TR-036*HCl

In the case of the synthesis of **TR-036**, the application of GWP 2 only led to a low yield of 10% in two steps, starting from the nitro precursor 1-(bromomethyl)-3-nitrobenzene. Given that work-up of the iron reduction proved difficult, in this case the nitro group was reduced using Pd/C (10 %) and hydrazine monohydrate to yield **ICTR-122*2HCl** in a yield of 58 %. The subsequent conversion to **TR-036*HCl** was carried out analogously to the previous sequence, as shown below**.**

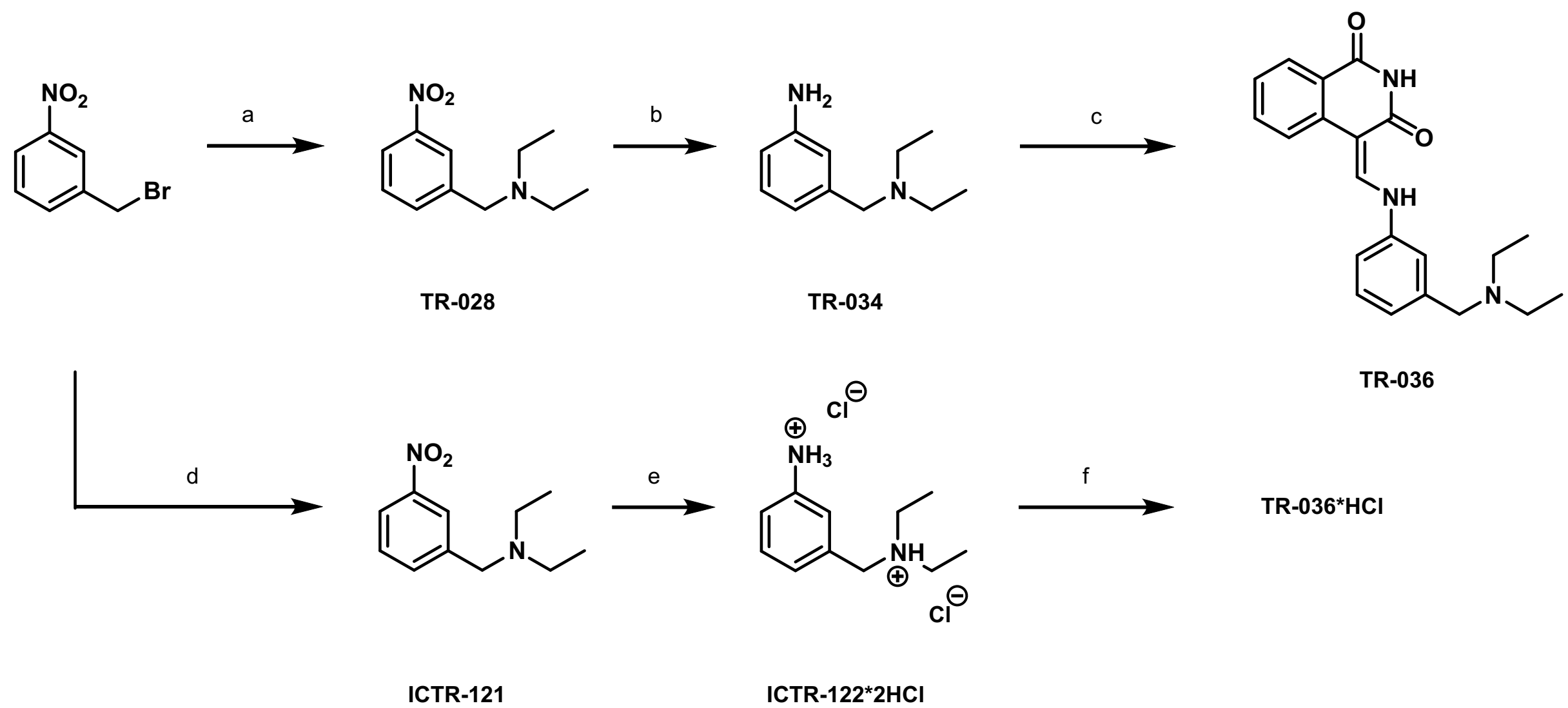

[a] Reagents and conditions: a) diethylamine, K_2CO_3, MeCN, @MW: 130 °C, 300 W, 30 min, 88%; b) Fe, NH_4Cl, $HCl_{(conc.)}$, H_2O/MeOH (1:1), 50 °C, 4 h; c) MeOH, @MW: 125 °C, 300 W, 30 min, 10% (o2s); d) diethylamine, THF, 60 °C, 2 h, 95%; e) 1: Pd/C (10%), NH_2NH_2*H_2O, EtOH, rt, 1 h; 2: 4 M $HCl_{(dioxane)}$, EtOAc, 58%; f) MeOH, @MW: 125 °C, 30 min, 66%.

N-Ethyl-*N*-(3-nitrobenzyl)ethanamine ICTR-121

To a solution of 1-(bromomethyl)-3-nitrobenzene (5.0 g, 23.15 mmol, 1.00 equiv) in THF (50 mL), diethylamine (9.0 mL, 86.81 mmol, 3.75 equiv) was added, and the mixture was stirred at 60 °C for 2 hours. After cooling to room temperature and upon completion of the reaction (according to TLC), it was diluted with H_2O (100 mL) and extracted with EtOAc (3 x 100 mL). The combined organic phase was washed with 1 M HCl (aq., 75 mL). The aqueous phase was adjusted to a basic pH with 2 *M* NaOH and then extracted with EtOAc (2 x 100 mL). The organic phases were combined, dried over $MgSO_4$, filtered, and the solvent was removed under reduced pressure yielding **ICTR-121** (4.583 g, 22.01 mmol) as an orange oil in a yield of 95%. ^{1}H-NMR (500 MHz, $CDCl_3$, 300 K): δ_H (ppm) = 8.22 (dd, ^4J = 2.3 Hz, 2.0 Hz, 1H), 8.09 (dd, ^3J = 8.0 Hz, ^4J = 2.3 Hz, 1H), 7.72 (d, ^3J = 7.7 Hz, 1H), 7.47 (dd, ^3J = 8.0 Hz, 7.7 Hz, 1H), 3.67 (s, 2H), 2.56 (q, ^3J = 7.2 Hz, 4H), 1.06 (t, ^3J = 7.2 Hz, 6H). ^{13}C-NMR (125 MHz, $CDCl_3$, 300 K): δ_C (ppm) = 148.5, 142.5, 135.0, 129.2, 123.7, 122.1, 57.0, 47.0(2C), 11.8(2C). HR-MS: calcd for $C_{11}H_{17}N_2O_2$ $[M+H]^+$ 209.1285; found: 209.1274.

3-[(Diethylammonio)methyl]benzenaminiumdichloride ICTR-122*2HCl

To a solution of **ICTR-121** (4.82 g, 23.15 mmol, 1.00 equiv) and Pd/C (500 mg, 0.47 mmol, 2 mol%, 10% Pd on activated carbon) in EtOH (50 mL), hydrazine monohydrate (2.91 mL, 60.19 mmol, 2.60 equiv) was slowly added dropwise and stirred at room temperature for 1 hour. The mixture was filtered over a Celite pad, washed with EtOH, and the solvent was removed under reduced pressure. The crude product was adsorbed on silica gel and purified by MPLC (10-100% EtOAc in CH over 25 min), yielding the product as a pale yellow oil. This was

redissolved in 50.0 mL EtOAc and treated with 4 *M* HCl (dioxane) (20.0 mL) and stirred overnight to obtain **ICTR-122*2HCl** (3.38 g, 13.46 mmol) as a white solid with a yield of 58%. ^{1}H-NMR (500 MHz, DMSO-d_6, 300 K): δ_H (ppm) = 11.10 (br s, 1H), 7.70 (d, ^{3}J = 7.7 Hz, 1H), 7.57 (dd, ^{4}J = 2.0 Hz, 1.4 Hz, 1H), 7.52 (dd, ^{3}J = 8.0 Hz, 7.7 Hz, 1H), 7.43 (ddd, ^{3}J = 8.0 Hz, ^{4}J = 2.0 Hz, 1.2 Hz, 1H), 4.31 (s, 2H), 3.05-2.99 (sm, 4H), 1.25 (t, ^{3}J = 7.3 Hz, 6H). ^{13}C-NMR (125 MHz, DMSO-d_6, 300 K): δ_C (ppm) = 133.8, 131.8, 130.0, 129.6, 125.0, 123.4, 53.9, 45.7(2C), 8.25(2C). HR-MS: calcd for $C_{11}H_{19}N_2$ [M+H]$^+$ 179.1543; found: 179.1539.

(*Z*)-*N*-(3-{[(1,3-Dioxo-2,3-dihydroisoquinoline-4(1*H*)-ylidene)methyl]amino}benzyl)-*N*-ethylethanaminiumchloride TR-036*HCl

To a solution of **ICTR-122*2HCl** (1.869 g, 7.44 mmol, 1.30 equiv) in MeOH (50.0 mL), Et_3N (2.58 mL, 3.25 equiv) was added dropwise and stirred for 10 minutes. Subsequently, **TR-02** (1.162 g, 5.72 mmol, 1.00 equiv) was added, and the mixture was stirred at room temperature. After 6 hours (no starting material remained according to TLC), the solvent was removed under reduced pressure. The remaining residue was resuspended in dioxane (50.0 mL), treated with 4 *M* HCl (dioxane), and stirred overnight at room temperature to obtain the corresponding hydrochloride. The solvent was removed under reduced pressure, and the crude product was dissolved in 8.0 mL of 5% MeCN in H_2O and purified by preparative HPLC through several runs (max. 400 mg crude product per run), yielding 1.46 g (3.78 mmol, 66%) of product **TR-036*HCl** as a yellow solid. mp 248.6 °C. ^{1}H-NMR (500 MHz, DMSO-d_6, 300 K): δ_H (ppm) = 12.36 (d, ^{3}J = 12.3 Hz, 1H), 11.34 (s, 1H), 11.02 (br s, 1H), 9.01 (d, ^{3}J = 12.3 Hz, 1H), 8.34 (d, ^{3}J = 8.3 Hz, 1H), 8.12 (dd, ^{4}J = 2.3 Hz, 1.7 Hz, 1H), 8.03 (dd, ^{3}J = 8.0 Hz, ^{4}J = 1.2 Hz, 1H), 7.60 (ddd, ^{3}J = 7.9 Hz, 7.6 Hz, ^{4}J = 1.4 Hz, 1H), 7.58 (ddd, ^{3}J = 8.0 Hz, ^{4}J = 2.0 Hz, ^{4}J = 1.2 Hz, 1H), 7.47 (dd, ^{3}J = 8.0 Hz, 7.7 Hz, 1H), 7.37 (dd, ^{3}J = 7.6 Hz, ^{4}J = 1.2 Hz, 1H), 7.30 (ddd, ^{3}J = 7.6 Hz, ^{4}J = 1.1 Hz, 1H), 4.32 (d, ^{3}J = 5.7 Hz, 2H), 3.13-3.03 (m, 4H), 1.29 (t, ^{3}J = 7.3 Hz, 6H). ^{13}C-NMR (125 MHz, DMSO-d_6, 300 K): δ_C (ppm) = 166.9, 163.8, 144.4, 139.6, 136.3, 133.2, 132.1, 130.0, 127.5, 126.6, 124.5, 121.1, 120.3, 119.4, 118.7, 96.2, 54.7, 45.7 (2C), 8.10 (2C). HR-MS: calcd for $C_{21}H_{24}N_3O_2$ [M]$^+$ 350.1863; found: 350.1857. Elemental analysis calcd (%) for $C_{21}H_{24}Cl_1N_3O_2$ x 0.75 H_2O: C: 63.15, H: 6.44, N: 10.52; found: C: 63.12, H: 6.23, N: 10.47. Purity was determined by qNMR using maleic acid as an internal standard: 98.8%.

Synthesis of TR-038

4-(3-Nitrobenzyl)morpholine TR-029

According to a slightly modified GWP 1 (130 °C, 300 W, 30 min), 1-(bromomethyl)-3-nitrobenzene (1000 mg, 4.63 mmol, 1.00 equiv), morpholine (0.48 mL, 5.55 mmol, 1.20 equiv), and K_2CO_3 (959 mg, 6.94 mmol, 1.50 equiv) were used. The crude product was adsorbed on silica gel and purified by MPLC (0-2% MeOH in DCM over 10 min), yielding 0.96 g (4.32 mmol, 93%) of **TR-029** as a yellow oil. ^{1}H-NMR (500 MHz, DMSO-d_6, 300 K): δ_H (ppm) = 8.16 (dd, ^4J = 2.0 Hz, 1.7 Hz, 1H), 8.12 (ddd, ^3J = 8.2 Hz, ^4J = 2.4 Hz, 1.2 Hz, 1H), 7.77 (ddd, ^3J = 7.5 Hz, ^4J = 1.7 Hz, 1.2 Hz, 1H), 7.63 (dd, ^3J = 8.0 Hz, 7.7 Hz, 1H), 3.61 (s, 2H), 3.59-3.57 (sm, 4H), 2.39-2.38 (sm, 4H). ^{13}C-NMR (125 MHz, DMSO-d_6, 300 K): δ_C (ppm) = 147.8, 140.5, 135.5, 129.7, 123.1, 122.0, 66.1, 61.1(2C), 53.0(2C). HR-MS: calcd for $C_{11}H_{15}N_2O_3$ $[M+H]^+$ 223.1077; found: 223.1074. Purity was determined by qNMR using maleic acid as an internal standard: 80.8%.

3-(Morpholinomethyl)aniline TR-037

Following GWP 2, **TR-029** (444 mg, 2.00 mmol, 1.00 equiv), NH_4Cl (53 mg, 1.00 mmol, 0.50 equiv), iron (1117 mg, 20 mmol, 10.0 equiv) were suspended in a mixture of H_2O (15.0 mL) and MeOH (15.0 mL). After the reaction was complete (according to TLC), the pH was adjusted to basic (8) with saturated $NaHCO_3$ (aq.), the mixture was filtered over Celite, and the pad was washed with MeOH. The solvent was removed under reduced pressure and the residue was dissolved in EtOAc (100.0 mL). The organic phase was washed with brine (50.0 mL), dried over $MgSO_4$, filtered, and the solvent was removed under reduced pressure to give 365 mg (1.90 mmol, 95%) of **TR-037**. The crude product was used in the next step without any further purification.

(*Z*)-4-({[3-(Morpholinomethyl)phenyl]amino}methylene)isoquinoline-1,3(2*H*,4*H*)-dione TR-038

In a microwave vessel (20 mL), **TR-02** (386 mg, 1.90 mmol, 1.00 equiv) and crude **TR-037** (365 mg, 1.90 mmol, 1.00 equiv) were suspended in MeOH (3.80 mL) and stirred in a microwave reactor (125 °C, 300 W) for 30 minutes. After cooling to room temperature, the solvent was removed under reduced pressure, and the residue was recrystallized from a mixture of MeOH/DMSO (20:1) affording **TR-038** (370 mg, 1.02 mmol) as a yellow solid with a yield of 51% over two steps. mp 213.9 °C. ^{1}H-NMR (500 MHz, DMSO-d_6, 300 K): δ_H (ppm) = 12.42 (d, ^3J = 12.6 Hz, 1H), 11.31 (s, 1H), 8.88 (d, ^3J = 12.6 Hz, 1H), 8.16 (d, ^3J = 8.3 Hz, 1H), 8.03 (dd, ^3J = 7.9 Hz, ^4J = 1.3 Hz, 1H), 7.62 (ddd, ^3J = 8.4 Hz, 7.0 Hz, ^4J = 1.4 Hz, 1H), 7.47 (ddd, ^3J = 8.0 Hz, ^4J = 2.0 Hz, 1.2 Hz, 1H), 7.43 (dd, ^4J = 2.0 Hz, 1.4 Hz, 1H), 7.38 (t, ^3J = 7.7 Hz, 1H), 7.28 (td, ^3J = 7.5 Hz, ^4J = 1.2 Hz, 1H), 7.13 (d, ^3J = 7.7 Hz, 1H), 3.60-3.58 (sm, 4H), 3.50 (s, 2H), 2.39-2.37 (sm, 4H). ^{13}C-NMR (125 MHz, DMSO-d_6, 300 K): δ_C (ppm) = 166.8, 163.7, 144.7, 139.7, 139.1, 136.5, 133.1, 129.5, 127.5, 125.1, 124.2, 121.0, 120.0, 118.1, 116.0, 95.6, 66.1(2C), 62.1, 53.2(2C). HR-MS: calcd for $C_{21}H_{22}N_3O_3$ $[M+H]^+$ 364.1656; found: 364.1653. Elemental analysis calcd (%) for $C_{21}H_{21}N_3O_3$: C: 69.41, H: 5.82, N: 11.56; found: C: 69.11, H: 5.72, N: 11.54.

Series III_B: Ortho substituted Aniline Derivatives

Synthesis of ICTR-022

N-Isopropyl-*N*-(2-nitrobenzyl)propan-2-amine ICTR-001

According to a slightly modified GWP 1 (120 °C, 150 W, 1 h), 1-(bromomethyl)-2-nitrobenzene (500 mg, 2.32 mmol, 1.00 equiv), diisopropylamine (0.36 mL, 2.55 mmol, 1.10 equiv), and K_2CO_3 (321 mg, 2.32 mmol, 1.00 equiv) were used in MeCN (7.00 mL). After complete conversion (according to TLC) and cooling to room temperature, the reaction mixture was taken up in EtOAc (50.00 mL) and extracted with 2 *M* HCl (aq., 3 x 30.00 mL). The combined aqueous phase was adjusted to a basic pH of 8-9 with K_2CO_3 and then extracted with DCM (3 x 50.00 mL). The combined organic phase was washed with brine (20.00 mL), dried over $MgSO_4$, and filtered. The solvent was removed under reduced pressure, the crude product was adsorbed on silica gel and purified by MPLC (10% EtOAc in CH, isocratic) to give **ICTR-001** (183 mg, 0.77 mmol) as a yellow oil in a yield of 33%. ^{1}H-NMR (500 MHz, $CDCl_3$, 300 K): δ_H (ppm) = 7.98 (d, ^3J = 7.7 Hz, 1H), 7.84 (d, ^3J = 8.0 Hz, 1H), 7.55 (t, ^3J = 7.7 Hz, 1H), 7.32 (dd, ^3J = 8.0 Hz, 7.5 Hz, 1H), 3.94 (s, 2H), 3.00 (sep., ^3J = 6,6 Hz, 2H), 1.00 (d, ^3J = 6.6 Hz, 12H). ^{13}C-NMR (125 MHz, $CDCl_3$, 300 K): δ_C (ppm) = 149.2, 139.1, 132.7, 130.8, 127.0, 124.2, 48.9(2C), 46.5, 20.8(4C). HR-MS: calcd for $C_{13}H_{21}N_2O_2$ $[M+H]^+$ 237.1598; found: 237.1588. Purity was determined by qNMR using maleic acid as an internal standard: 99.1%.

2-[(Diisopropylamino)methyl]aniline ICTR-017

According to GWP 3, **ICTR-001** (349 mg, 1.48 mmol, 1.00 equiv), NH_4Cl (40 mg, 0.74 mmol, 0.50 equiv) and iron (827 mg, 14.8 mmol, 10.0 equiv) were suspended in AcOH (20.0 mL). After the reaction was complete (according to TLC), the pH was adjusted to basic (14) with NaOH, the resulting mixture was filtered over Celite, and the pad was rinsed with MeOH. Subsequently, MeOH was removed under reduced pressure and the mixture was extracted with DCM (3 x 50.0 mL). The combined organic phase was washed with brine (50.0 mL), dried over $MgSO_4$, filtered, and the solvent removed under reduced pressure affording 254 mg (1.23 mmol, 83%) of **ICTR-017**. The crude product was immediately used in the next step without any further purification.

(*Z*)-4-[({2-[(Diisopropylamino)methyl]phenyl}amino)methylene]isoquinoline-1,3(2*H*,4*H*)-dione ICTR-022

In a microwave vessel (20 mL), **TR-02** (209 mg, 1.03 mmol, 1.00 equiv) and crude **ICTR-017** (254 mg, 1.23 mmol, 1.20 equiv) were suspended in MeOH (5.00 mL) and stirred in the microwave reactor (125 °C, 300 W) for 30 minutes. After cooling to room temperature, the precipitate was filtered, washed with MeOH (10 mL) and Et_2O (10 mL) and dried under high vacuum at 50 °C to yield **ICTR-022** (250 mg, 0.66 mmol) as a light yellow solid in a yield of 64%. mp 195 °C. ^{1}H-NMR (500 MHz, DMSO-d_6, 300 K): δ_H (ppm) = 12.63 (d, ^3J = 13.0 Hz, 1H), 11.18 (s, 1H), 8.62 (d, ^3J = 13.0 Hz, 1H), 8.06 (d, ^3J = 8.0 Hz, 1H), 8.02 (dd, ^3J = 8.0 Hz, ^4J = 1.1 Hz, 1H), 7.59 (d, ^3J = 7.6 Hz, 1H), 7.58 (td, ^3J = 7.8 Hz, ^4J = 1.4 Hz, 1H), 7.42 (d, ^3J = 7.6 Hz, 1H), 7.34 (td, ^3J = 7.7 Hz,

^4J = 1.1 Hz, 1H), 7.25 (t, ^3J = 7.6 Hz, 1H), 7.16 (t, ^3J = 7.6 Hz, 1H), 3.79 (s, 2H), 3.06 (sep., ^3J = 6.6 Hz, 2H), 1.06 (d, ^3J = 6.6 Hz, 12H). ^{13}C-NMR (125 MHz, DMSO-d_6, 300 K): δ_C (ppm) = 165.8, 163.8, 145.2, 139.5, 137.0, 133.1, 130.0, 129.9, 128.1, 127.5, 124.6, 124.0, 121.0, 119.9, 118.3, 96.3, 47.9 (2C), 46.7, 20.0 (4C). HR-MS: calcd for $C_{23}H_{28}N_3O_2$ [M+H]$^+$ 378.2176; found: 378.2175. Purity was determined by qNMR using maleic acid as an internal standard: 98.0%.

Synthesis of ICTR-023

1-(2-Nitrobenzyl)pyrrolidine ICTR-002

According to GWP 1 (120 °C, 150 W, 1 h), 1-(bromomethyl)-2-nitrobenzene (500 mg, 2.32 mmol, 1.00 equiv), pyrrolidine (0.22 mL, 2.55 mmol, 1.10 equiv), and K_2CO_3 (321 mg, 2.32 mmol, 1.00 equiv) were employed. The crude product was adsorbed on silica gel and purified by MPLC (0-5% MeOH in DCM over 10 min), yielding 344 mg (1.67 mmol, 72%) of **ICTR-002** as a yellow oil. ^{1}H-NMR (500 MHz, $CDCl_3$, 300 K): δ_H (ppm) = 7.85 (dd, ^3J = 8.3 Hz, ^4J = 1.4 Hz, 1H), 7.69 (d, ^3J = 7.7 Hz, 1H), 7.55 (td, ^3J = 7.6 Hz, ^4J = 1.4 Hz, 2H), 7.37 (ddd, ^3J = 8.0 Hz, 7.5 Hz, ^4J = 1.4 Hz, 1H), 3.93 (s, 2H), 2.52 (sm, 4H), 1.77 (sm, 4H). ^{13}C-NMR (125 MHz, $CDCl_3$, 300 K): δ_C (ppm) = 149.4, 135.3, 132.8, 130.9, 127.7, 124.4, 56.7, 54.4(2C), 23.8(2C). HR-MS: calcd for $C_{11}H_{15}N_2O_2$ [M+H]$^+$ 207.1128; found: 207.1121. Purity was determined by qNMR using maleic acid as an internal standard: 96.6%.

2-(Pyrrolidin-1-ylmethyl)aniline ICTR-021

According to GWP 3, **ICTR-002** (400 mg, 1.94 mmol, 1.00 equiv), NH_4Cl (52 mg, 0.97 mmol, 0.50 equiv) and iron (1084 mg, 19.4 mmol, 10.0 equiv) were suspended in AcOH (20.0 mL). After the reaction was completed (according to TLC), a basic pH (14) was adjusted using NaOH, the mixture was filtered over Celite, and the pad was washed with MeOH. Subsequently, MeOH was removed under reduced pressure and the mixture was extracted with DCM (3 x 50.0 mL). The combined organic phase was washed with brine (50.0 mL), dried over $MgSO_4$, filtered, and the solvent was removed under reduced pressure providing 286 mg (1.62 mmol, 84%) of **ICTR-021**. The crude product was used in the subsequent step without any further purification.

(*Z*)-4-({[2-(Pyrrolidin-1-ylmethyl)phenyl]amino}methylene)isoquinoline-1,3(2*H*,4*H*)-dione ICTR-023

In a microwave vessel (20 mL), **TR-02** (193 mg, 0.95 mmol, 1.00 equiv) and crude **ICTR-021** (200 mg, 1.13 mmol, 1.20 equiv) were suspended in MeOH (5.00 mL) and stirred in the microwave reactor (125 °C, 300 W) for 30 minutes. After cooling to room temperature, the solvent was removed under reduced pressure, and the crude product was adsorbed on silica gel and purified by MPLC (0-50% EtOAc in CH over 25 minutes), yielding 129 mg (0.37 mmol, 39%) of **ICTR-023** as a dark yellow solid. mp 148 °C. ^{1}H-NMR (500 MHz, DMSO-d_6, 300 K): δ_H (ppm) = 12.78 (d, ^3J = 13.0 Hz, 1H), 11.14 (s, 1H), 8.72 (d, ^3J = 13.0 Hz, 1H), 8.11 (d, ^3J = 8.2 Hz, 1H), 8.02 (dd, ^3J = 7.8 Hz, ^4J = 1.1 Hz, 1H), 7.78 (d, ^3J = 8.0 Hz, 1H), 7.59 (ddd, ^3J = 7.8 Hz, 7.6 Hz, ^4J = 1.4 Hz, 1H), 7.39 (td, ^3J = 7.7 Hz, ^4J = 1.1 Hz, 1H), 7.28 (dd, ^3J = 8.0 Hz, ^4J = 1.4 Hz, 1H), 7.25 (dd, ^3J = 7.6 Hz, 7.3 Hz, 1H),

7.12 (t, ^3J = 7.4 Hz, 1H), 3.67 (s, 2H), 2.52-2.49 (m, 4H), 1.78-1.73 (sm, 4H). ^{13}C-NMR (125 MHz, DMSO-d_6, 300 K): δ_C (ppm) = 166.0, 163.8, 144.5, 139.4, 136.9, 133.0, 129.9, 128.7, 128.6, 127.5, 124.1, 123.9, 121.0, 119.9, 117.0, 96.1, 57.4, 53.0 (2C), 23.3 (2C). HR-MS: calcd for $C_{21}H_{22}N_3O_2$ [M+H]$^+$ 348.1707; found: 348.1715. Purity was determined by qNMR using maleic acid as an internal standard: 97.5%.

Synthesis of ICTR-028

1-(2-Nitrobenzyl)piperidin ICTR-003

According to GWP 1 (120 °C, 150 W, 1 h), 1-(bromomethyl)-2-nitrobenzene (500 mg, 2.32 mmol, 1.00 equiv), piperidine (0.24 mL, 2.55 mmol, 1.10 equiv), and K_2CO_3 (321 mg, 2.32 mmol, 1.00 equiv) were unitized. Without further purification, product **ICTR-003** (507 mg, 2.30 mmol) was obtained as a yellow oil with a yield of 99%. ^{1}H-NMR (500 MHz, $CDCl_3$, 300 K): δ_H (ppm) = 7.80 (dd, ^3J = 8.0 Hz, ^4J = 1.4 Hz, 1H), 7.64 (d, ^3J = 7.5 Hz, 1H), 7.52 (td, ^3J = 7.6 Hz, ^4J = 1.4 Hz, 1H), 7.36 (ddd, ^3J = 8.0 Hz, 7.5 Hz, ^4J = 1.4 Hz, 1H), 3.73 (s, 2H), 2.35 (br s, 4H), 1.53 (sm, 4H), 1.43-1.39 (m, 2H). ^{13}C-NMR (125 MHz, $CDCl_3$, 300 K): δ_C (ppm) = 149.9, 134.8, 132.4, 131.0, 127.7, 124.4, 59.8, 54.8(2C), 26.1(2C), 24.4. HR-MS: calcd for $C_{12}H_{17}N_2O_2$ [M+H]$^+$ 221.1285; found: 221.1275. Purity was determined by qNMR using maleic acid as an internal standard: 87.1%.

2-(Piperidin-1-ylmethyl)aniline ICTR-024

According to GWP 3, **ICTR-003** (400 mg, 1.82 mmol, 1.00 equiv), NH_4Cl (49 mg, 0.91 mmol, 0.50 equiv) and iron (1017 mg, 18.2 mmol, 10.0 equiv) were suspended in AcOH (20.0 mL). After the reaction was completed (according to TLC), a basic pH (9) was adjusted using K_2CO_3, the mixture was filtered over Celite and the pad was washed with MeOH. Subsequently, MeOH was removed under reduced pressure and the mixture was extracted with DCM (3 x 50.0 mL). The combined organic phase was washed with brine (50.0 mL), dried over $MgSO_4$, filtered, and the solvent evaporated yielding 300 mg (1.58 mmol, 87%) of **ICTR-024** as a brown oil. The crude product was carried on to the next step without any further purification.

(*Z*)-4-({[2-(Piperidin-1-ylmethyl)phenyl]amino}methylene)isoquinoline-1,3(2*H*,4*H*)-dione ICTR-028

In a microwave vessel (20 mL), **TR-02** (140 mg, 0.69 mmol, 1.00 equiv) and crude **ICTR-024** (170 mg, 0.89 mmol, 1.30 equiv) were suspended in MeOH (5.00 mL) and stirred in the microwave reactor (125 °C, 300 W) for 30 minutes. After cooling to room temperature, the precipitate was filtered, washed with MeOH (10 mL) and Et_2O (10 mL), and dried in high vacuum at 50 °C to obtain product **ICTR-028** (173 mg, 0.48 mmol) as an orange solid with a yield of 69%. mp 213 °C. ^{1}H-NMR (500 MHz, DMSO-d_6, 300 K): δ_H (ppm) = 12.59 (d, ^3J = 13.2 Hz, 1H), 11.13 (s, 1H), 8.69 (d, ^3J = 13.2 Hz, 1H), 8.11 (d, ^3J = 8.0 Hz, 1H), 8.02 (dd, ^3J = 7.9 Hz, ^4J = 1.4 Hz, 1H), 7.77 (d, ^3J = 8.0 Hz, 1H), 7.59 (ddd, ^3J = 8.3 Hz, 6.9 Hz, ^4J = 1.4 Hz, 1H), 7.39 (ddd, ^3J = 7.9 Hz, 7.6 Hz, ^4J = 1.4 Hz, 1H), 7.26 (ddd, ^3J = 7.6 Hz, 7.5 Hz, ^4J = 0.9 Hz, 1H), 7.25 (d, ^3J = 7.9 Hz, 1H), 7.13 (td, ^3J = 7.5 Hz, ^4J = 1.2 Hz, 1H), 3.49 (s, 2H), 2.36 (br s, 4H), 1.59 (sm, 4H), 1.42-1.38 (m, 2H). ^{13}C-NMR (125 MHz, DMSO-d_6,

300 K): δ_C = 165.9, 163.8, 144.6, 139.6, 136.9, 133.0, 130.8, 128.7, 127.9, 127.5, 124.0, 124.0, 121.0, 119.9, 117.4, 96.2, 60.8, 53.8(2C), 25.0(2C), 24.0. HR-MS: calcd for $C_{22}H_{24}N_3O_2$ $[M+H]^+$ 362.1863; found: 362.1866. Elemental analysis calcd (%) for $C_{22}H_{23}N_3O_2$: C: 73.11, H: 6.41, N: 11.63; found: C: 72.74, H: 6.56, N: 11.54.

Synthesis of ICTR-036

1-(2-Nitrobenzyl)-1*H*-pyrrole ICTR-008

To a degassed solution of pyrrole (0.21 mL, 3.02 mmol, 1.30 equiv) in MeCN (7.0 mL), NaH (121 mg, 3.02 mmol, 1.30 equiv, 60% dispersion in mineral oil) was added and stirred for 20 minutes. Then, 1-(bromomethyl)-2-nitrobenzene (500 mg, 2.32 mmol, 1.00 equiv) was added, and the mixture was stirred for another 4 hours at room temperature. After the reaction was completed (according to TLC), the solvent was removed under reduced pressure and the crude product was adsorbed on silica gel. Purification by MPLC (0-7% EtOAc in CH over 10 minutes) yielded 272 mg (1.35 mmol, 58%) of **ICTR-008** as a brown oil. ^{1}H-NMR (300 MHz, DMSO-d_6, 300 K): δ_H (ppm) = 8.10 (dd, ^3J = 8.1 Hz, ^4J = 1.4 Hz), 7.67 (ddd, ^3J = 7.7 Hz, 7.5 Hz, ^4J = 1.2 Hz, 1H), 7.54 (ddd, ^3J = 7.9 Hz, 7.6 Hz, ^4J = 1.4 Hz, 1H), 6.83 (dd, ^3J = 2.6 Hz, ^4J = 1.7 Hz, 2H), 6.63 (dd, ^3J = 7.9 Hz, ^4J = 1.0 Hz, 1H), 6.10 (dd, ^3J = 2.6 Hz, ^4J = 1.7 Hz, 2H), 5.50 (s, 2H). ^{13}C-NMR (125 MHz, DMSO-d_6, 300 K): δ_C (ppm) = 146.9, 135.1, 134.2, 128.5, 128.3, 124.6, 121.7(2C), 108.5(2C), 49.3. HR-MS: calcd for $C_{11}H_{11}N_2O_2$ $[M+H]^+$ 203.0815; found: 203.0811.

2-[(1*H*-Pyrrol-1-yl)methyl]aniline ICTR-054

According to GWP 4, **ICTR-008** (350 mg, 1.73 mmol, 1.00 equiv), ammonium formate (764 mg, 12.11 mmol, 7.0 equiv) and Pd/C (138 mg, 0.13 mmol, 0.075 equiv, 10% Pd on activated carbon) were used in EtOH (35.0 mL). After a reaction time of 1.5 hours and complete conversion (according to TLC and staining with Ehrlich's reagent), the mixture was filtered over a Celite pad, thoroughly rinsed with MeOH, and finally the solvent was removed under reduced pressure. The residue was dissolved in DCM (30.0 mL) and washed with H_2O (30.0 mL), dried over $MgSO_4$, filtered, and the solvent was removed under reduced pressure giving rise to 257 mg (1.49 mmol, 86%) of **ICTR-054** as an orange oil. The crude product was used in the next reaction step without any further purification.

(*Z*)-4-[({2-[(1H-Pyrrol-1-yl)methyl]phenyl}amino)methylene]isoquinoline-1,3(2*H*,4*H*)-dione ICTR-036

In a microwave vessel (20 mL), **TR-02** (183 mg, 0.90 mmol, 1.00 equiv) and crude **ICTR-054** (200 mg, 1.16 mmol, 1.30 equiv) were suspended in MeOH (5.00 mL) and stirred in the microwave reactor (125 °C, 300 W) for 30 minutes. After cooling to room temperature, the precipitate was filtered, washed with MeOH (10 mL) and Et_2O (10 mL), and dried in high vacuum at 50 °C to afford **ICTR-036** (280 mg, 0.82 mmol) as a light yellow solid with a yield of 91%. mp 296 °C. ^{1}H-NMR (500 MHz, DMSO-d_6, 300 K): δ_H (ppm) = 12.78 (d, ^3J = 12.1 Hz, 1H), 11.48 (s, 1H), 8.95 (d, ^3J = 12.1 Hz, 1H), 8.19 (d, ^3J = 8.0 Hz, 1H), 8.04 (dd, ^3J = 8.0 Hz, ^4J = 1.1 Hz, 1H), 7.88

(d, ^3J = 8.2 Hz, 1H), 7.63 (td, ^3J = 7.6 Hz, ^4J = 1.4 Hz, 1H), 7.43 (td, ^3J = 7.7 Hz, ^4J = 1.1 Hz, 1H), 7.30 (t, ^3J = 7.6 Hz, 1H), 7.17 (t, ^3J = 7.6 Hz, 1H), 7.00 (dd, ^3J = 7.8 Hz, ^4J = 1.1 Hz, 1H), 6.88 (td, ^3J = 2.1 Hz, ^4J = 0.7 Hz, 2H), 6.04 (td, ^3J = 2.1 Hz, ^4J = 0.7 Hz, 2H), 5.25 (s, 2H). ^{13}C-NMR (125 MHz, DMSO-d_6, 300 K): δ_C (ppm) = 167.3, 163.8, 145.8, 136.9, 136.4, 133.3, 129.1, 128.1, 127.6, 124.9, 124.5, 121.2 (2C), 121.0, 120.2, 117.2, 108.3 (2C), 96.3, 48.2. HR-MS: calcd for $C_{21}H_{17}N_3O_2Na_1$ [M+Na]$^+$ 366.1213; found: 366.1215. Elemental analysis calcd (%) for $C_{21}H_{17}N_3O_2$: C: 73.45, H: 4.99, N: 12.24; found: C: 73.37, H: 5.14, N: 12.20.

Synthesis of ICTR-042

1-(2-Nitrobenzyl)-1*H*-1,2,4-triazole ICTR-011

According to GWP 1 (120 °C, 150 W, 1 h) using 1-(bromomethyl)-2-nitrobenzene (500 mg, 2.32 mmol, 1.00 equiv), 1H-1,2,4-triazole (176 mg, 2.55 mmol, 1.10 equiv), and K_2CO_3 (321 mg, 2.32 mmol, 1.00 equiv). After purification by MPLC (0-50% EtOAc in CH over 15 minutes), 269 mg (1.32 mmol, 57%) of **ICTR-011** were obtained as a yellow oil. ^{1}H-NMR (500 MHz, DMSO-d_6, 300 K): δ_H (ppm) = 8.64 (s, 1H), 8.11 (dd, ^3J = 8.3 Hz, ^4J = 1.4 Hz, 1H), 8.03 (s, 1H), 7.73 (ddd, ^3J = 7.7 Hz, 7.5 Hz, ^4J = 1.4 Hz, 1H), 7.62 (ddd, ^3J = 8.0 Hz, 7.5 Hz, ^4J = 1.4 Hz, 1H), 7.12 (dd, ^3J = 7.7 Hz, ^4J = 1.2 Hz, 1H), 5.80 (s, 2H). ^{13}C-NMR (125 MHz, DMSO-d_6, 300 K): δ_C (ppm) = 151.9, 147.6, 145.1, 134.1, 131.0, 130.2, 129.4, 124.9, 49.2. HR-MS: calcd for $C_9H_9N_4O_2$ [M+H]$^+$ 205.0720; found: 205.0715. Purity was determined by qNMR using maleic acid as an internal standard: 96.1%.

2-[(1*H*-1,2,4-Triazol-1-yl)methyl]aniline ICTR-041

According to GWP 4, **ICTR-011** (269 mg, 1.32 mmol, 1.00 equiv), ammonium formate (583 mg, 9.24 mmol, 7.0 equiv) and Pd/C (106 mg, 0.10 mmol, 0.075 equiv, 10% Pd on activated carbon) were used in EtOH (26.4 mL). After a reaction time of 1 hour and complete conversion (according to TLC and staining with Ehrlich's reagent), the mixture was filtered over a Celite pad and washed with MeOH. The solvent was removed under reduced pressure. The residue was dissolved in DCM (30.0 mL) and washed with H_2O (30.0 mL), dried over $MgSO_4$, filtered, and the solvent was removed using a rotary evaporator to furnish 145 mg (0.83 mmol, 63%) of **ICTR-041** as an oil, which crystallized into a pale orange solid over time. The crude product was carried on to the next reaction without any further purification.

(*Z*)-4-[({2-[(1*H*-1,2,4-Triazol-1-yl)methyl]phenyl}amino)methylene]isoquinoline-1,3(2*H*,4*H*)-dione ICTR-042

In a microwave vessel (20 mL), **TR-02** (126 mg, 0.62 mmol, 1.00 equiv) and crude **ICTR-041** (140 mg, 0.80 mmol, 1.30 equiv) were suspended in MeOH (5.00 mL) and stirred in the microwave reactor (125 °C, 300 W) for 30 minutes. After cooling to room temperature, the precipitate was filtered, washed with MeOH (10 mL) and Et_2O (10 mL), and dried in high vacuum at 50 °C to provide **ICTR-042** (194 mg, 0.56 mmol) as a yellow solid with a yield of 91%. mp 303 °C. ^{1}H-NMR (500 MHz, DMSO-d_6, 300 K): δ_H (ppm) = 12.75 (d, ^3J = 12.1 Hz, 1H), 11.36 (s, 1H), 8.85 (d, ^3J = 12.1 Hz, 1H), 8.66 (s, 1H), 8.14 (d, ^3J = 8.2 Hz, 1H), 8.04 (dd, ^3J = 7.8 Hz, ^4J = 1.1

Hz, 1H), 7.99 (s, 1H), 7.84 (d, ^3J = 8.0 Hz, 1H), 7.62 (ddd, ^3J = 7.7 Hz, 7.6 Hz, ^4J = 1.4 Hz, 1H), 7.48 (td, ^3J = 7.7 Hz, ^4J = 1.1 Hz, 1H), 7.31 (dd, ^3J = 7.6 Hz, ^4J = 1.1 Hz, 1H), 7.29 (td, ^3J = 7.4 Hz, ^4J = 1.1 Hz, 1H), 7.22 (td, ^3J = 7.4 Hz, ^4J = 1.1 Hz, 1H), 5.53 (s, 2H). ^{13}C-NMR (125 MHz, DMSO-d_6, 300 K): δ_C (ppm) = 166.9, 163.8, 151.9, 146.3, 144.1, 138.3, 136.4, 133.2, 130.5, 129.8, 127.5, 125.6, 125.1, 124.4, 121.0, 120.0, 118.6, 96.4, 48.4. HR-MS: calcd for $C_{19}H_{15}N_5O_2Na_1$ [M+Na]$^+$ 368.1118; found: 368.1118. Purity was determined by qNMR using maleic acid as an internal standard: 95.2%.

Synthesis of ICTR-058*HCl

tert-Butyl 4-(2-nitrobenzyl)piperazine-1-carboxylate ICTR-004

According to GWP 1 (120 °C, 150 W, 1 h), 1-(bromomethyl)-2-nitrobenzene (500 mg, 2.32 mmol, 1.00 equiv), 1-Boc-piperazine (475 mg, 1.48 mmol, 1.10 equiv), and K_2CO_3 (321 mg, 2.32 mmol, 1.00 equiv) were used. After purification of the crude product by MPLC (0-50% EtOAc in CH over 25 minutes), 381 mg (1.18 mmol, 51%) of **ICTR-004** were obtained as a yellow oil. ^{1}H-NMR (500 MHz, $CDCl_3$, 300 K): δ_H (ppm) = 7.81 (dd, ^3J = 7.7 Hz, ^4J = 1.4 Hz, 1H), 7.57 (d, ^3J = 7.7 Hz, 1H), 7.53 (td, ^3J = 7.5 Hz, ^4J = 1.4 Hz, 1H), 7.40 (ddd, ^3J = 7.7 Hz, 7.5 Hz, ^4J = 1.4 Hz, 1H), 3.79 (s, 2H), 3.37 (sm, 4H), 2.37 (sm, 4H), 1.44 (s, 9H). ^{13}C-NMR (125 MHz, $CDCl_3$, 300 K): δ_C (ppm) = 154.9, 150.0, 133.6, 132.5, 131.1, 128.2, 124.6, 79.8, 59.2(3C), 53.0(2C), 28.5(3C). HR-MS: calcd for $C_{16}H_{24}N_3O_4$ [M+H]$^+$ 322.1761; found: 322.1758. Purity was determined by qNMR using maleic acid as an internal standard: 99.2%.

tert-Butyl 4-(2-aminobenzyl)piperazine-1-carboxylate ICTR-029

According to GWP 4, **ICTR-004** (381 mg, 1.18 mmol, 1.00 equiv), ammonium formate (521 mg, 8.26 mmol, 7.0 equiv) and Pd/C (96 mg, 0.09 mmol, 0.075 equiv, 10% Pd on activated carbon) were used in EtOH (23.6 mL). After a reaction time of 1 hour and complete conversion (according to TLC and staining with Ehrlich's reagent), the reaction mixture was filtered over a Celite pad and washed with MeOH. The solvent was removed under reduced pressure. The residue was dissolved in DCM (30.0 mL) and washed with H_2O (30.0 mL), dried over $MgSO_4$, filtered, and the solvent was removed under reduced pressure. The crude product was adsorbed on silica gel and purified by MPLC (0-50% EtOAc in CH over 20 min), yielding 249 mg (0.86 mmol, 73%) of **ICTR-029** as an oil, which crystallized to a beige solid after a few days.

tert-Butyl (*Z*)-4-(2-{[(1,3-dioxo-2,3-dihydroisoquinolin-4(1*H*)-ylidene)methyl]amino}benz-yl)piperazine-1-carboxylate ICTR-034

In a microwave vessel (20 mL), **TR-02** (124 mg, 0.61 mmol, 1.00 equiv) and **ICTR-029** (249 mg, 0.85 mmol, 1.40 equiv) were suspended in MeOH (5.00 mL) and stirred in the microwave reactor (125 °C, 300 W) for 30 minutes. After cooling to room temperature, the solvent was evaporated to yield 340 mg (120%) of **ICTR-034** as a yellow solid, still contaminated with starting material ICTR-029. Since we were unable to separate the starting material from the product, the obtained crude product was further used in the next step without a complete

analytical characterization. ^{1}H-NMR (300 MHz, DMSO-d_6, 300 K): δ_H (ppm) = 12.68 (d, ^3J = 12.8 Hz, 1H), 11.16 (s, 1H), 8.73 (d, ^3J = 12.8 Hz, 1H), 8.13 (d, ^3J = 8.4 Hz, 1H), 8.02 (dd, ^3J = 7.9 Hz, ^4J = 1.1 Hz, 1H), 7.79 (d, ^3J = 8.3 Hz, 1H), 7.60 (dd, ^3J = 7.6 Hz, ^4J = 1.3 Hz, 1H), 7.42 (ddd, ^3J = 7.9 Hz, ^3J = 7.5 Hz, ^4J = 1.1 Hz, 1H), 7.26 (t, ^3J = 7.5 Hz, 1H), 7.27 (td, ^3J = 7.5 Hz, ^4J = 1.2 Hz, 1H), 7.14 (dd, ^3J = 7.3 Hz, ^3J = 7.2 Hz, 1H), 3.56 (s, 2H), 3.43 (m, 4H), 2.37 (m, 4H), 1.40 (s, 9H).

(*Z*)-4-(2-{[(1,3-Dioxo-2,3-dihydroisoquinolin-4(1*H*)-ylidene)methyl]amino}benzyl)piperazin-1-ium chloride ICTR-058*HCl

Crude **ICTR-034** (453 mg, 0.98 mmol, 1.00 equiv) was treated with 4 M HCl in dioxane (2.45 mL, 10.00 equiv) and stirred at room temperature for 2-3 hours. Subsequently, the solvent was removed, and the residue (two batches of 275 mg each) was dissolved in 5 mL of 5% MeCN in H_2O and purified by preparative HPLC. A total of 308 mg (0.77 mmol, 79% o2s) of **ICTR-058*HCl** was obtained as a yellow solid. mp 263.9 °C. ^{1}H-NMR (500 MHz, DMSO-d_6, 300 K): δ_H (ppm) = 12.64 (d, ^3J = 13.2 Hz, 1H), 11.23 (s, 1H), 9.20 (br s, 2H), 8.75 (d, ^3J = 13.2 Hz, 1H), 8.14 (d, ^3J = 8.0 Hz, 1H), 8.02 (dd, ^3J = 8.0 Hz, ^4J = 1.4 Hz, 1H), 7.82 (d, ^3J = 8.0 Hz, 1H), 7.60 (ddd, ^3J = 8.4 Hz, 7.1 Hz, ^4J = 1.4 Hz, 1H), 7.43 (td, ^3J = 7.7 Hz, ^4J = 1.4 Hz, 1H), 7.32 (dd, ^3J = 7.5 Hz, ^4J = 1.4 Hz, 1H), 7.27 (td, ^3J = 7.5 Hz, ^4J = 1.2 Hz, 1H), 7.16 (td, ^3J = 7.5 Hz, ^4J = 1.4 Hz, 1H), 3.63 (s, 2H), 3.17-3.15 (m, 4H), 2.65 (m, 4H). ^{13}C-NMR (125 MHz, DMSO-d_6, 300 K): δ_C (ppm) = 166.2, 163.8, 144.8, 139.7, 136.8, 133.1, 131.0, 129.2, 127.5, 126.6, 124.2, 124.1, 121.0, 120.0, 117.6, 96.2, 59.5, 48.9 (2C), 42.6 (2C). HR-MS: calcd for $C_{21}H_{23}N_4O_2$ $[M+H]^+$ 363.1816; found: 363.1818. Elemental analysis calcd (%) for $C_{21}H_{23}Cl_1N_4O_2$ x 1.5 H_2O: C: 59.22, H: 6.15, N: 13.15; found: C: 59.35, H: 6.30, N: 13.13.

Synthesis of ICTR-035

1-Methyl-4-(2-nitrobenzyl)piperazine ICTR-006

According to GWP 1 (120 °C, 150 W, 1 hour), 1-(bromomethyl)-2-nitrobenzene (500 mg, 2.32 mmol, 1.00 equiv), 1-methylpiperazine (0.28 mL, 2.55 mmol, 1.10 equiv), and K_2CO_3 (321 mg, 2.32 mmol, 1.00 equiv) were employed. The crude product was adsorbed on silica gel and purified by MPLC (0-5% MeOH in DCM over 10 minutes), yielding 397 mg (1.69 mmol, 73%) of **ICTR-006** as a yellow oil. ^{1}H-NMR (500 MHz, $CDCl_3$, 300 K): δ_H (ppm) = 7.79 (dd, ^3J = 8.0 Hz, ^4J = 1.4 Hz, 1H), 7.57 (dd, ^3J = 7.7 Hz, ^4J = 1.2 Hz, 1H), 7.51 (td, ^3J = 7.5 Hz, ^4J = 1.2 Hz, 1H), 7.37 (ddd, ^3J = 8.0 Hz, 7.3 Hz, ^4J = 1.4 Hz, 1H), 3.78 (s, 2H), 2.45 (br s, 8H), 2.26 (s, 3H). ^{13}C-NMR (125 MHz, $CDCl_3$, 300 K): δ_C (ppm) = 150.0, 134.0, 132.4, 131.1, 128.0, 124.5, 59.1, 55.2(2C), 53.2(2C), 46.1. HR-MS: calcd for $C_{12}H_{18}N_3O_2$ $[M+H]^+$ 236.1394; found: 236.1384. Purity was determined by qNMR using maleic acid as an internal standard: 99.3%.

2-[(4-Methylpiperazin-1-yl)methyl]aniline ICTR-027

According to GWP 3, **ICTR-006** (400 mg, 1.70 mmol, 1.00 equiv), NH_4Cl (46 mg, 0.85 mmol, 0.50 equiv), iron (950 mg, 17.0 mmol, 10.0 equiv) were used in AcOH (20.0 mL). After the reaction was completed (according to

TLC), a basic pH (9) was adjusted using K_2CO_3, the mixture was filtered over Celite, and the pad was washed with MeOH. Subsequently, MeOH was removed under reduced pressure, and the residue was taken up in DCM (3 x 50.0 mL). The combined organic phase was washed with brine (50.0 mL), dried over $MgSO_4$, filtered, and the solvent was removed under reduced pressure, yielding 270 mg (1.32 mmol, 78%) of **ICTR-027** as a brown oil. This was further reacted in the next step without any additional purification.

(*Z*)-4-[({2-[(4-Methylpiperazin-1-yl)methyl]phenyl}amino)methylene]isoquinoline-1,3(2*H*,4*H*)-dione ICTR-035

In a microwave vessel (20 mL), **TR-02** (191 mg, 0.94 mmol, 1.00 equiv) and **ICTR-027** (270 mg, 1.32 mmol, 1.40 equiv) were suspended in MeOH (5.00 mL) and stirred in the microwave reactor (125 °C, 300 W) for 30 minutes. After cooling to room temperature, the precipitate was filtered, washed with MeOH (10 mL) and Et_2O (10 mL), and dried in high vacuum at 50 °C to afford **ICTR-035** (215 mg, 0.57 mmol) as a yellow-brown solid with a yield of 61%. mp 237 °C. ^{1}H-NMR (500 MHz, DMSO-d_6, 300 K): δ_H (ppm) = 12.52 (d, ^3J = 13.0 Hz, 1H), 11.16 (s, 1H), 8.73 (d, ^3J = 13.0 Hz, 1H), 8.12 (d, ^3J = 8.0 Hz, 1H), 8.02 (d, ^3J = 7.7 Hz, 1H), 7.81 (d, ^3J = 8.0 Hz, 1H), 7.60 (t, ^3J = 7.3 Hz, 1H), 7.41 (t, ^3J = 7.6 Hz, 1H), 7.28-7.25 (m, 2H), 7.13 (dd, ^3J = 7.5 Hz, 7.2 Hz, 1H), 3.52 (s, 2H), 2.62-2.33 (br s, 8H), 2.18 (s, 3H). ^{13}C-NMR (125 MHz, DMSO-d_6, 300 K): δ_C (ppm) = 166.0, 163.8, 144.7, 139.5, 136.9, 133.0, 130.9, 128.9, 127.5, 127.5, 124.0, 121.0, 119.9, 117.3, 96.2, 59.9, 54.2 (2C), 52.2 (2C), 45.5. HR-MS: calcd for $C_{22}H_{25}N_4O_2$ [M+H]$^+$ 377.1972; found: 377.1970. Purity was determined by qNMR using maleic acid as an internal standard: 92.1%.

Synthesis of ICTR-040

4-Methyl-1-(2-nitrobenzyl)piperidine ICTR-005

According to GWP 1 (120 °C, 150 W, 1 hour), 1-(bromomethyl)-2-nitrobenzene (500 mg, 2.32 mmol, 1.00 equiv), 4-methylpiperidine (0.3 mL, 2.55 mmol, 1.10 equiv), and K_2CO_3 (321 mg, 2.32 mmol, 1.00 equiv) were used. The crude product was adsorbed on silica gel and purified by MPLC (0-5% MeOH in DCM over 10 minutes), yielding 393 mg (1.68 mmol, 72%) of **ICTR-005** as a yellow oil. ^{1}H-NMR (500 MHz, $CDCl_3$, 300 K): δ_H (ppm) = 7.80 (dd, ^3J = 8.0 Hz, ^4J = 1.4 Hz, 1H), 7.64 (d, ^3J = 7.7 Hz, 1H), 7.52 (td, ^3J = 7.6 Hz, ^4J = 1.4 Hz, 1H), 7.36 (ddd, ^3J = 8.0 Hz, 7.5 Hz, ^4J = 1.4 Hz, 1H), 3.74 (s, 2H), 2.75-2.72 (sm, 2H), 2.03-1.98 (sm, 2H), 1.57-1.54 (sm, 2H) 1.35-1.30 (m, 1H), 1.23-1.15 (sm, 2H), 0.90 (d, ^3J = 6.3 Hz, 3H). ^{13}C-NMR (125 MHz, $CDCl_3$, 300 K): δ_C (ppm) = 149.9, 134.9, 132.5, 131.0, 127.7, 124.4, 59.4, 54.2 (2C), 34.5 (2C), 30.8, 22.0. HR-MS: calcd for $C_{13}H_{19}N_2O_2$ [M+H]$^+$: 235.1441; found: 235.1432. Purity was determined by qNMR using maleic acid as an internal standard: 94.2%.

2-[(4-Methylpiperidin-1-yl)methyl]aniline ICTR-026

According to GWP 3, **ICTR-005** (400 mg, 1.71 mmol, 1.00 equiv) and NH_4Cl (46 mg, 0.86 mmol, 0.50 equiv), iron (955 mg, 17.10 mmol, 10.0 equiv) were used in AcOH (20.0 mL). After the reaction was completed

(according to TLC), a basic pH (9) was adjusted using K_2CO_3, the mixture was filtered over Celite, and the pad was washed with MeOH. Subsequently, MeOH was removed under reduced pressure, and the residue was taken up in DCM (3 x 50.0 mL). The combined organic phase was washed with brine (50.0 mL), dried over $MgSO_4$, filtered, and the solvent was removed under reduced pressure giving rise to 347 mg (1.70 mmol, 99%) of **ICTR-026** as a brown oil. This was further reacted in the next step without any additional purification.

(*Z*)-4-[({2-[(4-Methylpiperidin-1-yl)methyl]phenyl}amino)methylene]isoquinoline-1,3(2*H*,4*H*)-dione ICTR-040

In a microwave vessel (20 mL), **TR-02** (191 mg, 0.94 mmol, 1.00 equiv) and **ICTR-026** (250 mg, 1.22 mmol, 1.30 equiv) were suspended in MeOH (5.00 mL) and stirred in the microwave reactor (125 °C, 300 W) for 30 minutes. After cooling to room temperature, the solvent was removed using a rotary evaporator, and the crude product was adsorbed on silica gel and purified by MPLC (0-50% EtOAc in CH over 45 minutes), yielding 164 mg (0.44 mmol, 47%) of **ICTR-040** as a yellow solid. mp 150 °C. ^{1}H-NMR (500 MHz, DMSO-d_6, 300 K): δ_H (ppm) = 12.55 (d, ^{3}J = 13.0 Hz, 1H), 11.13 (s, 1H), 8.71 (d, ^{3}J = 13.0 Hz, 1H), 8.11 (d, ^{3}J = 8.2 Hz, 1H), 8.02 (dd, ^{3}J = 8.0 Hz, ^{4}J = 1.4, 1H), 7.79 (d, ^{3}J = 8.0 Hz, 1H), 7.59 (ddd, ^{3}J = 7.8 Hz, 7.6 Hz, ^{4}J = 1.4 Hz, 1H), 7.40 (td, ^{3}J = 7.7 Hz, ^{4}J = 1.1 Hz, 1H), 7.26 (t, ^{3}J = 7.3 Hz, 1H), 7.25 (d, ^{3}J = 7.3 Hz, 1H), 7.13 (t, ^{3}J = 7.3 Hz, 1H), 3.50 (s, 2H), 2.82 (d, $^{3}J_{trans}$ = 10.8 Hz, 2H), 1.95 (t, $^{3}J_{trans}$ = 10.3 Hz, 2H), 1.54 (d, $^{3}J_{cis}$ = 9.8 Hz, 2H), 1.35-1.30 (m, 3H), 0.89 (d, ^{3}J = 5.5 Hz, 3H). ^{13}C-NMR (125 MHz, DMSO-d_6, 300 K): δ_C (ppm) = 166.0, 163.8, 144.6, 139.6, 136.9, 133.0, 130.8, 128.7, 127.9, 127.5, 124.0(2C), 121.0, 119.9, 117.3, 96.2, 60.4, 53.2 (2C), 33.5 (2C), 30.3, 21.7. HR-MS: calcd for $C_{23}H_{26}N_3O_2$ [M+H]$^+$: 376.2020; found: 376.2023. Elemental analysis calcd (%) for $C_{23}H_{25}N_3O_2$ x 0.5 H_2O: C: 71.85, H: 6.82, N: 10.93; found: C: 72.23, H: 6.81, N: 10.84.

Synthesis of ICTR-050

1-Ethyl-4-(2-nitrobenzyl)piperazine ICTR-039

According to GWP 1 (120 °C, 150 W, 1 hour), 1-(bromomethyl)-2-nitrobenzene (500 mg, 2.32 mmol, 1.00 equiv), 1-ethylpiperazine (0.32 mL, 2.55 mmol, 1.10 equiv), and K_2CO_3 (321 mg, 2.32 mmol, 1.00 equiv) were used. Without any further purification, **ICTR-039** (570 mg, 2.29 mmol) was obtained as a yellow-brown oil with a yield of 99%. ^{1}H-NMR (500 MHz, $CDCl_3$, 300 K): δ_H (ppm) = 7.79 (dd, ^{3}J = 8.0 Hz, ^{4}J = 1.2 Hz, 1H), 7.57 (dd, ^{3}J = 7.7 Hz, ^{4}J = 1.4 Hz, 1H), 7.51 (td, ^{3}J = 7.5 Hz, ^{4}J = 1.2 Hz, 1H), 7.38 (td, ^{3}J = 7.6 Hz, ^{4}J = 1.4 Hz, 1H), 3.79 (s, 2H), 2.35-2.23 (br m, 8H), 2.41 (q, ^{3}J = 7.2 Hz, 2H), 1.07 (t, ^{3}J = 7.2 Hz, 3H). ^{13}C-NMR (125 MHz, $CDCl_3$, 300 K): δ_C (ppm) = 150.0, 134.0, 132.4, 131.1, 128.0, 124.5, 59.1, 53.1 (2C), 52.8 (2C), 52.3, 12.0. HR-MS: calcd for $C_{13}H_{20}N_3O_2$ [M+H]$^+$: 250.1550; found: 250.1545. Purity was determined by qNMR using maleic acid as an internal standard: 83.6%.

2-[(4-Ethylpiperazin-1-yl)methyl]aniline ICTR-047

According to GWP 4, **ICTR-039** (500 mg, 2.01 mmol, 1.00 equiv), ammonium formate (887 mg, 14.07 mmol, 7.0 equiv) and Pd/C (160 mg, 0.15 mmol, 0.075 equiv, 10% Pd on activated carbon) were employed in EtOH (40.0 mL). After a reaction time of 1 hour and complete conversion (according to TLC and staining with Ehrlich's reagent), the reaction mixture was filtered over a Celite pad and washed with MeOH. The solvent was removed under reduced pressure. The residue was redissolved in DCM (50.0 mL) and washed with H_2O (20.0 mL). The aqueous phase was adjusted to a pH of 9 with K_2CO_3 and extracted again with DCM. The organic phases were combined, dried over $MgSO_4$, and the solvent was removed under reduced pressure, yielding 318 mg (1.45 mmol, 72%) of **ICTR-047** as a light brown oil. This was further reacted in the next step without any additional purification.

(*Z*)-4-[({2-[(4-Ethylpiperazin-1-yl)methyl]phenyl}amino)methylene]isoquinoline-1,3(2*H*,4*H*)-dione ICTR-050

In a microwave vessel (20 mL), **TR-02** (179 mg, 0.88 mmol, 1.00 equiv) and **ICTR-047** (250 mg, 1.14 mmol, 1.30 equiv) were suspended in MeOH (5.00 mL) and stirred in the microwave reactor (125 °C, 300 W) for 30 minutes. After cooling to room temperature, the solvent was evaporated and the remaining residue was purified by recrystallization from MeOH, affording 127 mg (0.33 mmol, 37%) of **ICTR-050** as a yellow solid. mp 155.7 °C. ^{1}H-NMR (500 MHz, DMSO-*d*$_6$, 300 K): δ_H (ppm) = 12.52 (d, ^3J = 13.2 Hz, 1H), 11.16 (s, 1H), 8.72 (d, ^3J = 13.2 Hz, 1H), 8.12 (d, ^3J = 8.3 Hz, 1H), 8.02 (dd, ^3J = 8.0 Hz, ^4J = 1.4 Hz, 1H), 7.81 (d, ^3J = 8.0 Hz, 1H), 7.59 (ddd, ^3J = 8.4 Hz, 7.0 Hz, ^4J = 1.4 Hz, 1H), 7.40 (td, ^3J = 7.7 Hz, ^4J = 1.4 Hz, 1H), 7.26 (sm, 2H), 7.13 (td, ^3J = 7.5 Hz, ^4J = 0.9 Hz, 1H), 3.51 (s, 2H), 2.42 (br s, 8H), 2.29 (q, ^3J = 7.2 Hz, 2H), 0.97 (t, ^3J = 7.2 Hz, 3H). ^{13}C-NMR (125 MHz, DMSO-*d*$_6$, 300 K): δ_C (ppm) = 166.0, 163.8, 144.6, 139.5, 136.9, 133.0, 130.9, 128.8, 127.5, 127.5, 124.0 (2C), 121.0, 119.9, 117.2, 96.1, 59.9, 52.5 (2C), 52.0 (2C), 51.5, 12.1. HR-MS: calcd for $C_{23}H_{27}N_4O_2$ $[M+H]^+$: 391.2129; found: 391.2134. Elemental analysis calcd (%) for $C_{23}H_{26}N_4O_2$: C: 70.75, H: 6.71, N: 14.35; found: C: 70.63, H: 6.86, N: 14.32.

Synthesis of ICTR-086*HCl

N-Ethyl-*N*-(2-nitrobenzyl)ethanaminium chloride ICTR-084*HCl

According to a slightly modified GWP 1 (120 °C, 150 W, 1 hour), 1-(bromomethyl)-2-nitrobenzene (500 mg, 2.31 mmol, 1.00 equiv), diethylamine (0.31 mL, 3.00 mmol, 1.3 equiv), and K_2CO_3 (319 mg, 2.31 mmol, 1.00 equiv) were used. The crude product was adsorbed on silica gel and purified by MPLC (0-5% MeOH in DCM over 20 minutes) and then converted into the corresponding hydrochloride with 2 M HCl in Et_2O, yielding **ICTR-084*HCl** (336 mg, 1.37 mmol) as a beige solid with a yield of 59%. mp 130.9 °C. ^{1}H-NMR (500 MHz, $CDCl_3$, 300 K): δ_H (ppm) = 12.57 (br s, 1H), 8.68 (dd, ^3J = 8.0 Hz, ^4J = 1.2 Hz, 1H), 8.03 (dd, ^3J = 8.0 Hz, ^4J = 1.4 Hz, 1H), 7.80 (td, ^3J = 7.7 Hz, ^4J = 1.2 Hz, 1H), 7.62 (td, ^3J = 7.9 Hz, ^4J = 1.4 Hz, 1H), 4.56 (d, ^3J = 6.6 Hz, 2H), 3.18 (dq, ^3J = 7.3 Hz, 4.6 Hz, 4H), 1.43 (t, ^3J = 7.3 Hz, 6H). ^{13}C-NMR (125 MHz, $CDCl_3$, 300 K): δ_C (ppm) = 149.9, 135.0,

134.8, 131.2, 125.4, 124.1, 51.2, 46.5 (2C), 8.7 (2C). HR-MS: calcd for $C_{11}H_{17}N_2O_2$ $[M+H]^+$: 209.1285; found: 209.1279. Purity was determined by qNMR using maleic acid as an internal standard: 94.5%.

2-([Diethylamonio]methyl)benzenaminium dichloride ICTR-085*2HCl

To a suspension of **ICTR-084*HCl** (1.00 g, 4.80 mmol, 1.00 equiv) and 10% Pd/C (106 mg, 0.10 mmol, 2 mol%) in EtOH (10 mL), hydrazine monohydrate (0.61 mL, 12.48 mmol, 2.60 equiv) was slowly added dropwise and stirred at room temperature for 1 hour. Subsequently, the mixture was filtered over a Celite pad, washed with EtOH, and the solvent was removed under reduced pressure. The crude product was adsorbed on silica gel and purified by MPLC (10-100% EtOAc in CH over 25 minutes), yielding the **ICTR-085** as a pale yellow oil. This oil was redissolved in EtOAc (10 mL) and treated with 4 M HCl in dioxane (4 mL) and stirred overnight, resulting in the corresponding product **ICTR-085*2HCl** (1.01 g, 4.02 mmol) as a white solid with a yield of 84%. ^{1}H-NMR (500 MHz, DMSO-d_6, 300 K): δ_H (ppm) = 10.72 (s, 1H), 10.00 (br s, 3H), 7.69 (dd, ^3J = 7.7 Hz, ^4J = 1.2 Hz, 1H), 7.51 (ddd, ^3J = 8.0 Hz, 7.2 Hz, ^4J = 1.2 Hz, 1H), 7.46 (d, ^3J = 7.7 Hz, 1H), 7.34 (dd, ^3J = 7.5 Hz, 7.2 Hz, 1H), 4.59 (s, 2H), 3.15 (br s, 4H), 1.29 (t, ^3J = 7.2 Hz, 6H). ^{13}C-NMR (125 MHz, DMSO-d_6, 300 K): δ_C (ppm) = 135.7, 134.3, 131.2, 126.1, 123.2, 122.3, 51.0, 46.1 (2C), 8.4 (2C). HR-MS: calcd for $C_{11}H_{19}N_2$ $[M+H]^+$: 179.1543; found: 179.1535.

(*Z*)-N-(2-{[(1,3-Dioxo-2,3-dihydroisoquinolin-4(1*H*)-ylidene)methyl]amino}benzyl)-*N*-ethyl-ethanaminium chloride ICTR-086*HCl

To a solution of **ICTR-085*2HCl** (322 mg, 1.50 mmol, 1.30 equiv) in MeOH (10.0 mL), Et_3N (0.73 mL, 1.95 equiv) was added dropwise and stirred for 10 minutes. Subsequently, **TR-02** (250 mg, 1.15 mmol, 1.00 equiv) was added and stirred at room temperature until complete reaction (TLC: no starting material remained). After 1 hour, the solvent was removed under reduced pressure. The residue was resuspended in dioxane (2.0 mL), treated with 4 M HCl in dioxane, and stirred overnight at room temperature to obtain the corresponding hydrochloride. The solvent was removed under reduced pressure, and the crude product was dissolved in 6.0 mL of 5% MeCN in H_2O and purified by preparative HPLC, yielding 138 mg (0.36 mmol, 31%) of **ICTR-086*HCl** as a yellow solid. mp 163.6 °C. ^{1}H-NMR (500 MHz, DMSO-d_6, 300 K): δ_H (ppm) = 12.72 (d, ^3J = 11.7 Hz, 1H), 11.42 (s, 1H), 10.73 (br s, 1H), 8.88 (d, ^3J = 11.7 Hz, 1H), 8.17 (d, ^3J = 8.3 Hz, 1H), 8.04 (dd, ^3J = 8.0 Hz, ^4J = 1.2 Hz, 1H), 7.92 (d, ^3J = 8.0 Hz, 1H), 7.80 (dd, ^3J = 7.7 Hz, ^4J = 0.9 Hz, 1H), 7.63 (ddd, ^3J = 8.3 Hz, 7.0 Hz, ^4J = 1.4 Hz, 1H), 7.58 (td, ^3J = 7.7 Hz, ^4J = 1.2 Hz, 1H), 7.31 (td, ^3J = 7.5 Hz, ^4J = 0.9 Hz, 1H), 7.30 (td, ^3J = 8.0 Hz, 7.2 Hz, ^4J = 0.9 Hz, 1H), 4.37 (d, ^3J = 5.4 Hz, 2H), 3.19-3.10 (sm, 4H), 1.28 (t, ^3J = 7.3 Hz, 6H). ^{13}C-NMR (125 MHz, DMSO-d_6, 300 K): δ_C (ppm) = 167.3, 163.7, 146.8, 139.7, 136.3, 133.3, 133.2, 131.1, 127.5, 125.0, 124.5, 121.0, 120.4, 120.1, 119.1, 96.5, 50.8, 45.8 (2C), 8.10 (2C). HR-MS: calcd for $C_{21}H_{24}N_3O_2$ $[M+H]^+$: 350.1863; found: 350.1857. Purity was determined by qNMR using maleic acid as an internal standard: 96.2%.

Synthesis of ICTR-060

N,*N*-Diethyl-2-nitroaniline ICTR-048

In a microwave vessel (20 mL), 1-fluoro-2-nitrobenzene (0.27 mL, 2.57 mmol, 1.00 equiv), diethylamine (0.53 mL, 5.14 mmol, 2.00 equiv), and Et_3N (0.71 mL, 5.14 mmol, 2.00 eq.) were dissolved in MeCN (7.0 mL) and stirred under microwave irradiation (120 °C, 300 W) for 2 hours. After complete conversion (according to TLC) and cooling to room temperature, the mixture was taken up in H_2O (50 mL) and extracted with DCM (3 × 50 mL). The combined organic phase was washed with brine (20 mL), dried over $MgSO_4$, filtered, and the solvent was removed under reduced pressure providing 500 mg (2.57 mmol, 100%) of **ICTR-048** in quantitative yield as an orange oil. ^{1}H-NMR (500 MHz, $CDCl_3$, 300 K): δ_H (ppm) = 7.65 (dd, ^3J = 8.0 Hz, ^4J = 1.7 Hz, 1H), 7.41 (ddd, ^3J = 8.6 Hz, 7.2 Hz, ^4J = 1.7 Hz, 1H), 7.15 (d, ^3J = 8.3 Hz, 1H), 6.94 (dd, ^3J = 7.7 Hz, 7.5 Hz, 1H), 3.15 (q, ^3J = 7.2 Hz, 4H), 1.08 (t, ^3J = 7.2 Hz, 6H). ^{13}C-NMR (125 MHz, $CDCl_3$, 300 K): δ_C (ppm) = 144.6, 144.3, 132.6, 125.7, 122.6, 120.6, 46.8 (2C), 12.7. HR-MS: calcd for $C_{10}H_{15}N_2O_2$ $[M+H]^+$: 195.1128; found: 195.1126. Purity was determined by qNMR using maleic acid as an internal standard: 99.1%.

N,*N*-Diethylbenzen-1,2-diamin ICTR-057

According to GWP 4, **ICTR-048** (250 mg, 1.29 mmol, 1.00 equiv), ammonium formate (569 mg, 9.03 mmol, 7.0 equiv), and Pd/C (106 mg, 0.10 mmol, 0.075 equiv, 10% Pd on activated carbon) were used in EtOH (26.0 mL). After a reaction time of 1 hour and complete conversion (according to TLC and staining with Ehrlich's reagent), the mixture was filtered over a Celite pad and washed with MeOH. The solvent was removed under reduced pressure. The residue was redissolved in DCM (30.0 mL), washed with H_2O (10.0 mL), dried over $MgSO_4$, filtered, and the solvent was removed under reduced pressure to give 145 mg (0.88 mmol, 68%) of **ICTR-057** as a violet oil, which was used without any further purification.

(*Z*)-4-({[2-(Diethylamino)phenyl]amino}methylene)isoquinoline-1,3(2*H*,4*H*)-dione ICTR-060

In a microwave vessel (20 mL), **TR-02** (132 mg, 0.65 mmol, 1.00 equiv) and crude **ICTR-057** (140 mg, 0.85 mmol, 1.30 equiv) were suspended in MeOH (5.00 mL) and stirred in the microwave reactor (125 °C, 300 W) for 30 minutes. After cooling to room temperature, the solvent was removed under reduced pressure, and the crude product was adsorbed on silica gel and purified by MPLC (0-5% MeOH in DCM over 20 minutes), yielding 106 mg (0.32 mmol, 49%) of **ICTR-060** as a pale orange solid. mp 211.1 °C. ^{1}H-NMR (500 MHz, DMSO-d_6, 300 K): δ_H (ppm) = 12.87 (d, ^3J = 13.0 Hz, 1H), 11.23 (s, 1H), 8.92 (d, ^3J = 13.0 Hz, 1H), 8.20 (d, ^3J = 8.2 Hz, 1H), 8.03 (dd, ^3J = 7.8 Hz, ^4J = 1.1 Hz, 1H), 7.93 (d, ^3J = 8.2 Hz, 1H), 7.61 (td, ^3J = 7.6 Hz, ^4J = 1.4 Hz, 1H), 7.32 (dd, ^3J = 8.0 Hz, ^4J = 1.1 Hz, 1H), 7.26 (t, ^3J = 7.8 Hz, 1H), 7.24 (t, ^3J = 8.2 Hz, 1H), 7.15 (td, ^3J = 7.6 Hz, ^4J = 1.1 Hz, 1H), 2.96 (q, ^3J = 7.1 Hz, 4H), 0.94 (t, ^3J = 7.1 Hz, 6H). ^{13}C-NMR (125 MHz, DMSO-d_6, 300 K): δ_C (ppm) = 166.3, 163.9, 142.8, 140.0, 136.9, 136.8, 133.1, 127.6, 125.6, 124.4, 124.2, 124.0, 120.9, 120.0, 114.7,

95.7, 48.0 (2C), 12.5 (2C). HR-MS: calcd for $C_{20}H_{22}N_3O_2$ $[M+H]^+$: 336.1707; found: 336.1706. Elemental analysis calcd (%) for $C_{20}H_{21}N_3O_2$: C: 71.62, H: 6.31, N: 12.53; found: C: 71.27, H: 6.23, N: 12.61.

Synthesis of ICTR-059*HCl

N,*N*-Dimethyl-2-(2-nitrophenyl)ethan-1-amine ICTR-044

According to a slightly modified GWP 1 (120 °C, 150 W, 2 hours), 1-(2-bromoethyl)-2-nitrobenzene (500 mg, 2.17 mmol, 1.00 equiv), dimethylamino hydrochloride (195 mg, 2.39 mmol, 1.10 equiv), Et_3N (0.45 mL, 3.26 mmol, 1.50 equiv), and K_2CO_3 (300 mg, 2.17 mmol, 1.00 equiv) were employed. After purification of the crude product by MPLC (0-10% MeOH in DCM over 20 minutes), 40 mg (0.21 mmol, 10%*) of **ICTR-044** were obtained as a brownish oil. ^{1}H-NMR (500 MHz, $CDCl_3$, 300 K): δ_H (ppm) = 8.03 (dd, ^3J = 8.3 Hz, ^4J = 1.4 Hz, 1H), 7.65 (dd, ^3J = 7.7 Hz, ^4J = 1.7 Hz, 1H), 7.62 (ddd, ^3J = 7.7 Hz, 6.9 Hz, ^4J = 1.4 Hz, 1H), 7.46 (ddd, ^3J = 8.4 Hz, 6.8 Hz, ^4J = 1.7 Hz, 1H), 3.54-3.51 (m, 2H), 3.35-3.32 (m, 2H), 2.91 (s, 6H). ^{13}C-NMR (125 MHz, $CDCl_3$, 300 K): δ_C (ppm) = 148.8, 134.6, 133.7, 131.4, 129.1, 125.5, 58.1, 43.0 (2C), 29.3. HR-MS: calcd for $C_{10}H_{15}N_2O_2$ $[M+H]^+$: 195.1128; found: 195.1120.

2-[2-(Dimethylamino)ethyl]aniline ICTR-056

According to GWP 4, **ICTR-044** (250 mg, 1.29 mmol, 1.00 equiv), ammonium formate (569 mg, 9.03 mmol, 7.0 equiv) and Pd/C (106 mg, 0.10 mmol, 0.075 equiv, 10% Pd on activated carbon) in EtOH (26.0 mL) were used. After a reaction time of 1 hour and complete conversion (according to TLC and staining with Ehrlich's reagent), the mixture was filtered over a Celite pad and washed with MeOH. The solvent was removed under reduced pressure, the residue redissolved in DCM (30.0 mL) and washed with H_2O (10.0 mL). The aqueous phase was adjusted to a basic pH (9) with K_2CO_3 and extracted again with DCM. The combined organic phase was dried over $MgSO_4$, filtered, and the solvent was removed under reduced pressure to provide 164 mg (1.00 mmol, 77%) of **ICTR-056** as a reddish-brown oil. This was used in the next reaction without any further purification.

(*Z*)-2-(2-{[(1,3-Dioxo-2,3-dihydroisoquinolin-4(1H)-ylidene)methyl]amino}phenyl)-*N*,*N*-dimethylethan-1-aminium chloride ICTR-059*HCl

In a microwave vessel (20 mL), **TR-02** (152 mg, 0.75 mmol, 1.00 equiv) and crude **ICTR-056** (160 mg, 0.97 mmol, 1.30 equiv) were suspended in MeOH (5.00 mL) and stirred in the microwave reactor (125 °C, 300 W) for 30 minutes. After cooling to room temperature, the solvent was subsequently removed under reduced pressure, and the crude product was adsorbed on silica gel. Purification by MPLC (0-20% MeOH in DCM over 35 minutes) gave rise to **ICTR-059** (209 mg, 0.62 mmol) as a yellow solid in an 83% yield. This was then converted to the corresponding hydrochloride **ICTR-059*HCl** by stirring in 2 M HCl in Et_2O. mp 245.2 °C. ^{1}H-NMR (500 MHz, DMSO-d_6, 300 K): δ_H (ppm) = 12.78 (d, ^3J = 12.0 Hz, 1H), 11.40 (s, 1H), 11.01 (br s, 1H), 8.97 (d, ^3J = 12.0 Hz, 1H), 8.20 (d, ^3J = 8.3 Hz, 1H), 8.04 (dd, ^3J = 7.7 Hz, ^4J = 1.2 Hz, 1H), 7.90 (d, ^3J = 7.7 Hz, 1H), 7.63 (ddd, ^3J = 7.9 Hz, 7.6 Hz, ^4J = 1.4 Hz, 1H), 7.41 (dd, ^3J = 8.3 Hz, 7.7 Hz, 1H), 7.37 (d, ^3J = 7.5 Hz, 1H),

7.29 (t, ^3J = 7.5 Hz, 1H), 7.20 (t, ^3J = 7.5 Hz, 1H), 3.31 – 3.28 (m, 2H), 3.19 – 3.15 (m, 2H), 2.86 (s, 6H). ^{13}C-NMR (125 MHz, DMSO-d_6, 300 K): δ_C (ppm) = 167.3, 163.7, 145.6, 137.6, 136.3, 133.2, 130.6, 128.5, 127.5, 125.8, 124.9, 124.4, 120.9, 120.1, 117.0, 96.1, 55.3, 42.0 (2C), 25.4. HR-MS: calcd for $C_{20}H_{22}N_3O_2$ [M+H]$^+$: 336.1707; found: 336.1714. Elemental analysis calcd (%) for $C_{20}H_{22}ClN_3O_2$ x 1.0 H_2O: C: 61.61, H: 6.20, N: 10.78; found: C: 61.77, H: 6.19, N: 10.67.

(*Z*)-4-{[(2-Aminophenyl)amino]methylene}isoquinoline-1,3(2*H*,4*H*)-dione ICTR-019

In a microwave vessel (20 mL), **TR-02** (250 mg, 1.23 mmol, 1.00 equiv) and *N*,*N*-benzen-1,2-diamine (399 mg, 3.69 mmol, 3.00 equiv) were suspended in MeOH (5.00 mL) and stirred in the microwave reactor (125 °C, 300 W) for 30 minutes. After cooling to room temperature, the precipitate was filtered, washed with MeOH (10 mL) and Et_2O (10 mL), and dried under high vacuum at 50 °C to give **ICTR-019** (330 mg, 1.18 mmol) as a light yellow solid in a yield of 96%. mp 274 °C. ^{1}H-NMR (500 MHz, DMSO-d_6, 300 K): δ_H (ppm) = 12.26 (d, ^3J = 12.6 Hz, 1H), 11.24 (s, 1H), 8.80 (d, ^3J = 12.6 Hz, 1H), 8.11 (d, ^3J = 8.0 Hz, 1H), 8.03 (dd, ^3J = 8.0 Hz, ^4J = 1.1 Hz, 1H), 7.59 (td, ^3J = 7.7 Hz, ^4J = 1.4 Hz, 1H), 7.51 (d, ^3J = 8.0 Hz, 1H), 7.25 (t, ^3J = 7.6 Hz, 1H), 6.98 (ddd, ^3J = 7.7 Hz, 7.4 Hz, ^4J = 1.1 Hz, 1H), 6.88 (dd, ^3J = 8.0 Hz, ^4J = 1.1 Hz, 1H), 6.76 (td, ^3J = 7.6 Hz, ^4J = 1.1 Hz, 1H), 4.98 (br s, 2H). ^{13}C-NMR (125 MHz, DMSO-d_6, 300 K): δ_C (ppm) = 166.6, 163.8, 146.2, 139.3, 136.9, 133.1, 127.5, 127.2, 125.7, 123.8, 120.7, 119.7, 118.4, 118.2, 117.2, 95.2. HR-MS: calcd for $C_{16}H_{14}N_3O_2$ [M+H]$^+$: 280.1081; found: 280.1079. Purity was determined by qNMR using maleic acid as an internal standard: 100%.

(*Z*)-4-({[2-(Dimethylamino)phenyl]amino}methylene)isoquinoline-1,3(2*H*,4*H*)-dione ICTR-015

In a microwave vessel (20 mL), **TR-02** (250 mg, 1.23 mmol, 1.00 equiv) and *N*,*N*-dimethylbenzen-1,2-diamine (0.25 mL, 1.85 mmol, 1.50 equiv) were suspended in MeOH (5.00 mL) and stirred in the microwave reactor (125 °C, 300 W) for 30 minutes. After cooling to room temperature, the precipitate was filtered, washed with MeOH (10 mL) and Et_2O (10 mL), and dried under high vacuum at 50 °C to afford **ICTR-015** (313 mg, 1.02 mmol) as a light yellow solid in a yield of 82%. mp 222 °C. ^{1}H-NMR (500 MHz, DMSO-d_6, 300 K): δ_H (ppm) = 12.71 (d, ^3J = 13.0 Hz, 1H), 11.22 (s, 1H), 8.95 (d, ^3J = 13.0 Hz, 1H), 8.19 (d, ^3J = 8.0 Hz, 1H), 8.04 (dd, ^3J = 8.0 Hz, ^4J = 1.4 Hz, 1H), 7.87 (dd, ^3J = 7.8 Hz, ^4J = 1.4 Hz, 1H), 7.62 (td, ^3J = 7.7 Hz, ^4J = 1.4 Hz, 1H), 7.27 (dd, ^3J = 7.8 Hz, 7.7 Hz, 1H), 7.26 (d, ^3J = 7.3 Hz, 1H), 7.17 (td, ^3J = 7.6 Hz, ^4J = 1.4 Hz, 1H), 7.13 (td, ^3J = 7.3 Hz, ^4J = 1.5 Hz, 1H), 2.68 (s, 6H). ^{13}C-NMR (125 MHz, DMSO-d_6, 300 K): δ_C (ppm) = 166.5, 163.8, 143.4, 143.3, 136.7, 133.7, 133.1, 127.5, 124.6, 124.4, 124.0, 120.9, 120.4, 120.0, 115.3, 95.7, 44.1 (2C). HR-MS: calcd for $C_{18}H_{18}N_3O_2$ [M+H]$^+$: 308.1394; found: 308.1393. Purity was determined by qNMR using maleic acid as an internal standard: 100%.

(*Z*)-4-({[2-(2-Hydroxyethyl)phenyl]amino}methylene)isoquinoline-1,3(2*H*,4*H*)-dione ICTR-053

In a microwave vessel (20 mL), **TR-02** (329 mg, 1.62 mmol, 1.00 equiv) and 2-(2-aminophenyl)ethan-1-ol (0.28 mL, 2.11 mmol, 1.30 equiv) were suspended in MeOH (5.00 mL) and stirred in the microwave reactor (125 °C,

300 W) for 30 minutes. After cooling to room temperature, the precipitate was filtered, washed with MeOH (10 mL) and Et_2O (10 mL), and dried under high vacuum at 50 °C to obtain **ICTR-053** (461 mg, 1.50 mmol) as a yellow solid in a yield of 93%. mp 252 °C. ^{1}H-NMR (500 MHz, DMSO-d_6, 300 K): δ_H (ppm) = 12.72 (d, ^{3}J = 12.4 Hz, 1H), 11.29 (br s, 1H), 8.87 (d, ^{3}J = 12.4 Hz, 1H), 8.15 (d, ^{3}J = 8.2 Hz, 1H), 8.04 (dd, ^{3}J = 7.8 Hz, ^{4}J = 1.2 Hz, 1H), 7.78 (d, ^{3}J = 7.8 Hz, 1H), 7.61 (ddd, ^{3}J = 8.4 Hz, 7.0 Hz, ^{4}J = 1.4 Hz, 1H), 7.33 (td, ^{3}J = 7.7 Hz, ^{4}J = 1.7 Hz, 1H), 7.30 (dd, ^{3}J = 7.9 Hz, ^{4}J = 1.7 Hz, 1H), 7.27 (td, ^{3}J = 7.7 Hz, ^{4}J = 1.1 Hz, 1H), 7.15 (td, ^{3}J = 7.3 Hz, ^{4}J = 1.1 Hz, 1H), 4.81 (t, ^{3}J = 4.8 Hz, 1H), 3.71 (dq, ^{3}J = 6.6 Hz, 2H), 2.85 (t, ^{3}J = 6.6 Hz, 2H). ^{13}C-NMR (125 MHz, DMSO-d_6, 300 K): δ_C (ppm) = 166.8, 163.8, 145.8, 138.1, 136.6, 133.1, 130.8, 129.2, 127.5 (2C), 124.7, 124.1, 120.9, 119.9, 117.0, 95.6, 60.5, 34.2. HR-MS: calcd for $C_{18}H_{16}N_2O_3Na_1$ $[M+Na]^+$: 331.1053; found: 331.1057. Elemental analysis calcd (%) for $C_{18}H_{16}N_2O_3$: C: 70.12, H: 5.23, N: 9.09; found: C: 70.19, H: 5.28, N: 9.17.

Series IV: Planar, rigid Ring System

Since a further substitution at the pyridine nitrogen in **1** would lead to the introduction of a permanent charge (**2**), we decided on the commercially available 6-(5*H*)-phenanthridinone (**3**) as the new core structure, where further substituents can be introduced at the 2-position via the corresponding bromide derivative using a SUZUKI coupling.

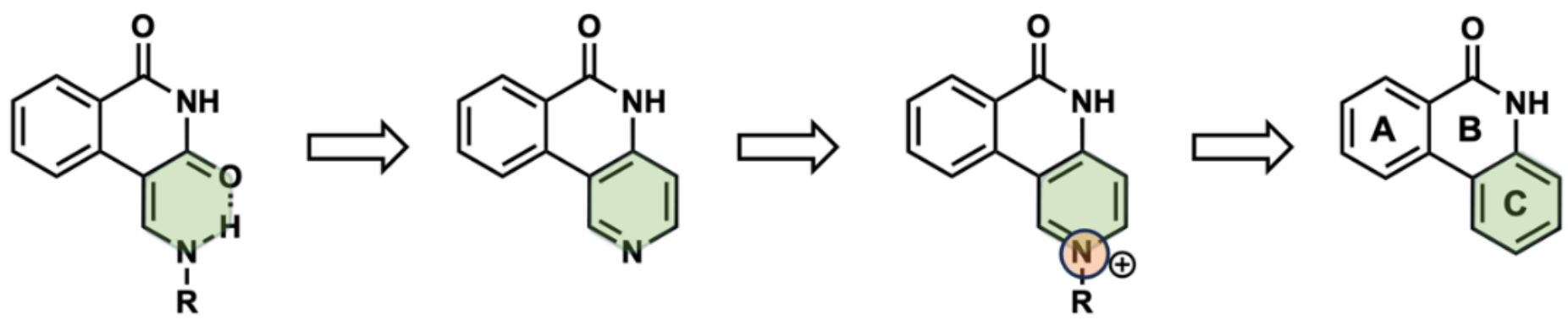

1 2 3

2-Bromphenanthridin-6(5*H*)-one TR-027

Following a published procedure, 6-(5*H*)-phenanthridinone was brominated with Br_2 in pure AcOH under reflux, yielding **TR-027** in good yield.[5,6]

To a suspension of 6-(5*H*)-phenanthridinone (500 mg, 2.56 mmol, 1.00 equiv) in AcOH (4.0 mL), a solution of bromine (0.14 mL, 2.82 mmol, 1.10 equiv) in AcOH (2.0 mL) was added dropwise and stirred under reflux for 3 hours. After cooling to room temperature, the resulting precipitate was filtered, washed with H_2O (2 × 5 mL) and 10% $Na_2S_2O_3$ (aq., 2 × 3 mL), and dried under high vacuum at 50 °C to obtain **TR-027** (563 mg, 2.05 mmol) as a white solid in an 80% yield. mp 324 °C. ^{1}H-NMR (300 MHz, DMSO-d_6, 300 K): δ_H (ppm) = 11.83 (s, 1H), 8.58 (d, ^4J = 2.1 Hz, 1H), 8.56 (d, ^3J = 8.6 Hz, 1H), 8.31 (dd, ^3J = 7.9 Hz, ^4J = 1.3 Hz, 1H), 7.85 (td, ^3J = 7.7 Hz, 7.5 Hz, ^4J = 1.1 Hz; 1H), 7.67 (t, ^3J = 7.2 Hz, 1H), 7.64 (dd, ^3J = 8.6 Hz, ^4J = 2.0 Hz, 1H), 7.32 (d, ^3J = 8.6 Hz, 1H). HR-MS: calcd for $C_{13}H_9Br^{79}{}_1N_1O_1$/$C_{13}H_9Br^{81}{}_1N_1O_1$ $[M+H]^+$: 273.9862/275.9842; found: 273.9858/275.9838.

2-{3-[(Dimethylamino)methyl]phenyl}phenanthridin-6(5*H*)-one TR-033

In a preheated round-bottom flask, **TR-027** (490 mg, 1.79 mmol, 1.00 equiv), {3-[(dimethylamino)methyl]phenyl}boronic acid hydrochloride (500 mg, 2.32 mmol, 1.30 equiv), and K_2CO_3 (742 mg, 5.37 mmol, 3.00 equiv) were suspended in a mixture of dioxane/H_2O (4:1) under argon. Then, tetrakis(triphenylphosphine)palladium(0) (104 mg, 5 mol%) was added, and the solvent was degassed again with argon. The mixture was heated to 100 °C and stirred under reflux until the reaction was complete, as indicated by TLC. After cooling to room temperature, the reaction mixture was filtered over a Celite pad, washed with MeOH (3 × 15 mL) and the solvent was removed under reduced pressure. The crude product was adsorbed on silica gel and purified by MPLC (5-15% MeOH in DCM over 20 CV), yielding 141 mg (0.43 mmol, 24%) of **TR-033** as a pale brown solid. mp 198.1 °C. ^{1}H-NMR (400 MHz, DMSO-d_6, 300 K): δ_H (ppm) = 11.75 (s, 1H), 8.70 (d, ^3J = 8.2 Hz, 1H), 8.61 (d, ^4J = 1.8 Hz, 1H), 8.35 (dd, ^3J = 8.0 Hz, ^4J = 1.4 Hz, 1H), 7.87 (ddd, ^3J = 7.6

Hz, ^4J = 1.4 Hz, 1H), 7.80 (dd, ^3J = 8.5 Hz, ^4J = 1.8 Hz, 1H), 7.71 (s, 1H), 7.70-7.69 (m, 1H), 7.66 (dd, ^3J = 8.0 Hz, 7.4 Hz, 1H), 7.46 (d, ^3J = 8.5 Hz, 1H), 7.43 (dd, ^3J = 8.2 Hz, 7.6 Hz, 1H), 7.29 (d, ^3J = 7.6 Hz, 1H), 3.49 (s, 2H), 2.19 (s, 6H). ^{13}C-NMR (100 MHz, DMSO-*d6*): δ_C (ppm) = 160.7, 139.6, 139.5, 135.9, 134.3, 134.2, 132.7, 128.7, 128.2, 128.0, 127.6, 127.4, 127.0, 125.8, 125.4, 123.0, 121.0, 117.9, 116.7, 63.4, 44.9 (2C). HR-MS: calcd for $C_{22}H_{21}N_2O_1$ [M+H]$^+$: 329.1648; found: 329.1639. Purity was determined by qNMR using maleic acid as an internal standard: 93.7%.

2-{3-[(Diethylamino)methyl]phenyl}phenanthridin-6(5*H*)-one ICTR-096

In a preheated round-bottom flask, **TR-027** (255 mg, 0.93 mmol, 1.00 equiv), {3-[(diethylamino)methyl]phenyl}boronic acid (250 mg, 1.21 mmol, 1.30 equiv), and K_2CO_3 (385 mg, 2.79 mmol, 3.00 equiv) were suspended in a mixture of dioxane/H_2O (4:1) under argon. Then, tetrakis(triphenylphosphine)palladium(0) (54 mg, 5 mol%) was added, and the solvent was degassed again with argon. The mixture was heated to 100 °C and stirred under reflux until the reaction was complete, as indicated by TLC. After cooling to room temperature, the reaction mixture was diluted with EtOAc (10.0 mL) and washed with brine (10.0 mL). The aqueous phase was extracted with EtOAc (3 × 15.0 mL). The combined organic phase was washed with brine (10.0 mL), dried over $MgSO_4$, filtered, and the solvent was removed under reduced pressure. The crude product was adsorbed on silica gel and purified by MPLC (0-10% MeOH in DCM over 25 minutes), yielding 266 mg (0.75 mmol, 80%) of **ICTR-096** as a yellow solid. mp 171,3 °C. ^{1}H-NMR (500 MHz, DMSO-*d6*, 300 K): δ_H (ppm) = 11.74 (s, 1H), 8.69 (d, ^3J = 8.0 Hz), 8.61 (d, ^4J = 2.0 Hz, 1H), 8.35 (dd, ^3J = 8.0 Hz, ^4J = 1.2 Hz, 1H), 7.88 (ddd, ^3J = 8.4 Hz, 7.1 Hz, ^4J = 1.4 Hz, 1H), 7.79 (dd, ^3J = 8.6 Hz, ^4J = 2.0 Hz, 1H), 7.71 (s, 1H), 7.67 (d, ^3J = 8.6 Hz, 1H), 7.67 (td, ^3J = 7.5 Hz, ^4J = 1.2 Hz, 1H), 7.46 (d, ^3J = 8.0 Hz, 1H), 7.43 (t, ^3J = 7.5 Hz, 1H), 7.33 (d, ^3J = 7.5 Hz, 1H), 3.63 (s, 2H), 2.52 (m, 4H), 1.01 (t, ^3J = 7.0 Hz, 6H). ^{13}C-NMR (125 MHz, DMSO-*d6*): δ_C (ppm) = 160.7, 140.7, 139.5, 135.9, 134.4, 134.2, 132.7, 128.6, 128.2, 128.0, 127.4, 127.3, 126.8, 125.8, 125.1, 122.9, 121.0, 117.9, 116.7, 57.0, 46.1 (2C), 11.6 (2C). HR-MS: calcd for $C_{24}H_{25}N_2O_1$ [M+H]$^+$: 357.1961; found: 357.1657. Elemental analysis calcd (%) for $C_{24}H_{24}N_2O_1$ x 1.25 H_2O: C: 76.06, H: 7.05, N: 7.39; found: C: 75.83, H: 6.98, N: 7.58. Purity was determined by qNMR using maleic acid as an internal standard: 99.7%.

2-{2-[(Diethylamino)methyl]phenyl}phenanthridin-6(5*H*)-one ICTR-094

In a preheated round-bottom flask, **TR-027** (250 mg, 0.91 mmol, 1.00 equiv), *N*-ethyl-*N*-[2-(4,4,5,5-tetramethyl-1,3,2-dioxaborolan-2-yl)benzyl]ethanamine (367 mg, 1.27 mmol, 1.40 equiv), and K_2CO_3 (377 mg, 2.73 mmol, 3.00 equiv) were suspended under argon in a mixture of dioxane/water (4:1). Tetrakis(triphenylphosphine)palladium(0) (53 mg, 5 mol%) was then added, and the solvent was degassed again with argon. The mixture was heated to 100 °C and stirred under reflux until the reaction was complete, as indicated by TLC. After cooling to room temperature, the reaction mixture was diluted with EtOAc (10.0 mL) and washed with brine (10.0 mL). The aqueous phase was extracted with EtOAc (3 × 15.0 mL). The combined

organic phase was washed with brine (10.0 mL), dried over $MgSO_4$, filtered, and the solvent was removed under reduced pressure. The crude product was adsorbed on silica gel and purified by MPLC (0-10% MeOH in DCM over 25 minutes), yielding 232 mg (0.65 mmol, 72%) of **ICTR-094** as a yellow solid. mp 210 °C. ^{1}H-NMR (500 MHz, DMSO-*d*$_6$, 300 K): δ_H (ppm) = 11.73 (s, 1H), 8.53 (d, ^3J = 8.0 Hz, 1H), 8.40 (d, ^4J = 1.7 Hz, 1H), 8.34 (dd, ^3J = 8.0 Hz, ^4J = 1.2 Hz, 1H), 7.83 (ddd, ^3J = 8.4 Hz, 7.1 Hz, ^4J = 1.4 Hz, 1H), 7.65 (td, ^3J = 7.6 Hz, ^4J = 1.2 Hz, 1H), 7.58 (d, ^3J = 7.5 Hz, 1H), 7.49 (dd, ^3J = 8.3 Hz, ^4J = 1.7 Hz, 1H), 7.41 (d, ^3J = 8.3 Hz, 1H), 7.37 (t, ^3J = 7.6 Hz, 1H), 7.33 (t, ^3J = 7.5 Hz, 1H), 7.30 (dd, ^3J = 7.5 Hz, ^4J = 1.4 Hz, 1H), 3.49 (s, 2H), 2.36 (q, ^3J = 7.0 Hz, 4H), 0.83 (t, ^3J = 7.0 Hz, 6H). ^{13}C-NMR (125 MHz, DMSO-*d*$_6$, 300 K): δ_C (ppm) = 160.7, 141.4, 137.3, 135.5, 134.9, 134.3, 132.7, 130.6, 130.0, 129.7, 127.9, 127.4, 127.0, 126.5, 125.8, 123.7, 122.7, 117.1, 115.6, 54.6, 45.9 (2C), 11.4 (2C). HR-MS: calcd for $C_{24}H_{25}N_2O_1$ [M+H]$^+$: 357.1961; found: 357.1956. Elemental analysis calcd (%) for $C_{24}H_{24}N_2O_1$ x 0.25 H_2O: C: 79.86, H: 6.84, N: 7.76; found: C: 80.20, H: 7.07, N: 7.74. Purity was determined by qNMR using maleic acid as an internal standard: 95.1%.

References

[1] O.S. Wolfbeis, I. Trummer, A. Knierzinger, 2-Substituierte pyrano[2,3-c]isochinolin-3,6-dione und merocyaninfarbstoffe aus homophthalsäureimiden, *Liebigs. Ann. Chem.* (**1981**) 811–818. https://doi.org/10.1002/JLAC.198119810506.

[2] J. Kankanala, C. Marchand, M. Abdelmalak, H. Aihara, Y. Pommier, Z. Wang, Isoquinoline-1,3-diones as selective inhibitors of tyrosyl DNA phosphodiesterase II (TDP2), *J. Med. Chem.* 59 (**2016**) 2734–2746. https://doi.org/10.1021/ACS.JMEDCHEM.5B01973

[3] D.K. Kölmel, A. Hörner, F. Rönicke, M. Nieger, U. Schepers, S. Bräse, Cell-penetrating peptoids: introduction of novel cationic side chains, *Eur. J. Med. Chem.* 79 (**2014**) 231–243. https://doi.org/10.1016/j.ejmech.2014.03.078.

[4] W.L. Mosby, 2-Bromophenanthridone, *J. Am. Chem. Soc.* 76 (**1954**) 936–937. https://doi.org/10.1021/JA01632A103.

[5] H. Gilman, J. Eisch, The bromination of phenanthridine, *J. Am. Chem. Soc.* 77 (**1955**) 6379–6380. https://doi.org/10.1021/JA01628A097.

[6] Albrecht & Fischer. Vinylamine - VI: Vinylamine aus Pyridylketon und primären Aminine. Tetrahedron 26, 4777–4782 (1970).

HPLC traces of compounds TR-07*HCl, TR-033, TR-036*HCl, and ICTR-059*HCl

Figure S 14: HPLC trace of TR-07*HCl

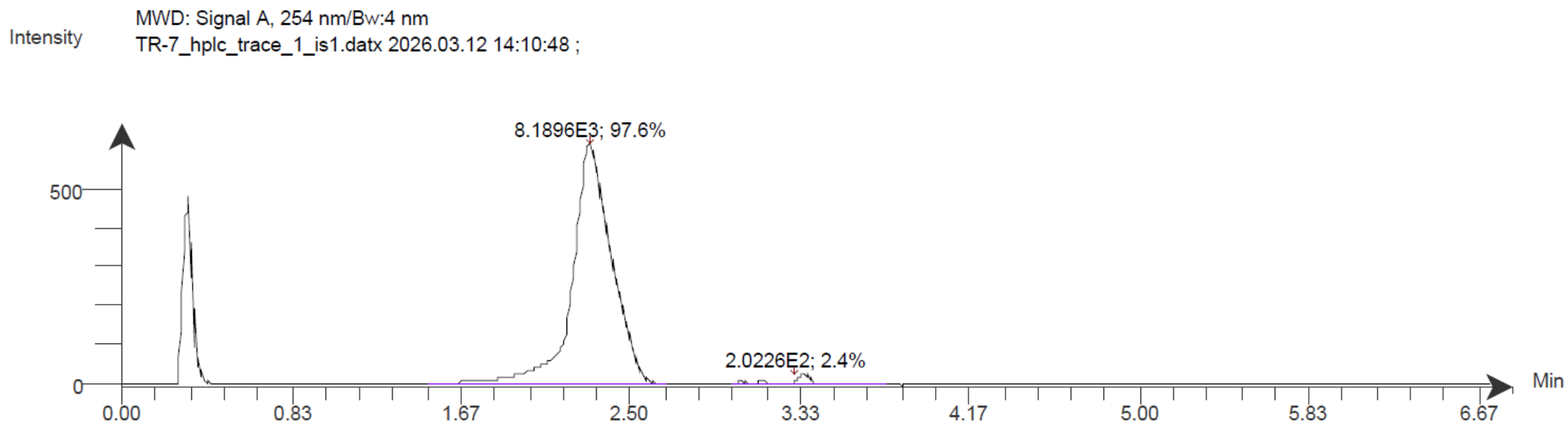

Time (Peak Maximum M:S/Minutes)	Maximum Intensity (c/s)	Time (Peak Centroid M:S/Minutes)	Peak Area	% Peak Area	Peak Resolution	Label
2.30	6.2E2	2.30	8.1896E3	97.6	11.4	
3.35	2.8E1	3.31	2.0226E2	2.4	3.7	

Figure S 15: HPLC trace of TR-033

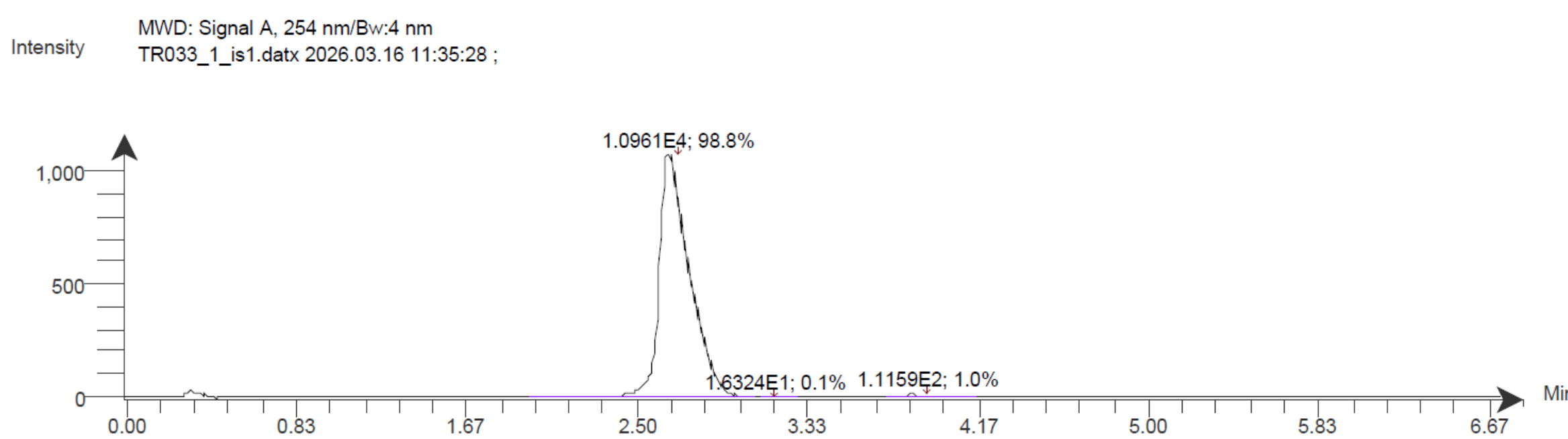

Time (Peak Maximum M:S/Minutes)	Maximum Intensity (c/s)	Time (Peak Centroid M:S/Minutes)	Peak Area	% Peak Area	Peak Resolution	Label
2.65	1.1E3	2.69	1.0961E4	98.8	9.0	
3.16	3.5E0	3.17	1.6324E1	0.1	5.8	
3.84	1.2E1	3.91	1.1159E2	1.0	7.3	

Figure S 16: HPLC trace of TR-036*HCl

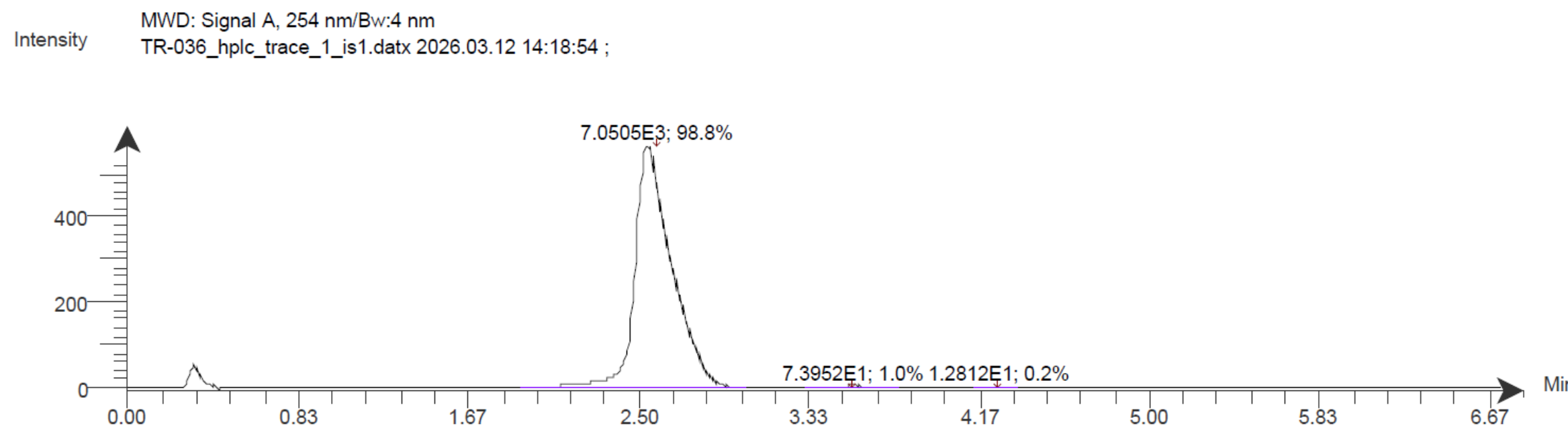

Time (Peak Maximum M:S/Minutes)	Maximum Intensity (c/s)	Time (Peak Centroid M:S/Minutes)	Peak Area	% Peak Area	Peak Resolution	Label
2.54	5.7E2	2.58	7.0505E3	98.8	10.8	
3.55	6.1E0	3.54	7.3952E1	1.0	15.4	
0.00	6.7E0	4.25	1.2812E1	0.2	13.2	

Figure S 17: HPLC trace of ICTR-059*HCl

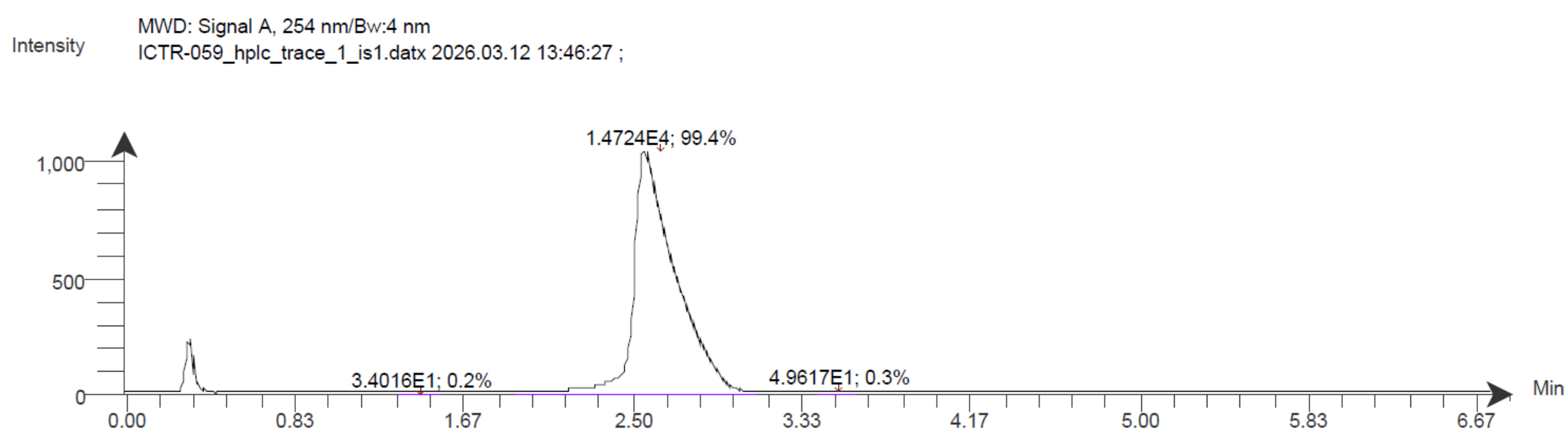

Time (Peak Maximum M:S/Minutes)	Maximum Intensity (c/s)	Time (Peak Centroid M:S/Minutes)	Peak Area	% Peak Area	Peak Resolution	Label
1.48	3E0	1.45	3.4016E1	0.2	28.0	
2.56	1E3	2.63	1.4724E4	99.4	12.0	
3.52	1.1E1	3.51	4.9617E1	0.3	4.6	

Figure S 18: ^{1}H-NMR spectra (500 MHz) of TR-01 in DMSO-d_6

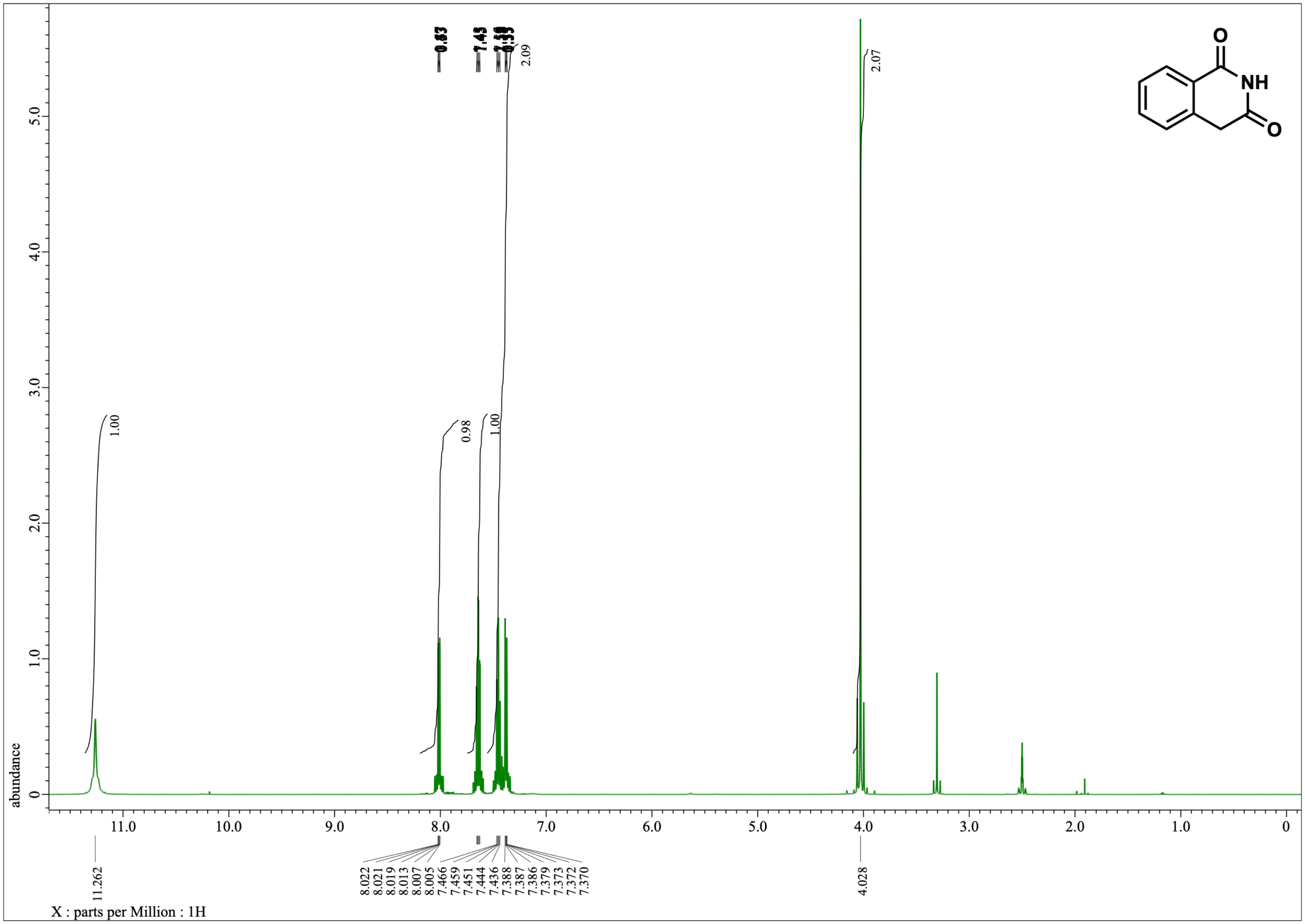

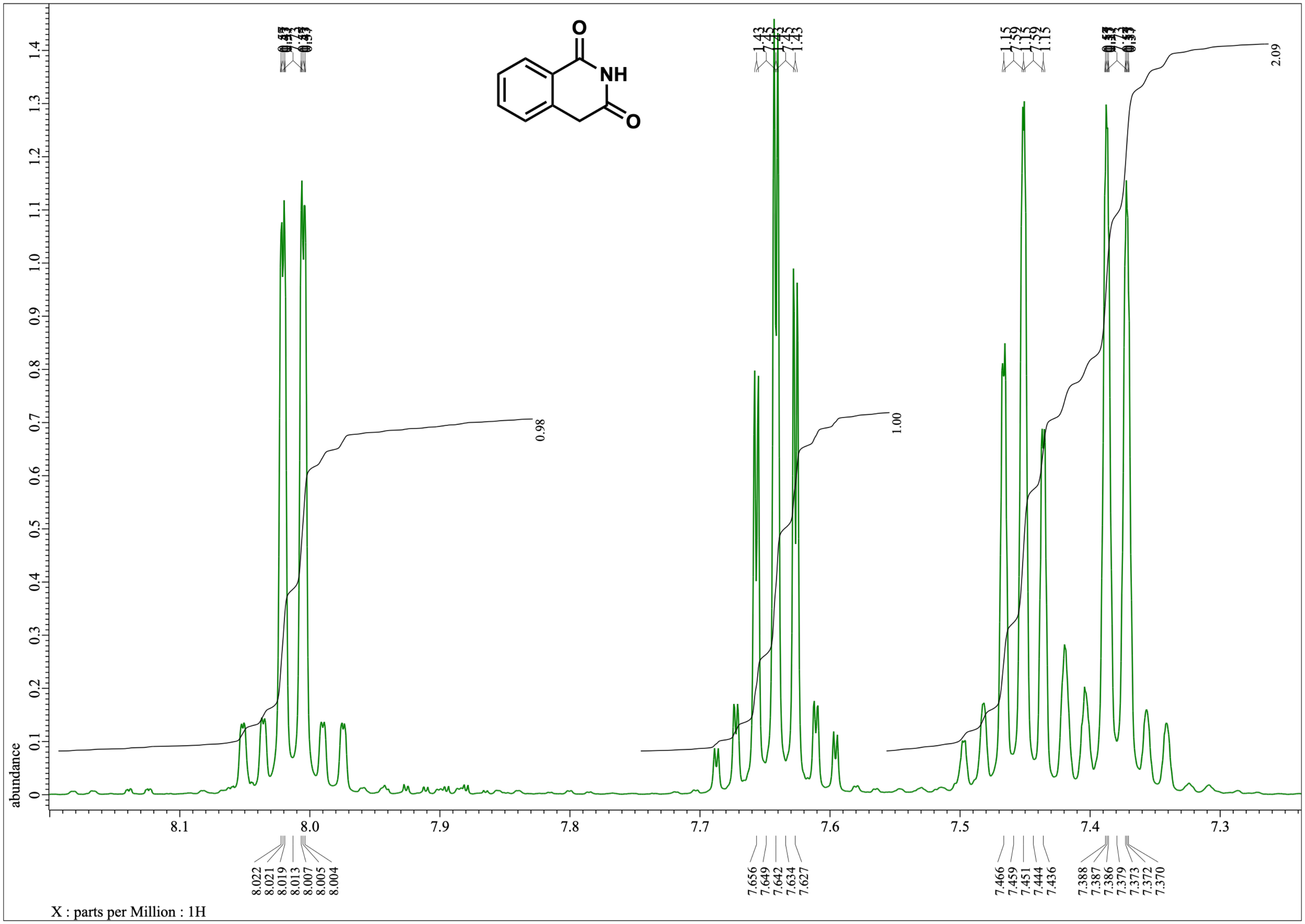
abundance
X : parts per Million : 1H
0.98
1.00
2.09
8.022
8.021
8.019
8.013
8.007
8.005
8.004
7.656
7.649
7.642
7.634
7.627
7.466
7.459
7.451
7.444
7.436
7.388
7.387
7.386
7.379
7.373
7.372
7.370

Figure S 19: ^{13}C-NMR (APT) spectra (125 MHz) of TR-01 in DMSO-d_6

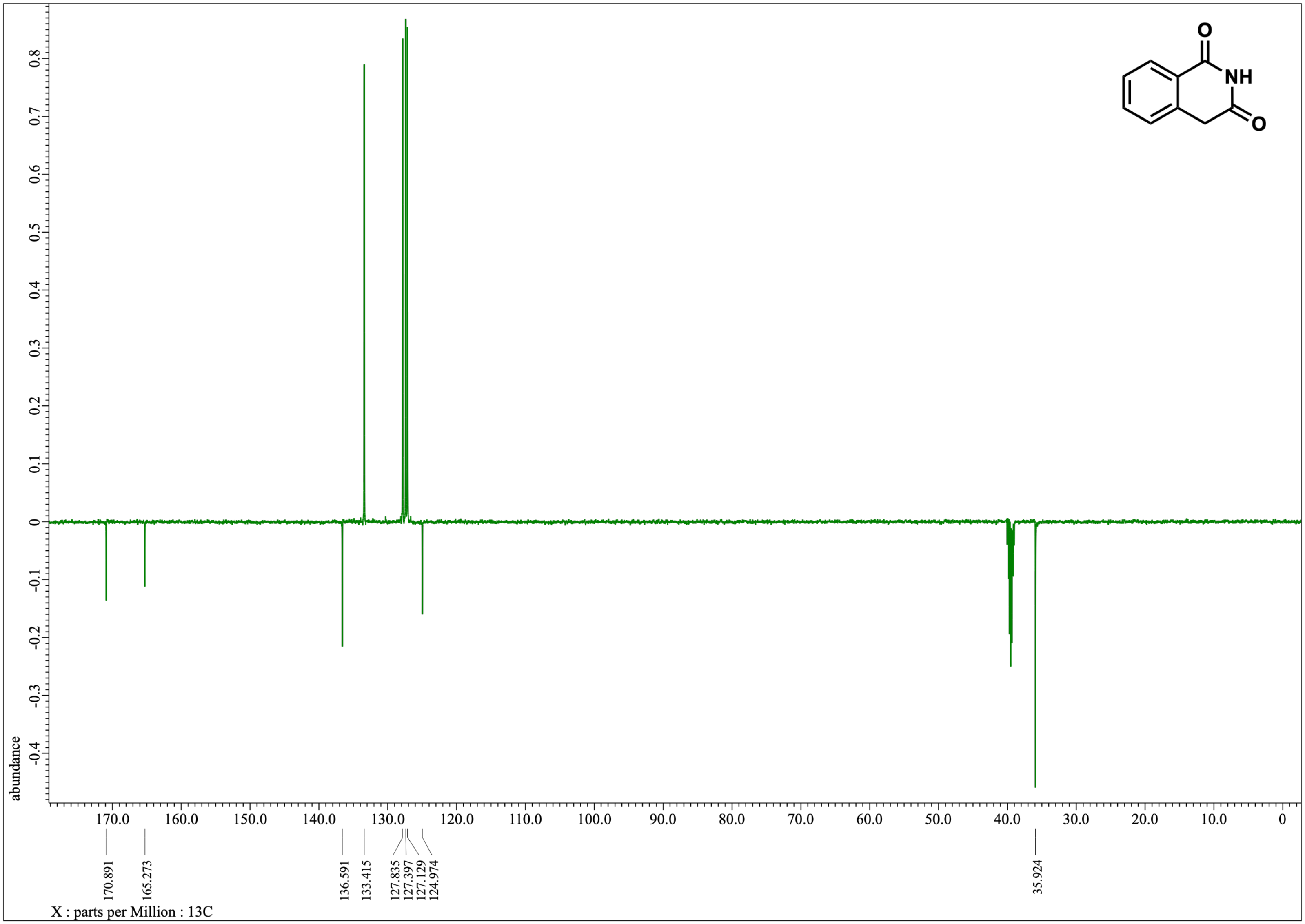

Figure S 20: ^{1}H-NMR spectra (500 MHz) of TR-02 in DMSO-*d*₆

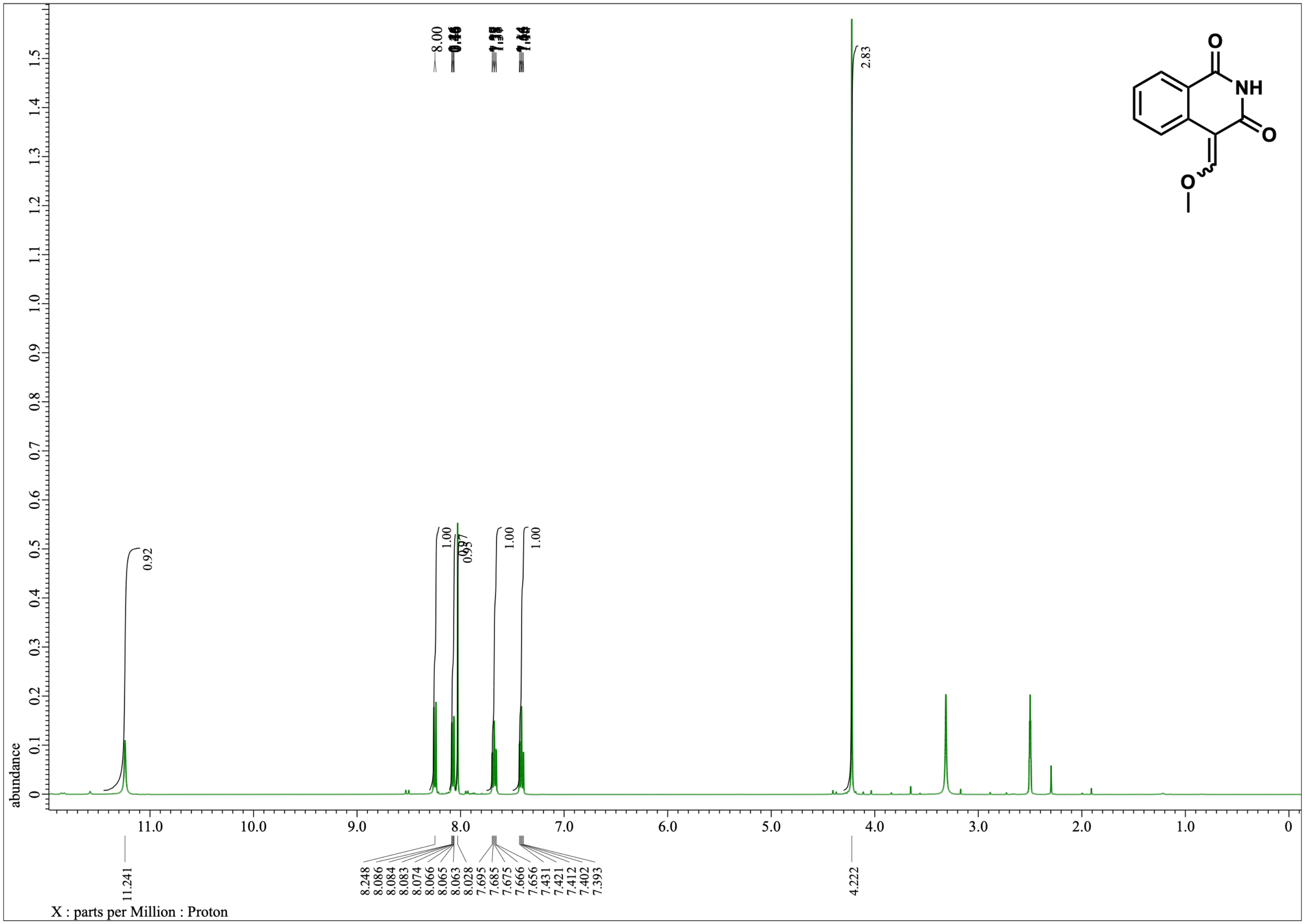

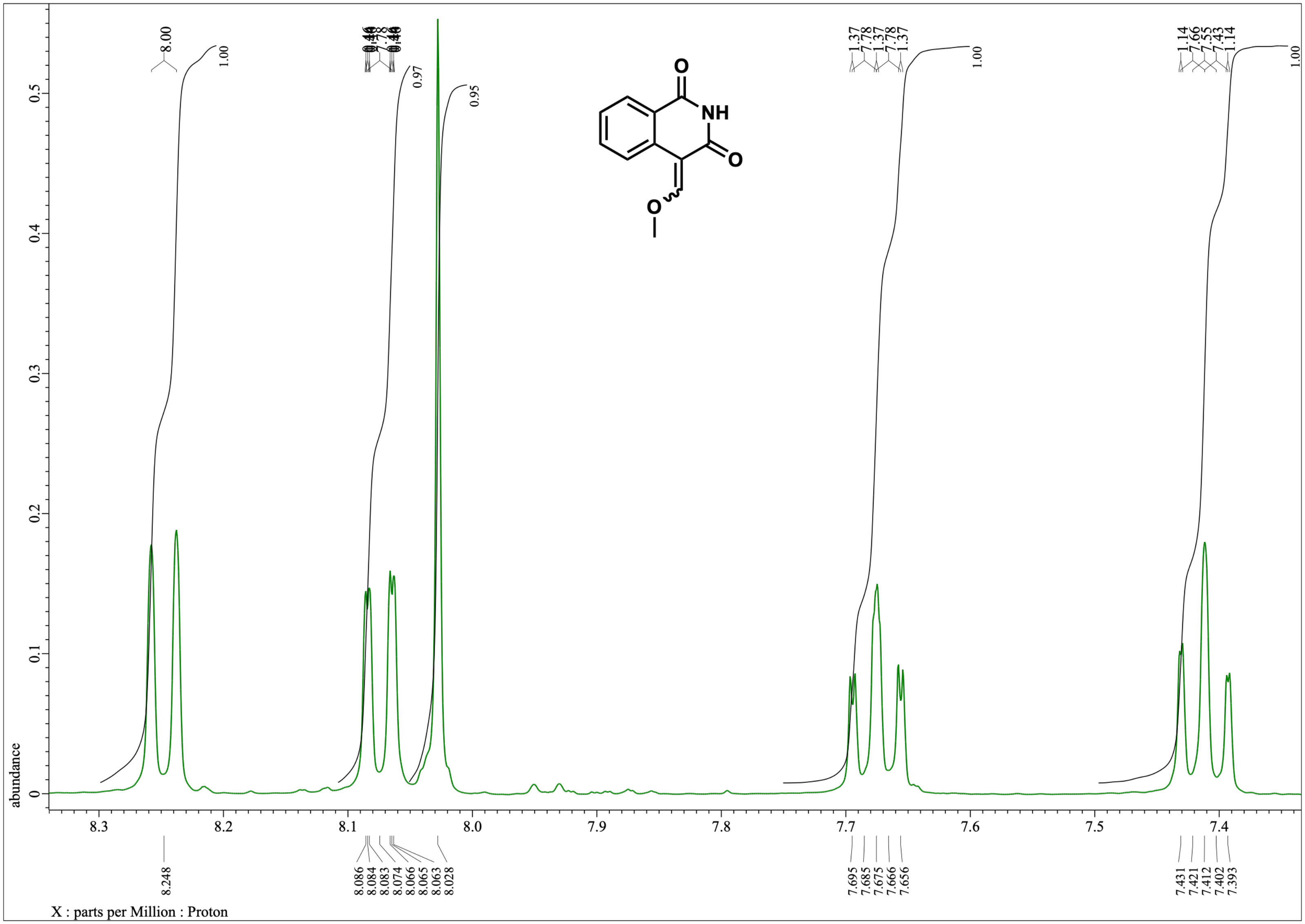
NH
O
O
O
abundance
X : parts per Million : Proton
8.248
8.086
8.084
8.083
8.074
8.066
8.065
8.063
8.028
7.695
7.685
7.675
7.666
7.656
7.431
7.421
7.412
7.402
7.393
1.00
0.97
0.95
1.00
1.00

Figure S 21: ^{13}C-NMR (APT) spectra (125 MHz) of TR-02 in DMSO-d_6

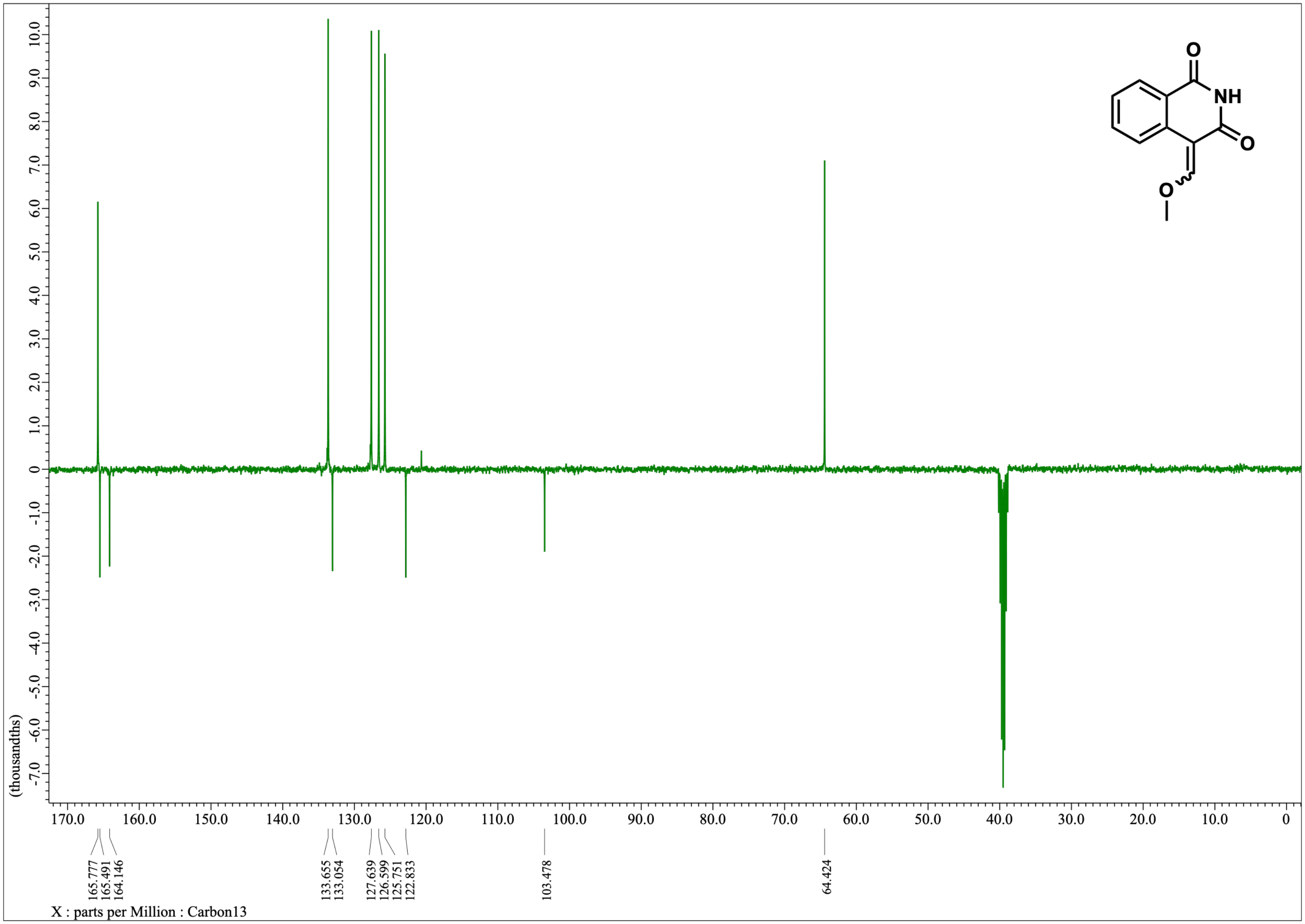

Figure S 22: ^{1}H-NMR spectra (500 MHz) of TR-03 in DMSO-d_6

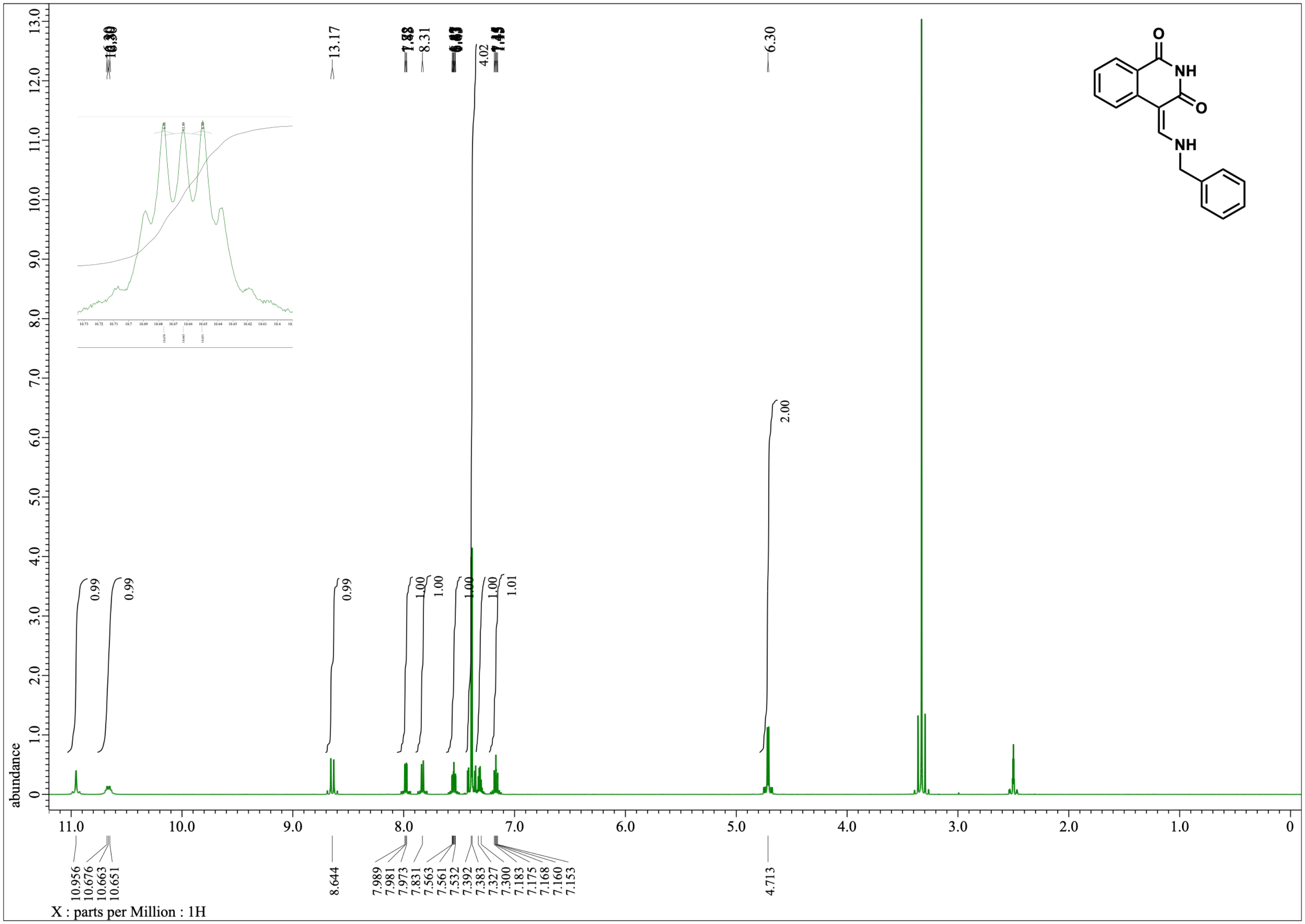

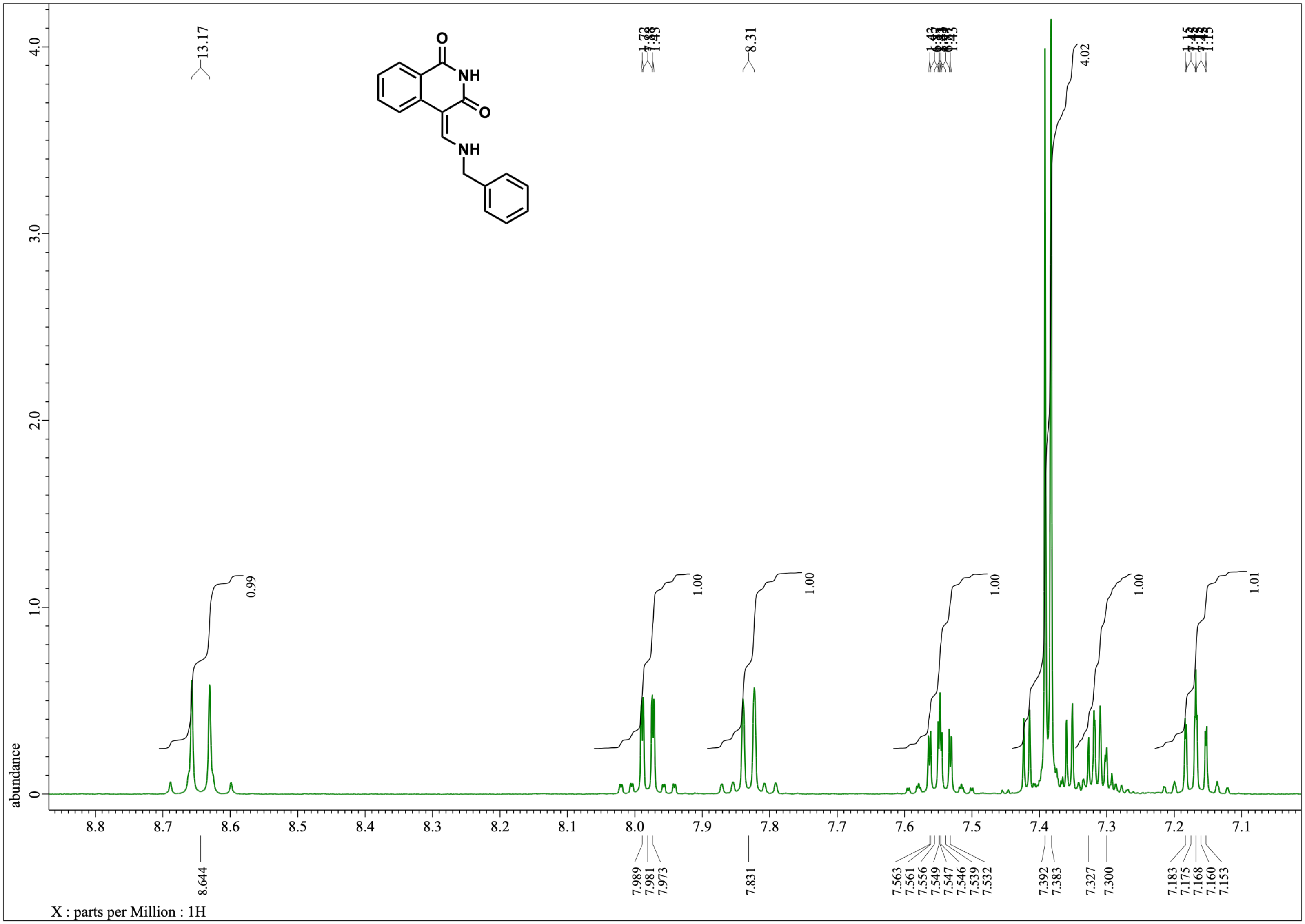

NH
NH
O
O
13.17
8.31
4.02
0.99
1.00
1.00
1.00
1.00
1.01
abundance
0
1.0
2.0
3.0
4.0
8.8 8.7 8.6 8.5 8.4 8.3 8.2 8.1 8.0 7.9 7.8 7.7 7.6 7.5 7.4 7.3 7.2 7.1
8.644
7.989
7.981
7.973
7.831
7.563
7.561
7.556
7.549
7.547
7.546
7.539
7.532
7.392
7.383
7.327
7.300
7.183
7.175
7.168
7.160
7.153
X : parts per Million : 1H

Figure S 23: ^{13}C-NMR (APT) spectra (125 MHz) of TR-03 in DMSO-d_6

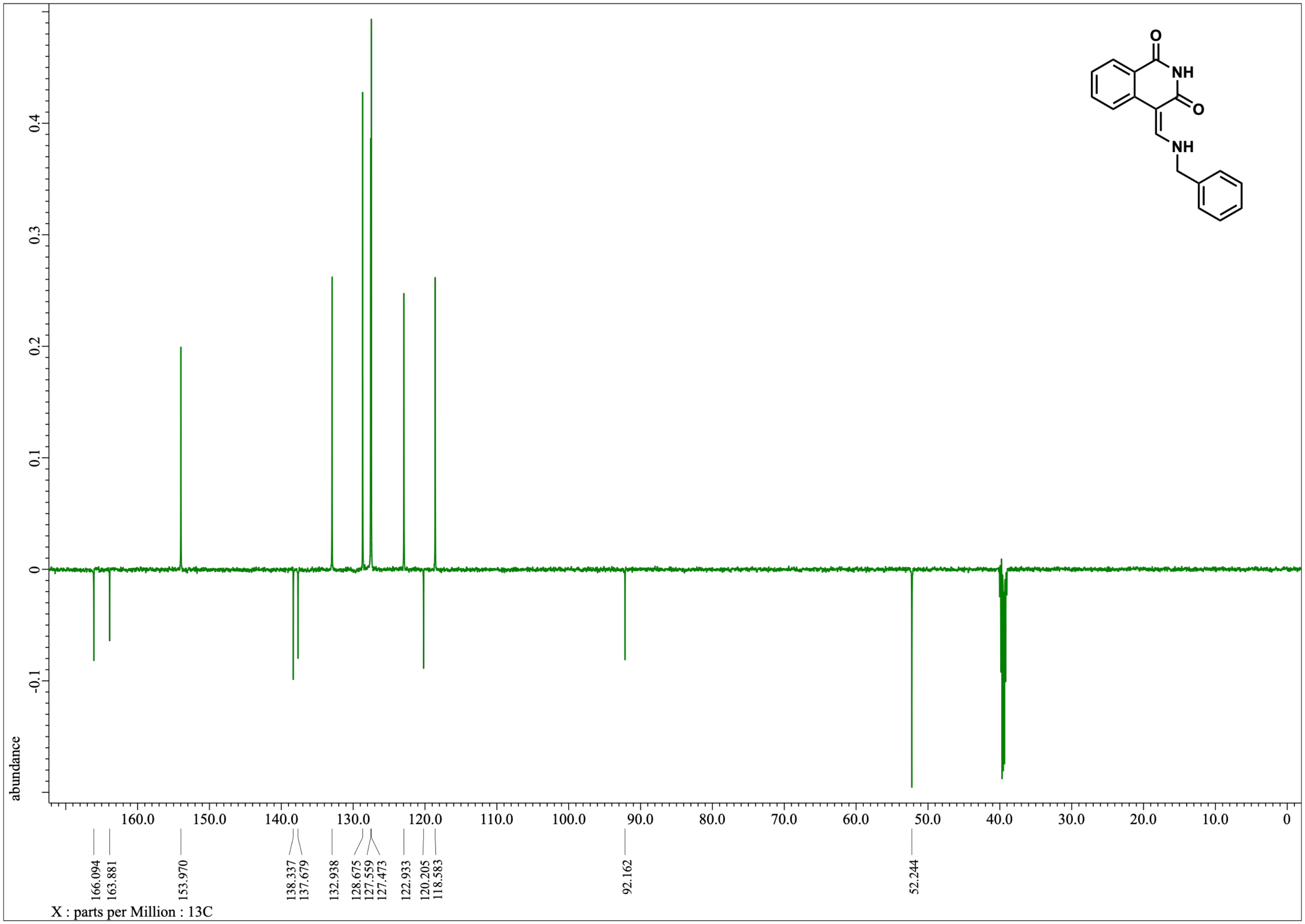

Figure S 24: ^{1}H-NMR spectra (500 MHz) of TR-07*HCl in DMSO-*d₆*

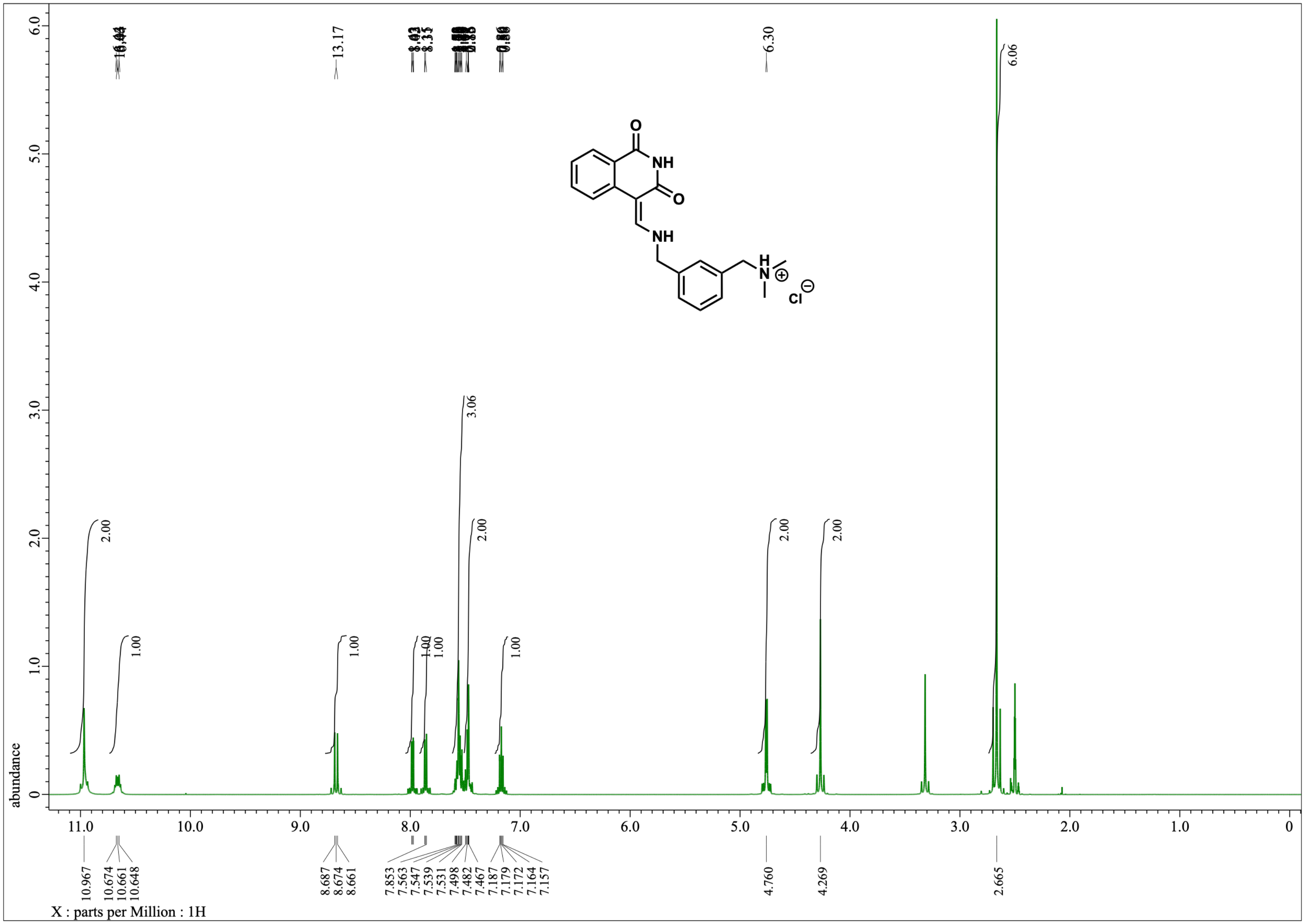

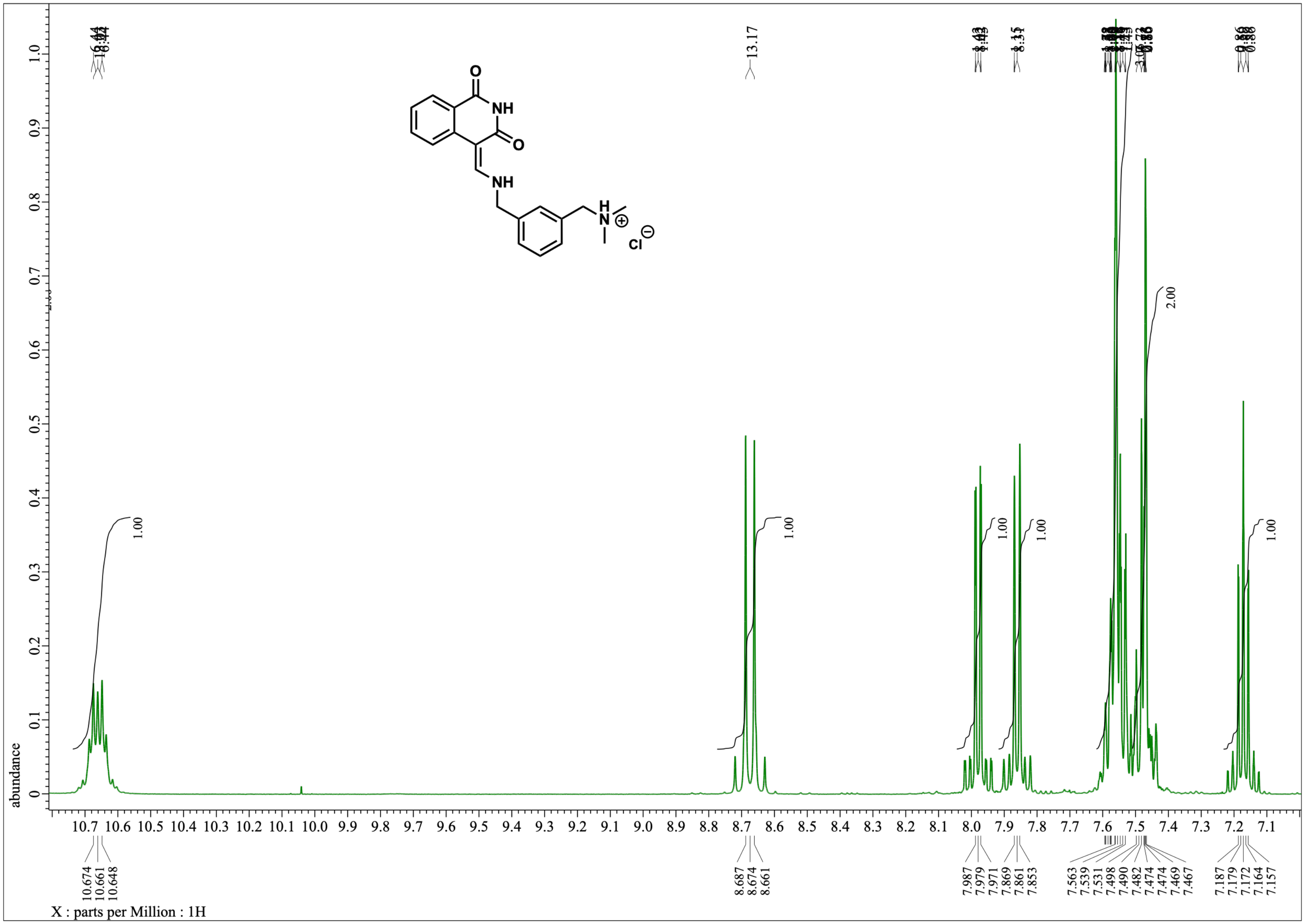
X : parts per Million : 1H
abundance

Figure S 25: ^{13}C-NMR (APT) spectra (125 MHz) of TR-07*HCl in DMSO-d_6

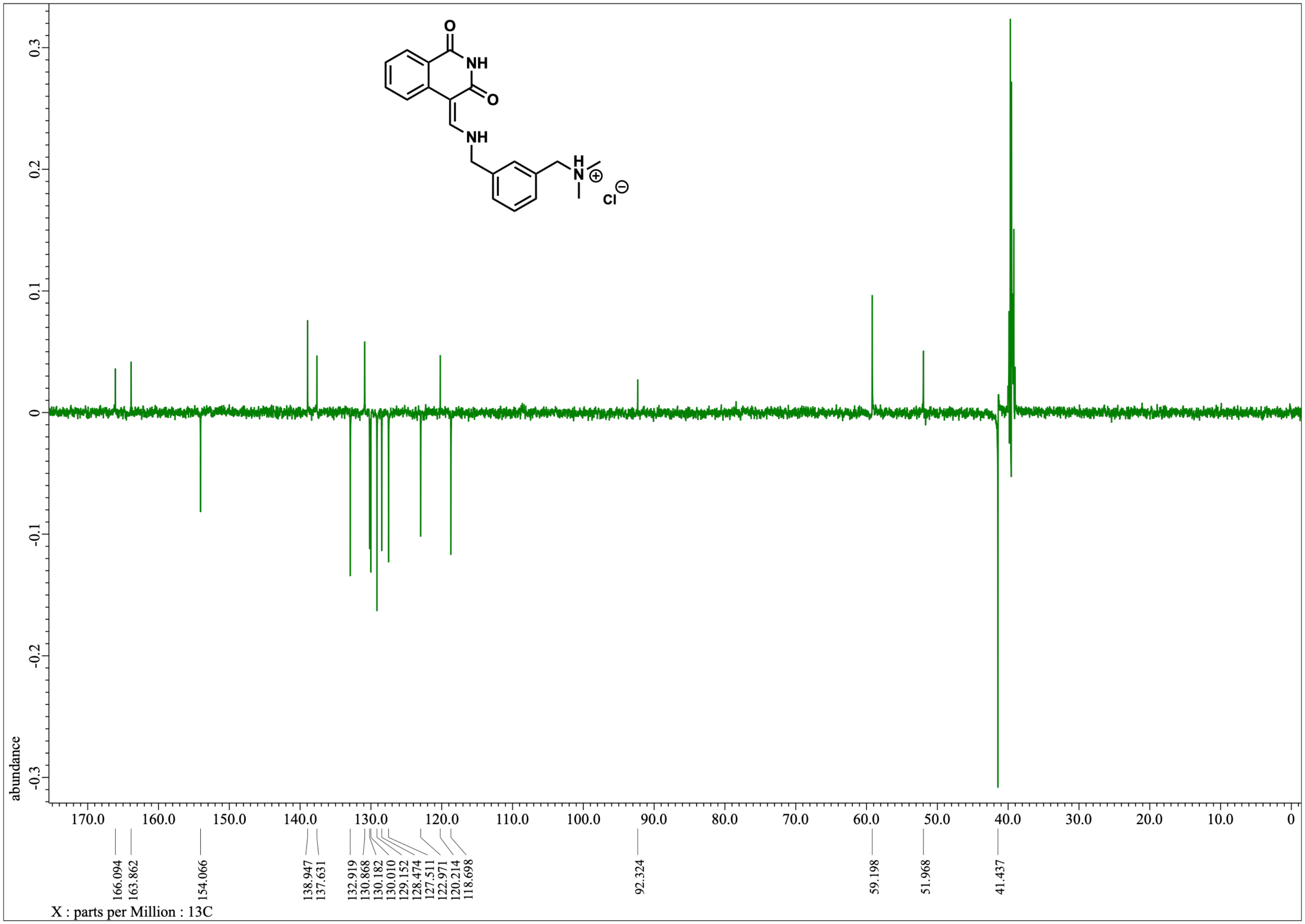

Figure S 26: HMBC spectra of TR-07*HCl in DMSO-d_6

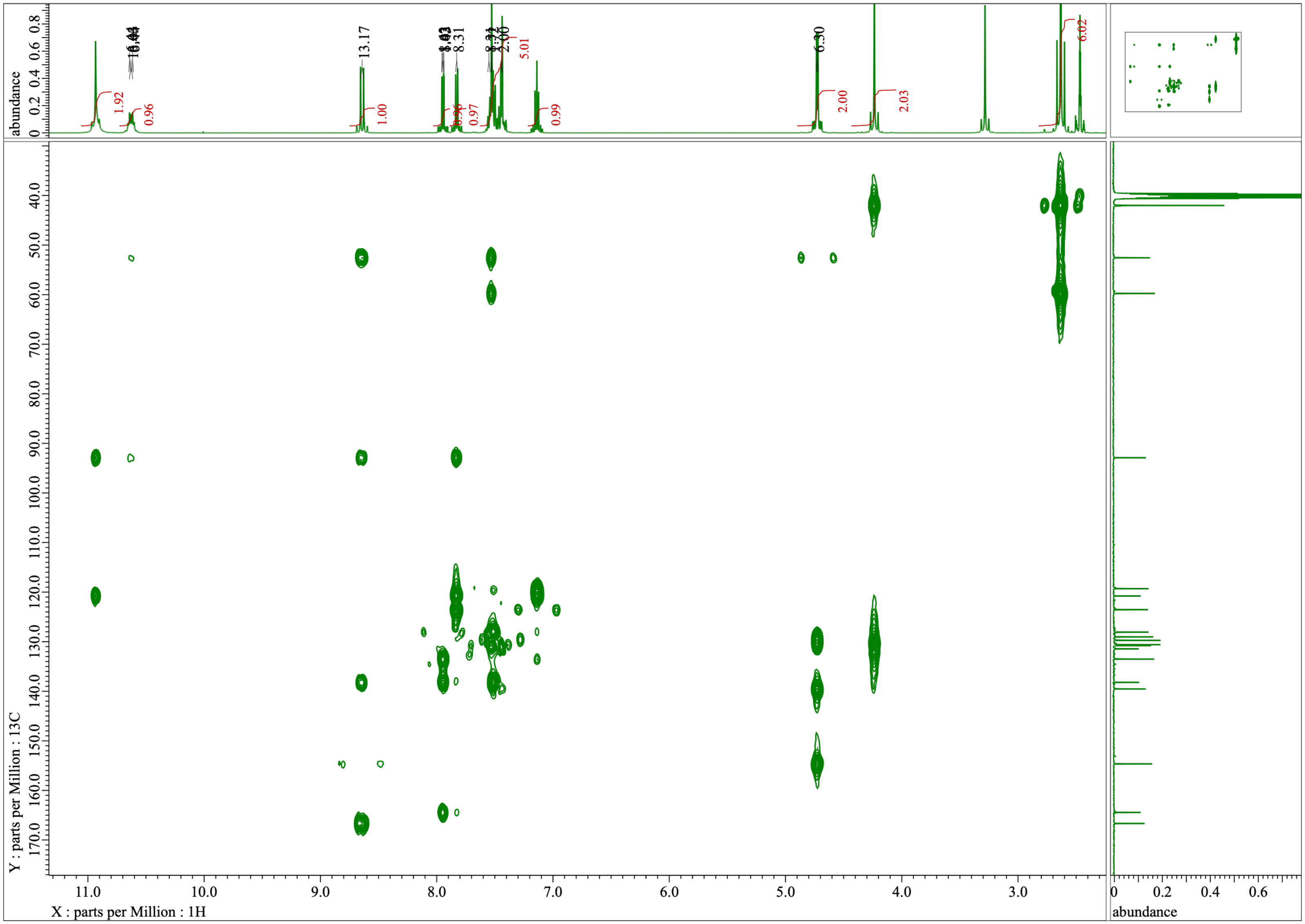

Figure S 27: ^{1}H-NMR spectra (500 MHz) of TR-010 in DMSO-*d₆*

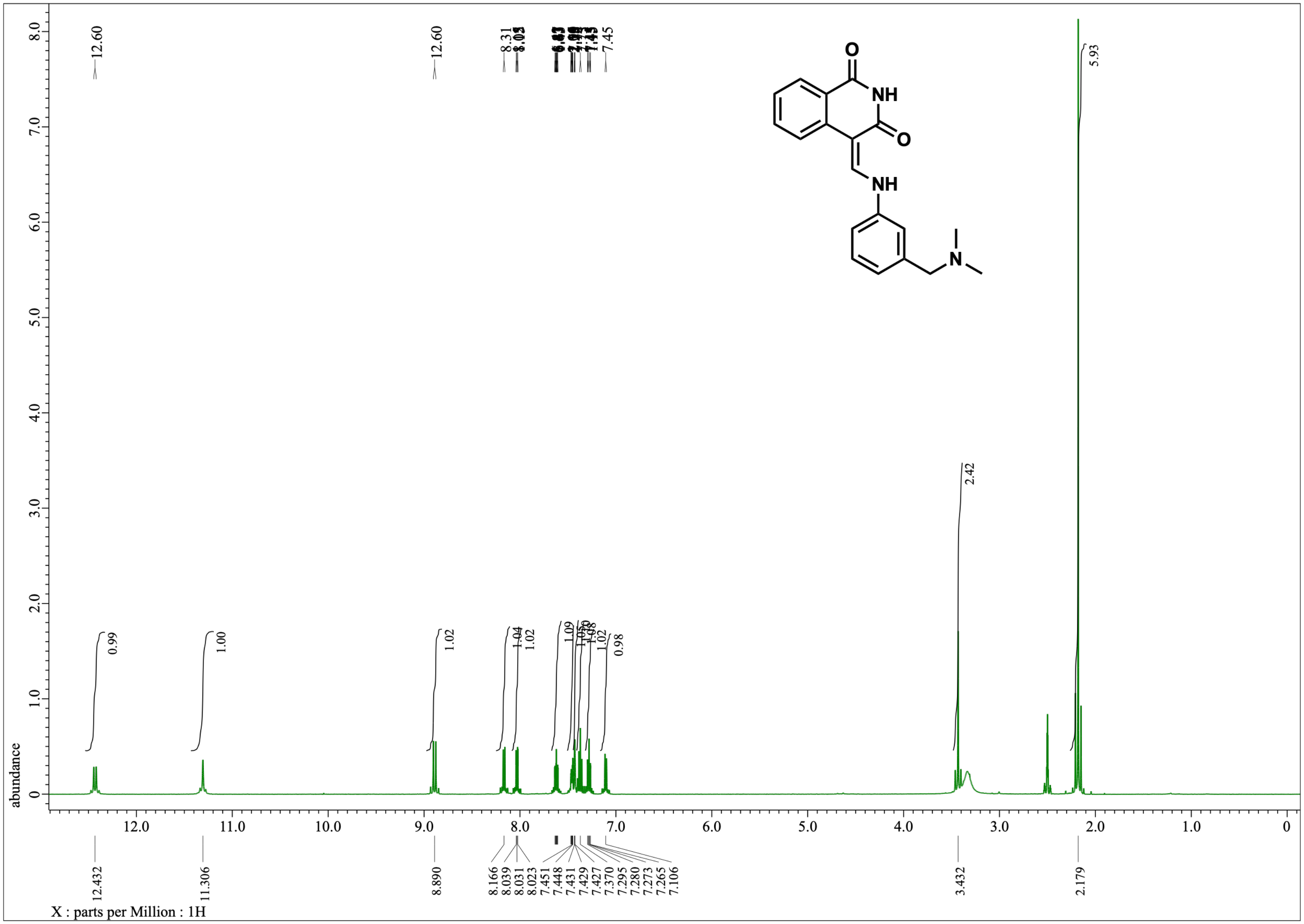

Figure S 28: ^{13}C-NMR (APT) spectra (100 MHz) of TR-010 in DMSO-d_6

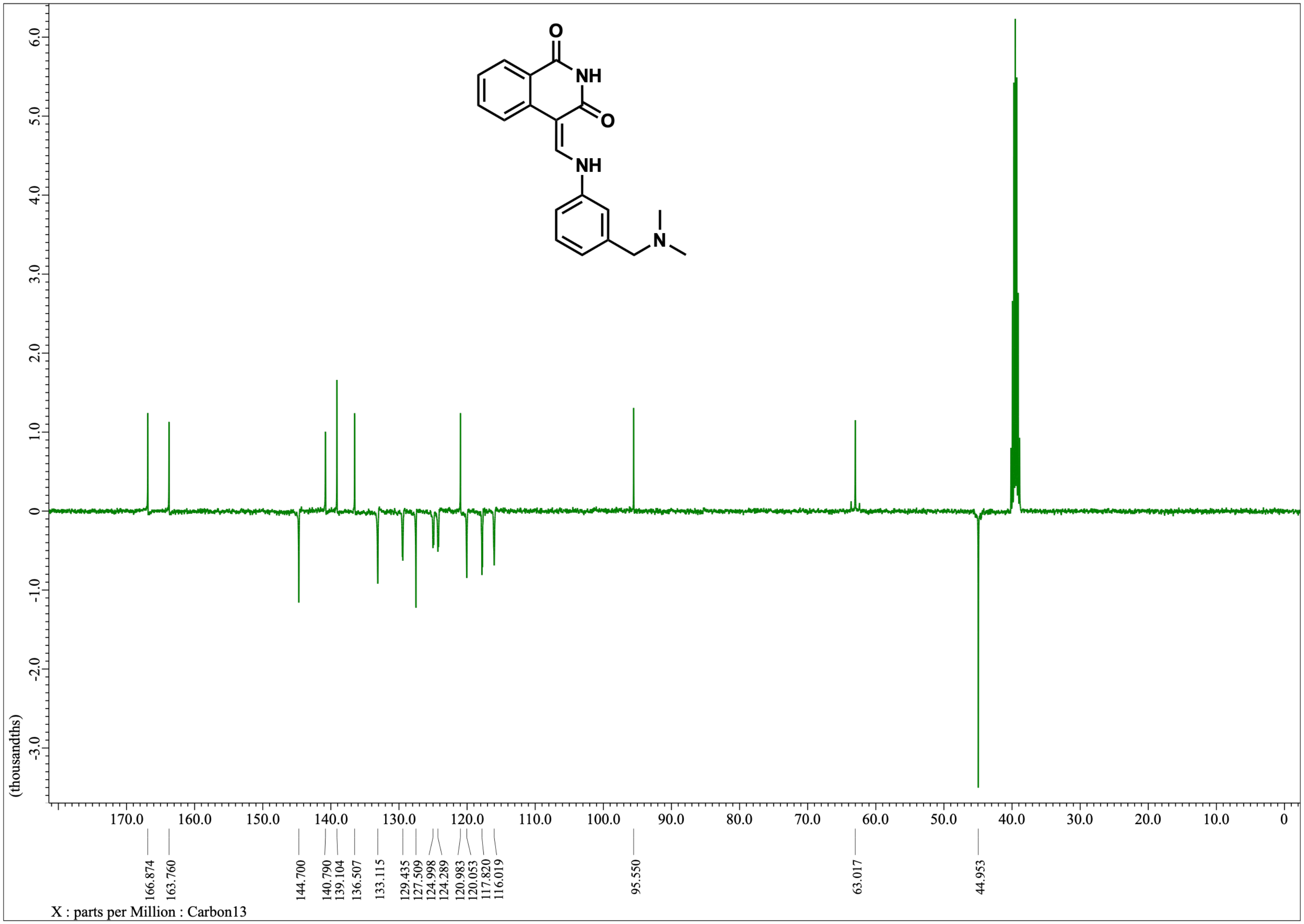

Figure S 29: ^{1}H-NMR spectra (500 MHz) of TR-014 in DMSO-d_6

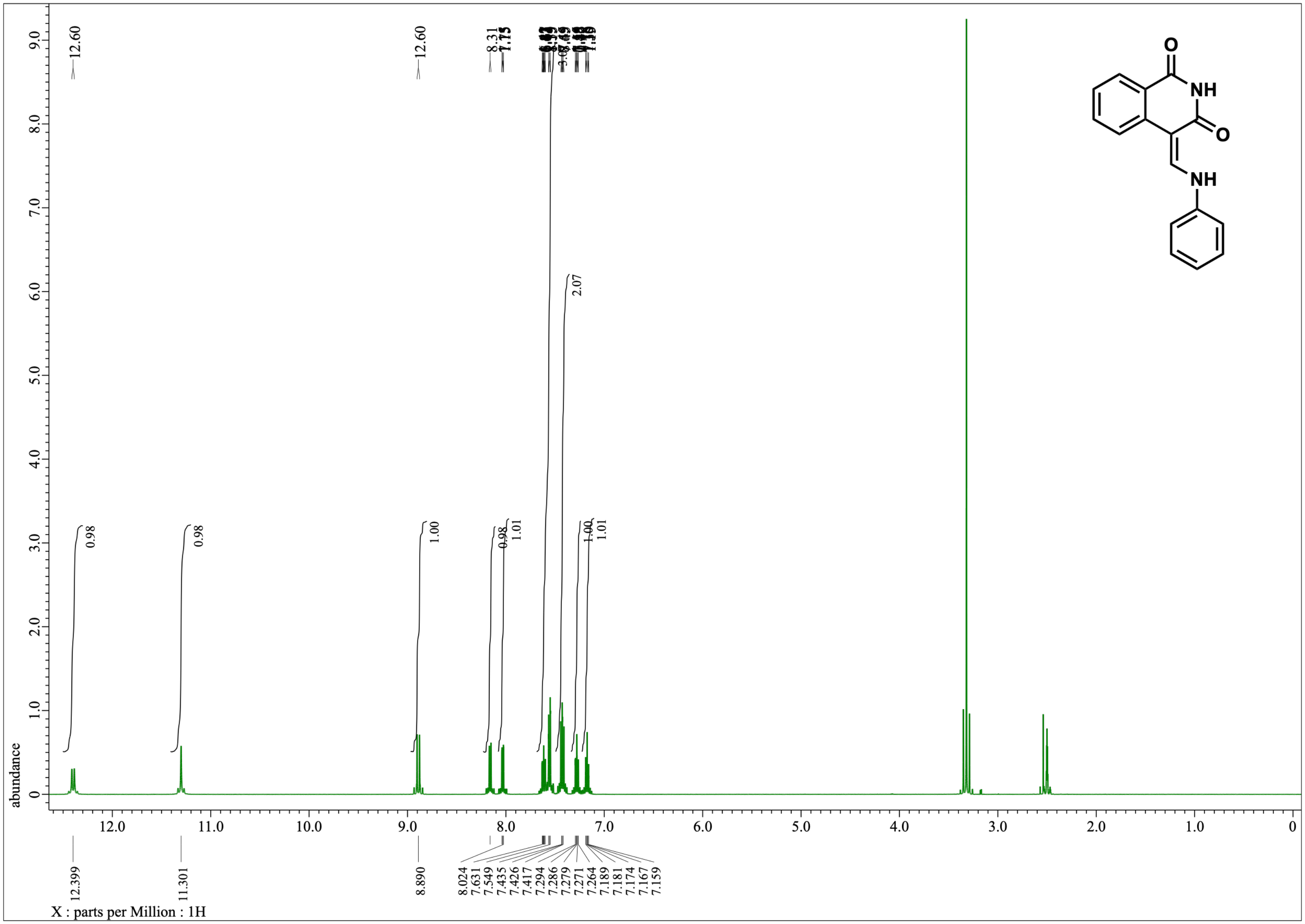

Figure S 30: ^{13}C-NMR (APT) spectra (100 MHz) of TR-014 in DMSO-d_6

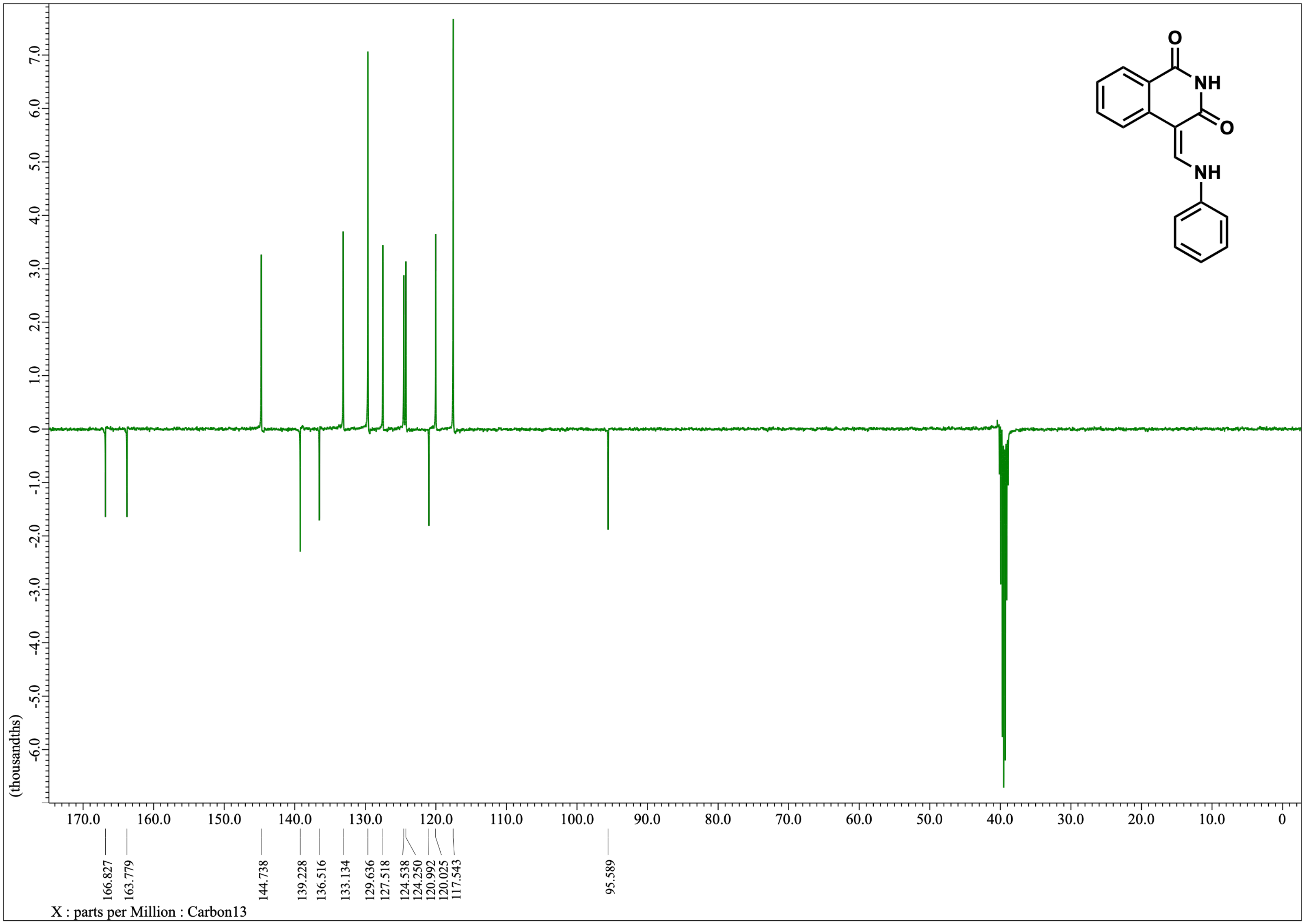

Figure S 31: 1D-NOESY-NMR spectra (500 MHz) of TR-014 in DMSO-d_6

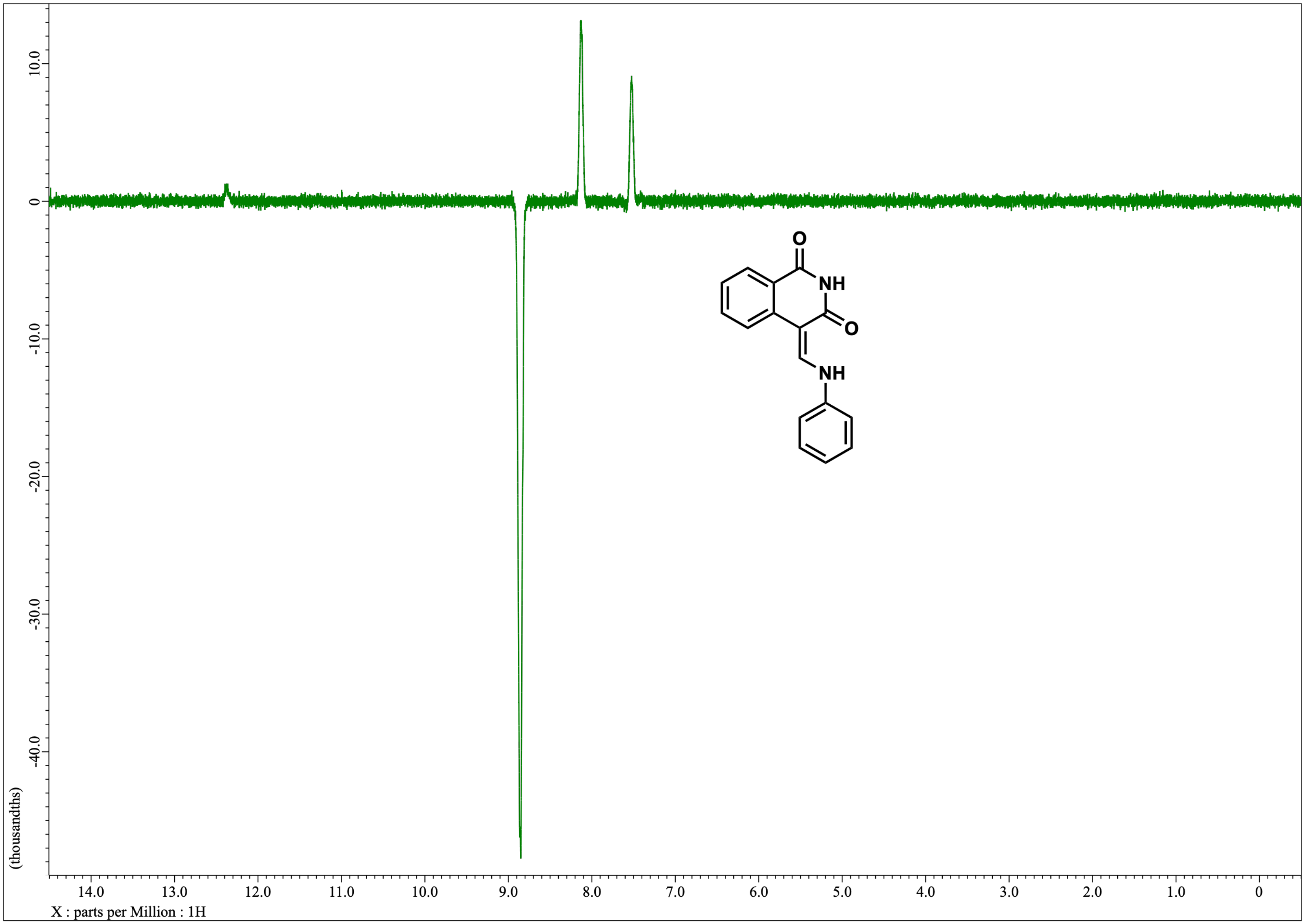

Figure S 32: ^{1}H-NMR spectra (500 MHz) of TR-018 in DMSO-*d₆*

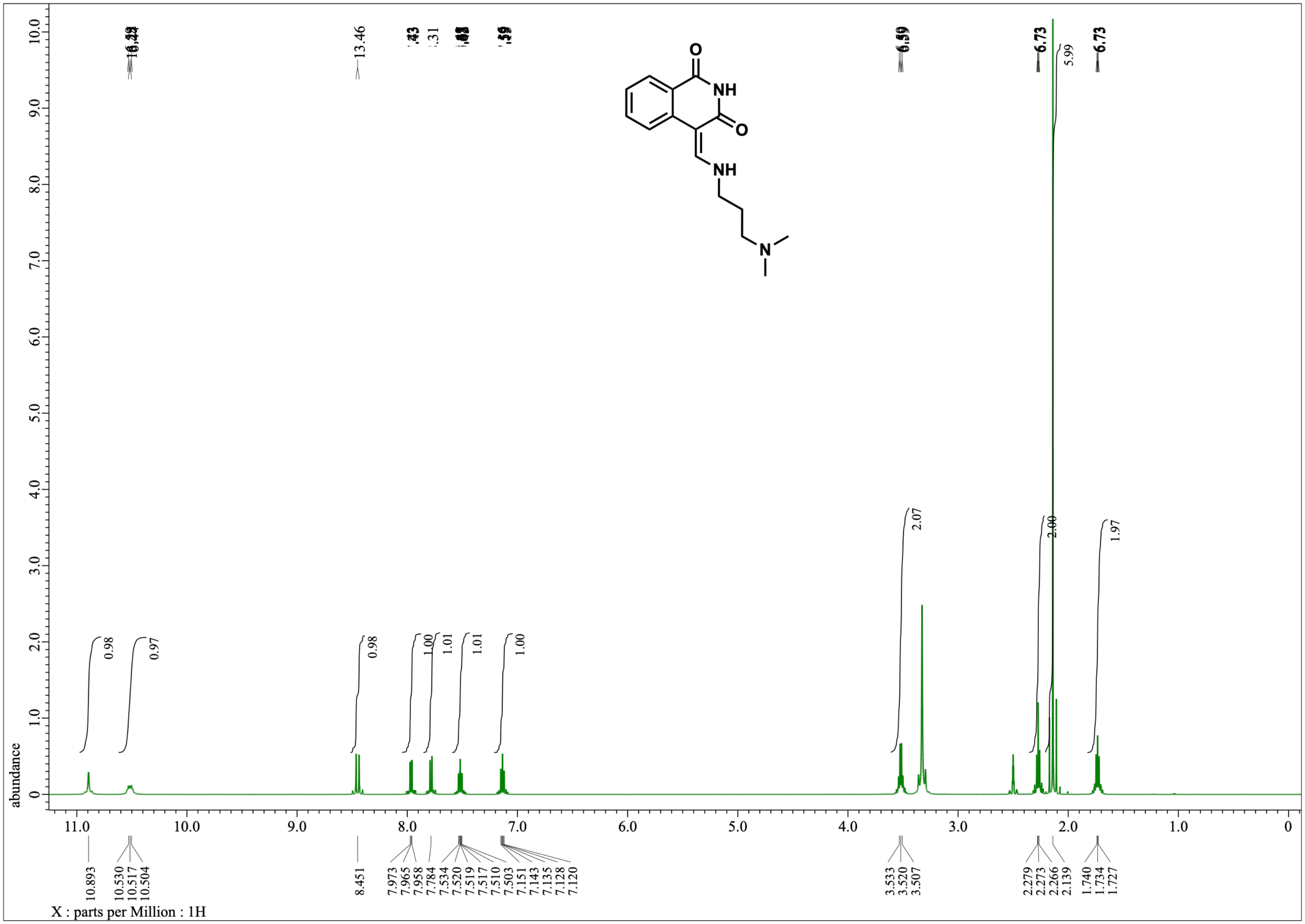

Figure S 33: ^{13}C-NMR (APT) spectra (125 MHz) of TR-018 in DMSO-d_6

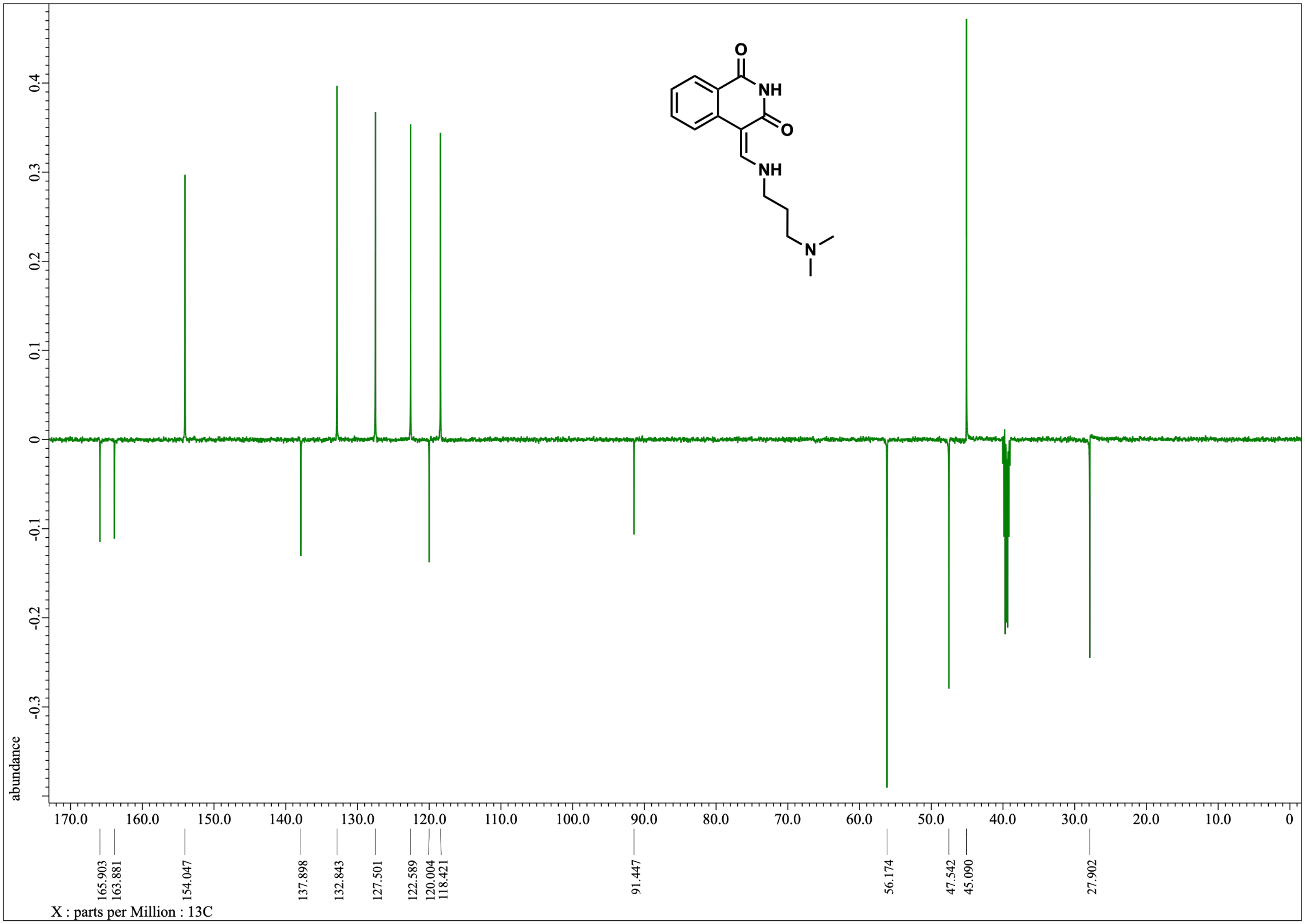

Figure S 34: ^{1}H-NMR spectra (500 MHz) of TR-021 in DMSO-d_6

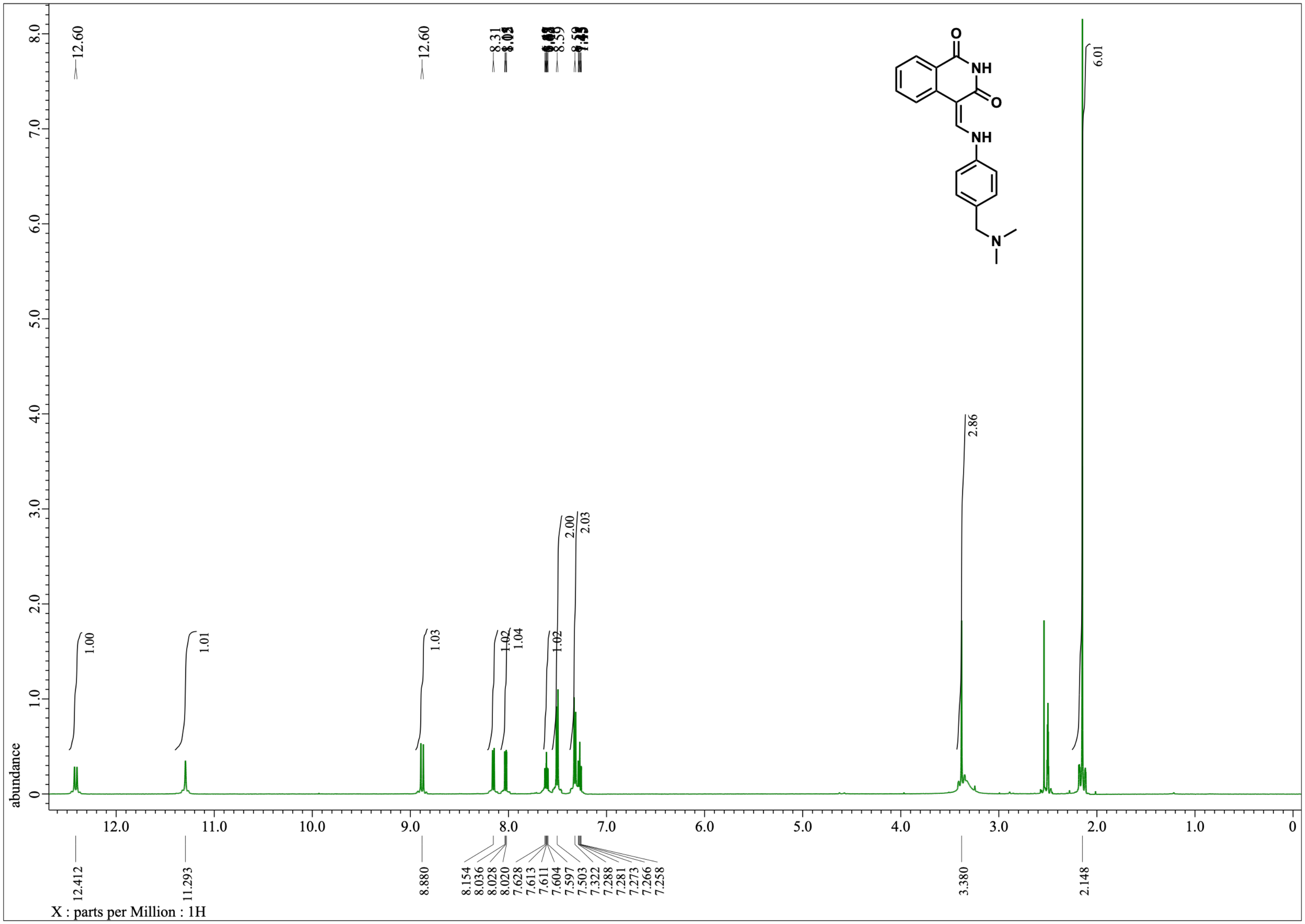

Figure S 35: ^{13}C-NMR (APT) spectra (125 MHz) of TR-021 in DMSO-d_6

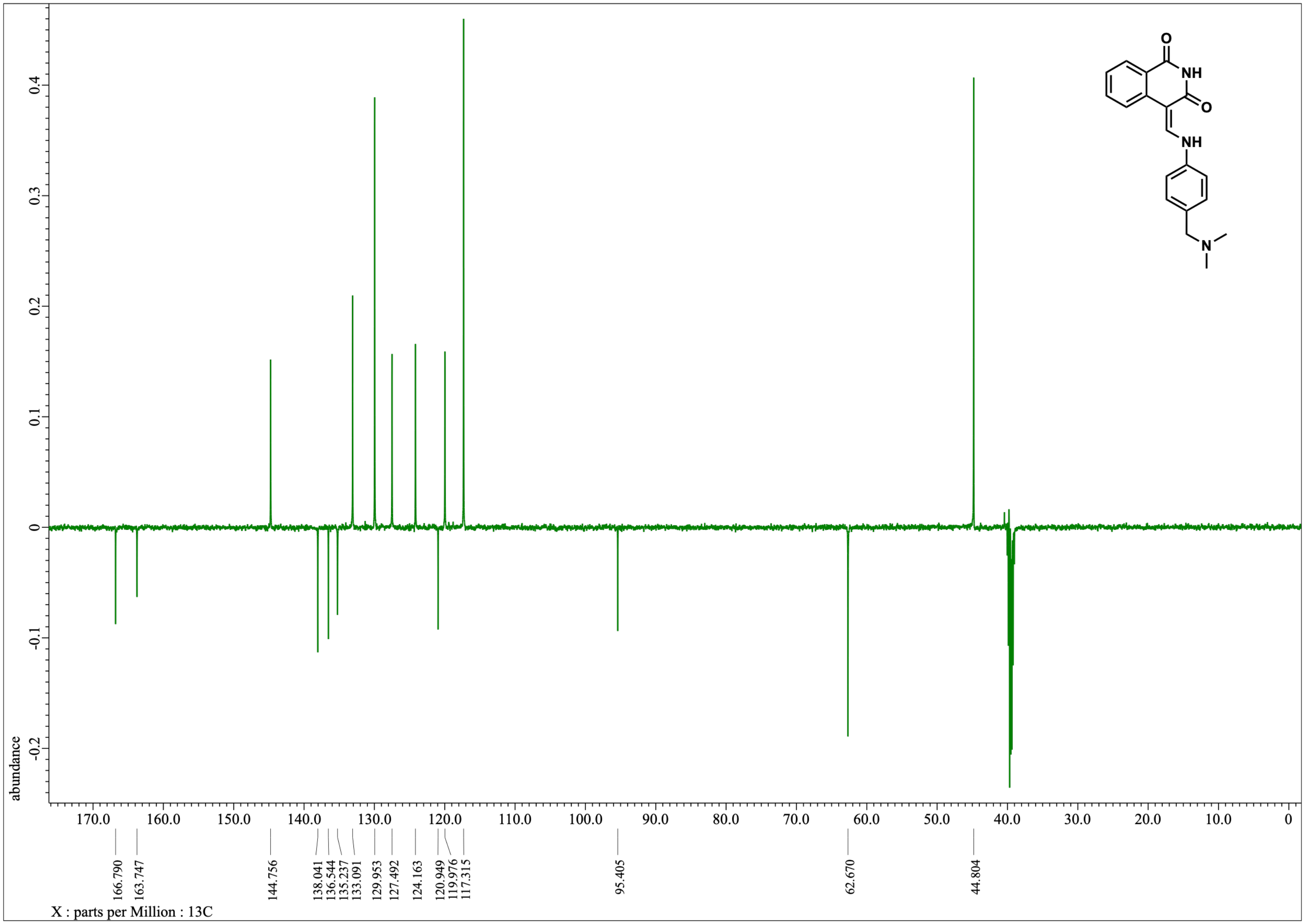

Figure S 36: ^{1}H-NMR spectra (500 MHz) of TR-022 in DMSO-d_6

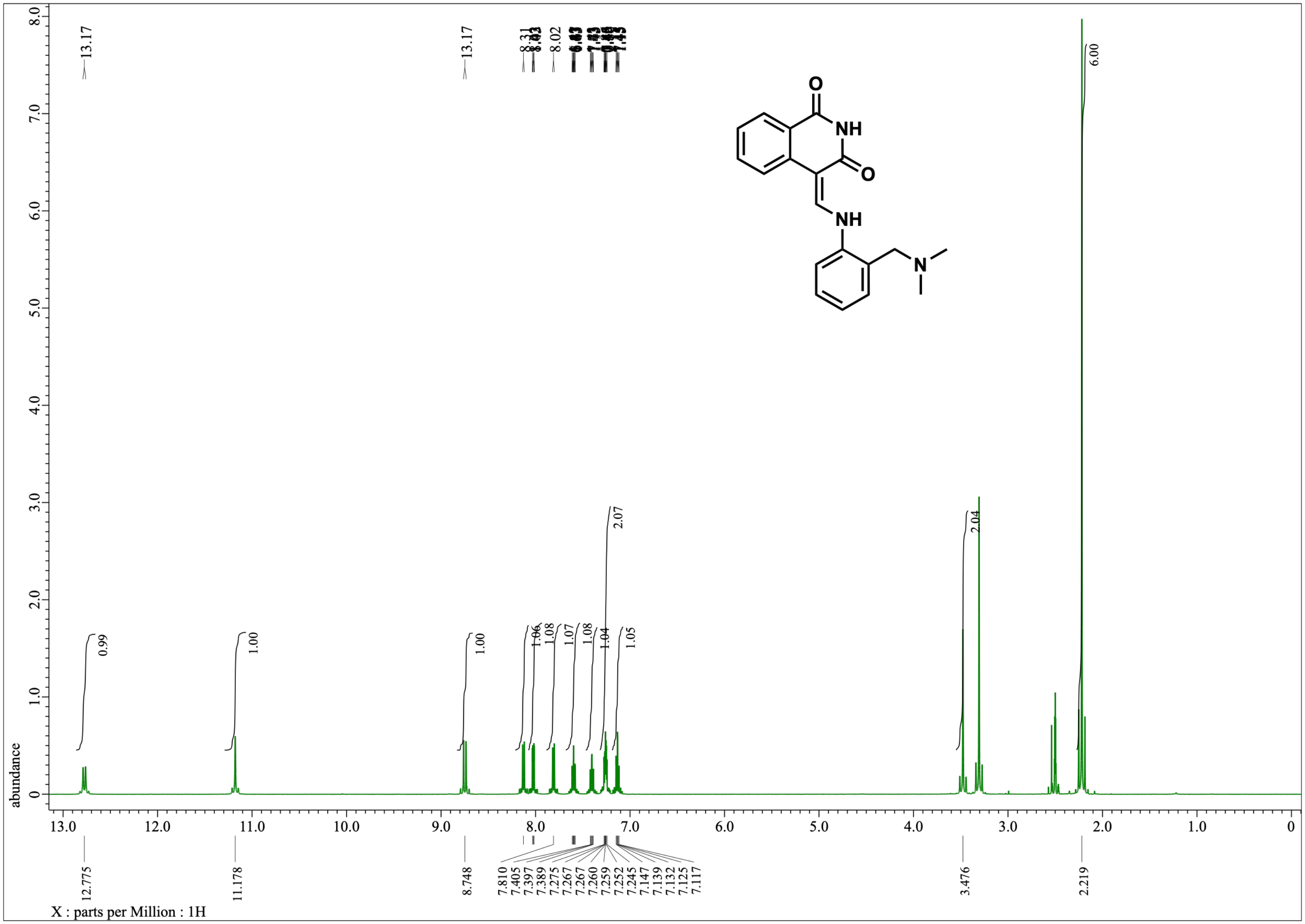

Figure S 37: ^{13}C-NMR (APT) spectra (125 MHz) of TR-022 in DMSO-d_6

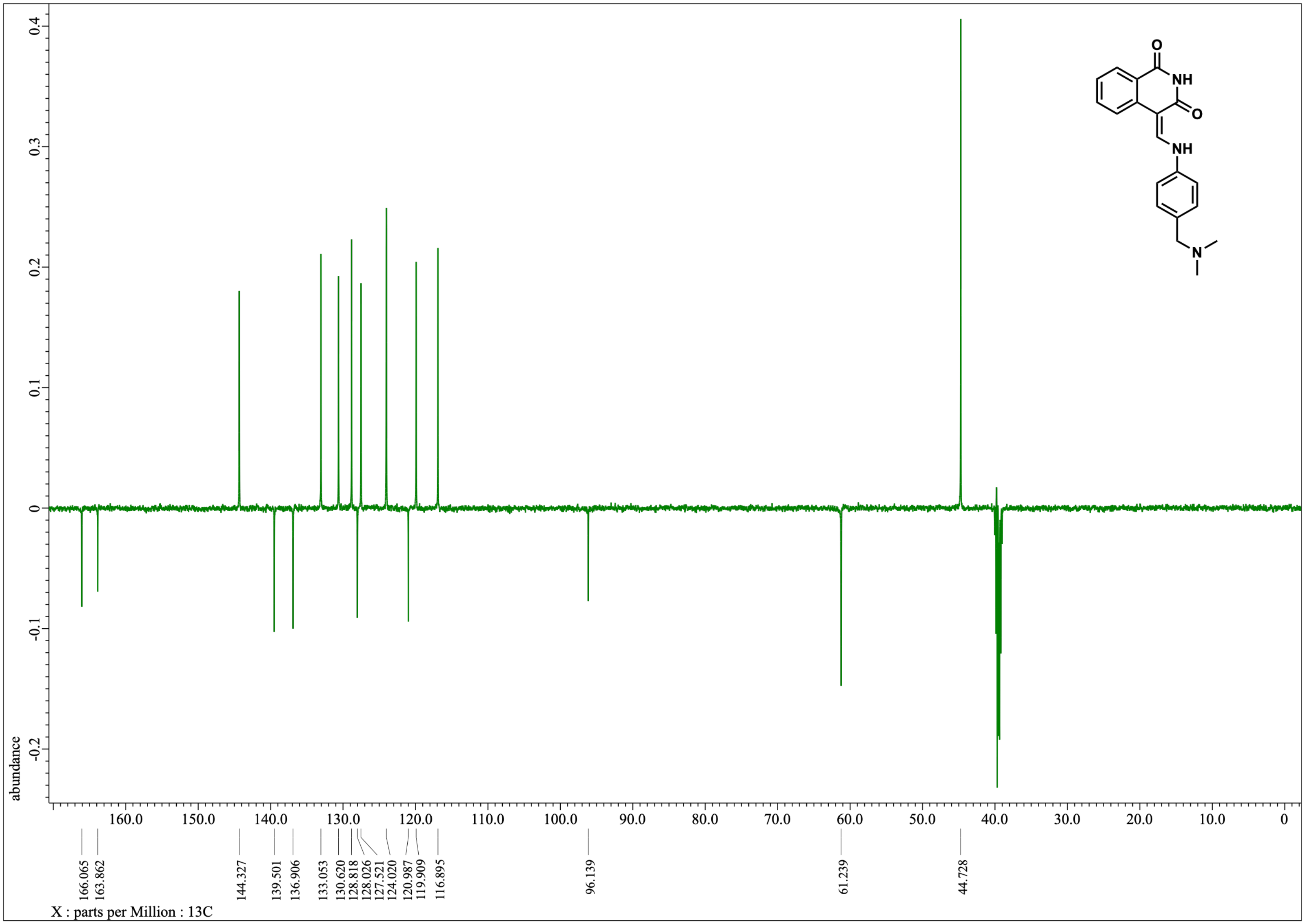

Figure S 38: ^{1}H-NMR spectra (500 MHz) of TR-030 in DMSO-d_6

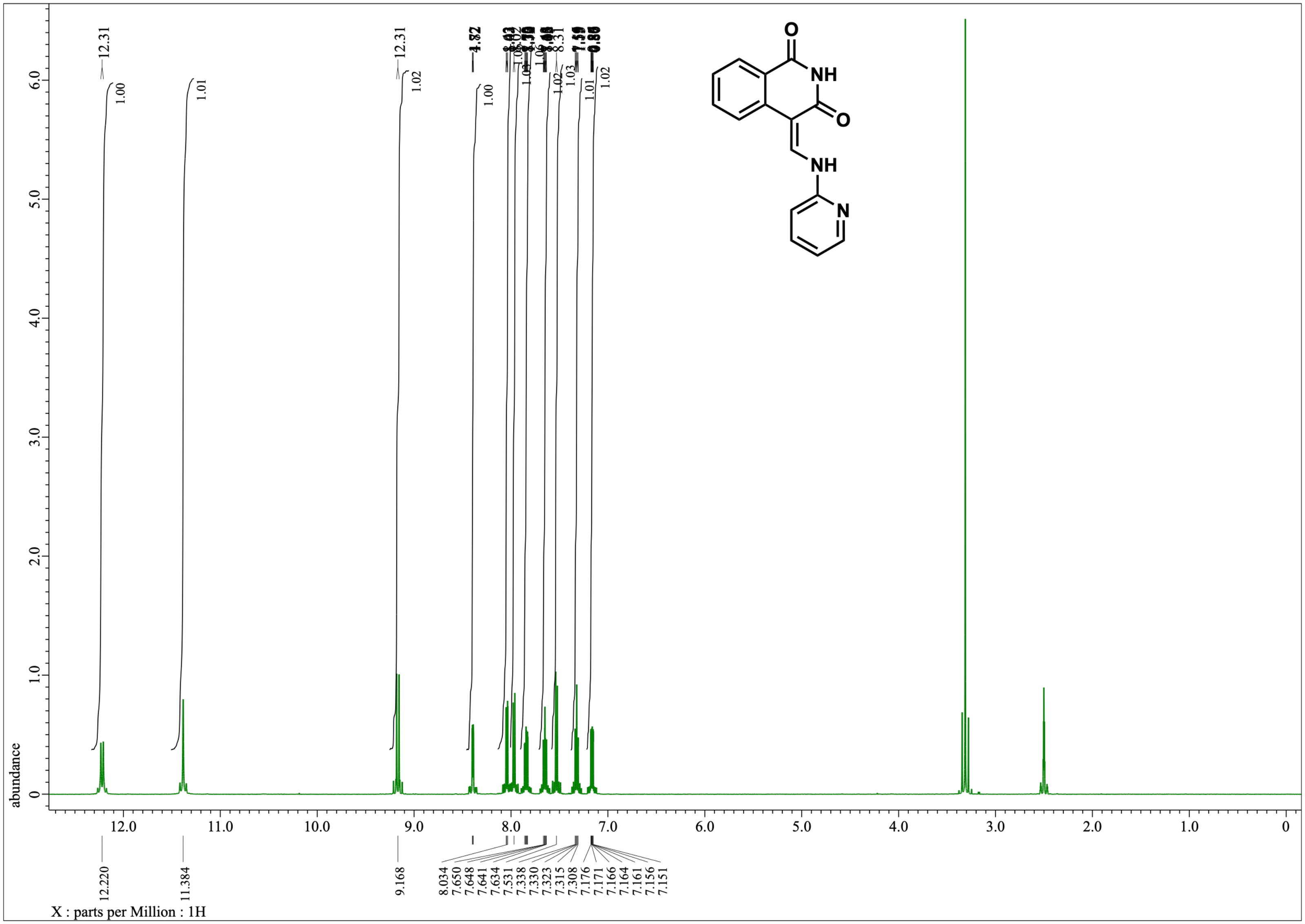

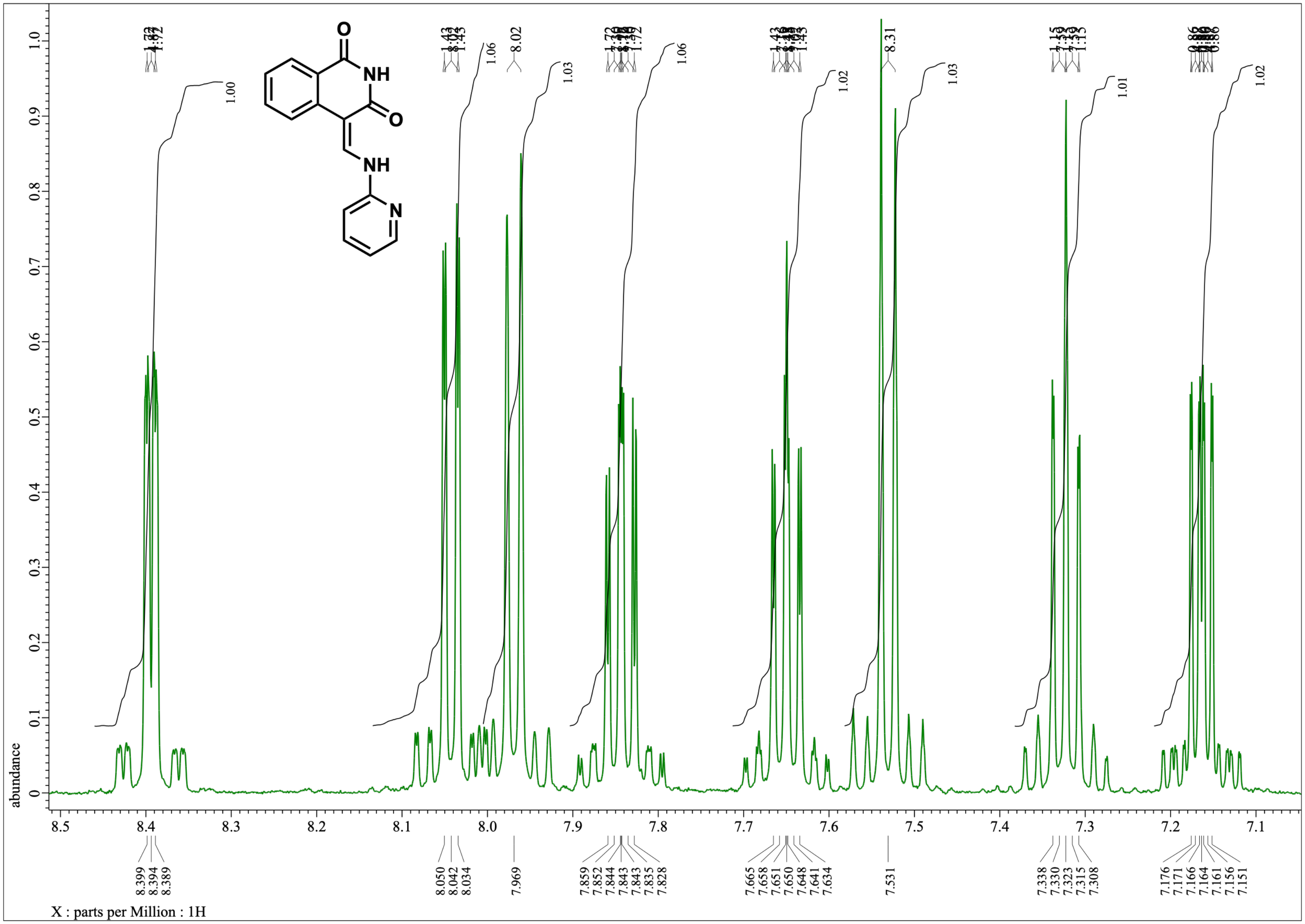

Figure S 39: ^{13}C-NMR (APT) spectra (125 MHz) of TR-030 in DMSO-d_6

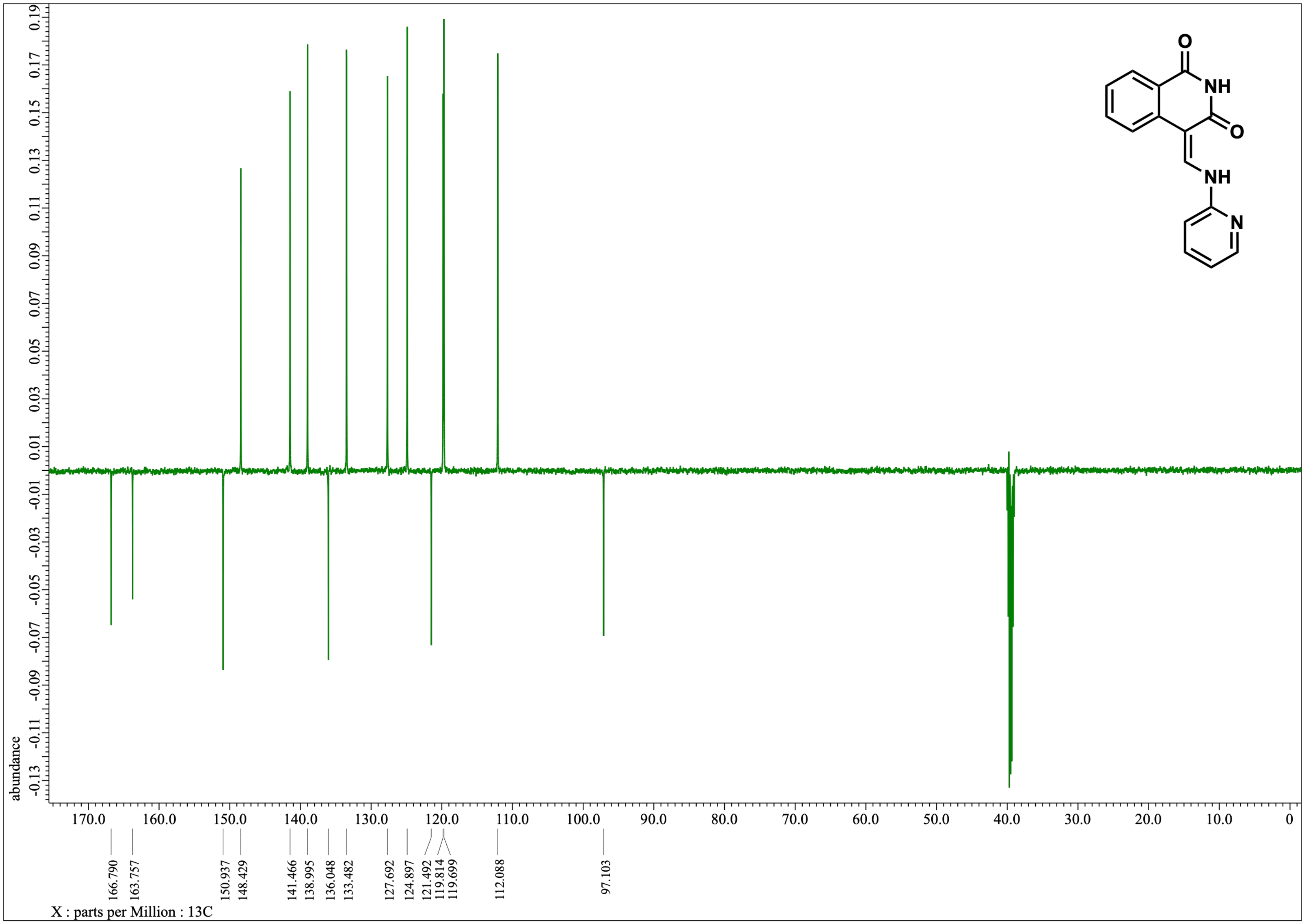

Figure S 40: ^{1}H-NMR spectra (500 MHz) of TR-031 in DMSO-d_6

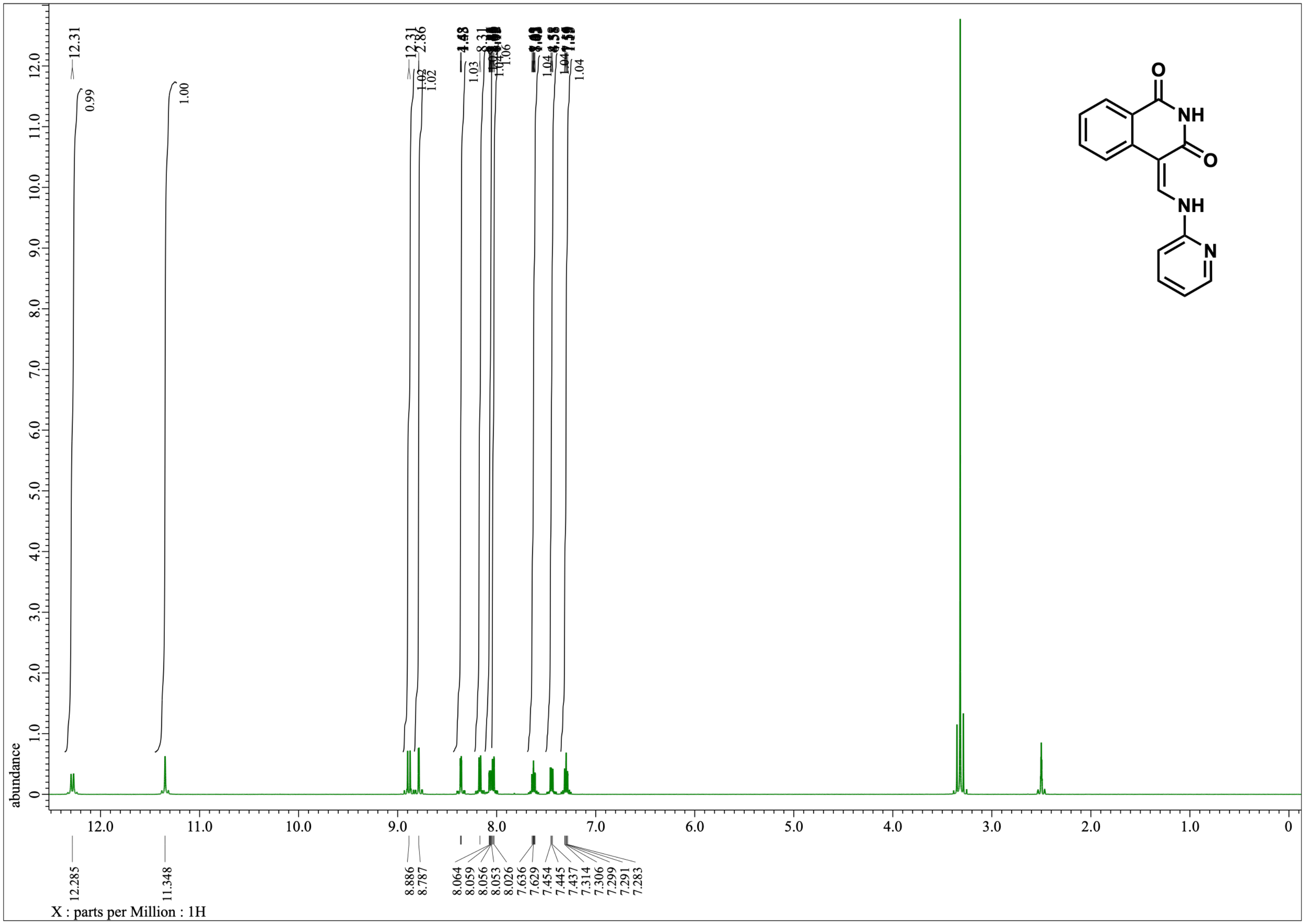

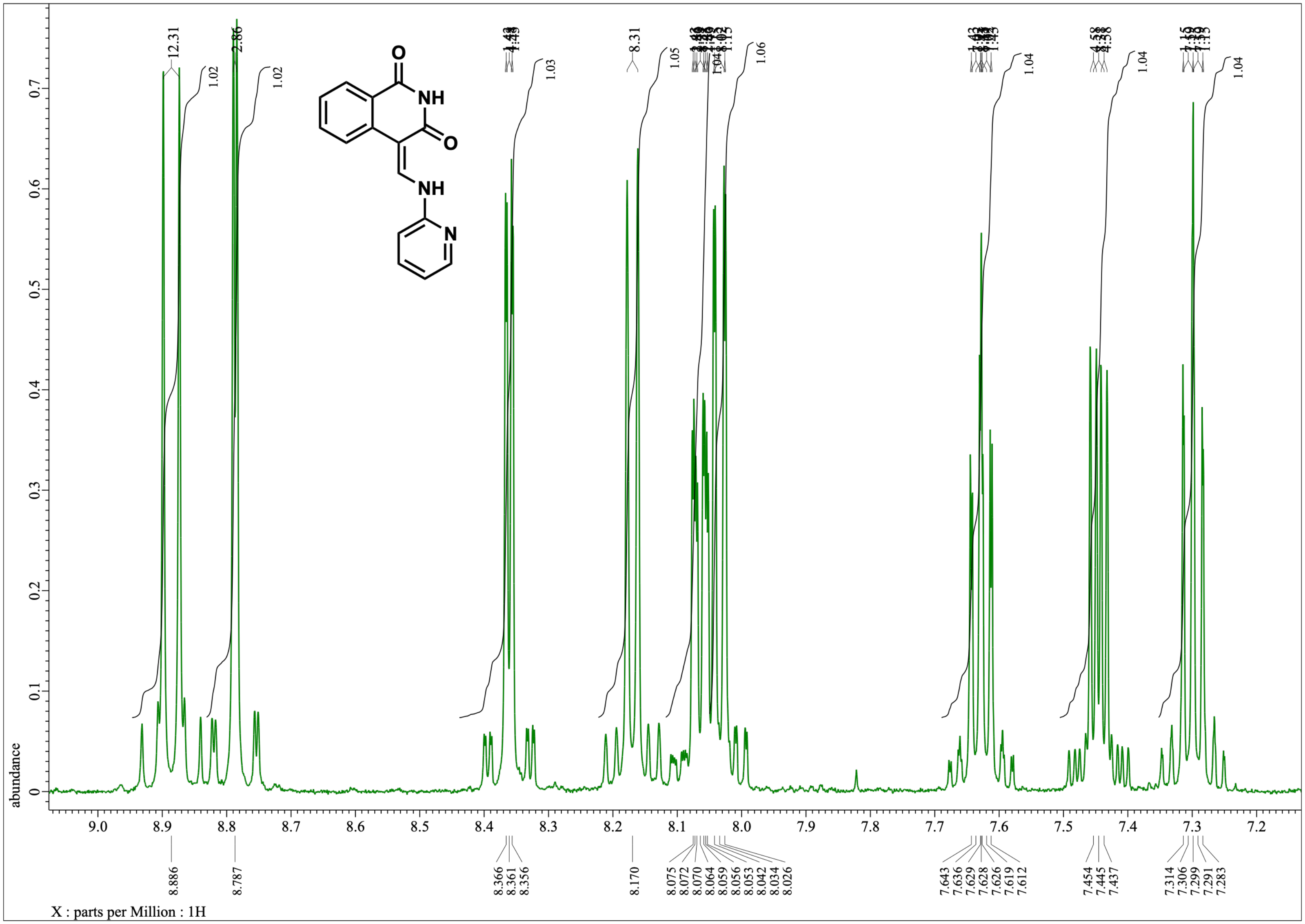

O
NH
O
NH
N
abundance
X : parts per Million : 1H
9.0
8.9
8.8
8.7
8.6
8.5
8.4
8.3
8.2
8.1
8.0
7.9
7.8
7.7
7.6
7.5
7.4
7.3
7.2
0
0.1
0.2
0.3
0.4
0.5
0.6
0.7
8.886
8.787
8.366
8.361
8.356
8.170
8.075
8.072
8.070
8.064
8.059
8.056
8.053
8.042
8.034
8.026
7.643
7.636
7.629
7.628
7.626
7.619
7.612
7.454
7.445
7.437
7.314
7.306
7.299
7.291
7.283
12.31
2.86
8.31
1.02
1.02
1.03
1.05
1.06
1.04
1.04
1.04

Figure S 41: ^{13}C-NMR (APT) spectra (100 MHz) of TR-031 in DMSO-d_6

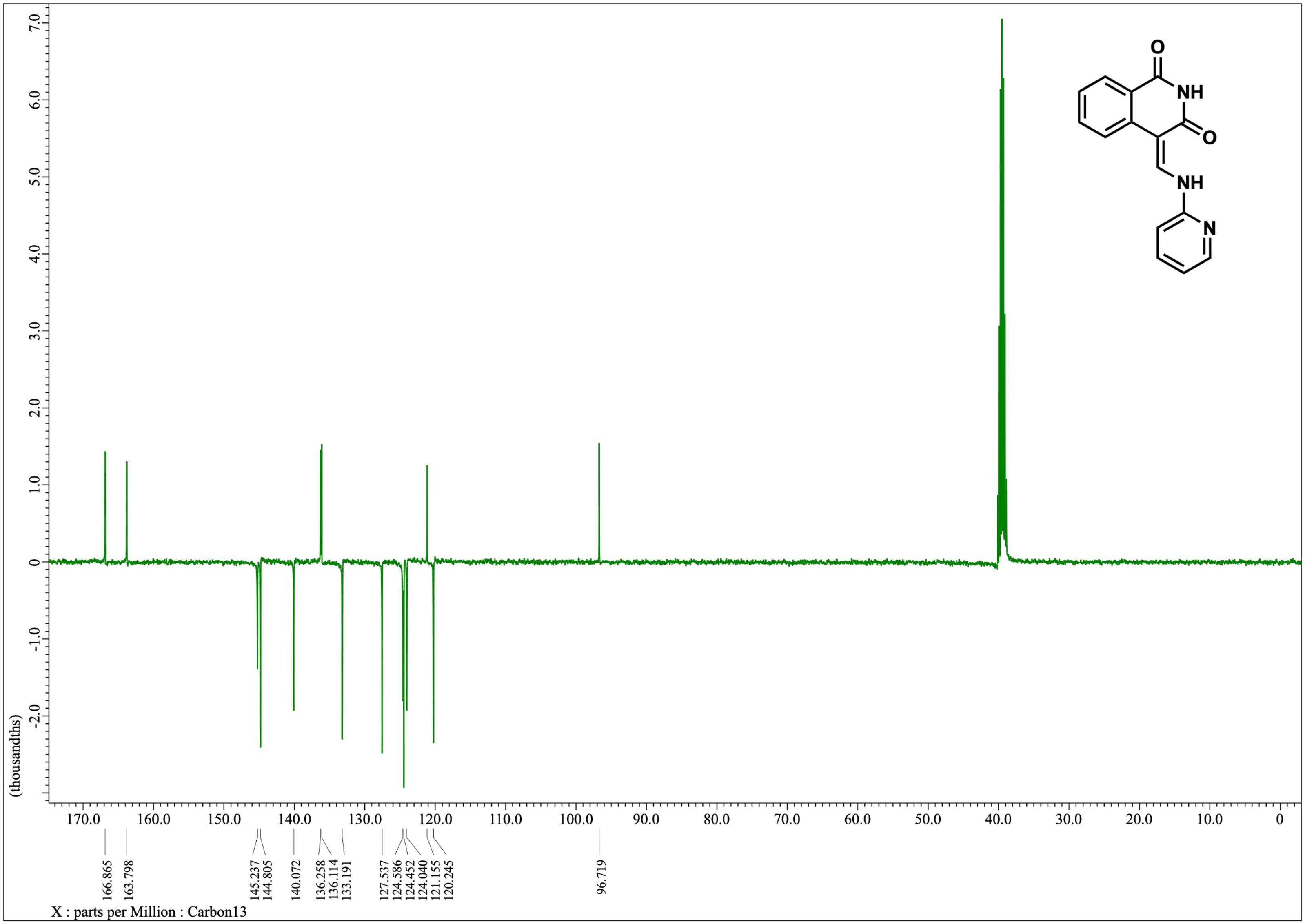

Figure S 42: ^{1}H-NMR spectra (500 MHz) of TR-032 in DMSO-*d₆*

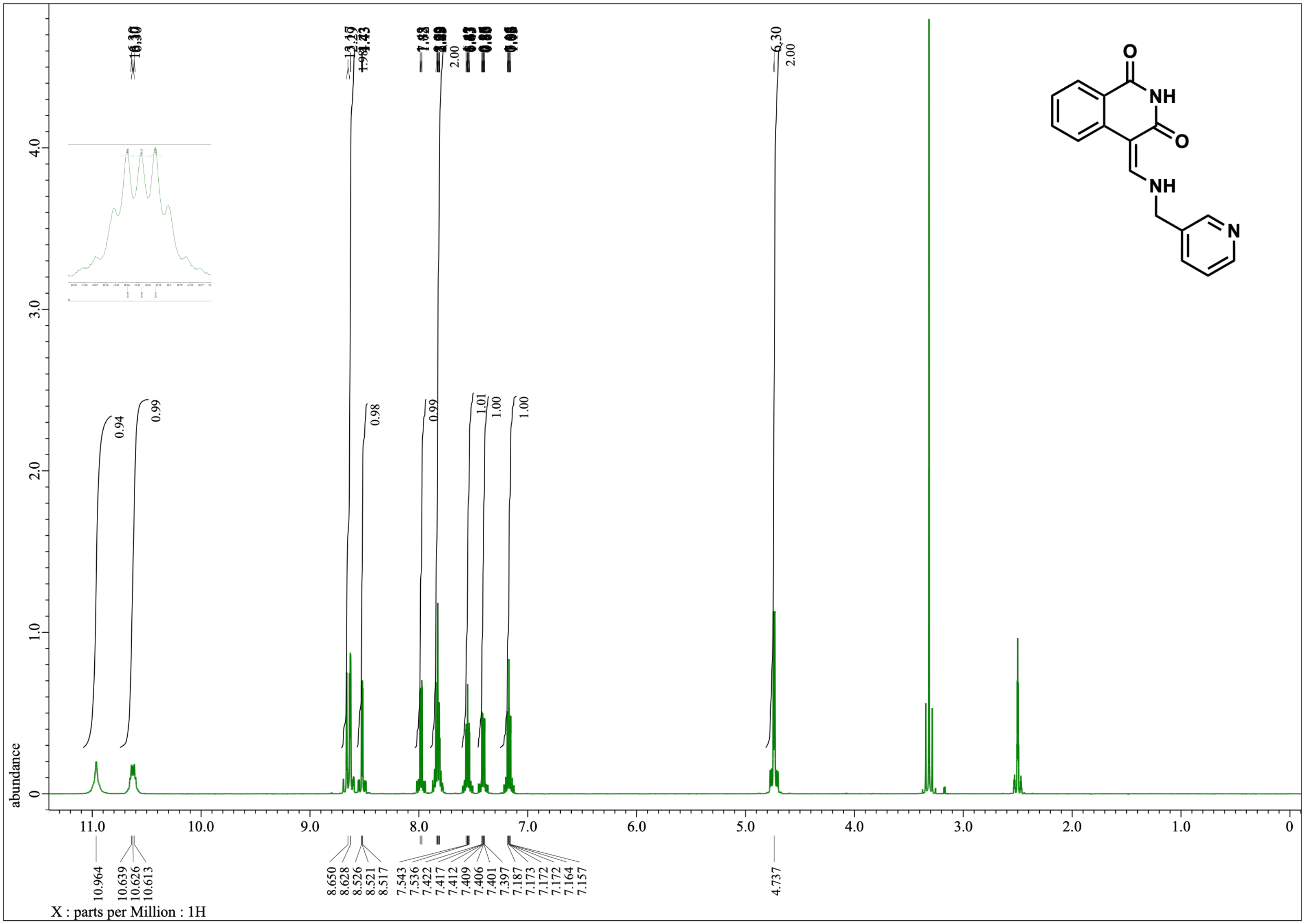

Figure S 43: ^{13}C-NMR (APT) spectra (125 MHz) of TR-032 in DMSO-d_6

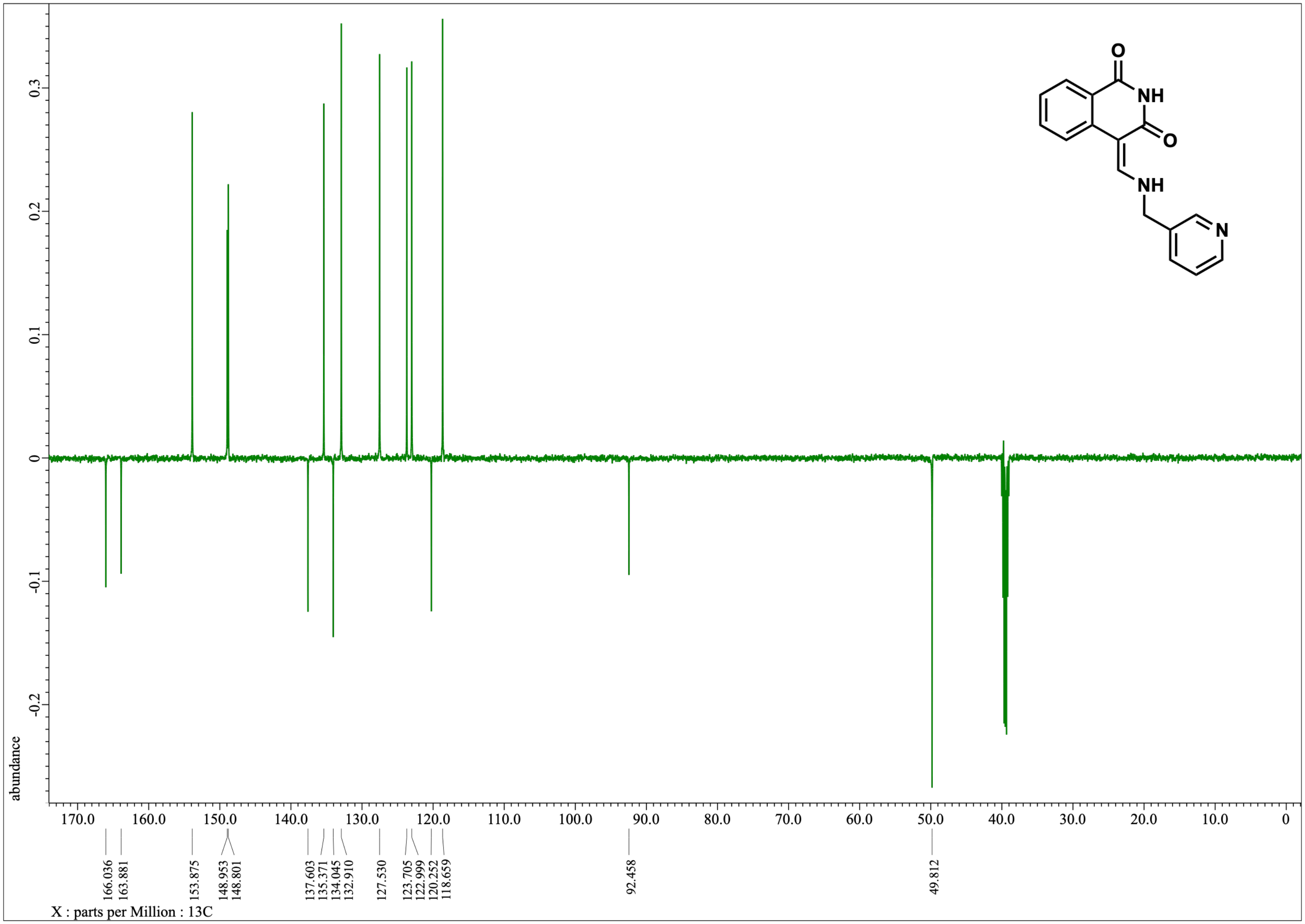

Figure S 44: ^{1}H-NMR spectra (400 MHz) of TR-033 in DMSO-d_6

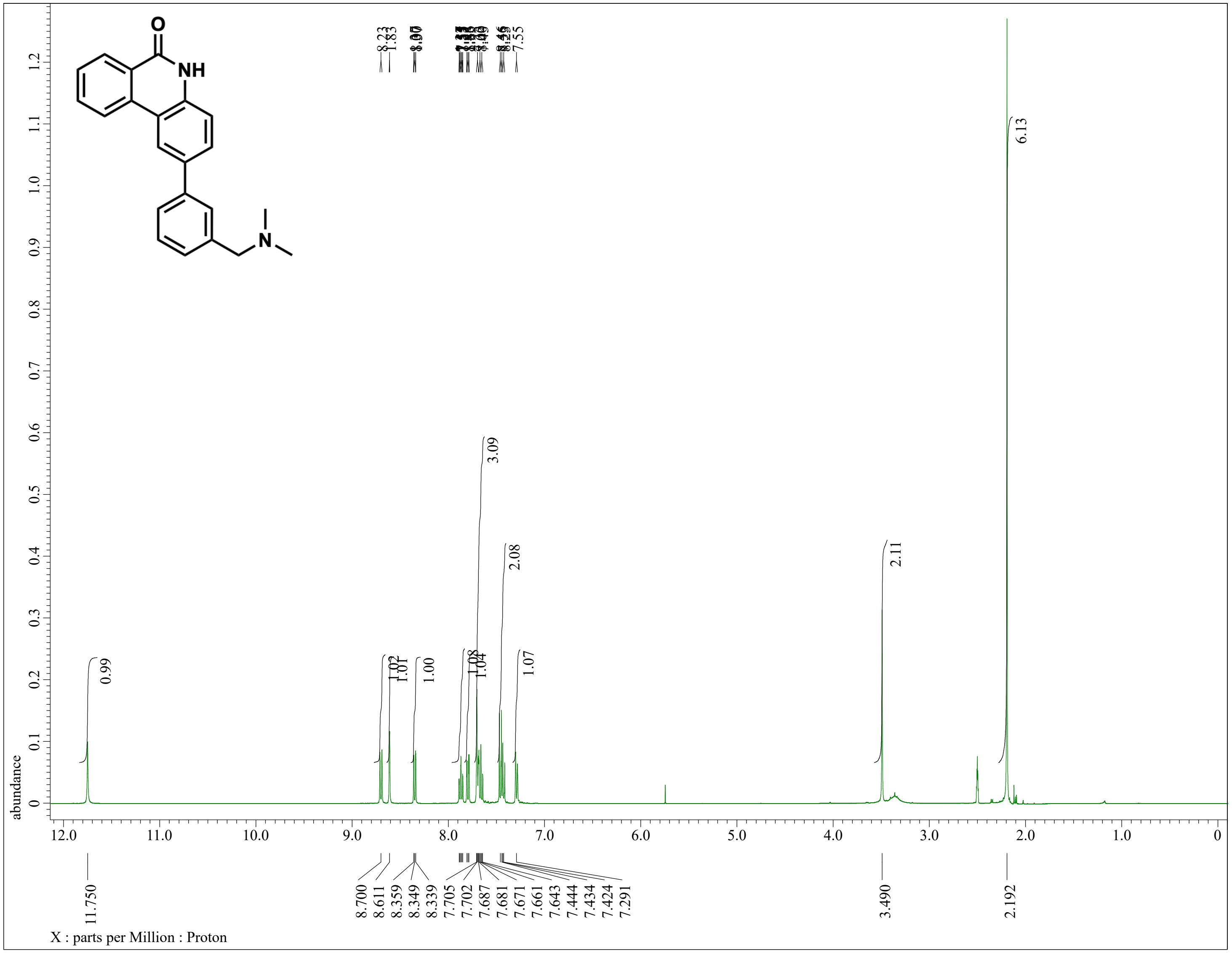

Figure S 45: ^{13}C-NMR (APT) spectra (100 MHz) of TR-033 in DMSO-d_6

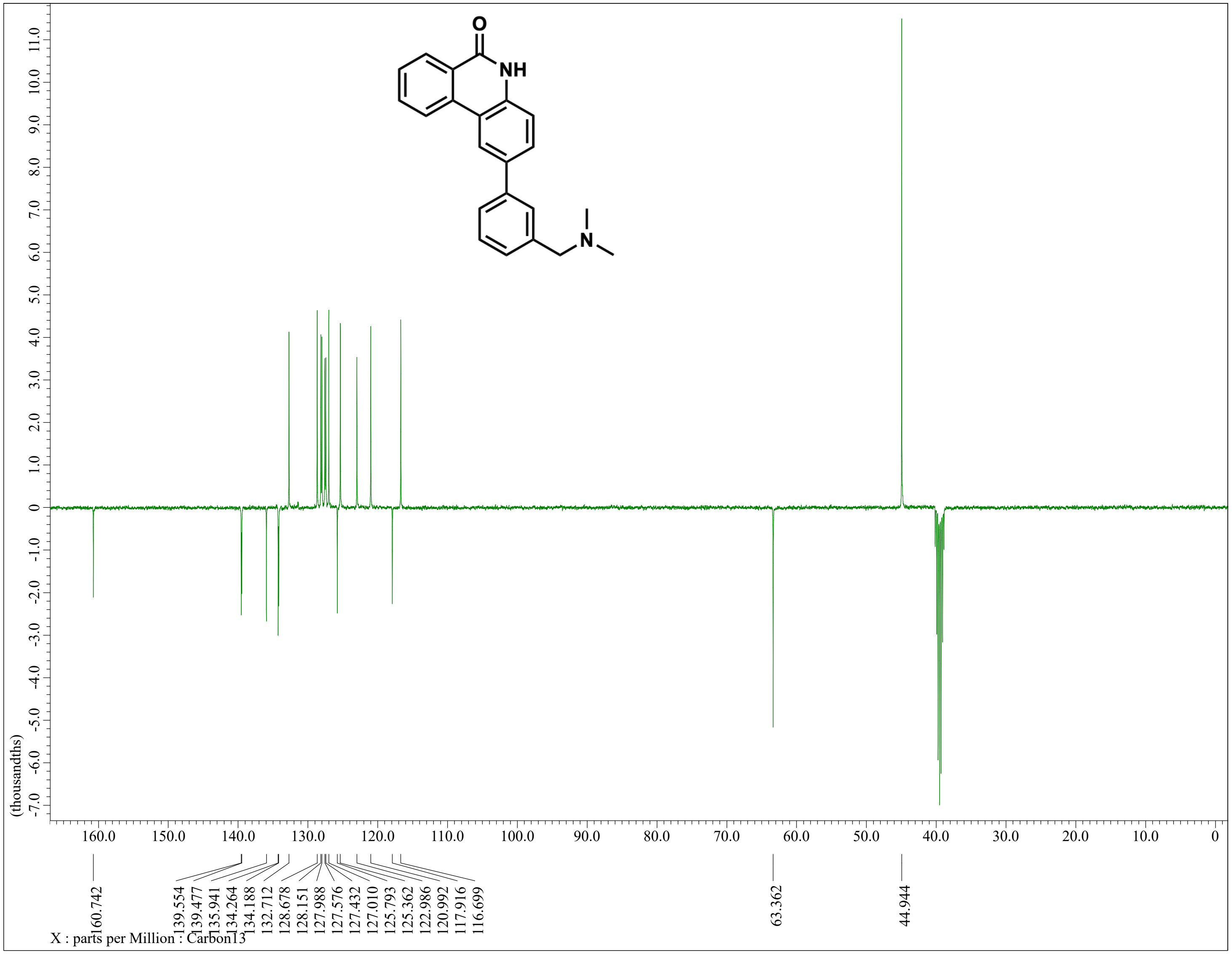

Figure S 46: ^{1}H-NMR spectra (500 MHz) of TR-036*HCl in DMSO-*d₆*

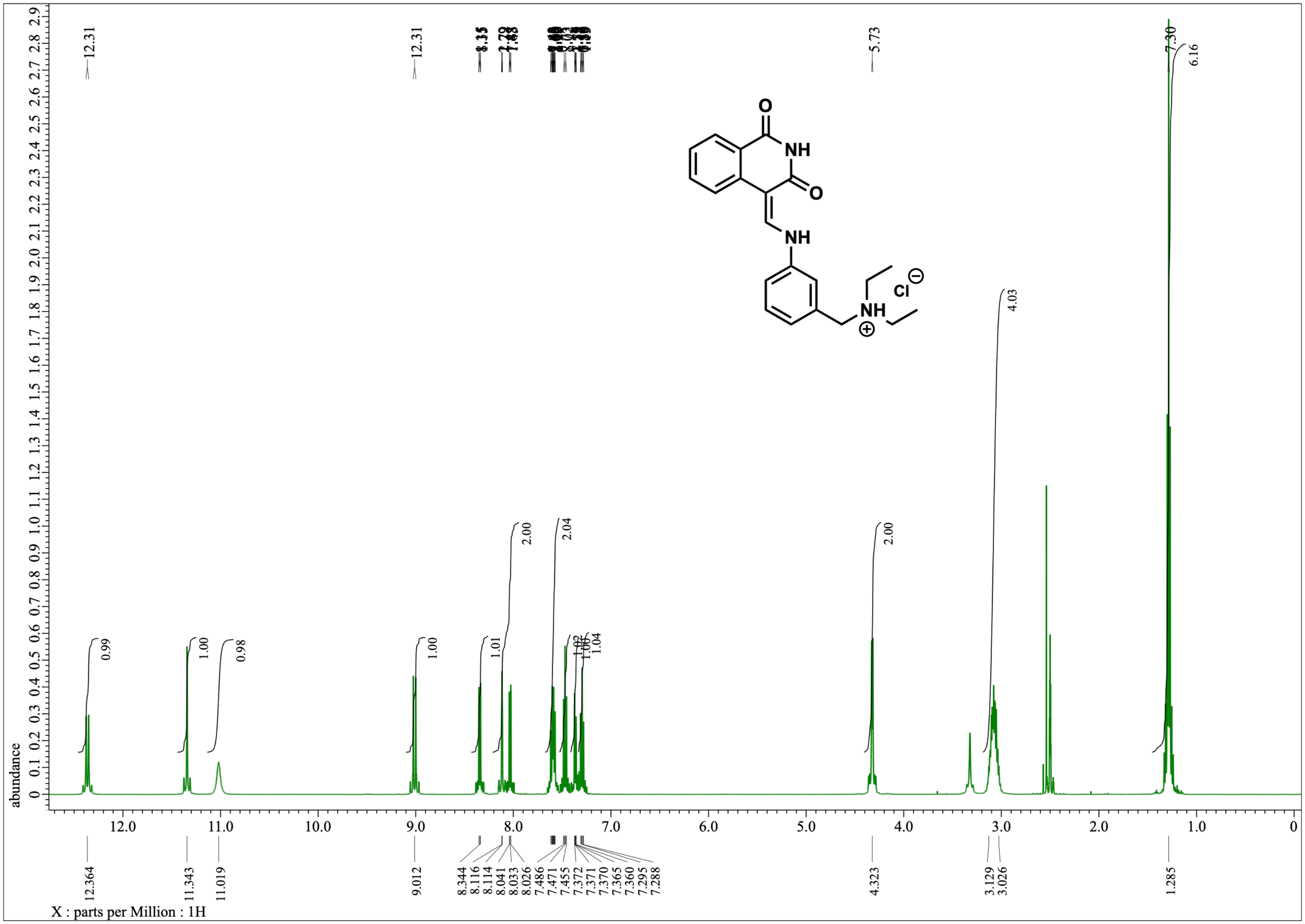

Figure S 47: ^{13}C-NMR (APT) spectra (125 MHz) of TR-036*HCl in DMSO-d_6

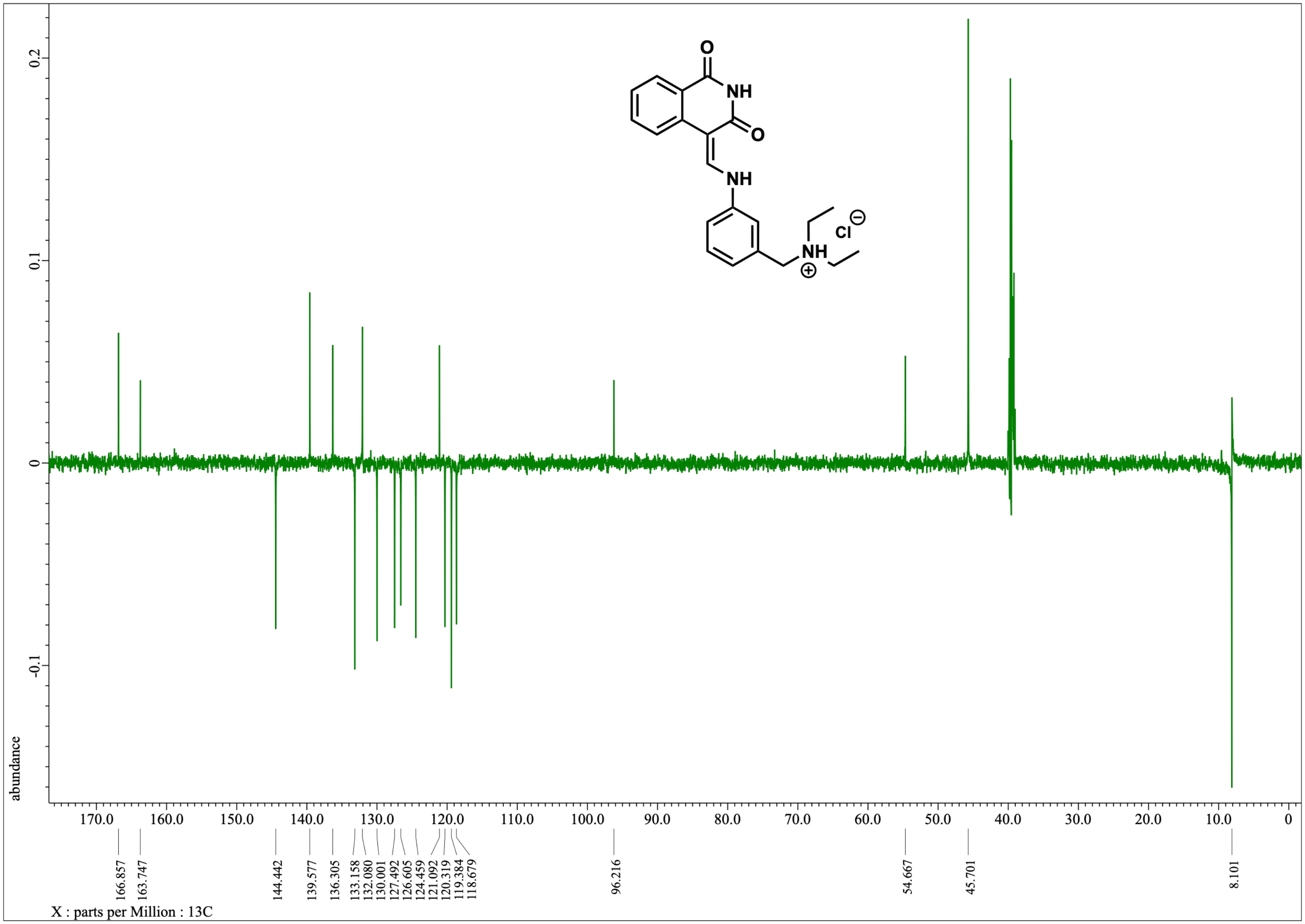

Figure S 48: ^{1}H-NMR spectra (500 MHz) of TR-038 in DMSO-d_6

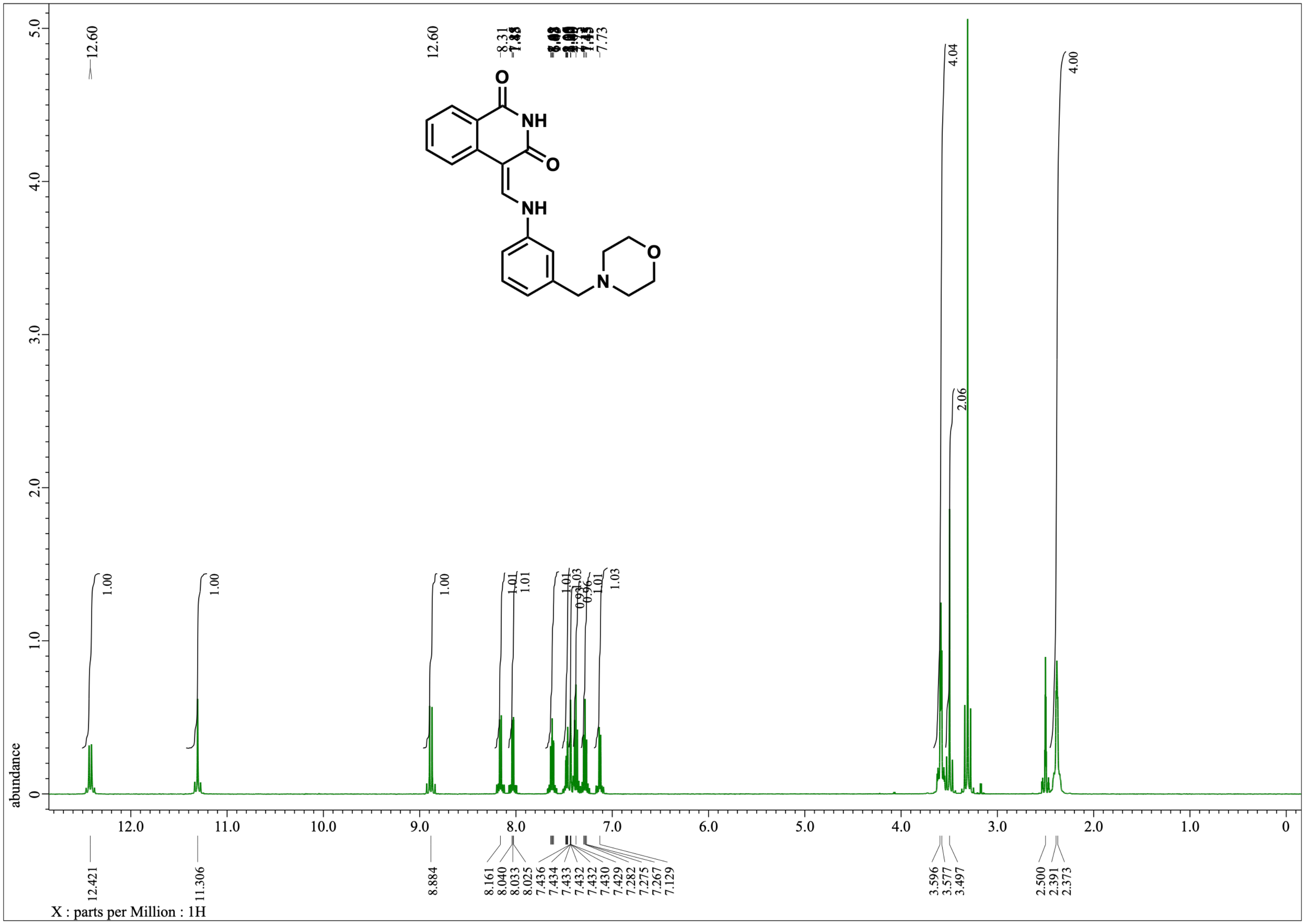

Figure S 49: ^{13}C-NMR (APT) spectra (125 MHz) of TR-038 in DMSO-d_6

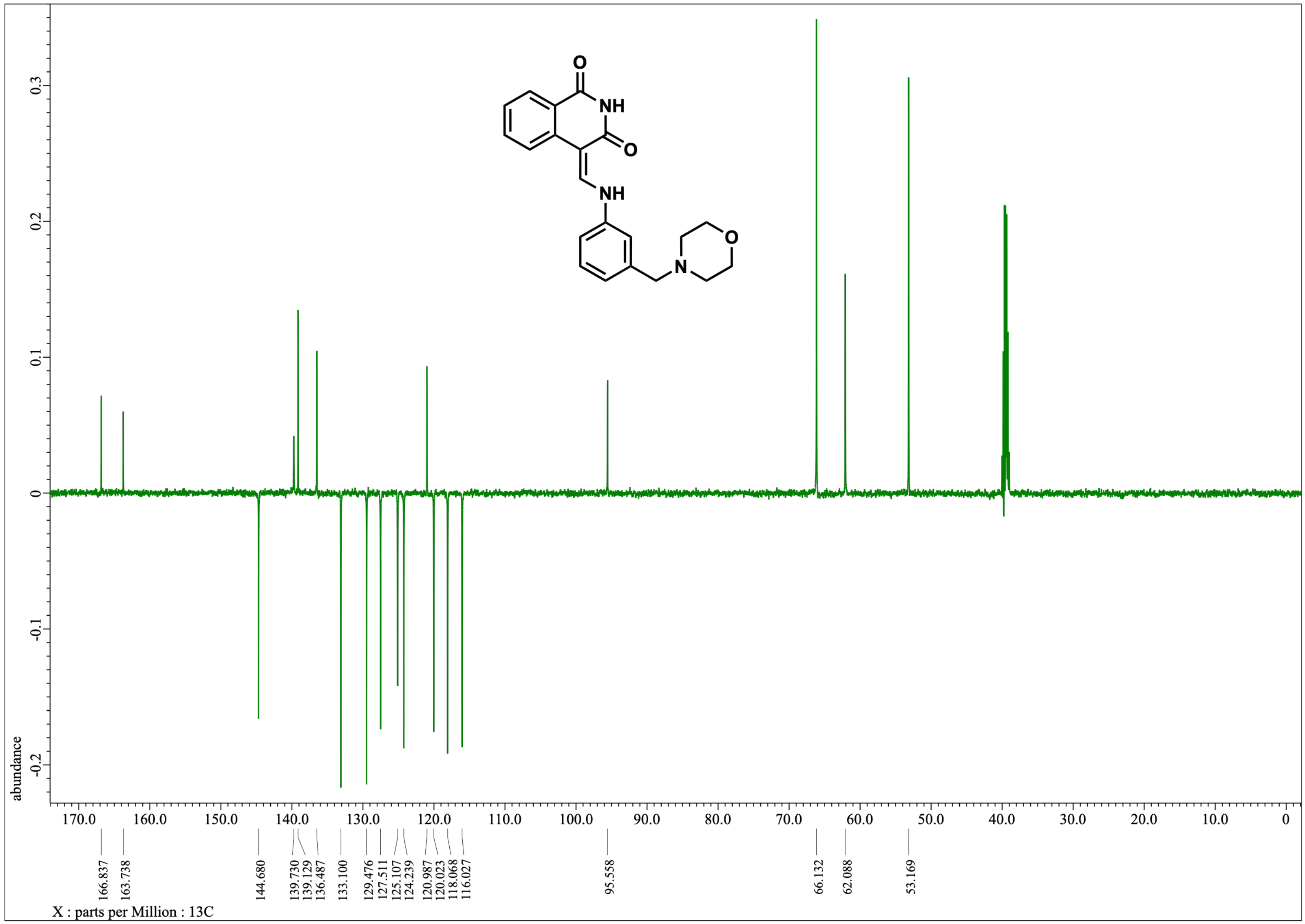

Figure S 50: ^{1}H-NMR spectra (500 MHz) of ICTR-015 in DMSO-d_6

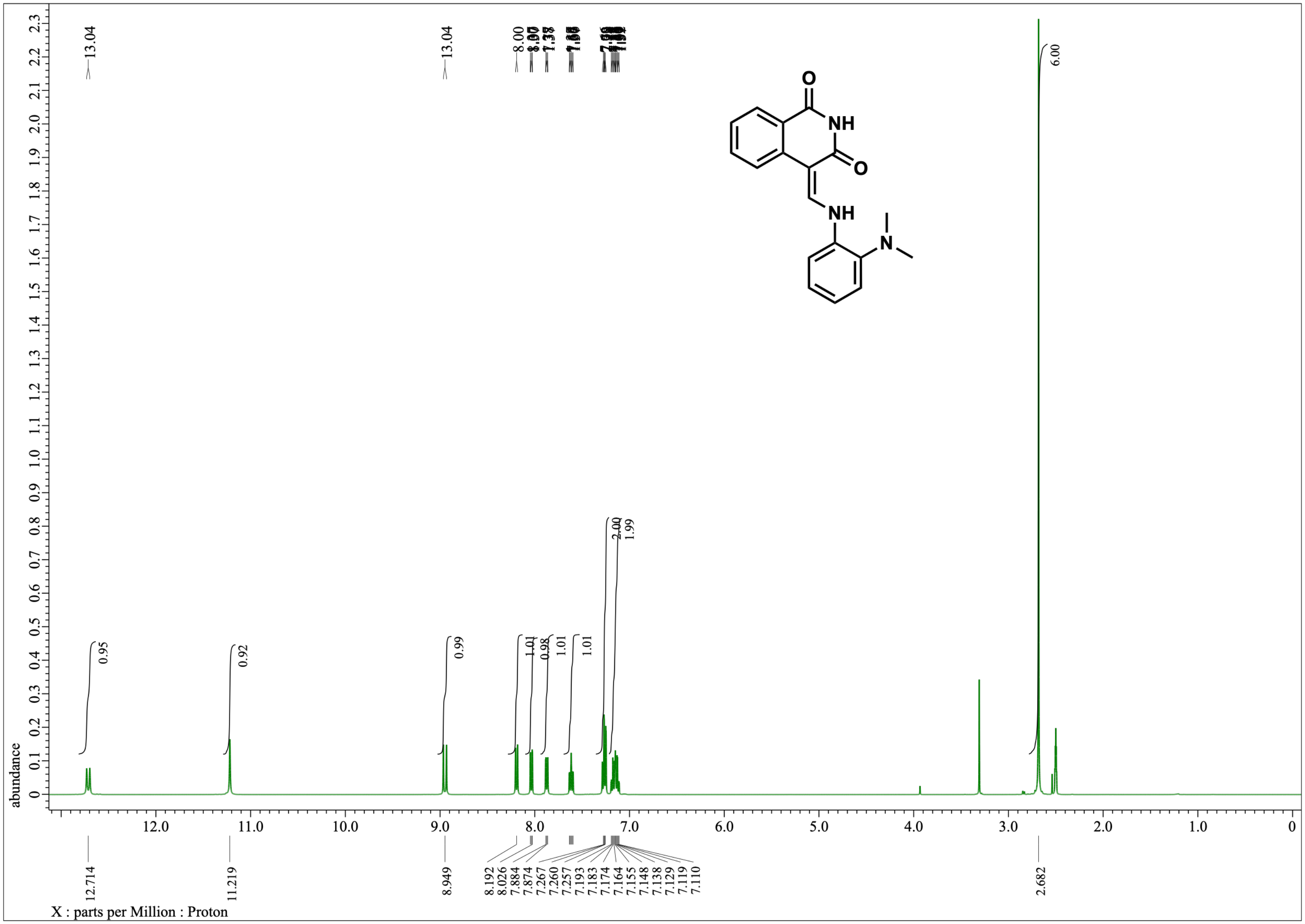

Figure S 51: ^{13}C-NMR (APT) spectra (100 MHz) of ICTR-015 in DMSO-d_6

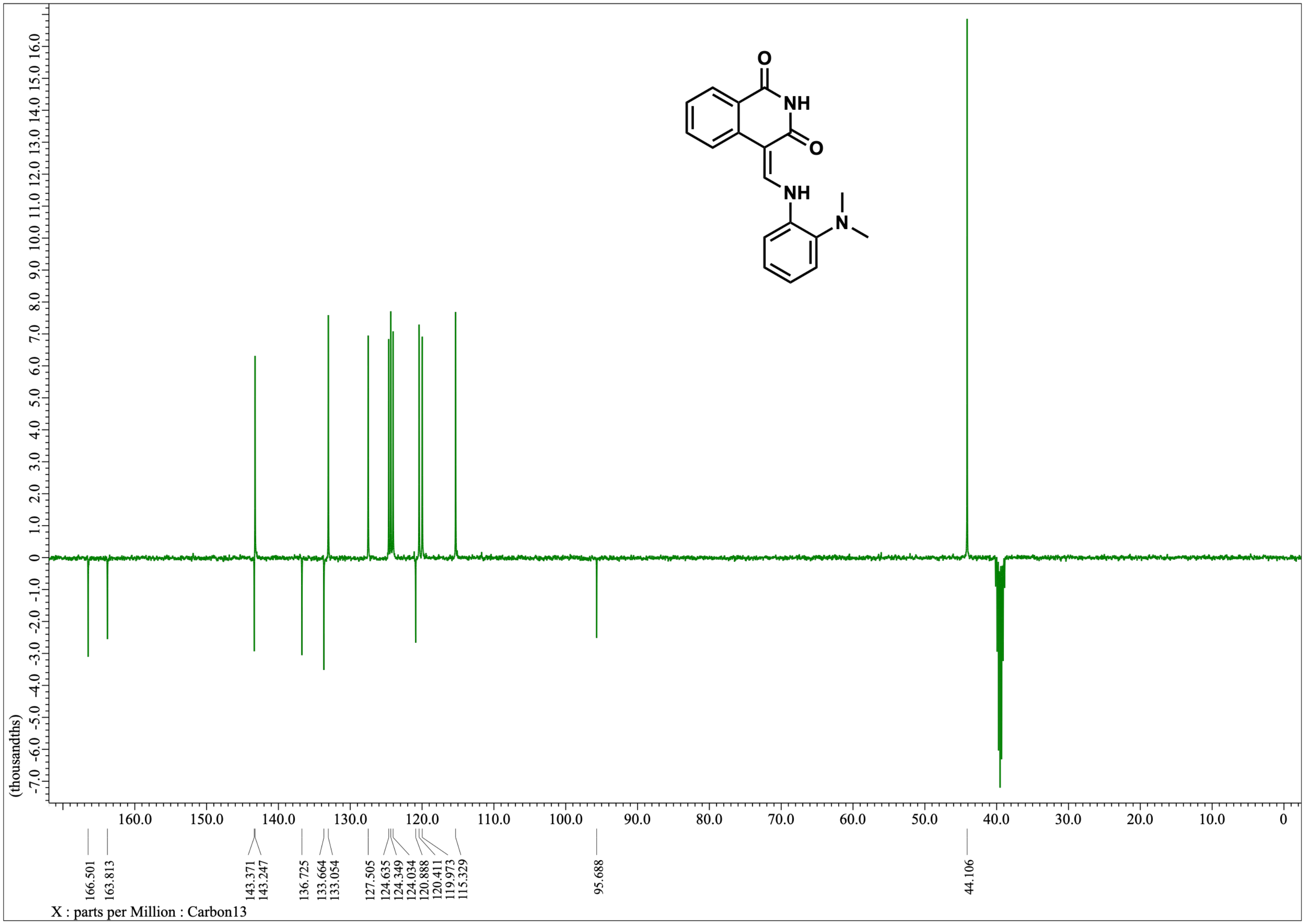

Figure S 52: ^{1}H-NMR spectra (500 MHz) of ICTR-019 in DMSO-d_6

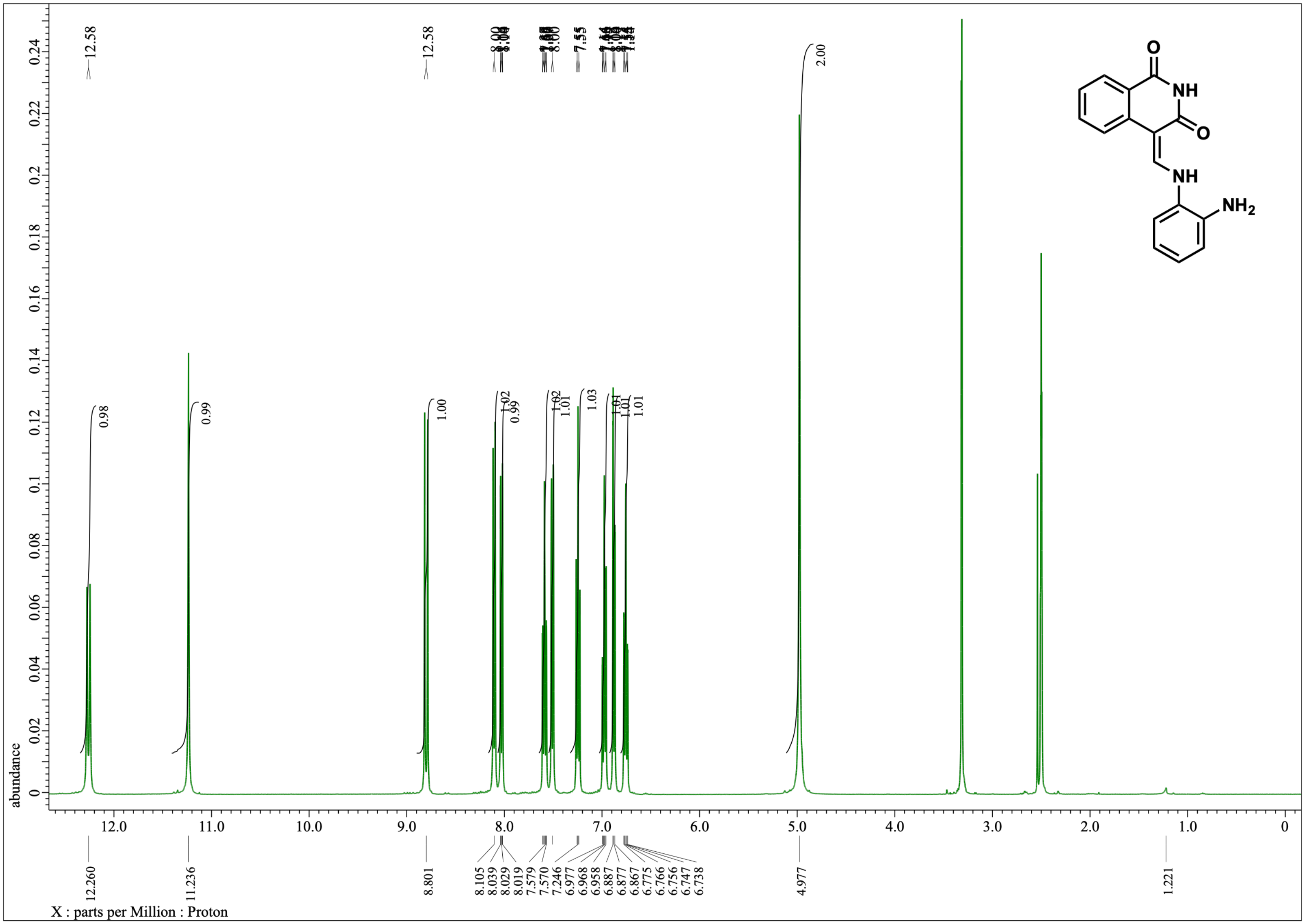

Figure S 53: ^{13}C-NMR (APT) spectra (100 MHz) of ICTR-019 in DMSO-d_6

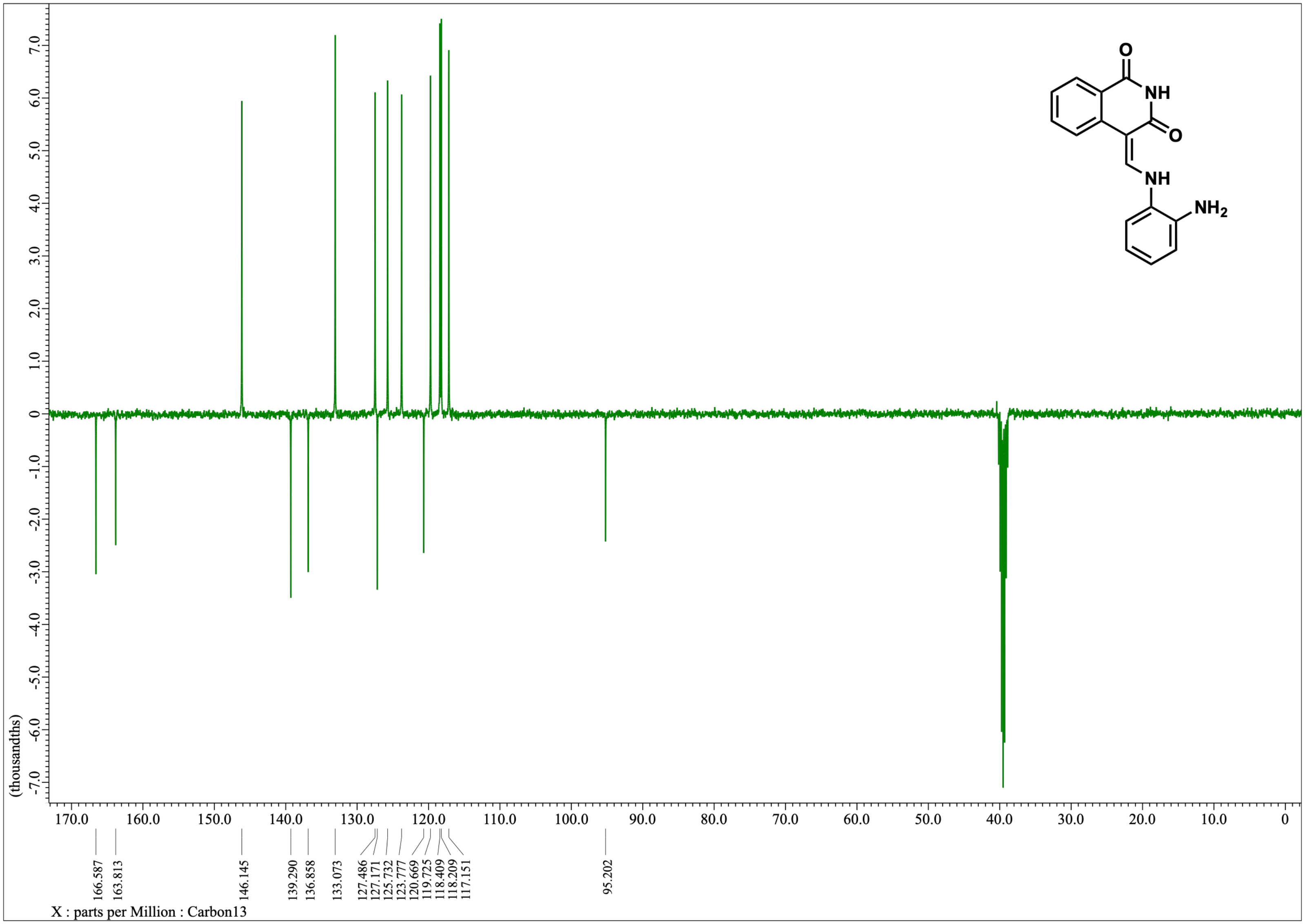

Figure S 54: ^{1}H-NMR spectra (500 MHz) of ICTR-022 in DMSO-d_6

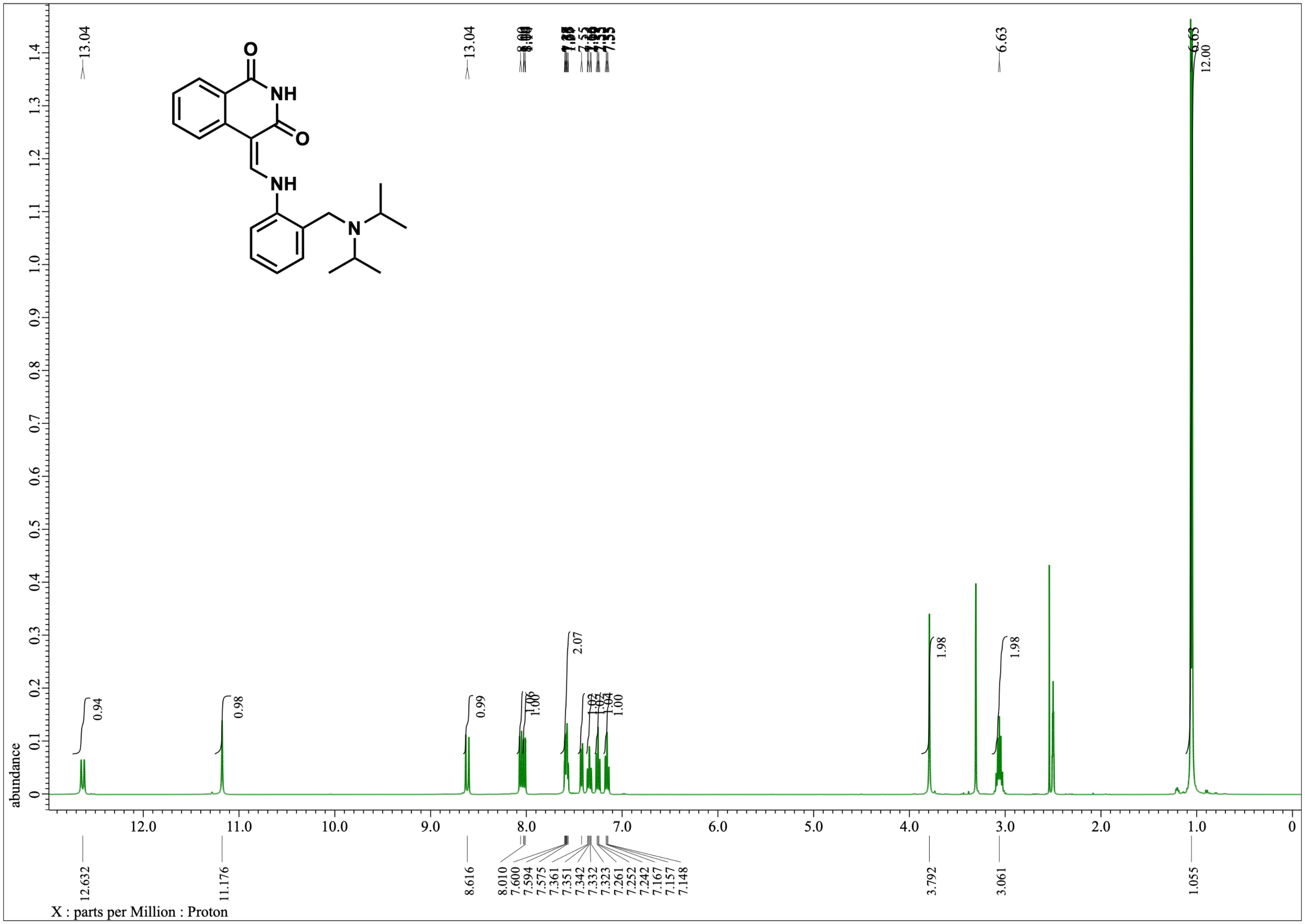

Figure S 55: ^{13}C-NMR (APT) spectra (100 MHz) of ICTR-022 in DMSO-d_6

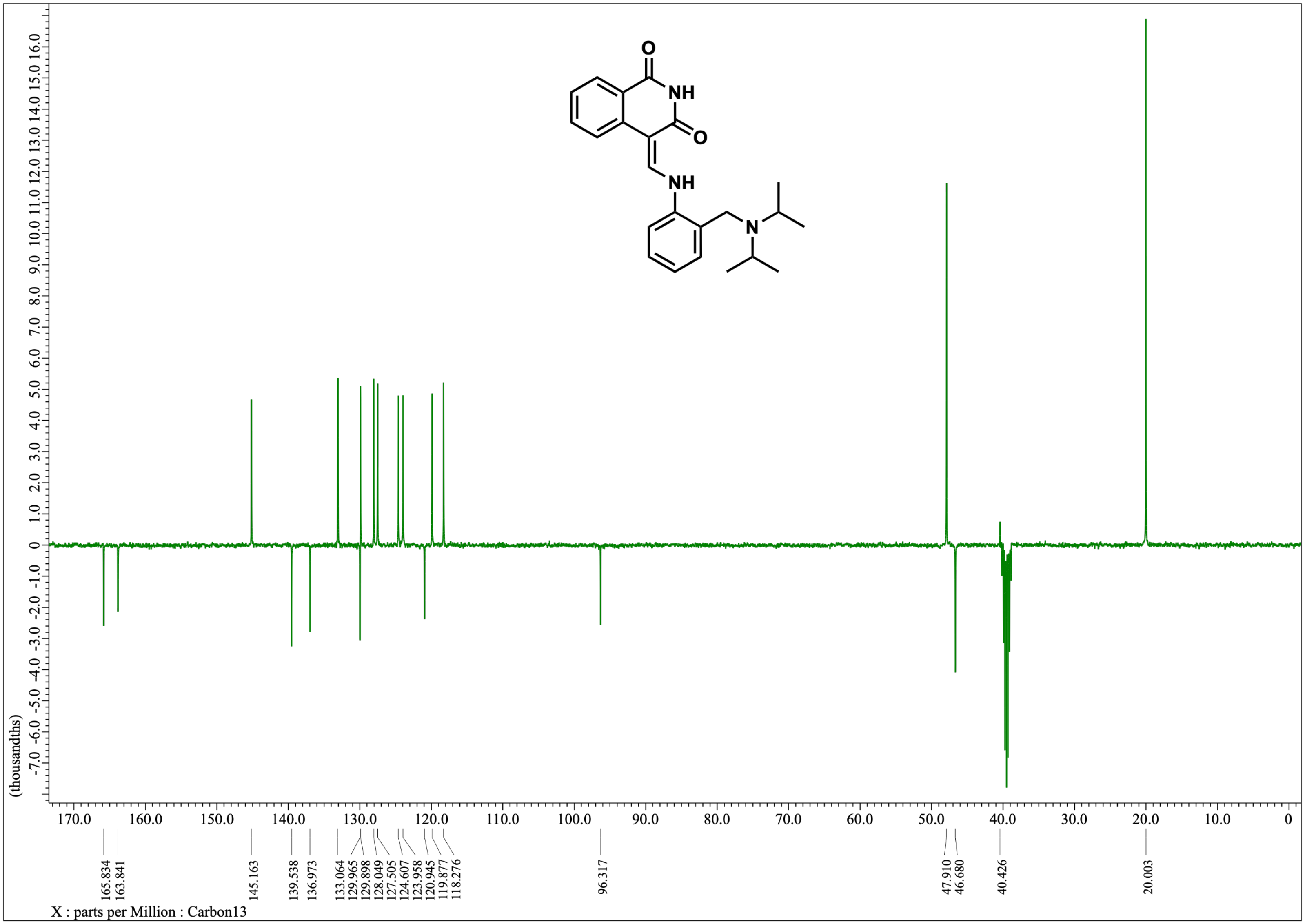

Figure S 56: ^{1}H-NMR spectra (500 MHz) of ICTR-023 in DMSO-d_6

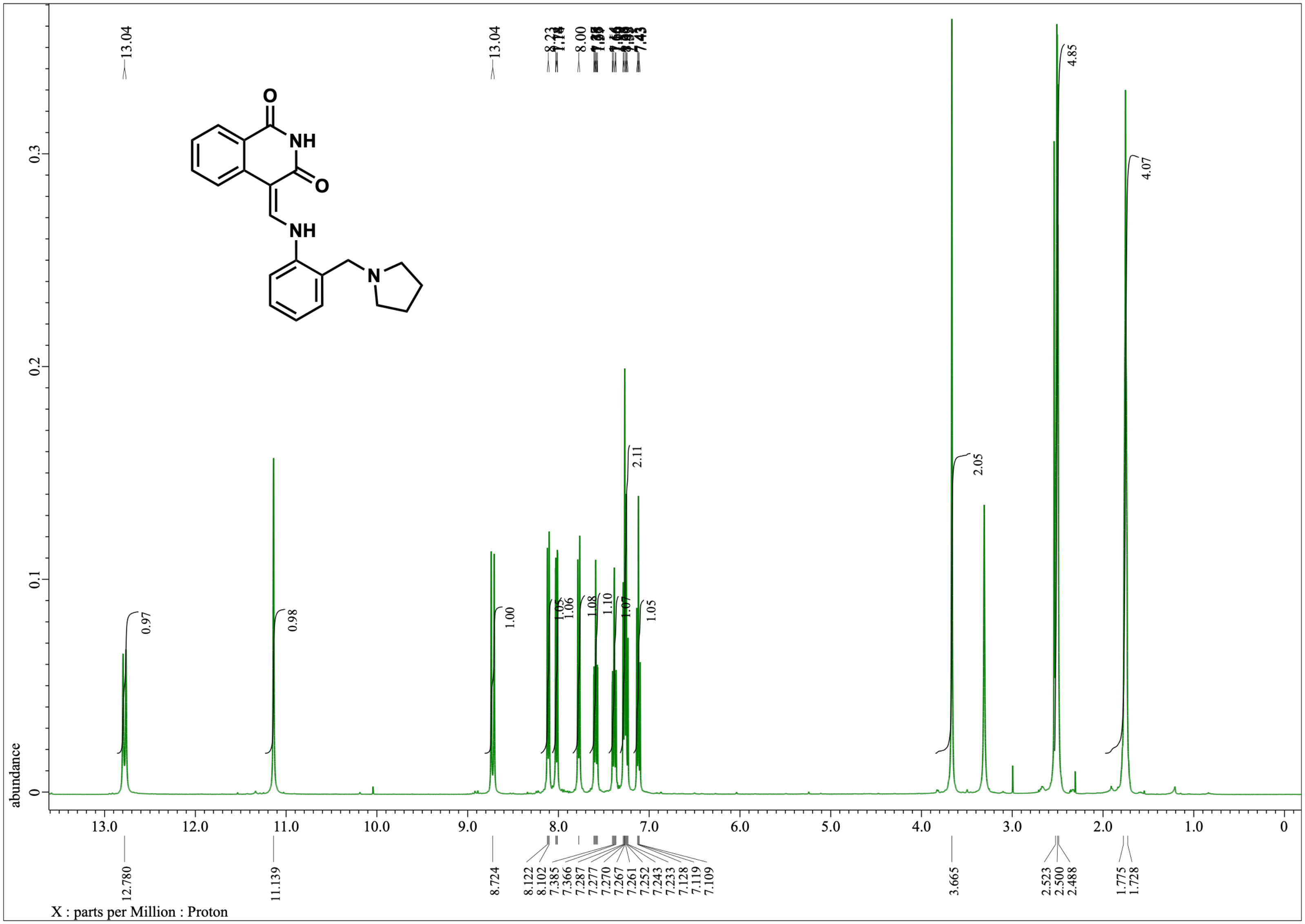

Figure S 57: ^{13}C-NMR (APT) spectra (100 MHz) of ICTR-023 in DMSO-d_6

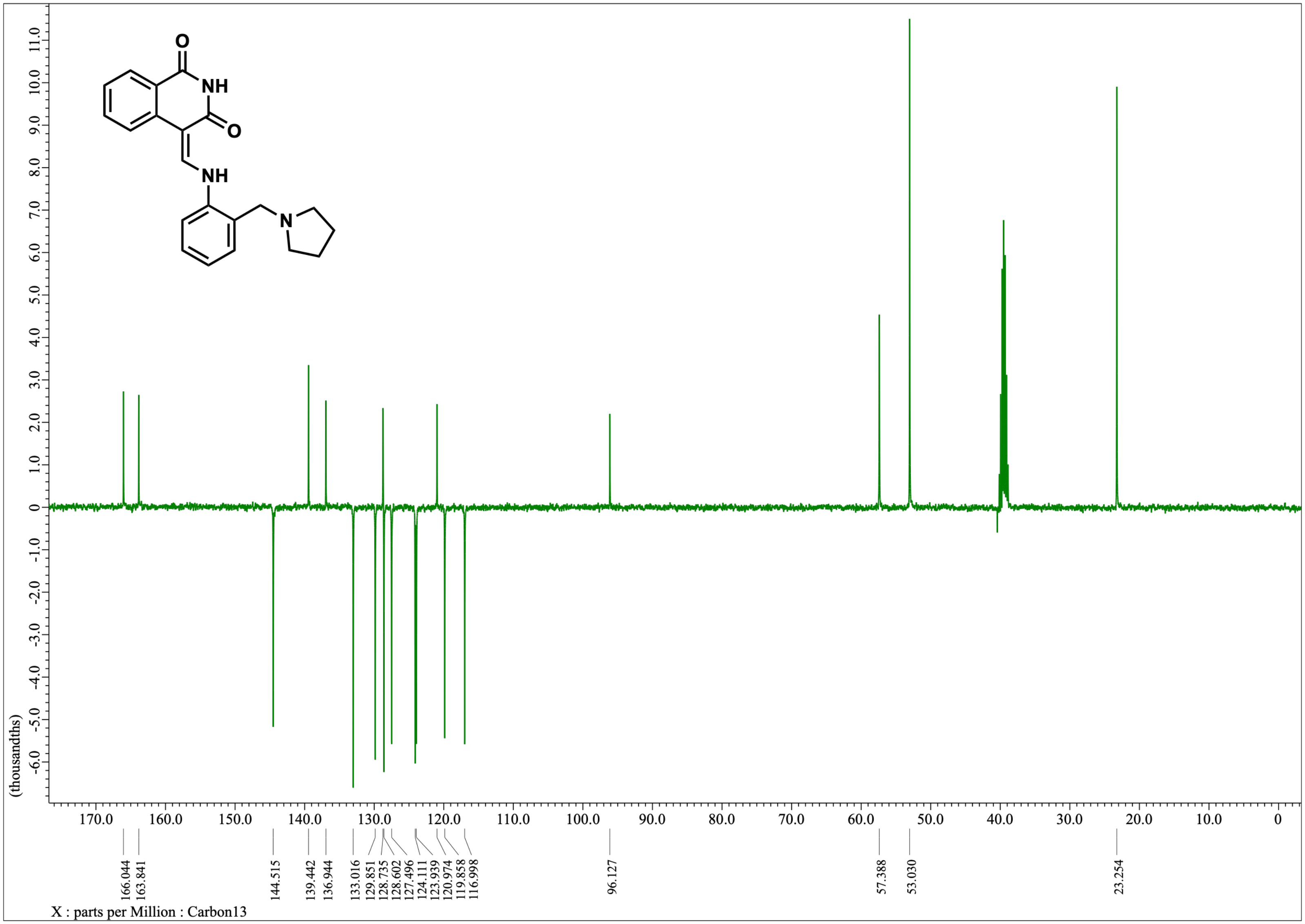

Figure S 58: ^{1}H-NMR spectra (500 MHz) of ICTR-028 in DMSO-d_6

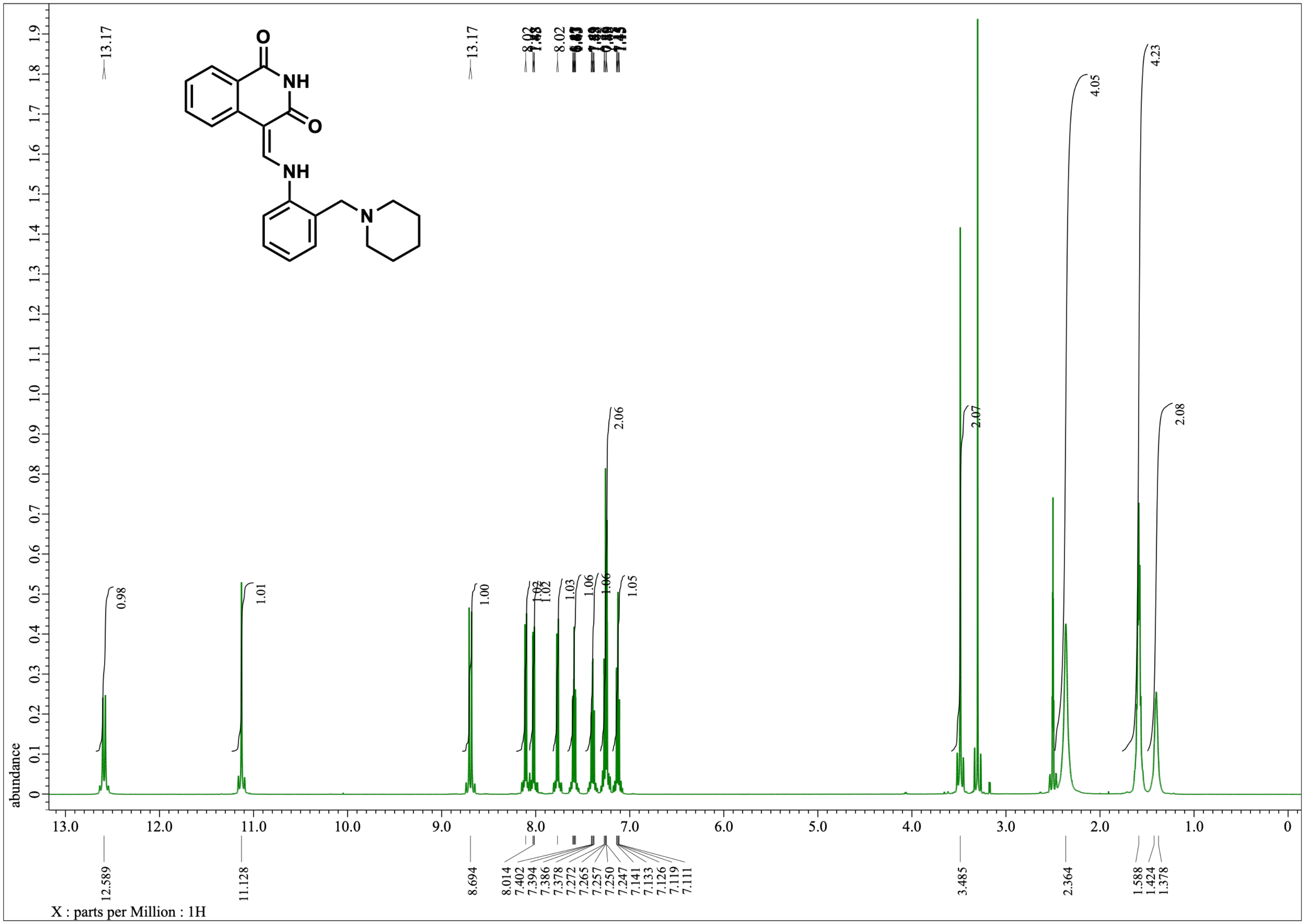

Figure S 59: ^{13}C-NMR (APT) spectra (125 MHz) of ICTR-028 in DMSO-d_6

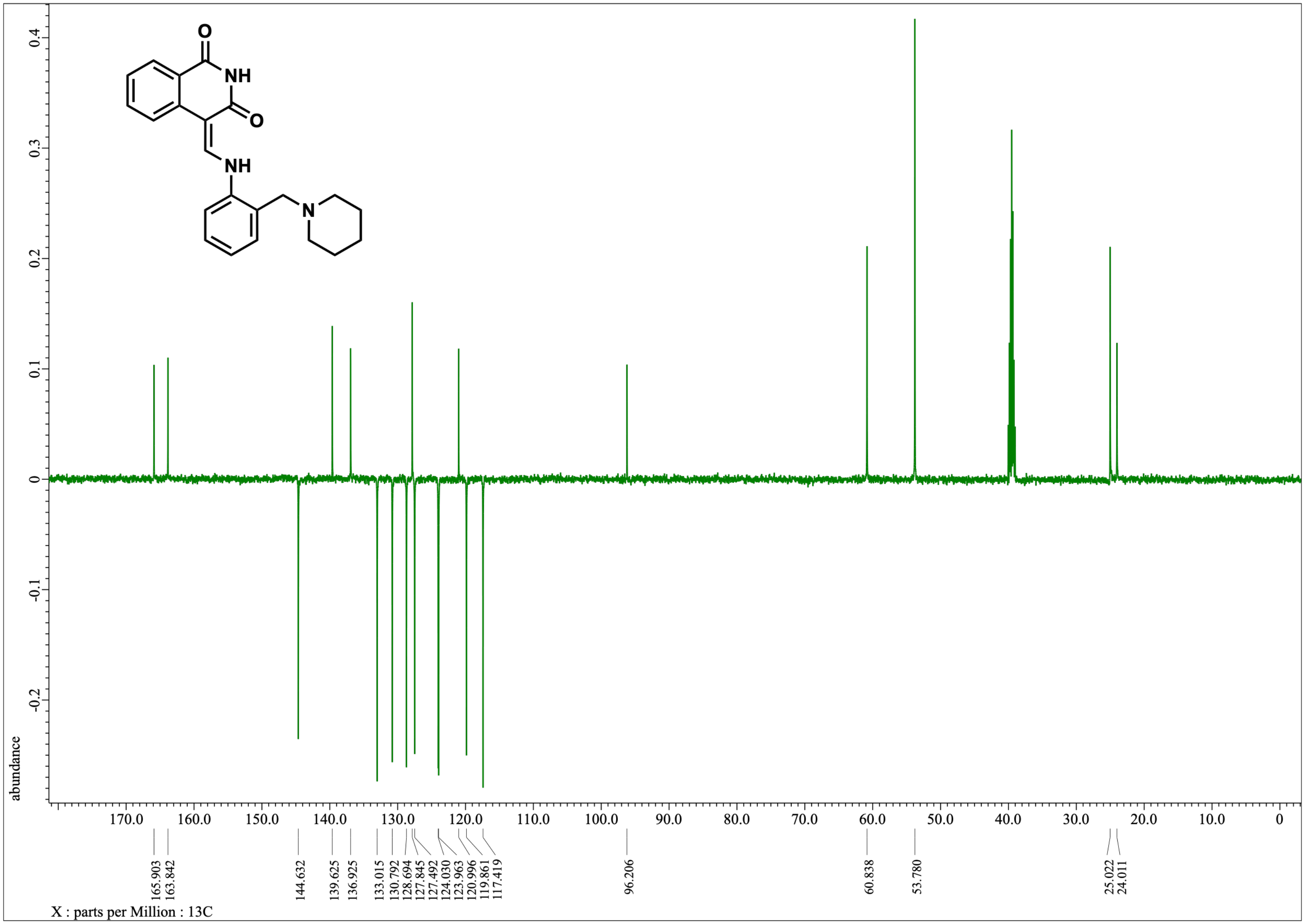

Figure S 60: ^{1}H-NMR spectra (500 MHz) of ICTR-035 in DMSO-d_6

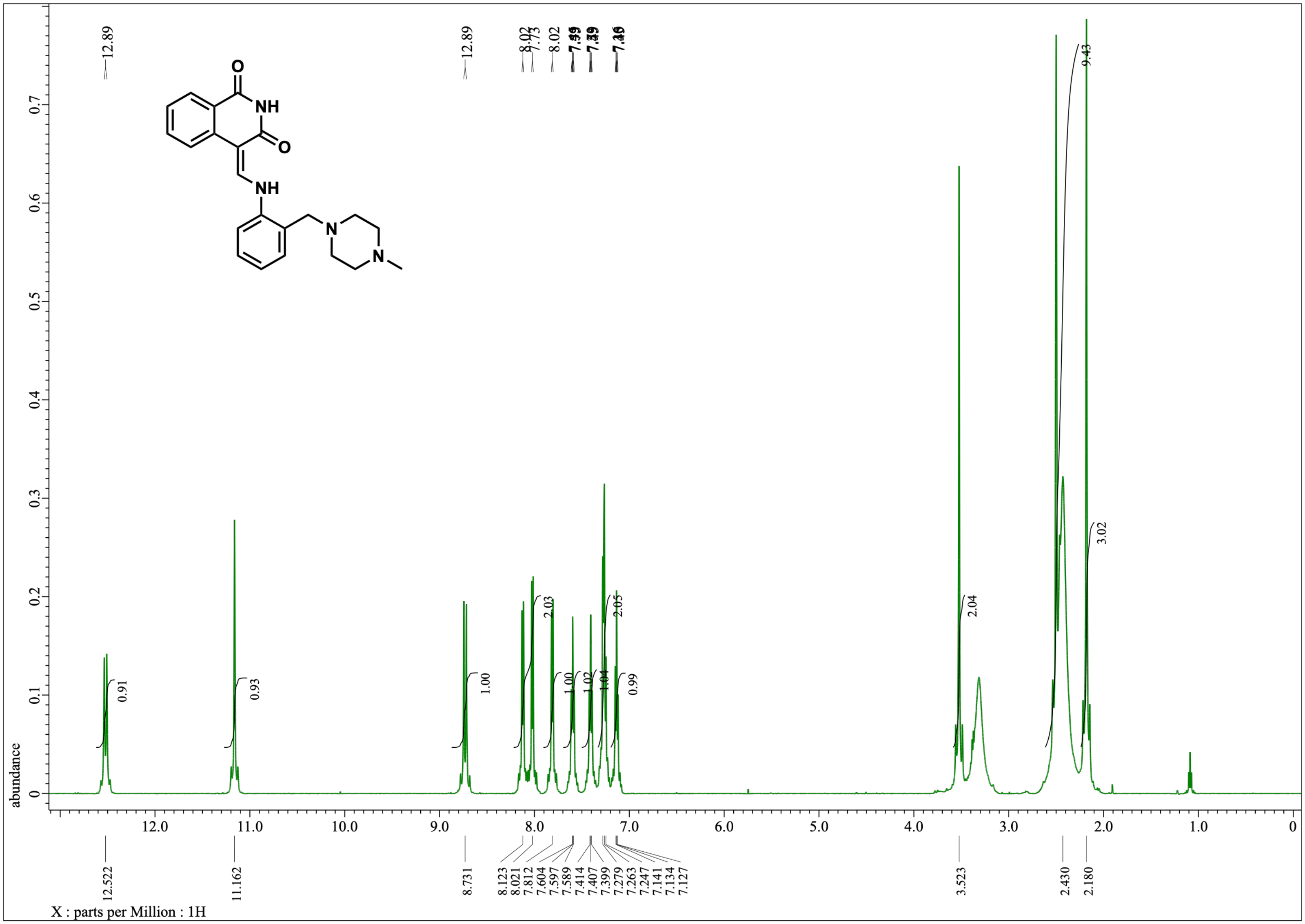

Figure S 61: ^{13}C-NMR (APT) spectra (100 MHz) of ICTR-035 in DMSO-*d₆*

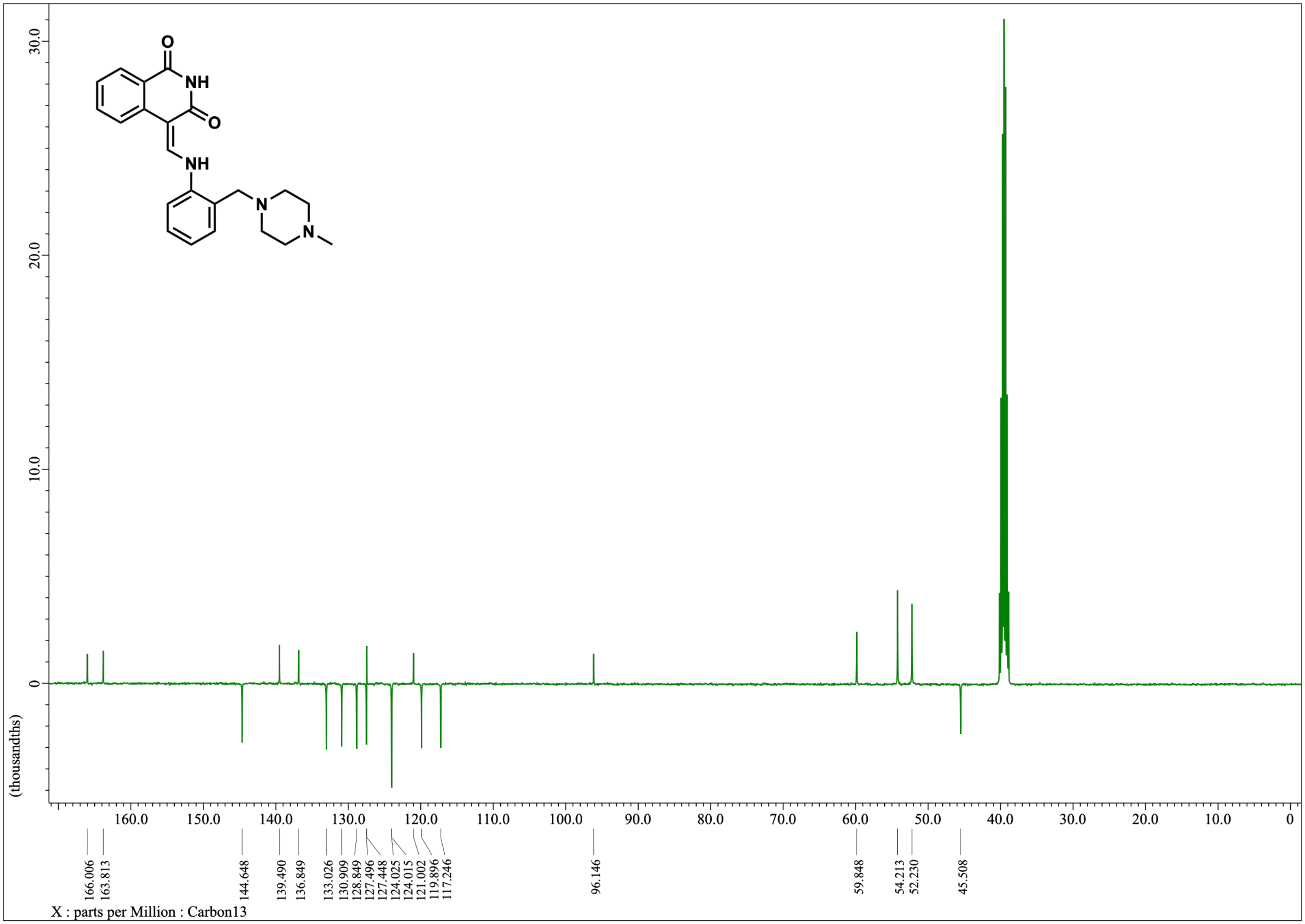

Figure S 62: ^{1}H-NMR spectra (500 MHz) of ICTR-036 in DMSO-d_6

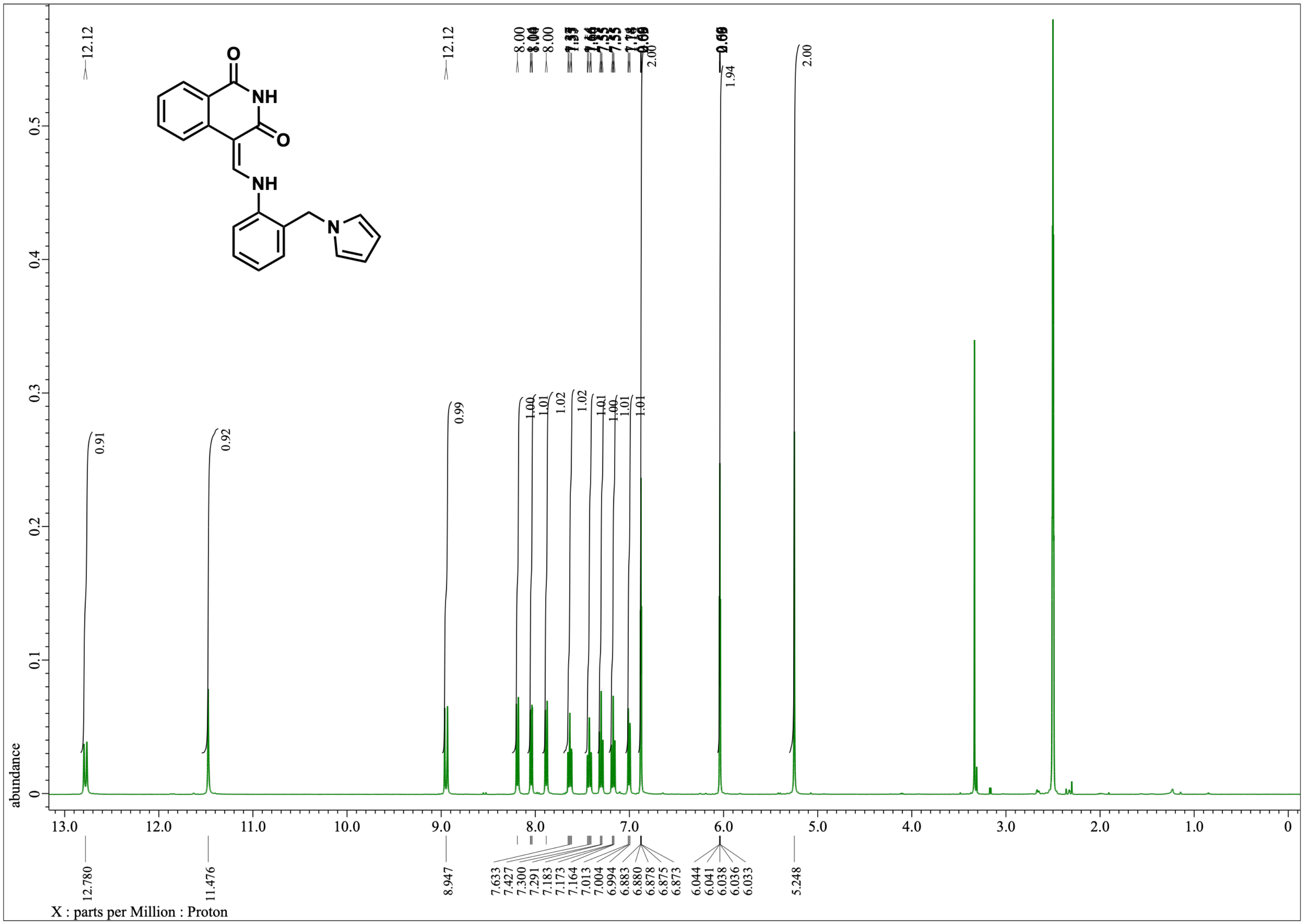

Figure S 63: ^{13}C-NMR (APT) spectra (100 MHz) of ICTR-036 in DMSO-d_6

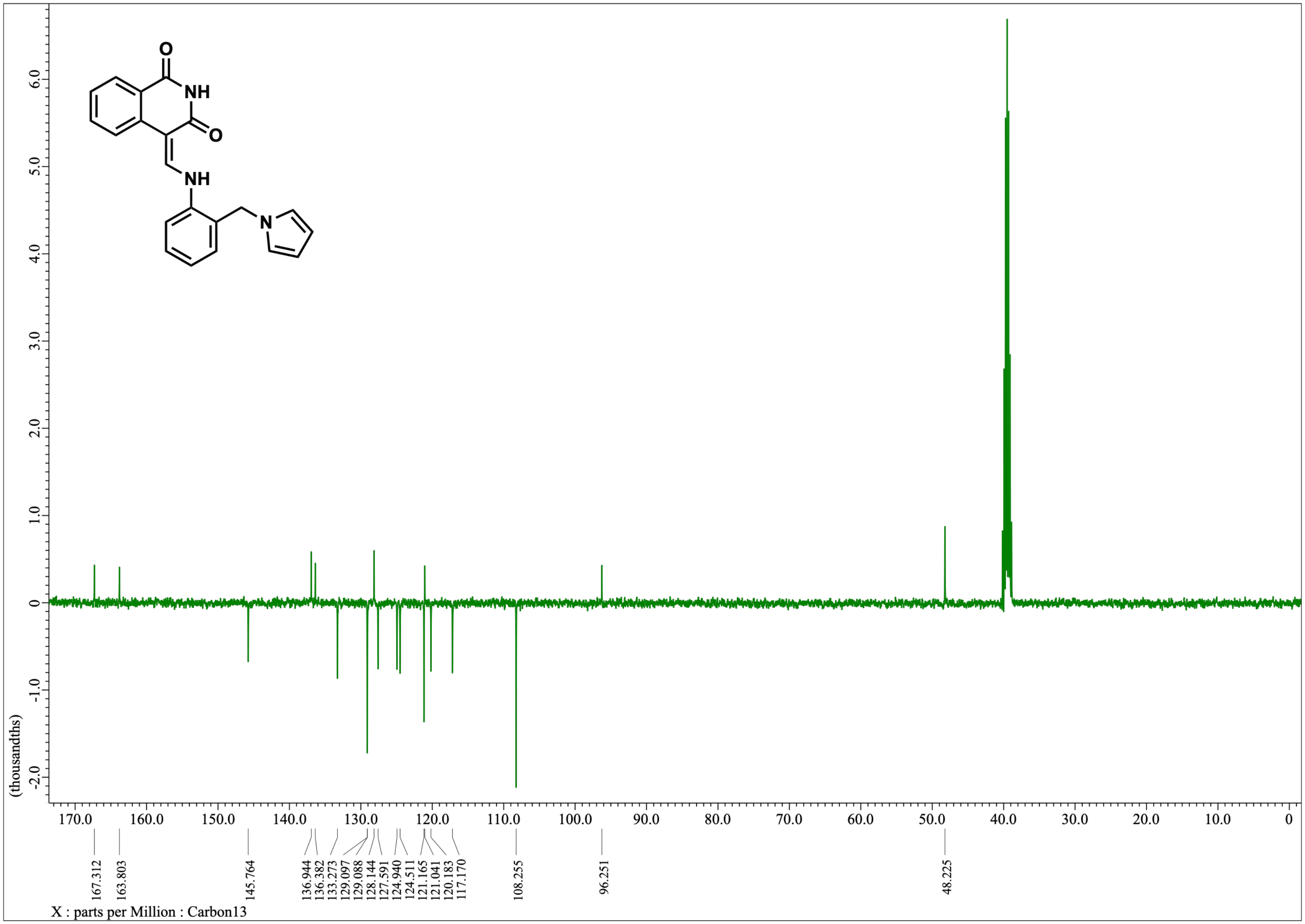

Figure S 64: ^{1}H-NMR spectra (500 MHz) of ICTR-040 in DMSO-d_6

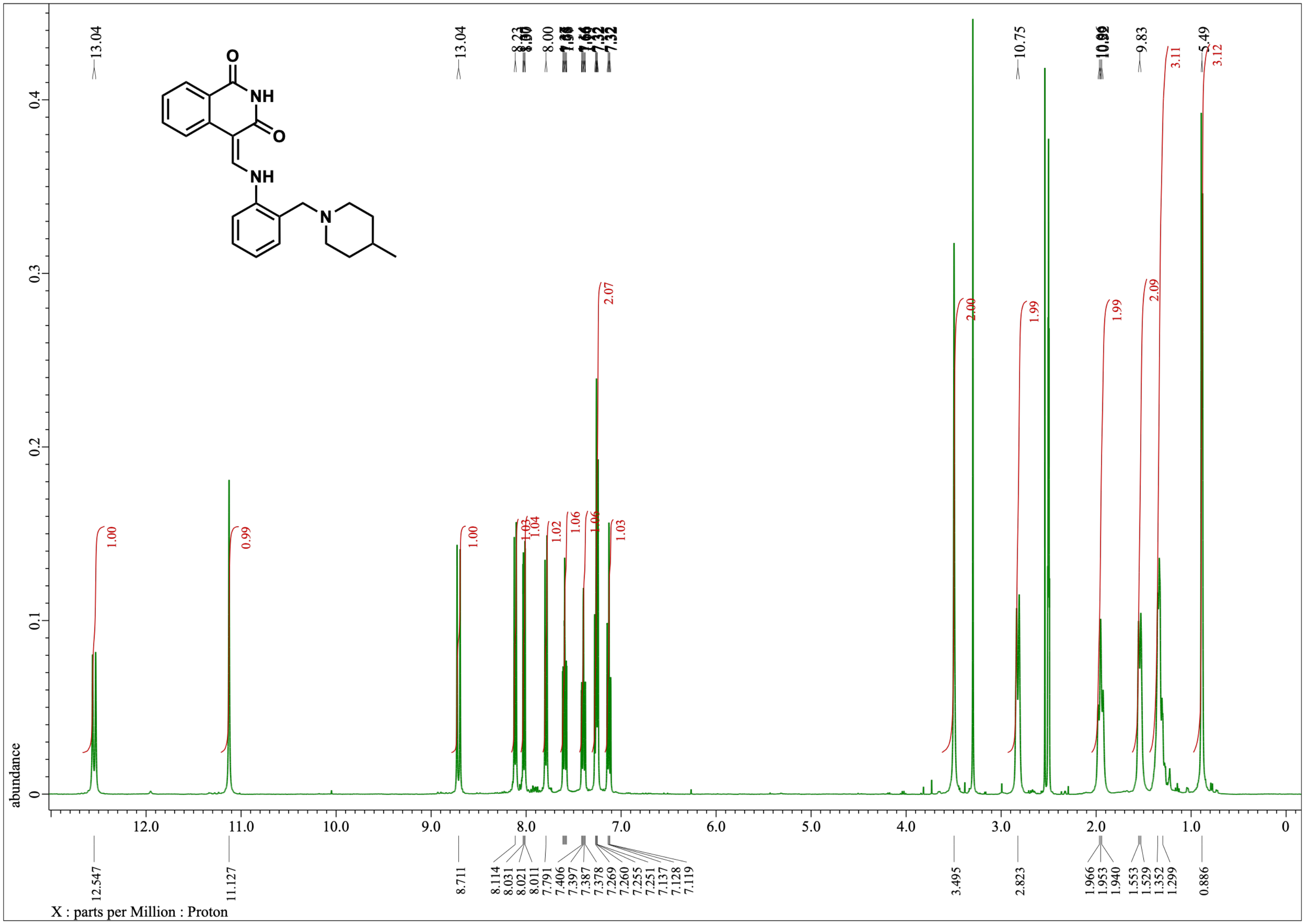

Figure S 65: ^{13}C-NMR (APT) spectra (100 MHz) of ICTR-040 in DMSO-d_6

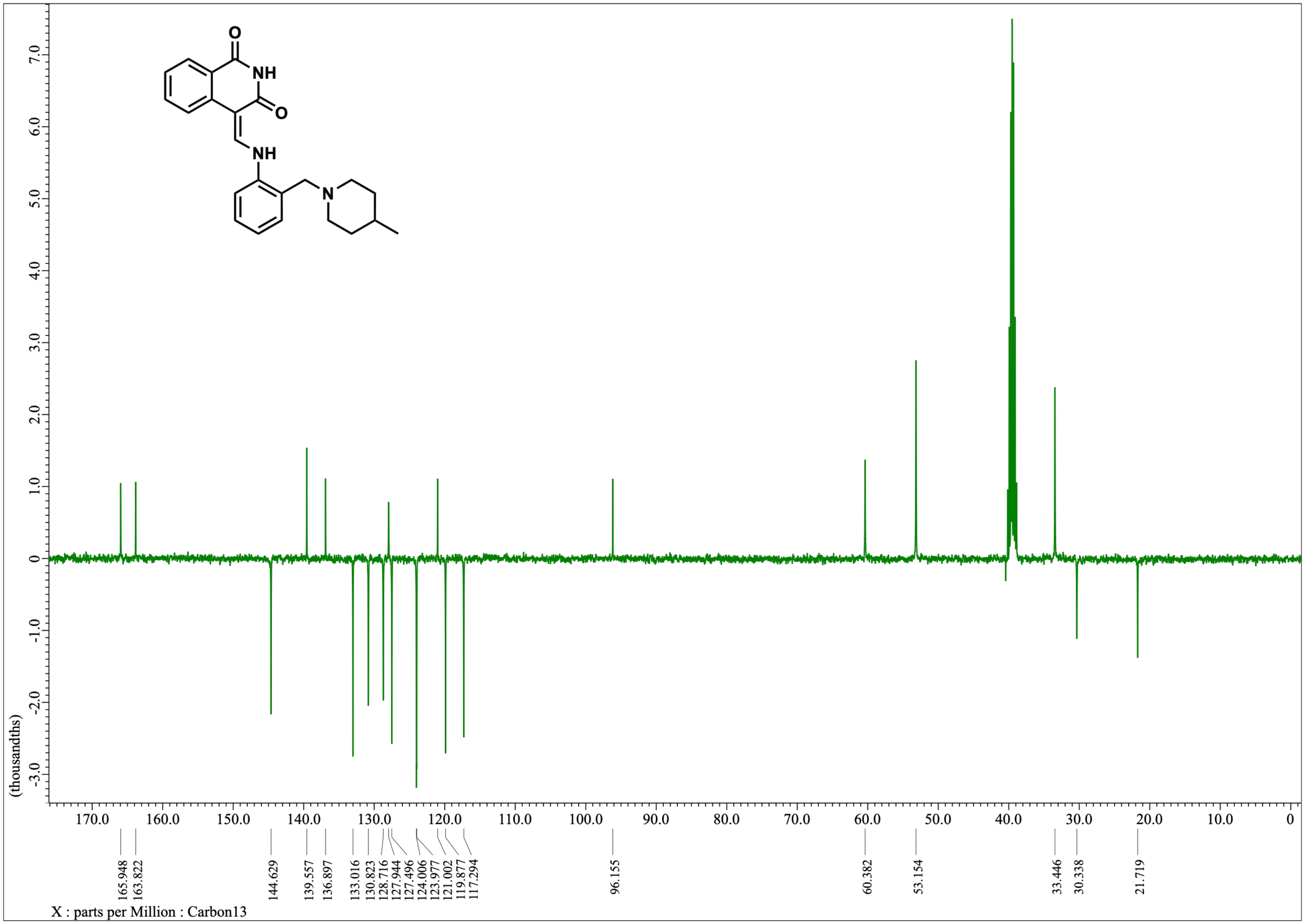

Figure S 66: ^{1}H-NMR spectra (500 MHz) of ICTR-042 in DMSO-d_6

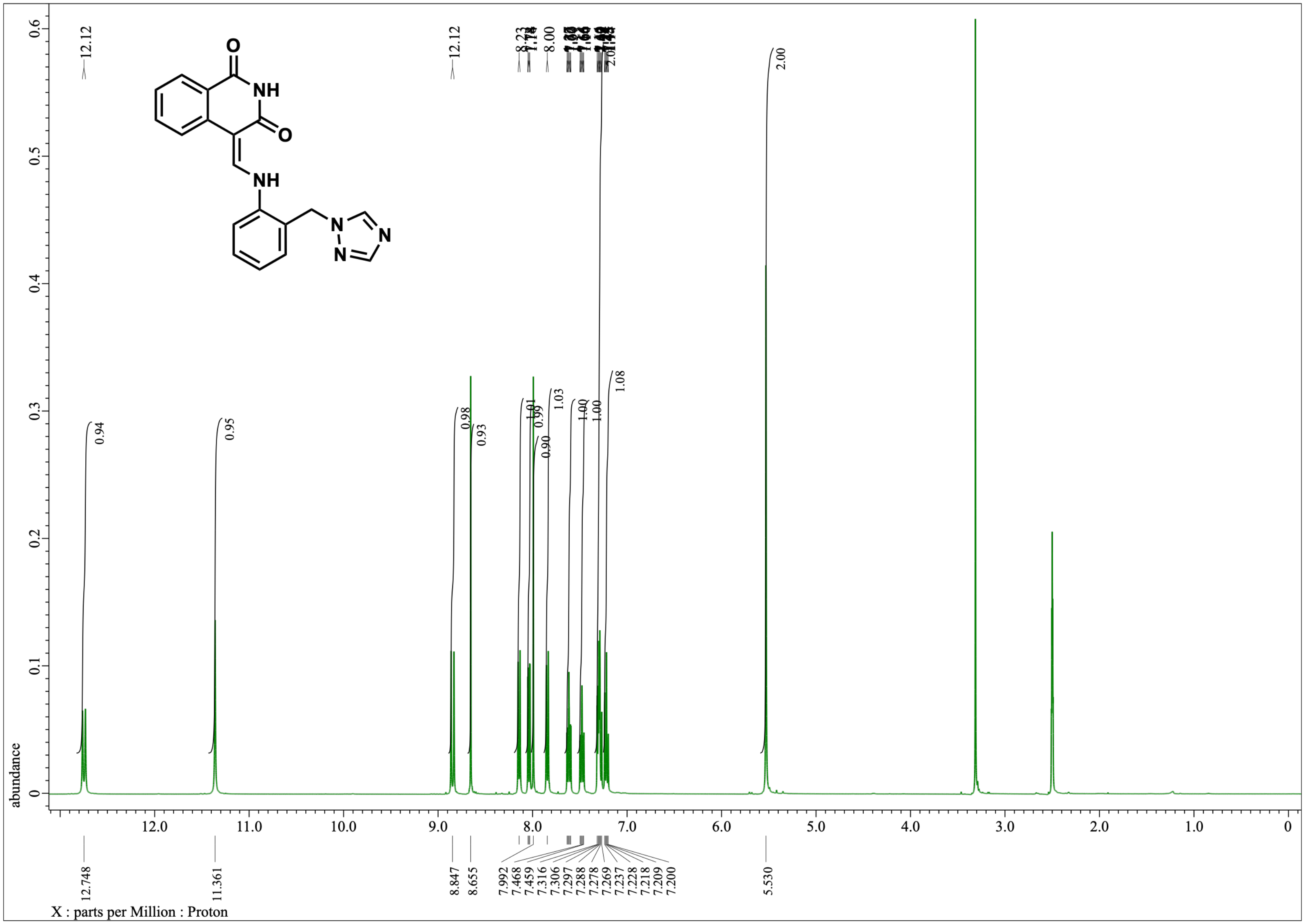

Figure S 67: ^{13}C-NMR (APT) spectra (100 MHz) of ICTR-042 in DMSO-d_6

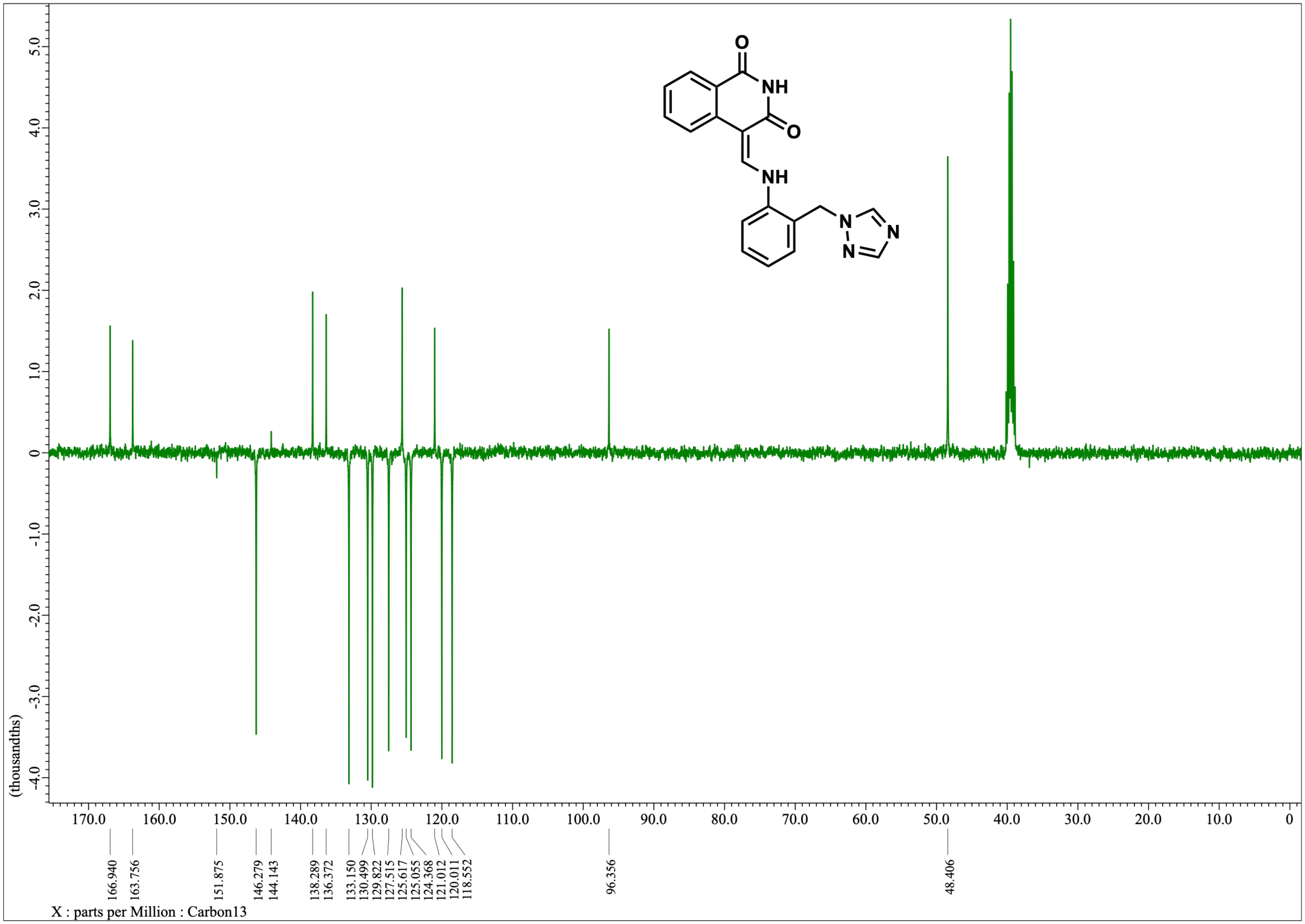

Figure S 68: ^{1}H-NMR spectra (500 MHz) of ICTR-050 in DMSO-*d₆*

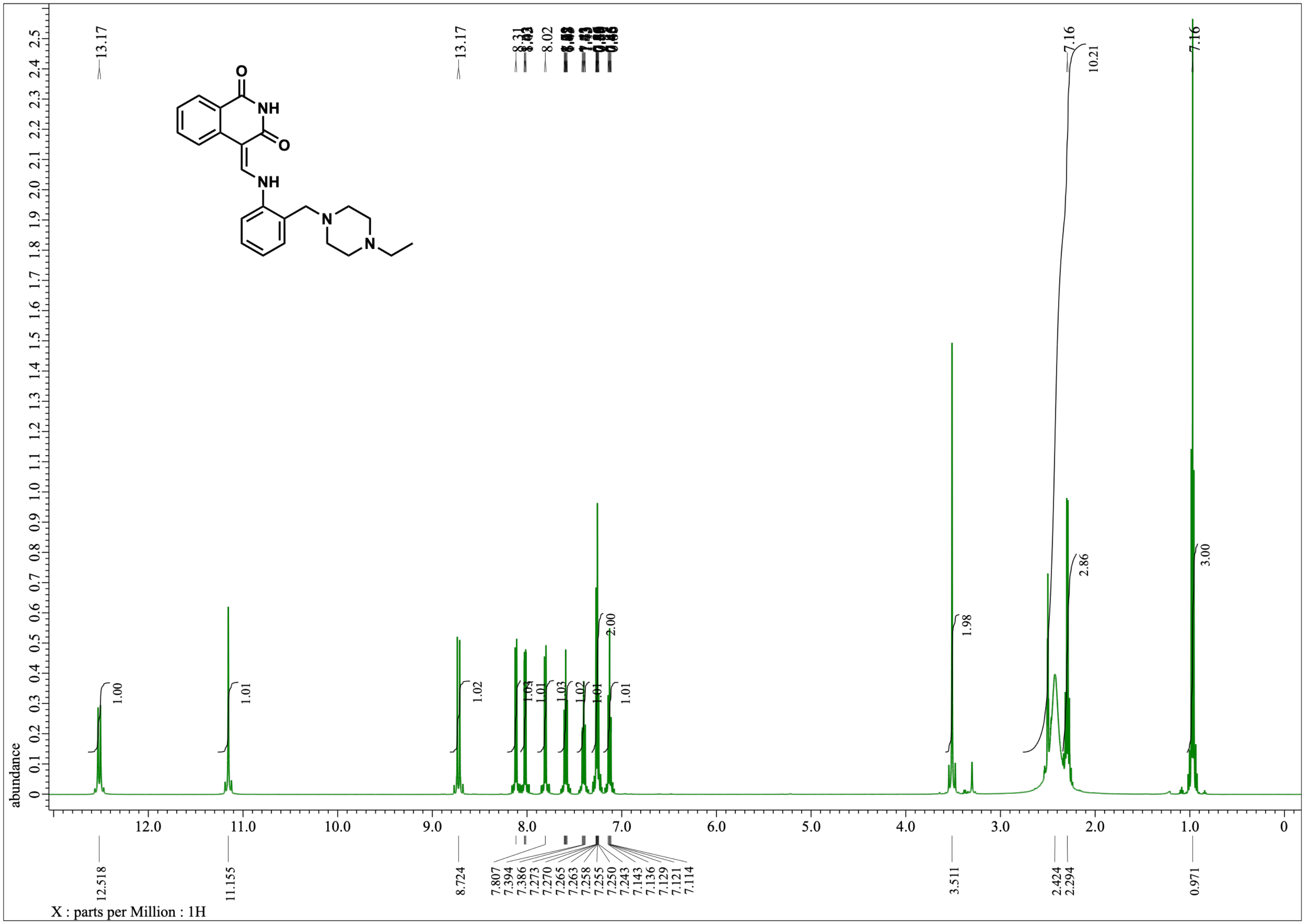

Figure S 69: ^{13}C-NMR (APT) spectra (125 MHz) of ICTR-050 in DMSO-*d_6*

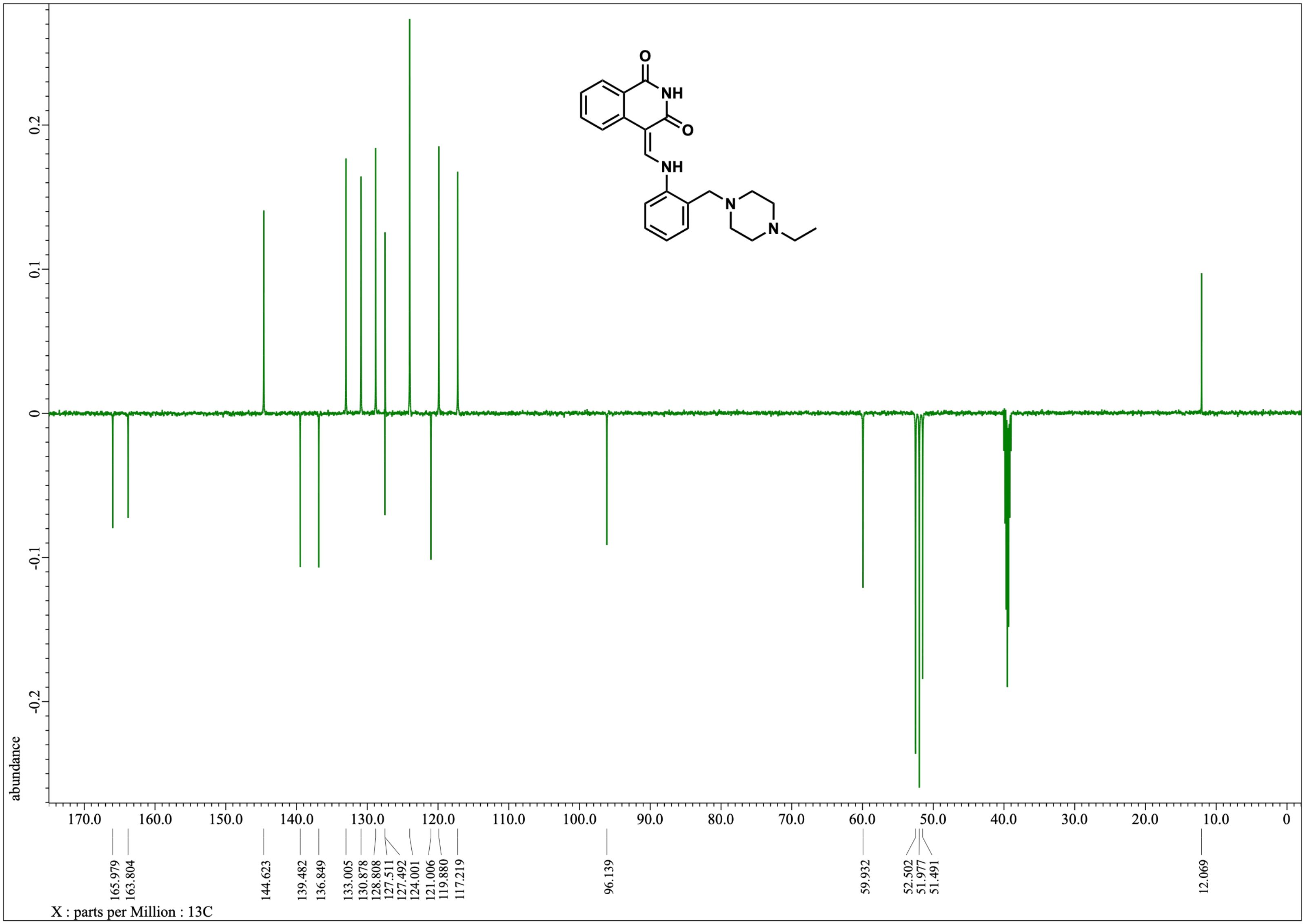

Figure S 70: ^{1}H-NMR spectra (500 MHz) of ICTR-052 in DMSO-d_6

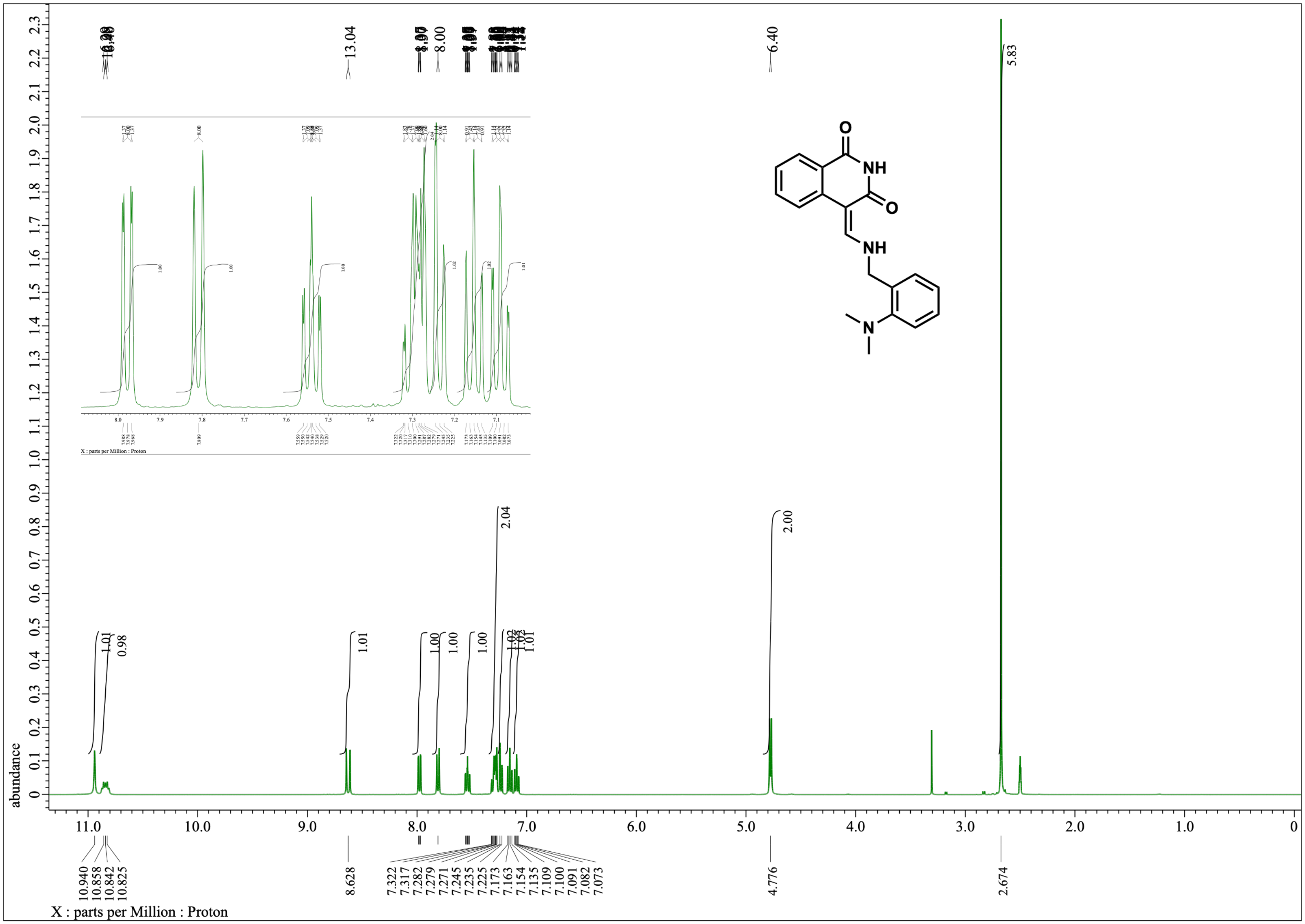

Figure S 71: ^{13}C-NMR (APT) spectra (100 MHz) of ICTR-052 in DMSO-*d_6*

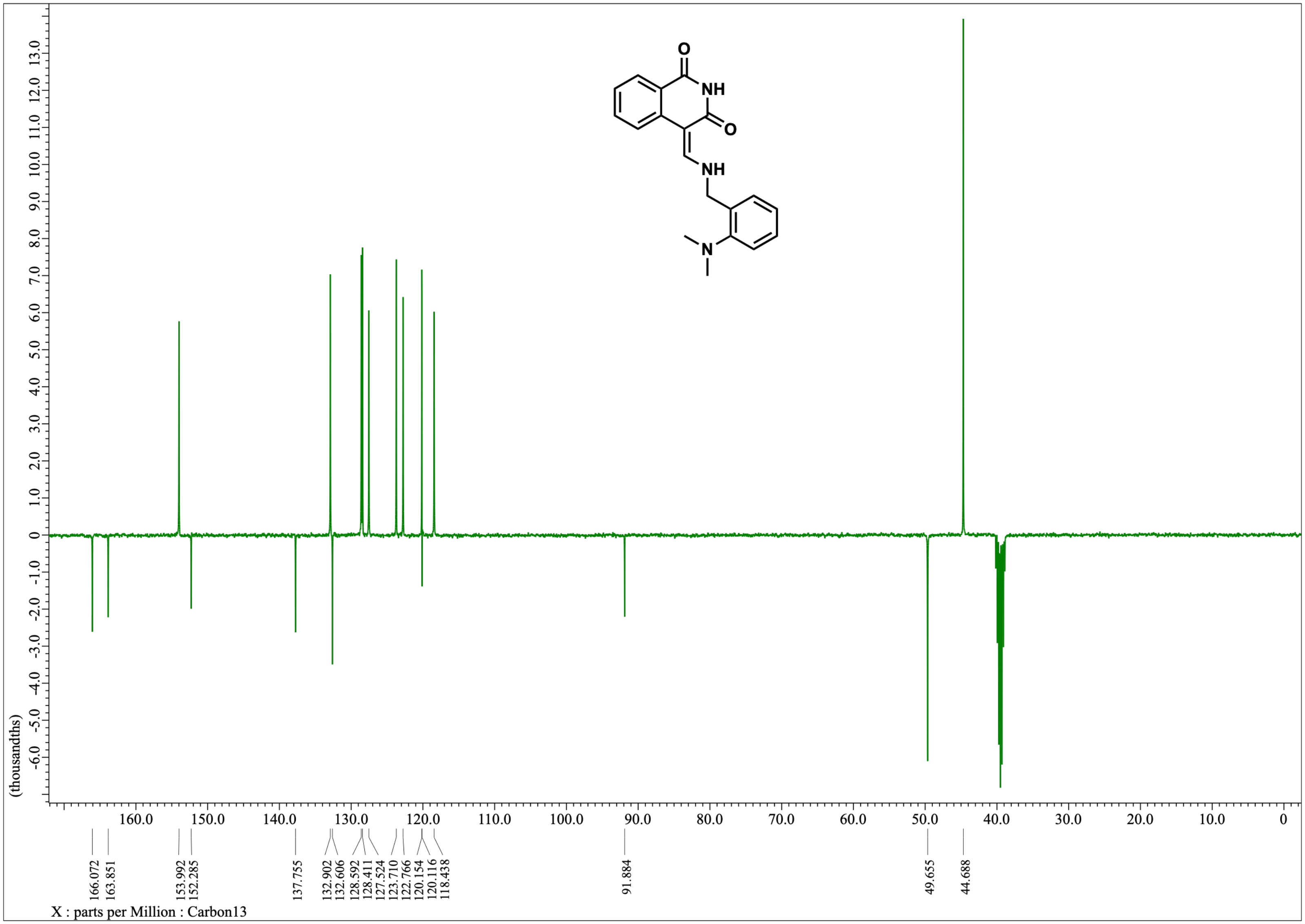

Figure S 72: ^{1}H-NMR spectra (500 MHz) of ICTR-053 in DMSO-d_6

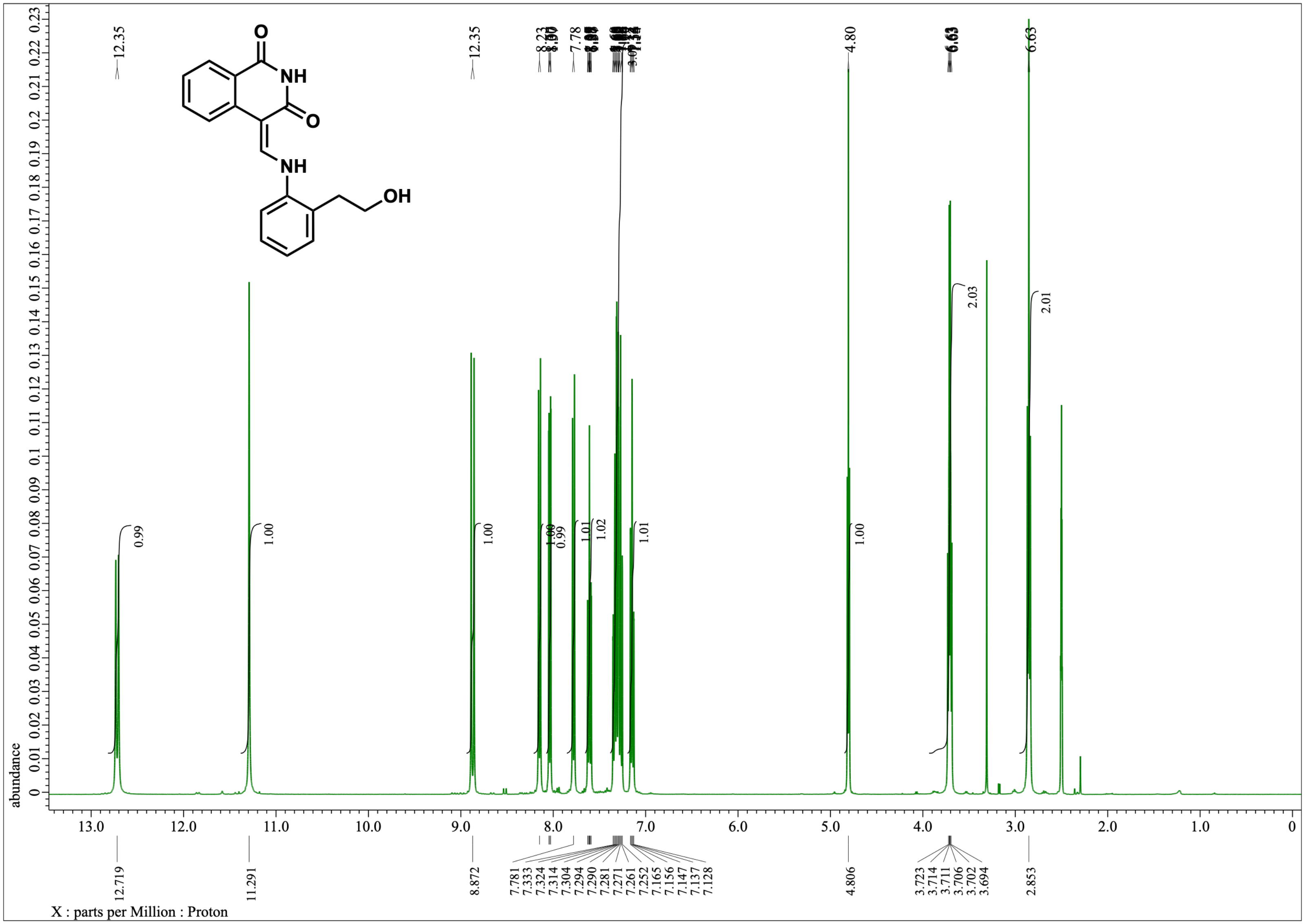

Figure S 73: ^{13}C-NMR (APT) spectra (100 MHz) of ICTR-053 in DMSO-d_6

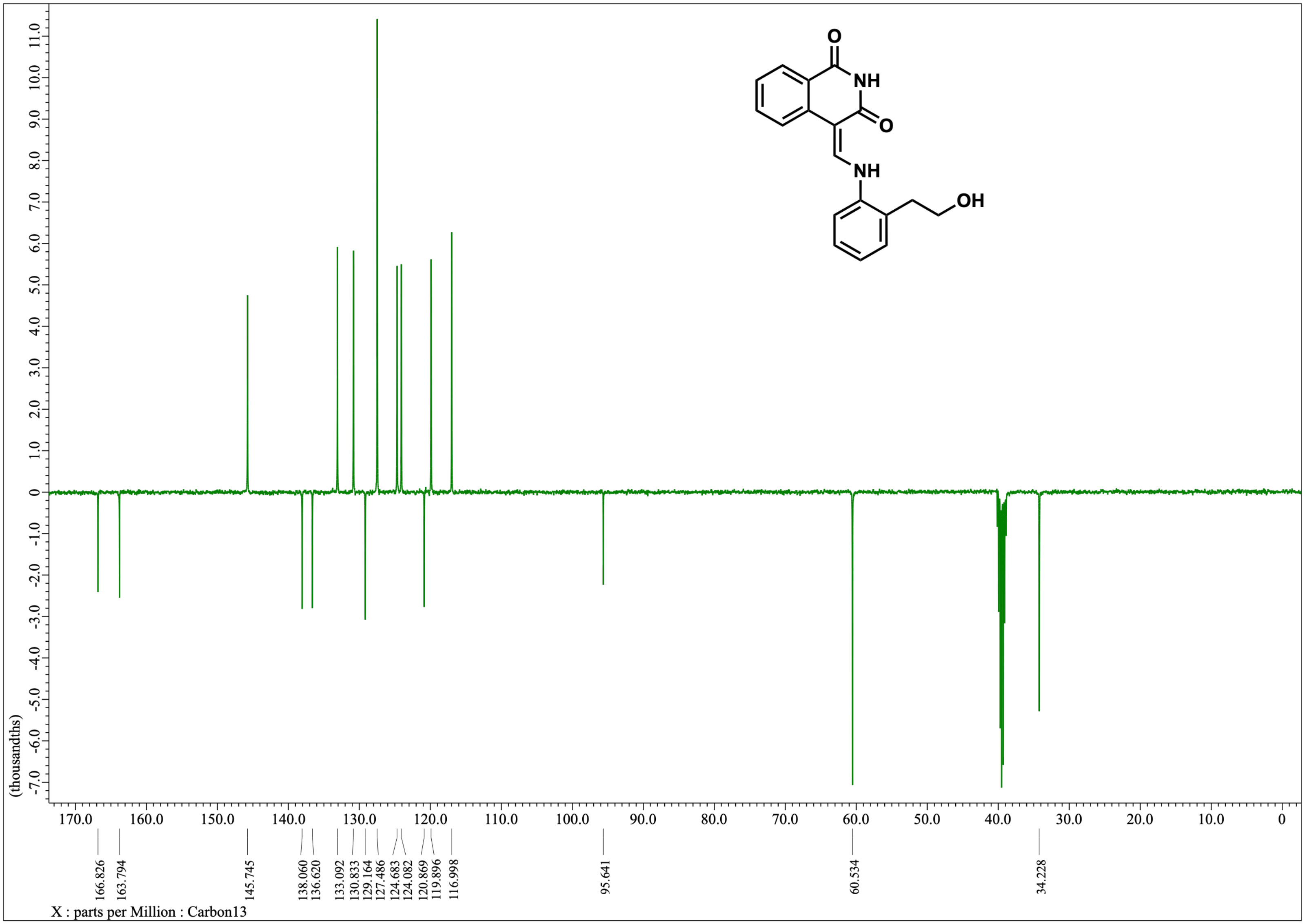

Figure S 74: ^{1}H-NMR spectra (500 MHz) of ICTR-058*HCl in DMSO-d_6

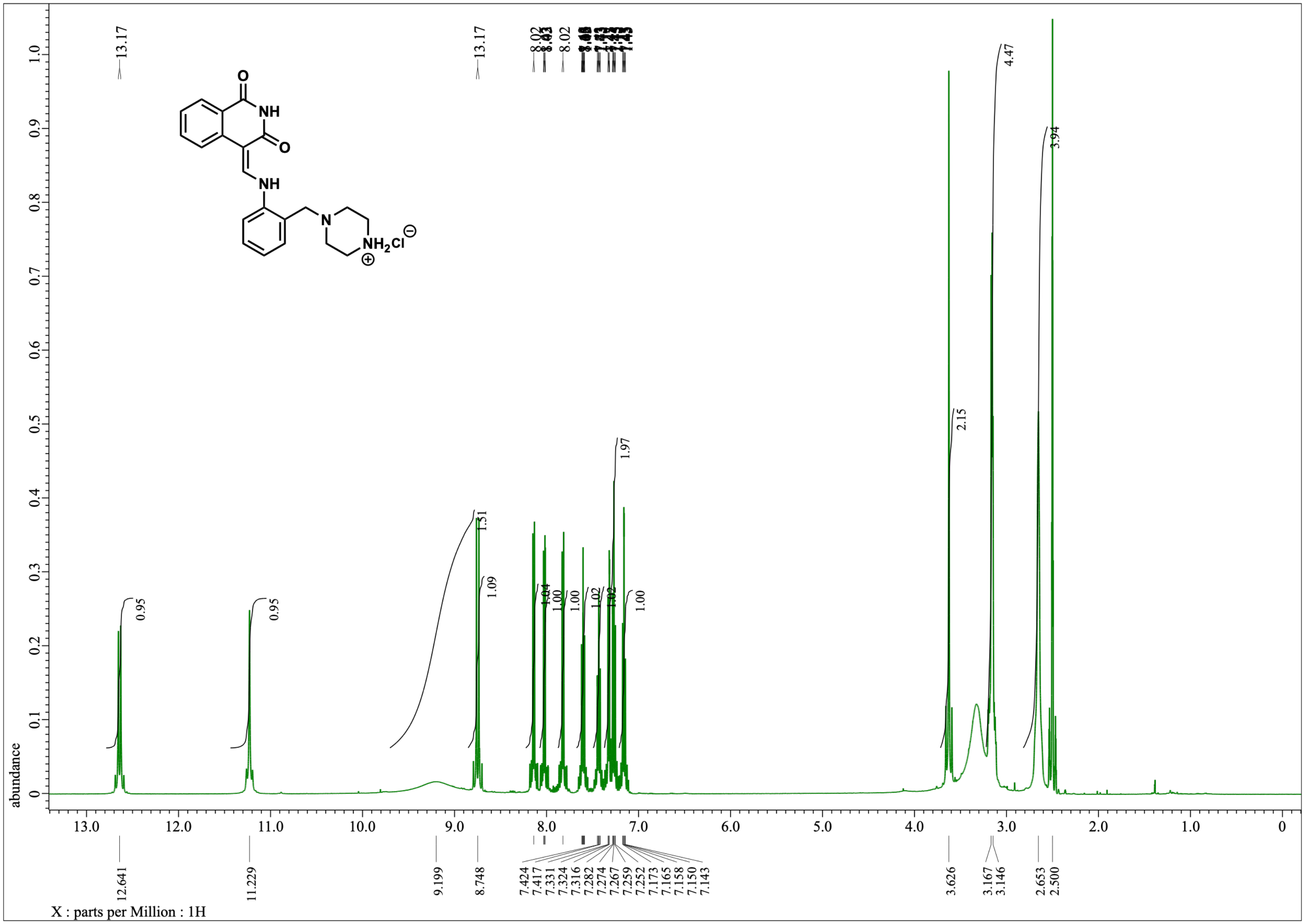

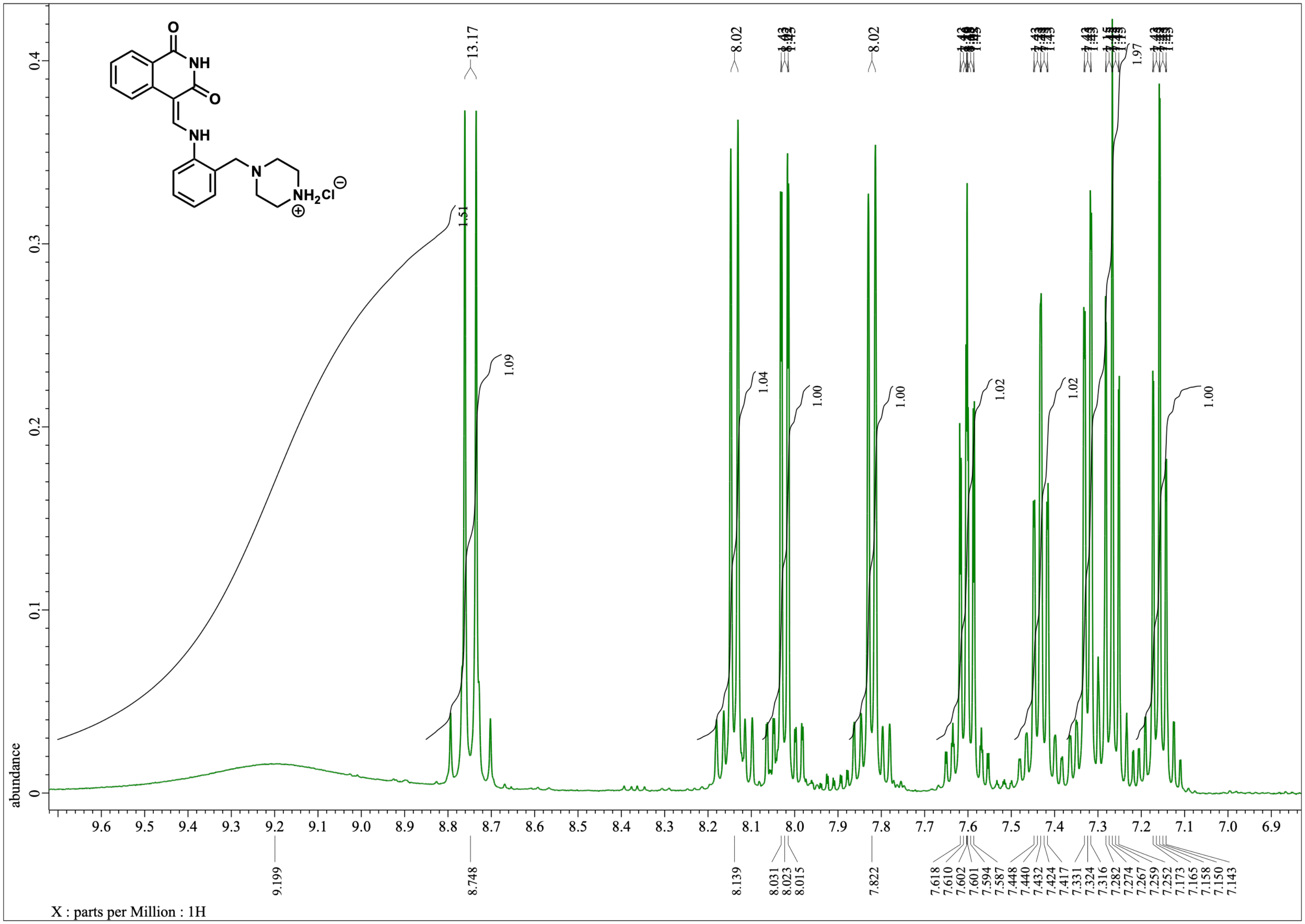

abundance
X : parts per Million : 1H
9.199
8.748
8.139
8.031
8.023
8.015
7.822
7.618
7.610
7.602
7.601
7.594
7.587
7.448
7.440
7.432
7.424
7.417
7.331
7.324
7.316
7.282
7.274
7.267
7.259
7.252
7.173
7.165
7.158
7.150
7.143
1.51
1.09
1.04
1.00
1.00
1.02
1.02
1.97
1.00

Figure S 75: ^{13}C-NMR (APT) spectra (125 MHz) of ICTR-058*vHCl in DMSO-d_6

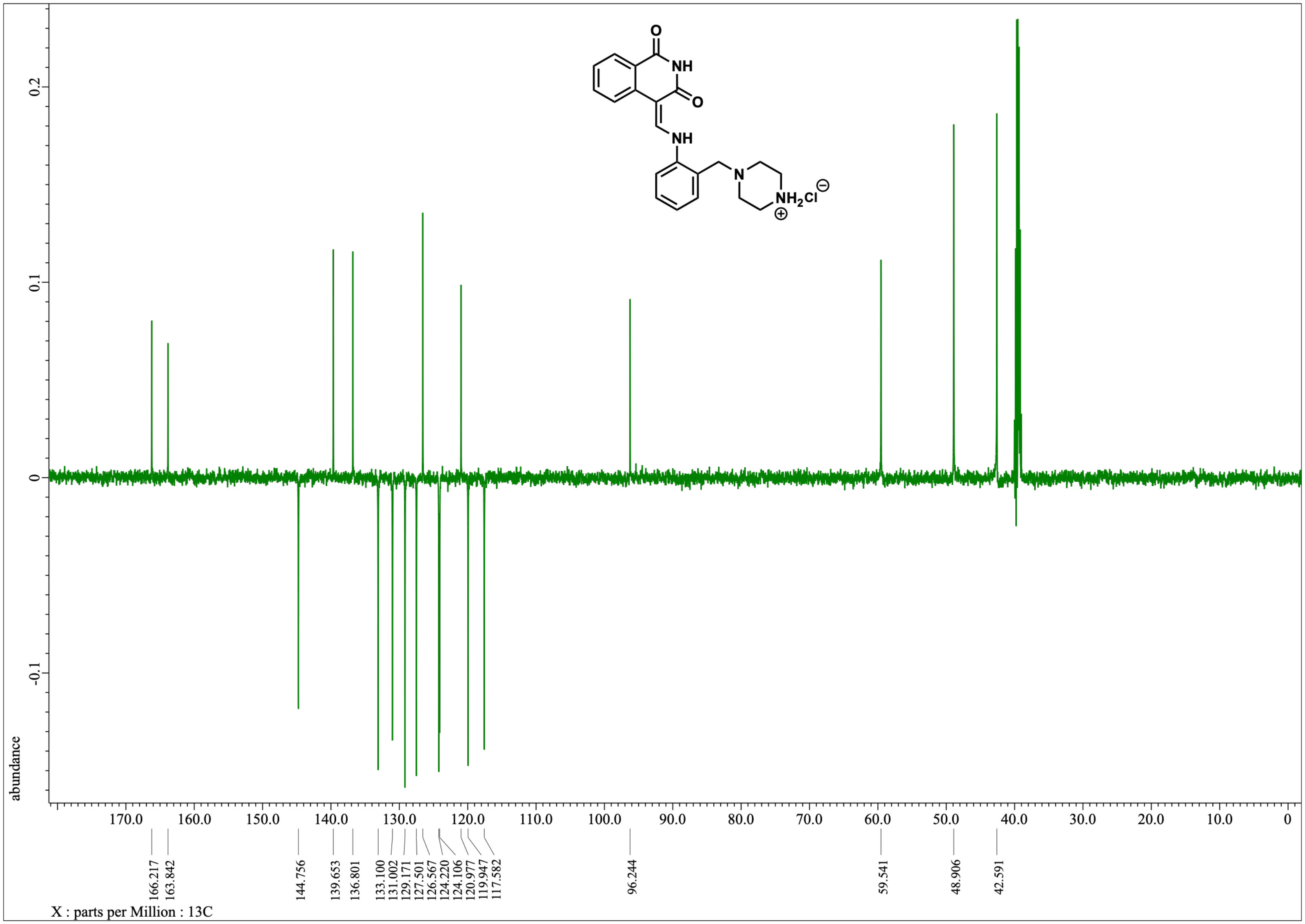

Figure S 76: ^{1}H-NMR spectra (500 MHz) of ICTR-059*HCl in DMSO-d_6

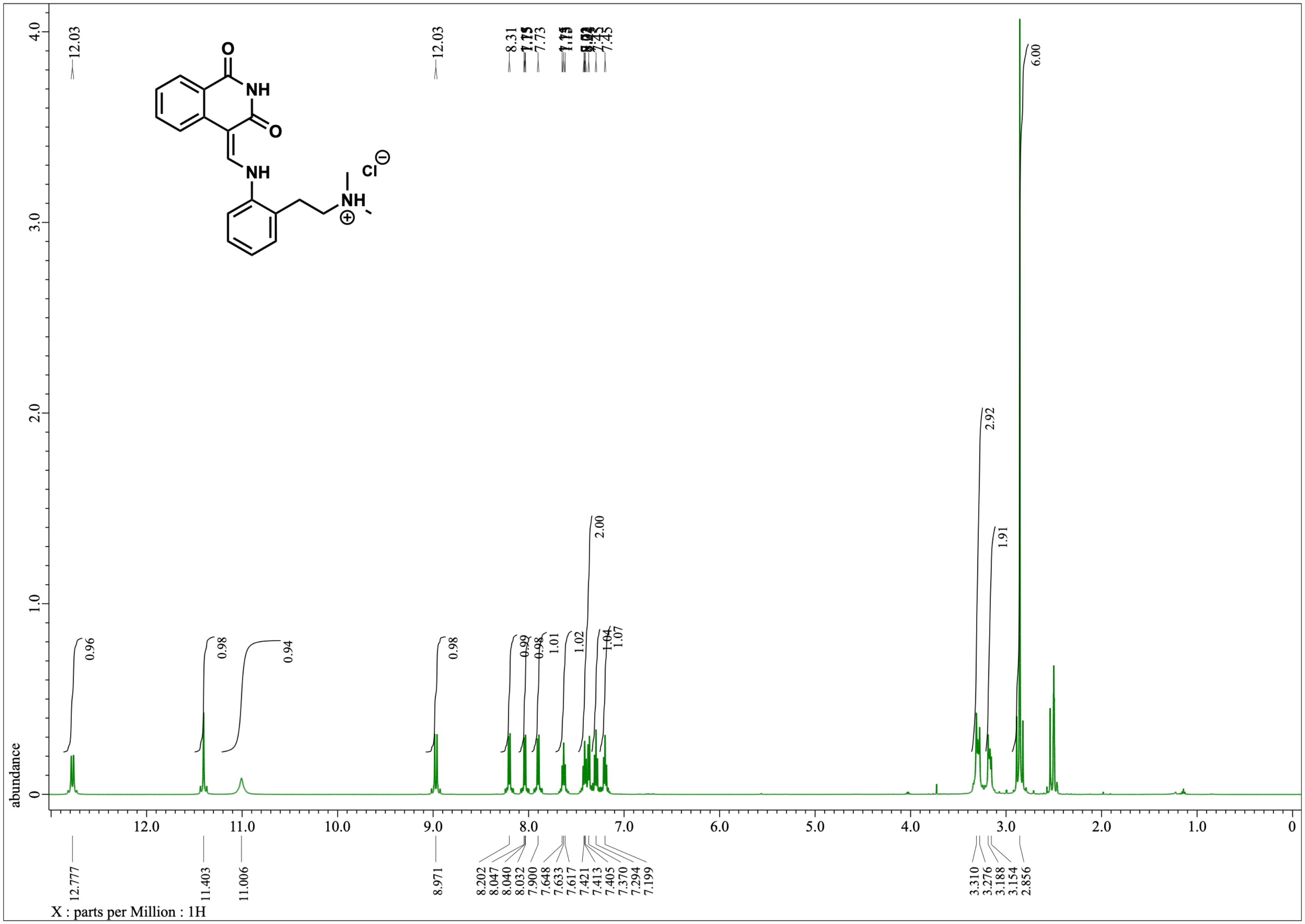

Figure S 77: ^{13}C-NMR (APT) spectra (125 MHz) of ICTR-059*HCl in DMSO-d_6

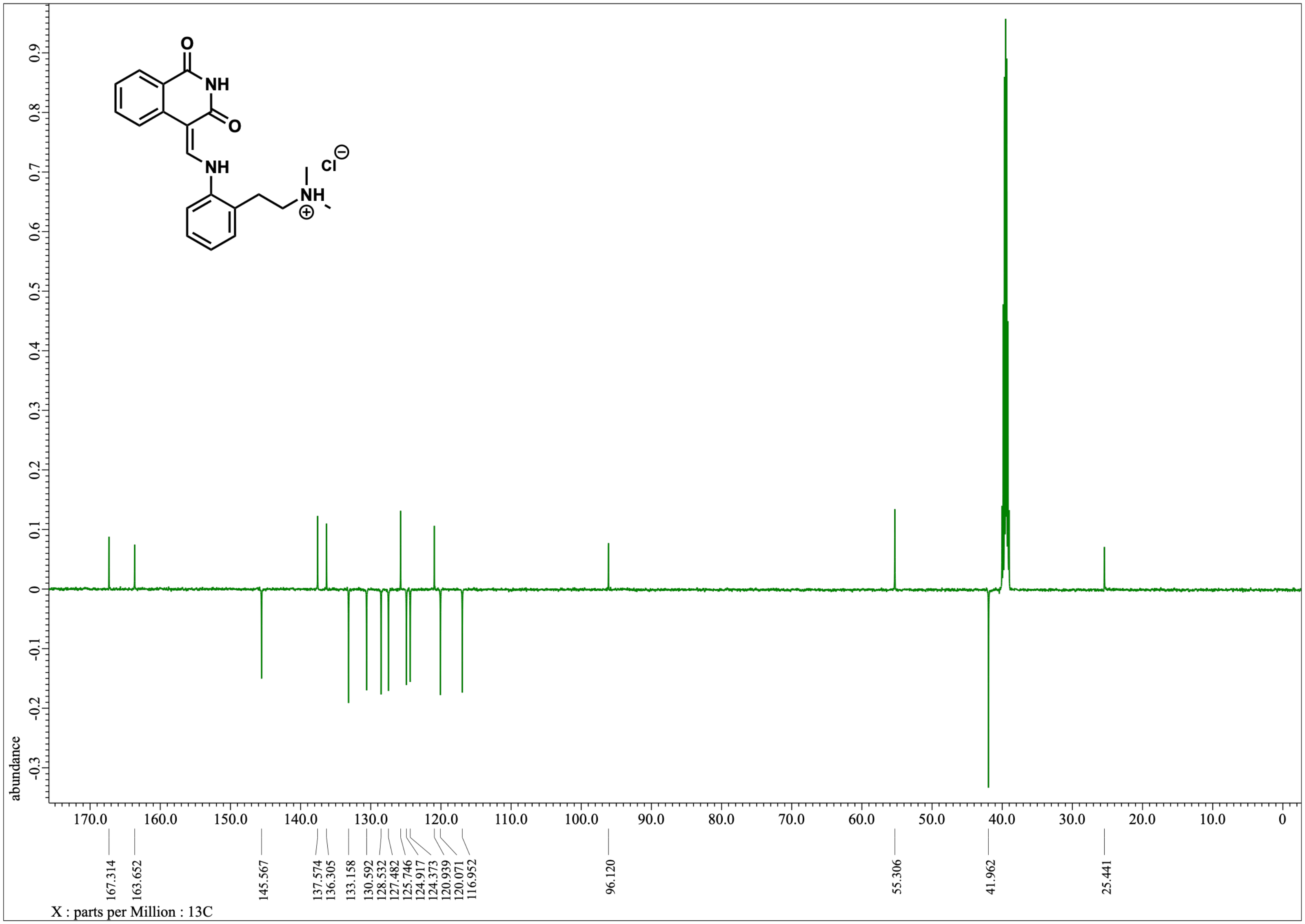

Figure S 78: 1H-NMR spectra (500 MHz) of ICTR-060 in DMSO-d_6

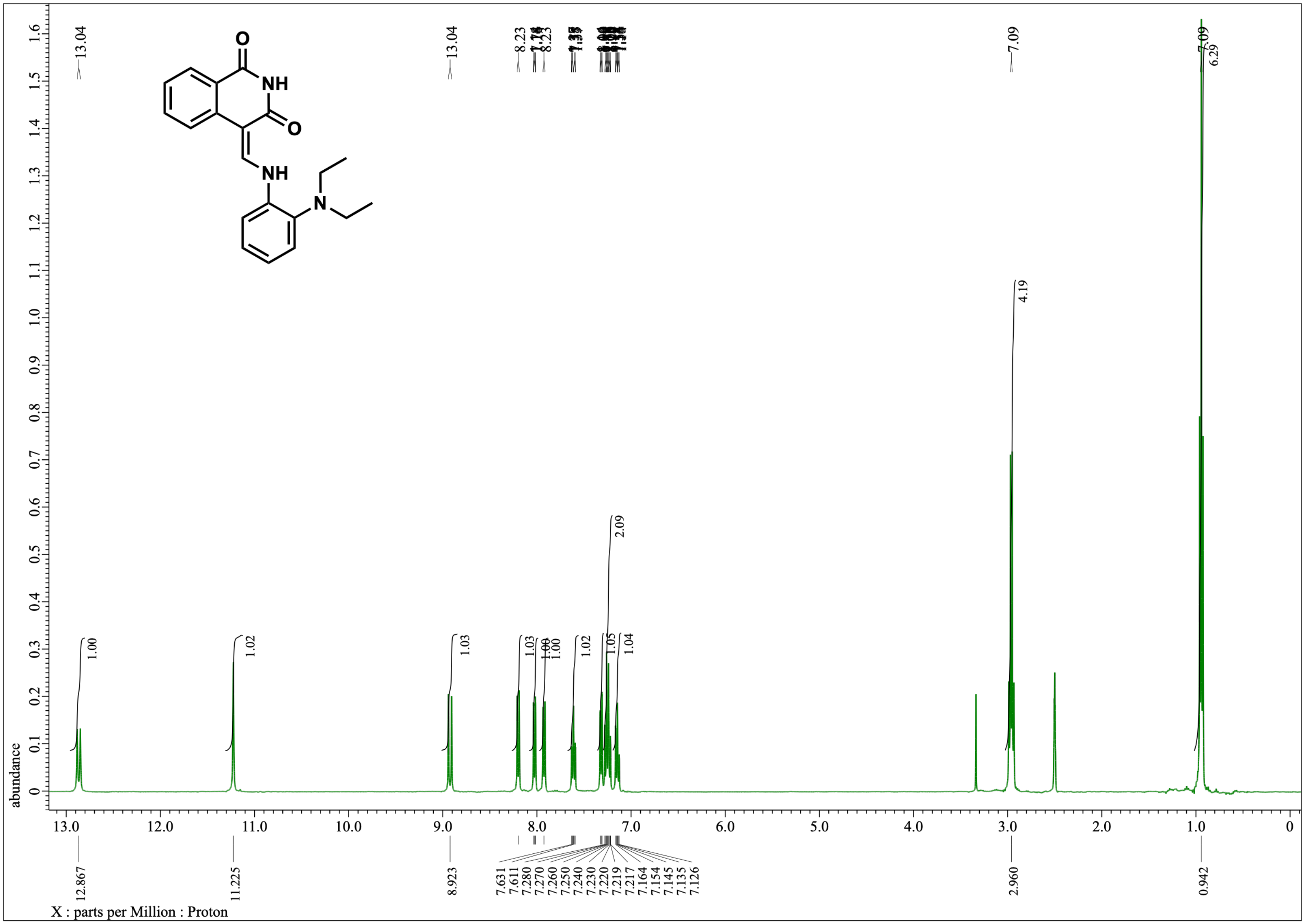

Figure S 79: ^{13}C-NMR (APT) spectra (100 MHz) of ICTR-060 in DMSO-d_6

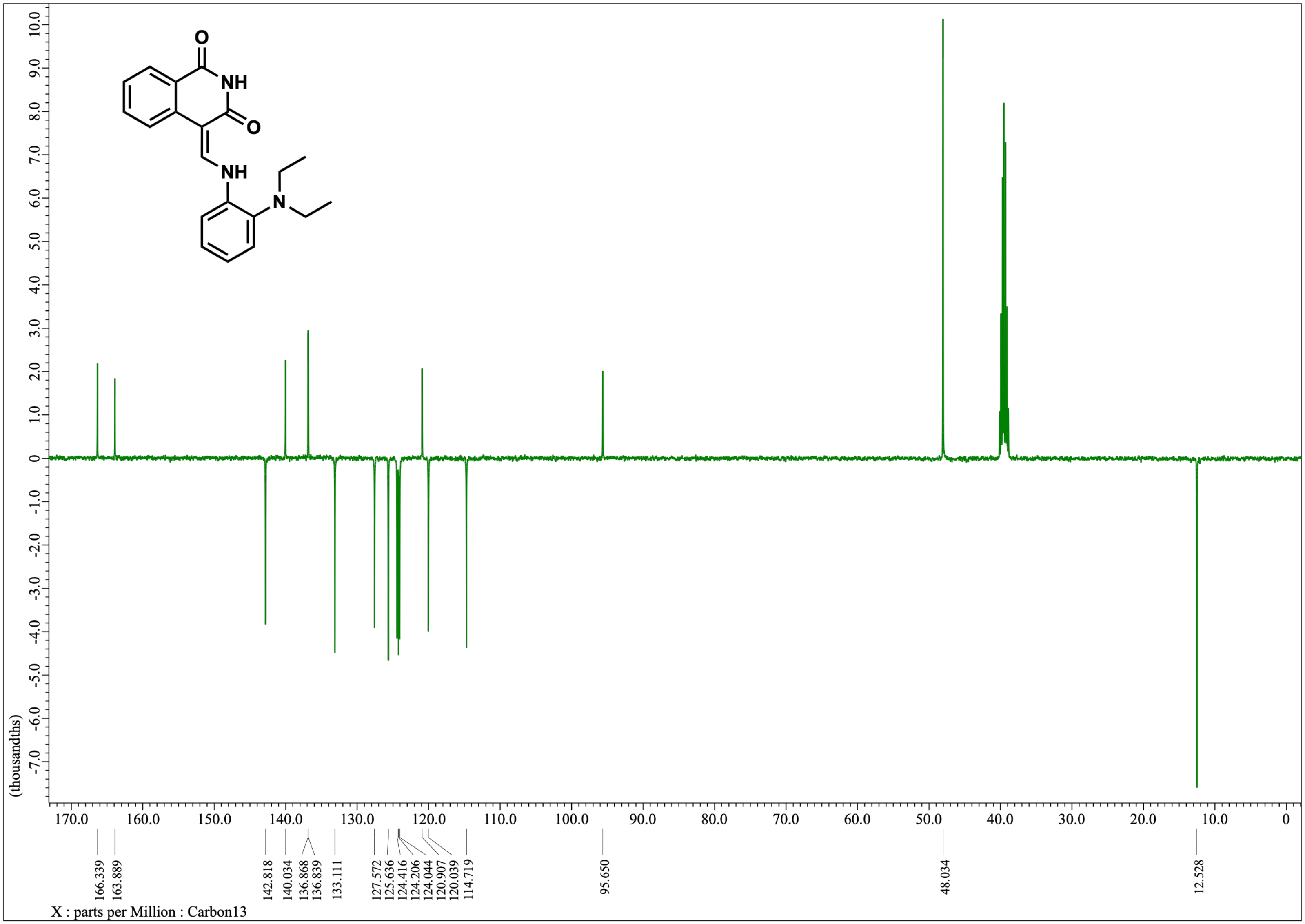

Figure S 80: ^{1}H-NMR spectra (500 MHz) of ICTR-063 in DMSO-d_6

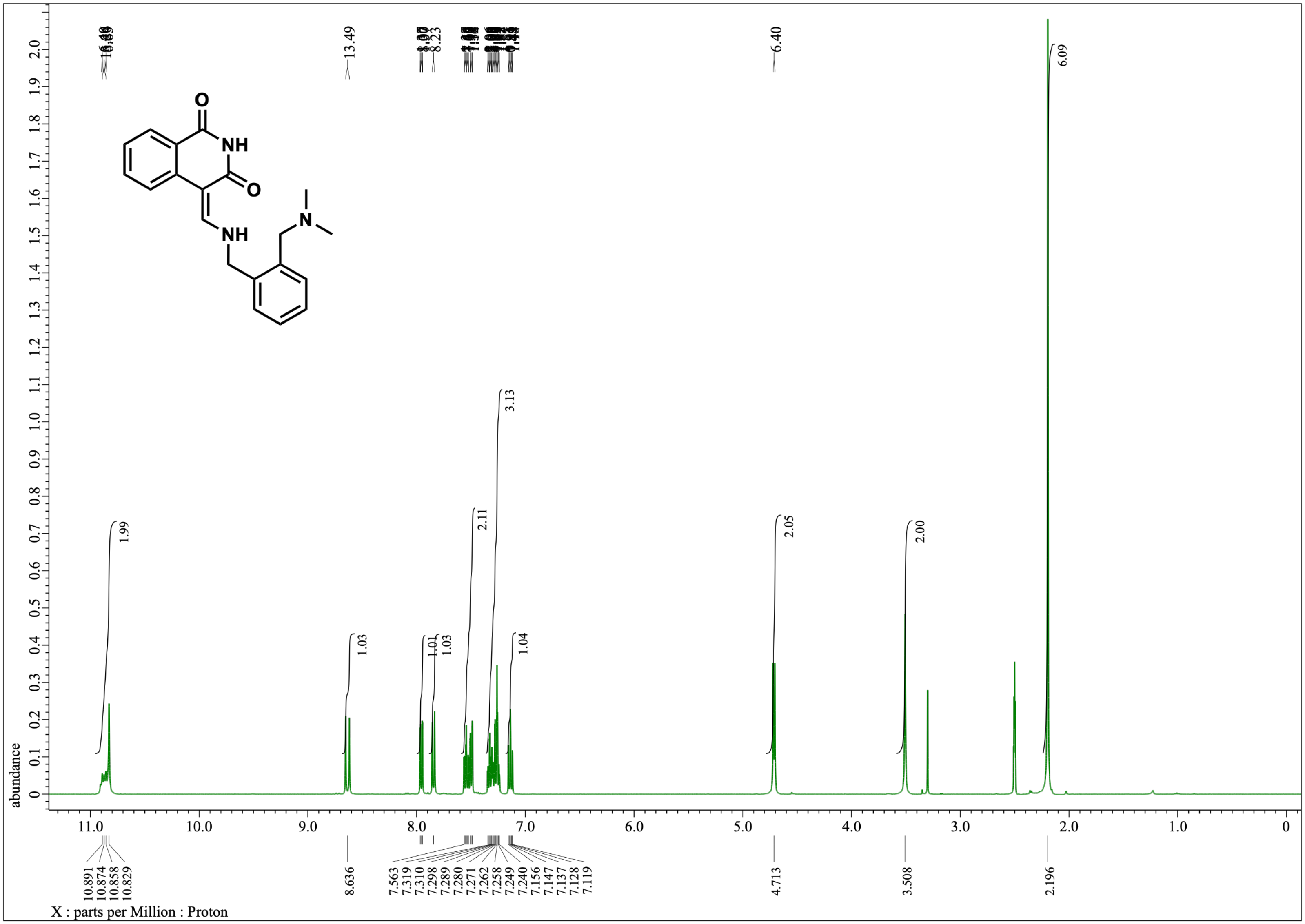

Figure S 81: ^{13}C-NMR (APT) spectra (100 MHz) of ICTR-063 in DMSO-d_6

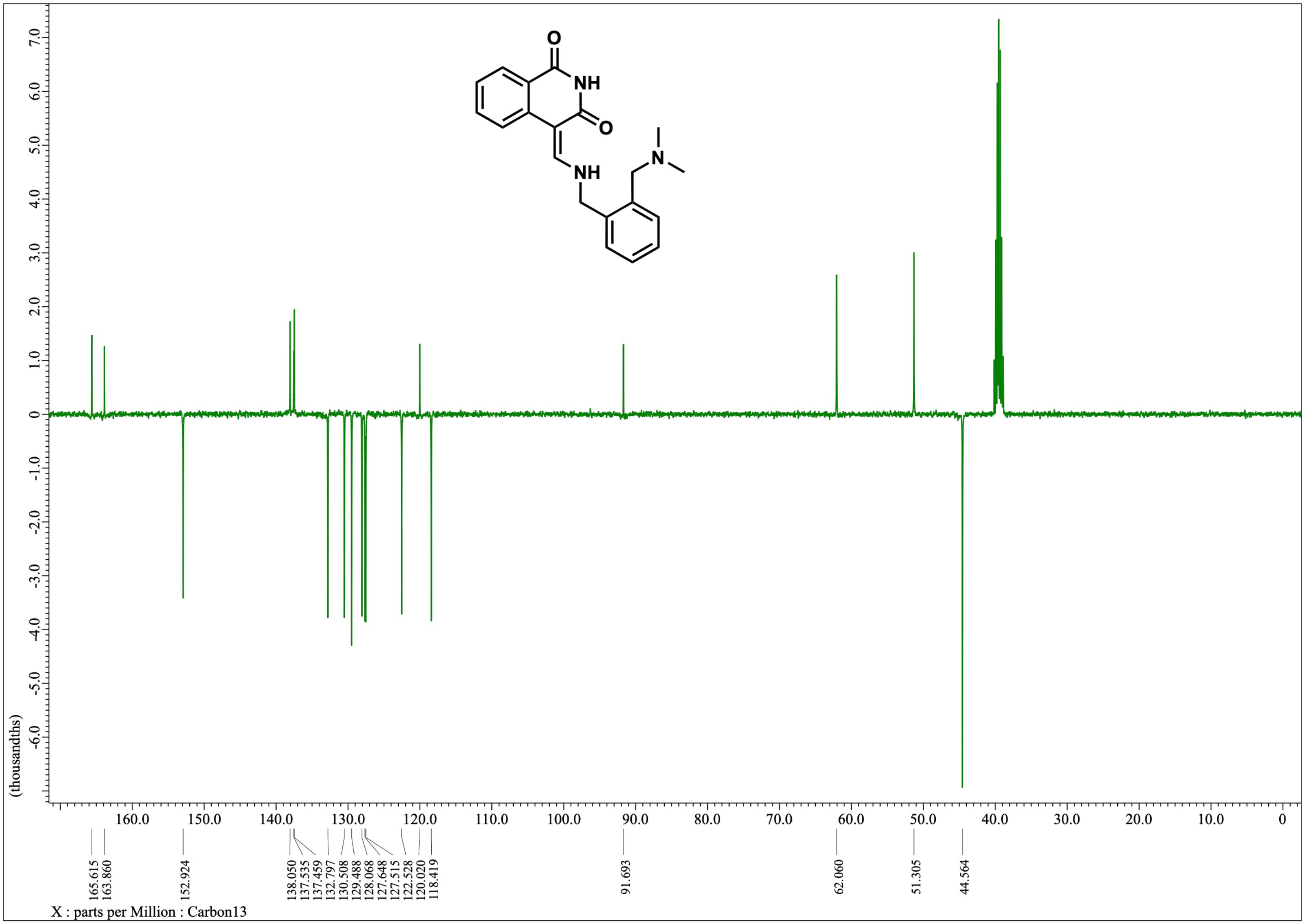

Figure S 82: ^{1}H-NMR spectra (500 MHz) of ICTR-076 in DMSO-d_6

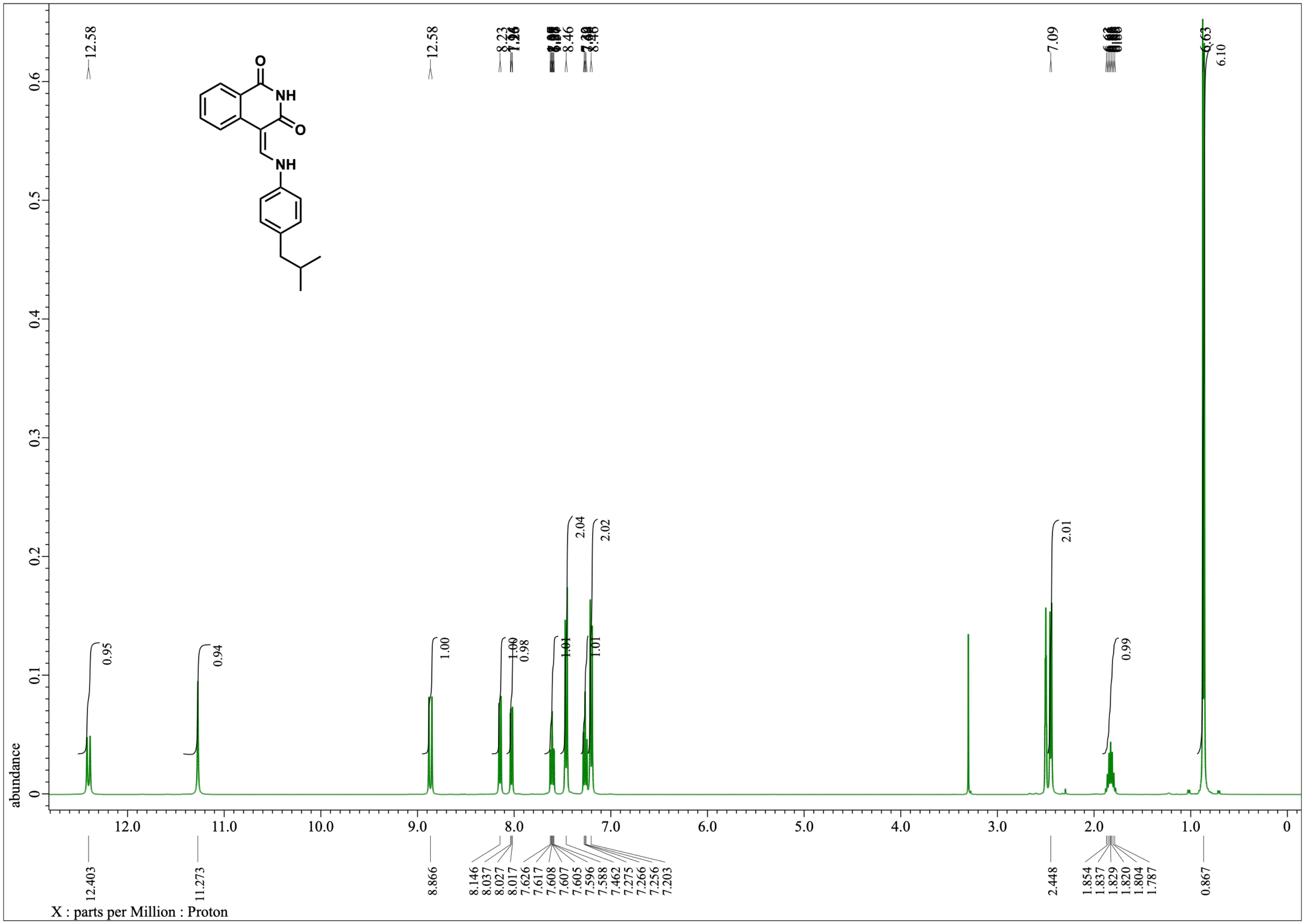

Figure S 83: ^{13}C-NMR (APT) spectra (100 MHz) of ICTR-076 in DMSO-d_6

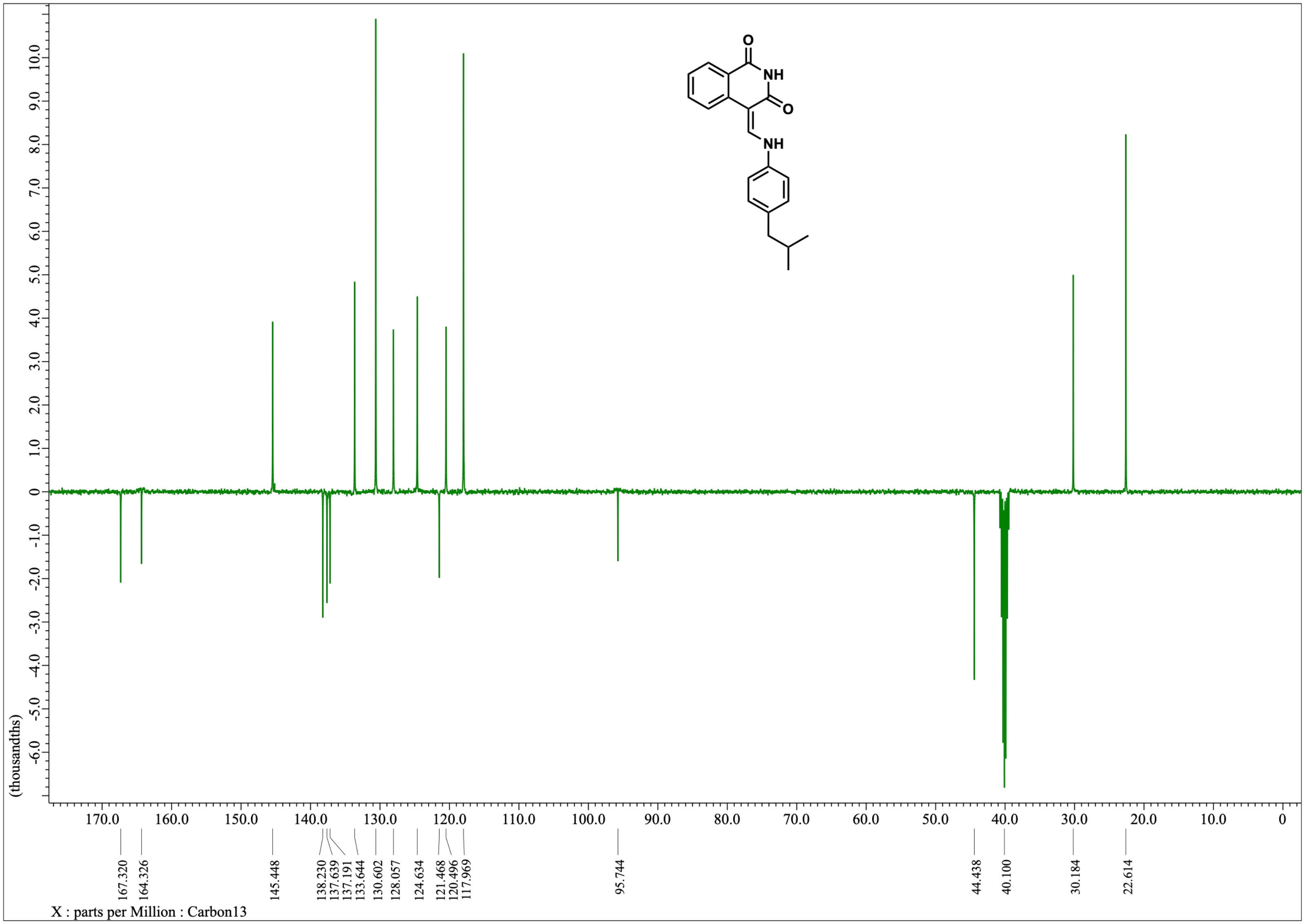

Figure S 84: ^{1}H-NMR spectra (500 MHz) of ICTR-078 in DMSO-d_6

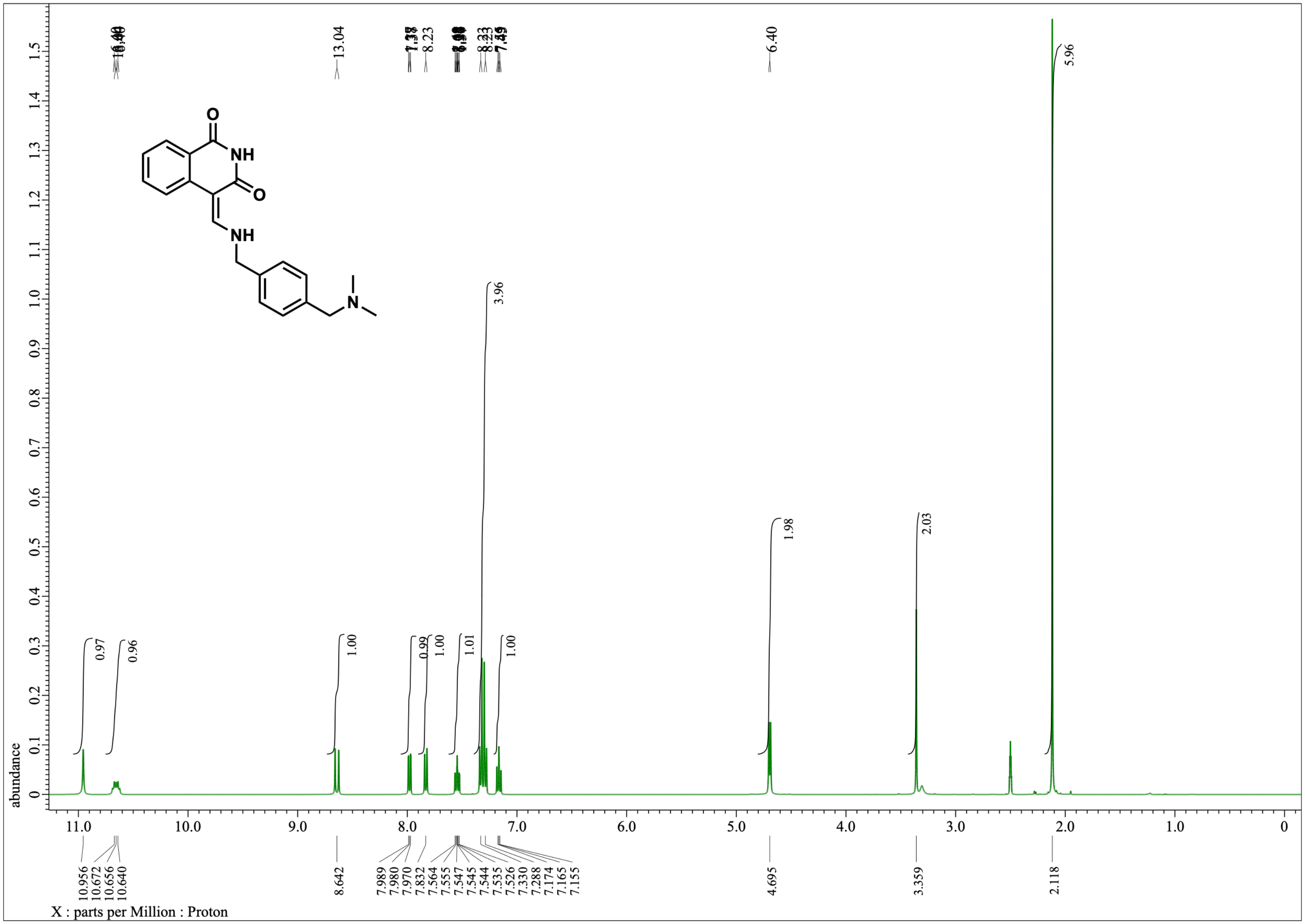

Figure S 85: ^{13}C-NMR (APT) spectra (100 MHz) of ICTR-078 in DMSO-d_6

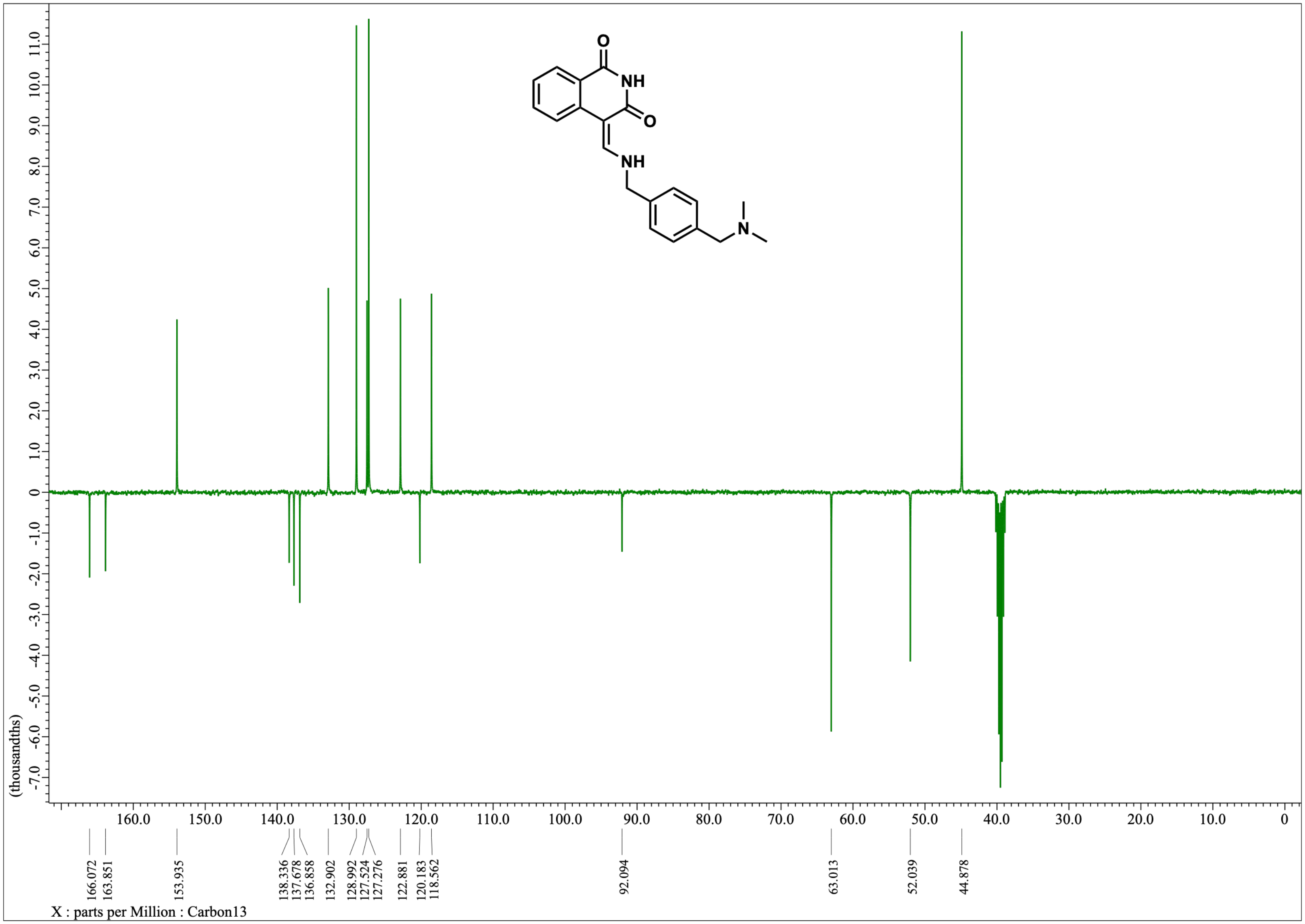

Figure S 86: ^{1}H-NMR spectra (500 MHz) of ICTR-086*HCl in DMSO-d_6

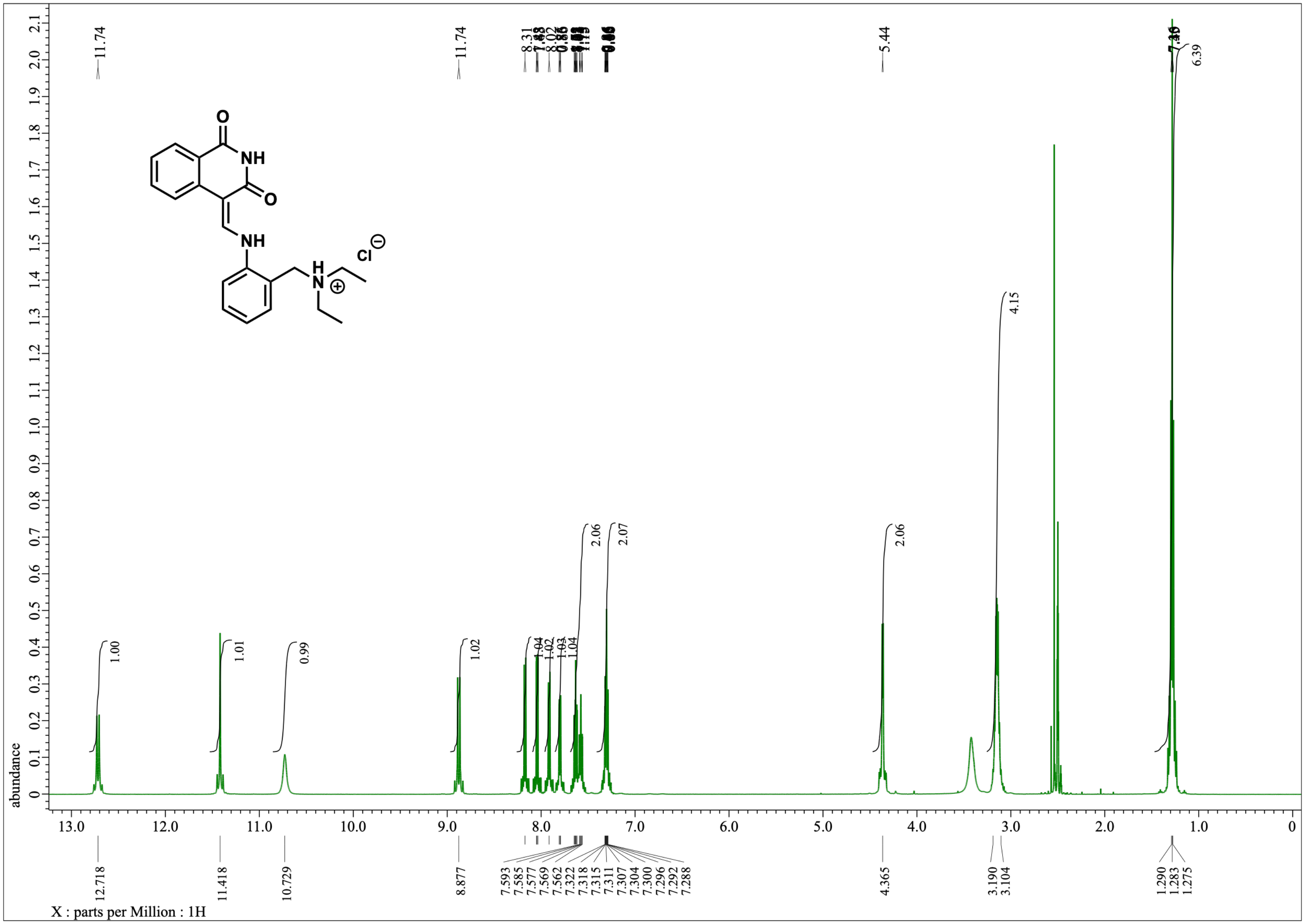

Figure S 87: ^{13}C-NMR (APT) spectra (125 MHz) of ICTR-086*HCl in DMSO-d_6

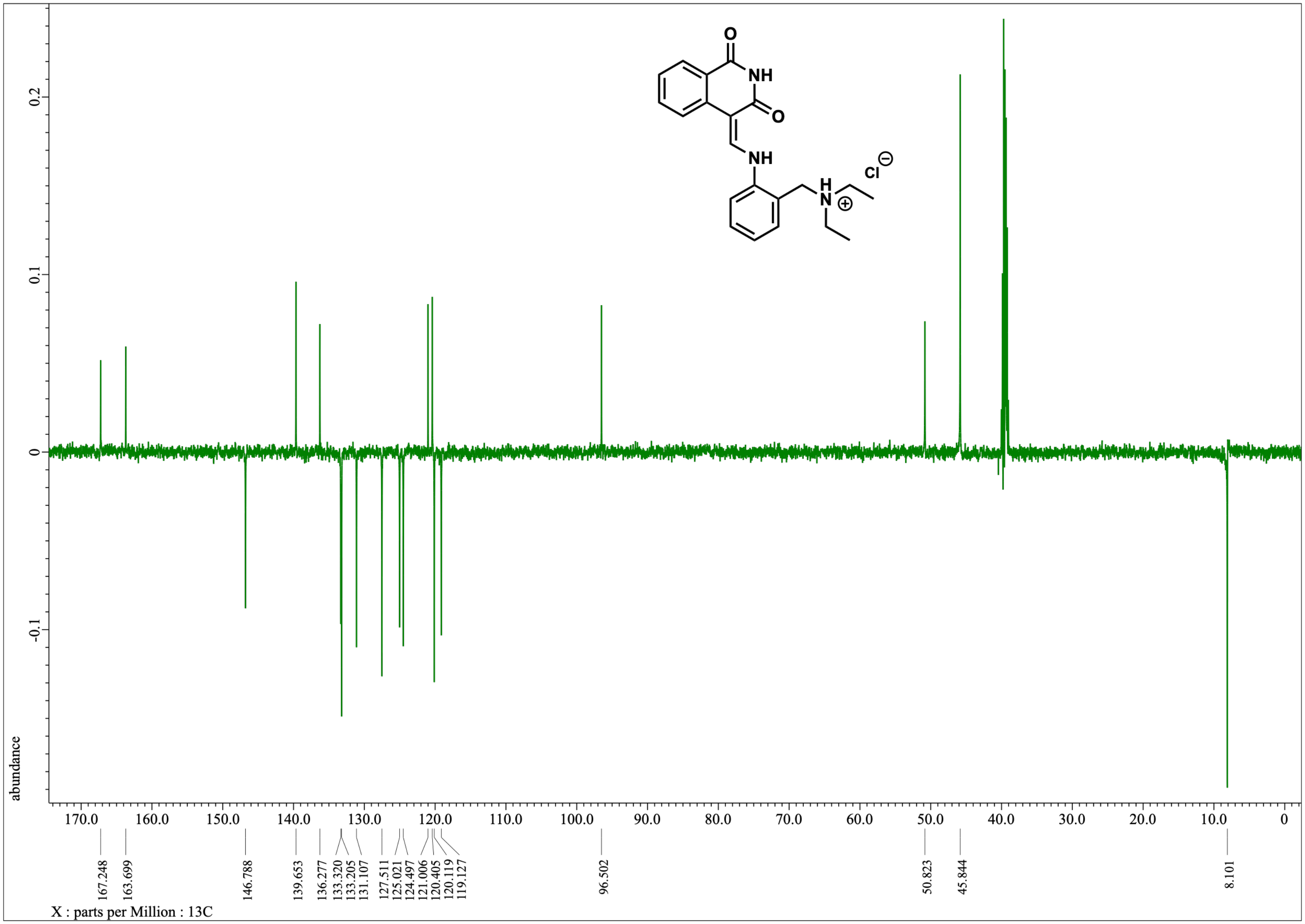

Figure S 88: ^{1}H-NMR spectra (500 MHz) of ICTR-094 in DMSO-*d₆*

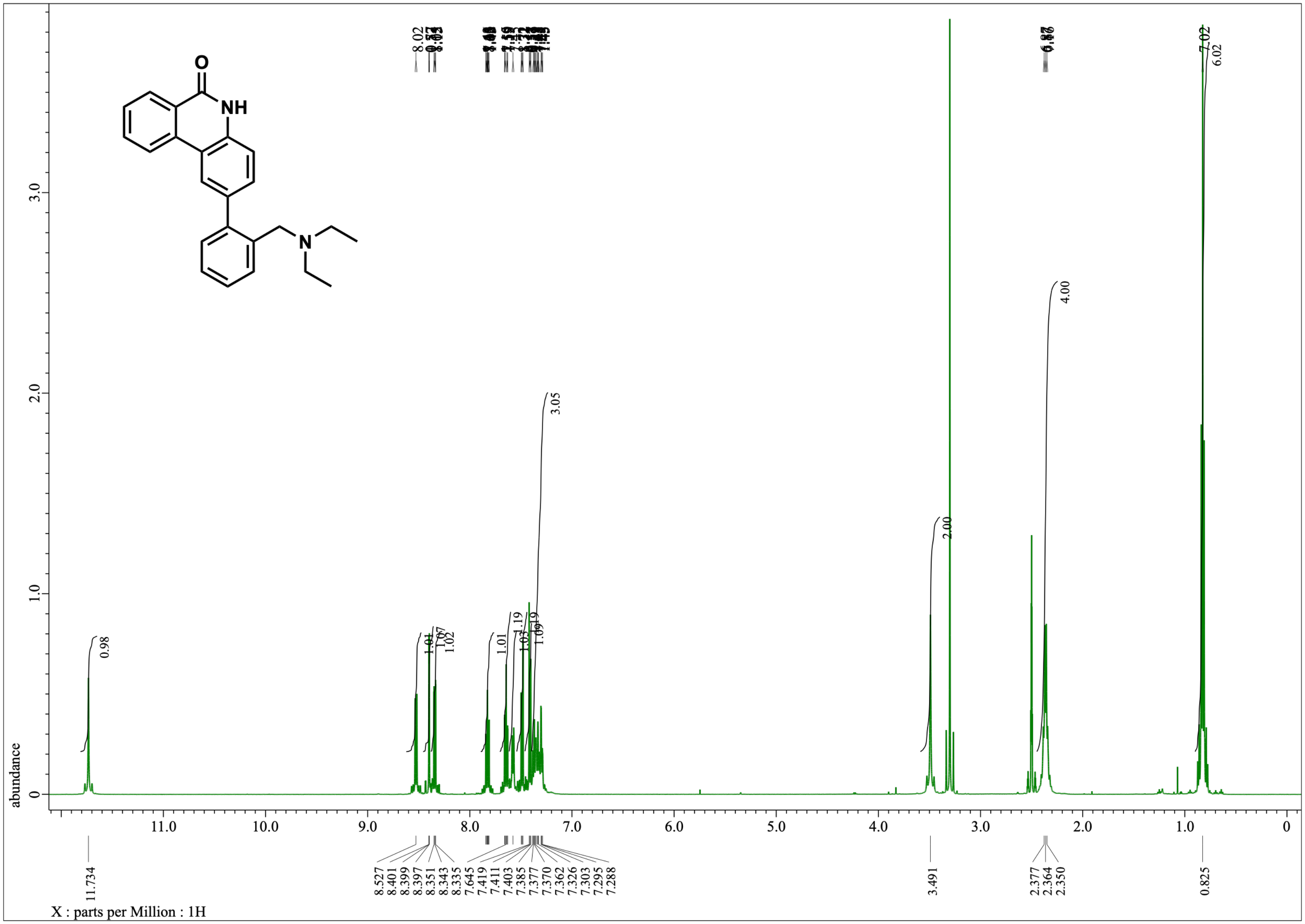

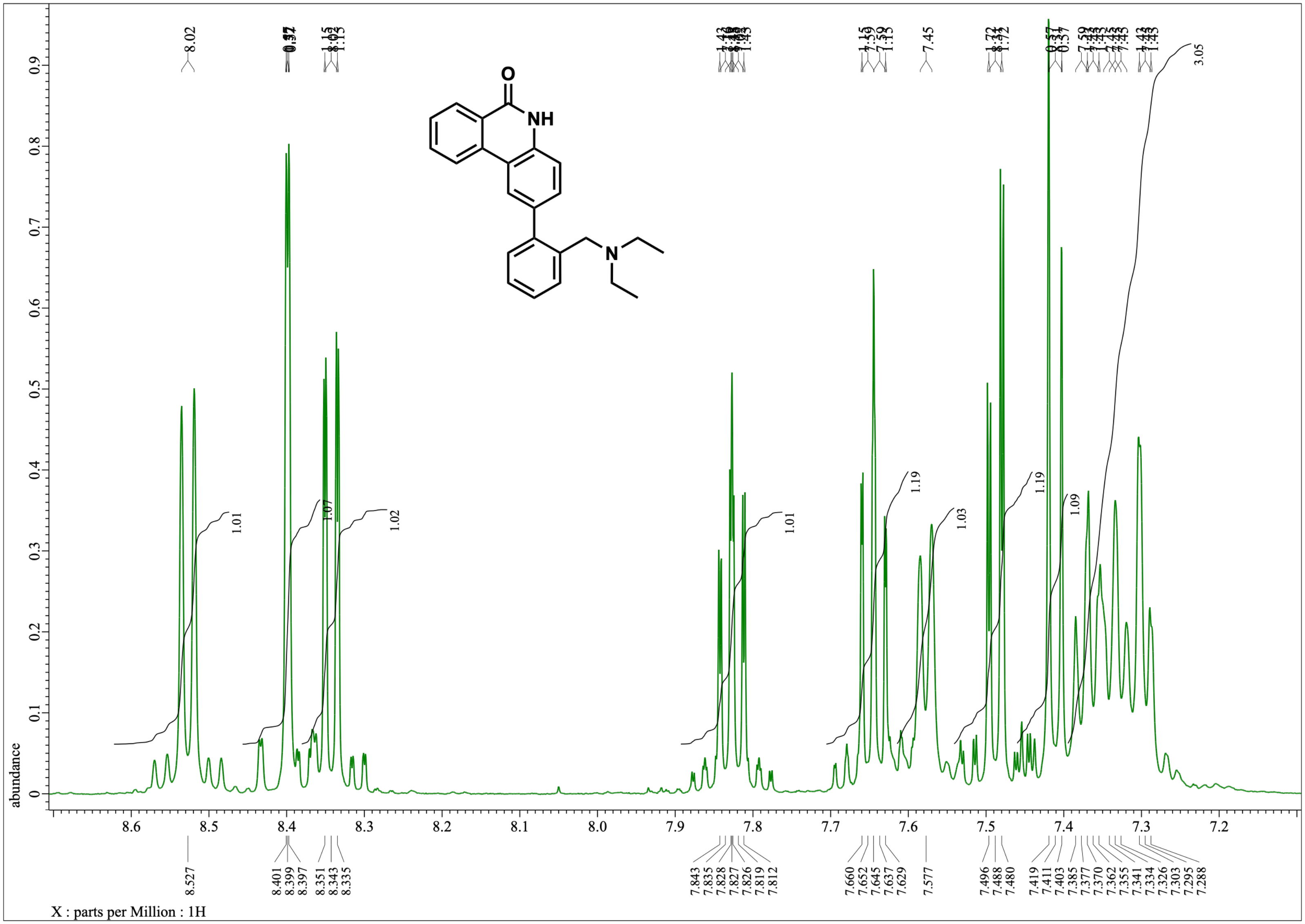

O
NH
N
abundance
X : parts per Million : 1H
8.527
8.401
8.399
8.397
8.351
8.343
8.335
7.843
7.835
7.828
7.827
7.826
7.819
7.812
7.660
7.652
7.645
7.637
7.629
7.577
7.496
7.488
7.480
7.419
7.411
7.403
7.385
7.377
7.370
7.362
7.355
7.341
7.334
7.326
7.303
7.295
7.288
1.01
1.07
1.02
1.01
1.19
1.03
1.19
1.09
3.05

Figure S 89: ^{13}C-NMR (APT) spectra (125 MHz) of ICTR-094 in DMSO-d_6

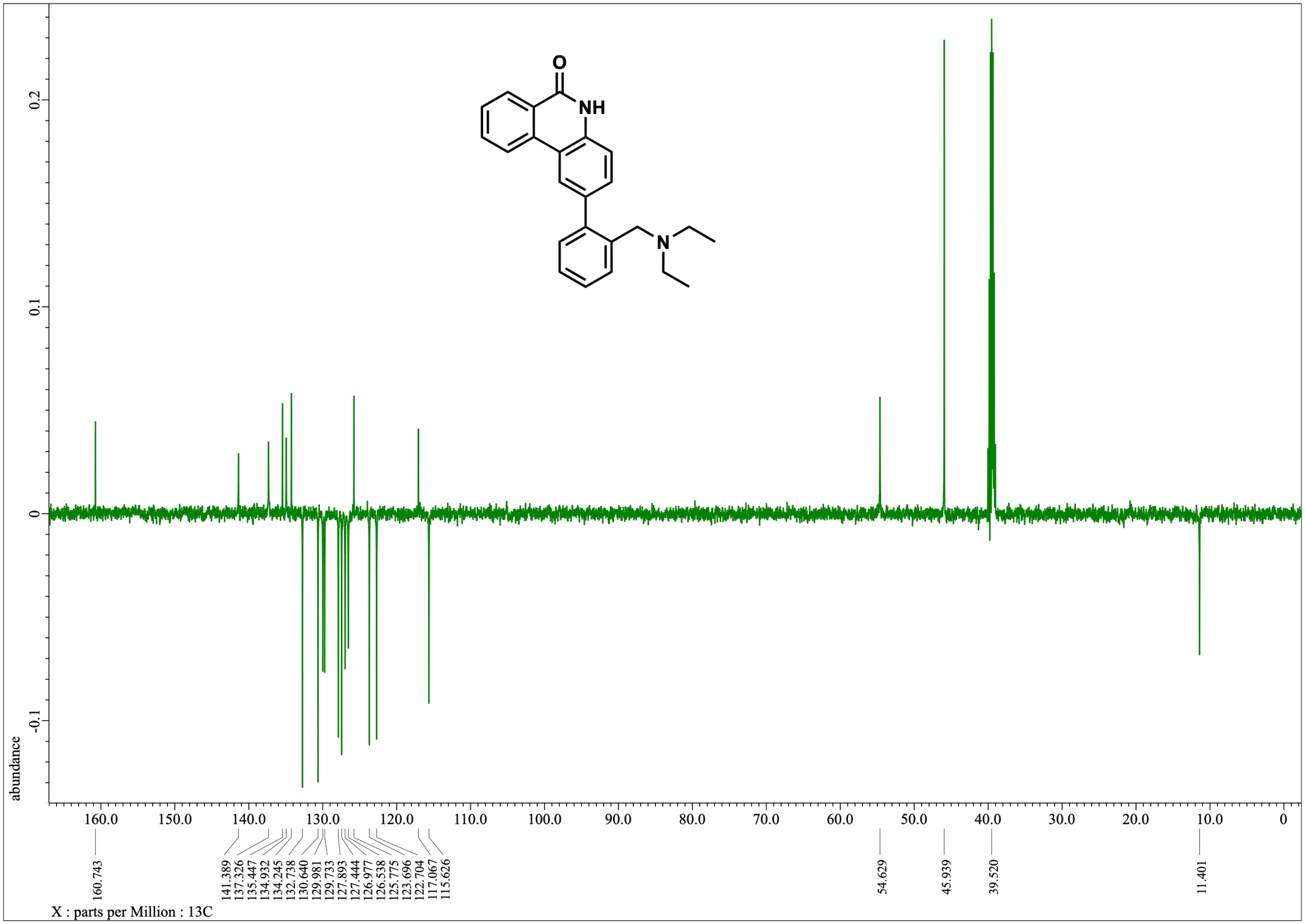

Figure S 90: ^{1}H-NMR spectra (500 MHz) of ICTR-096 in DMSO-d_6

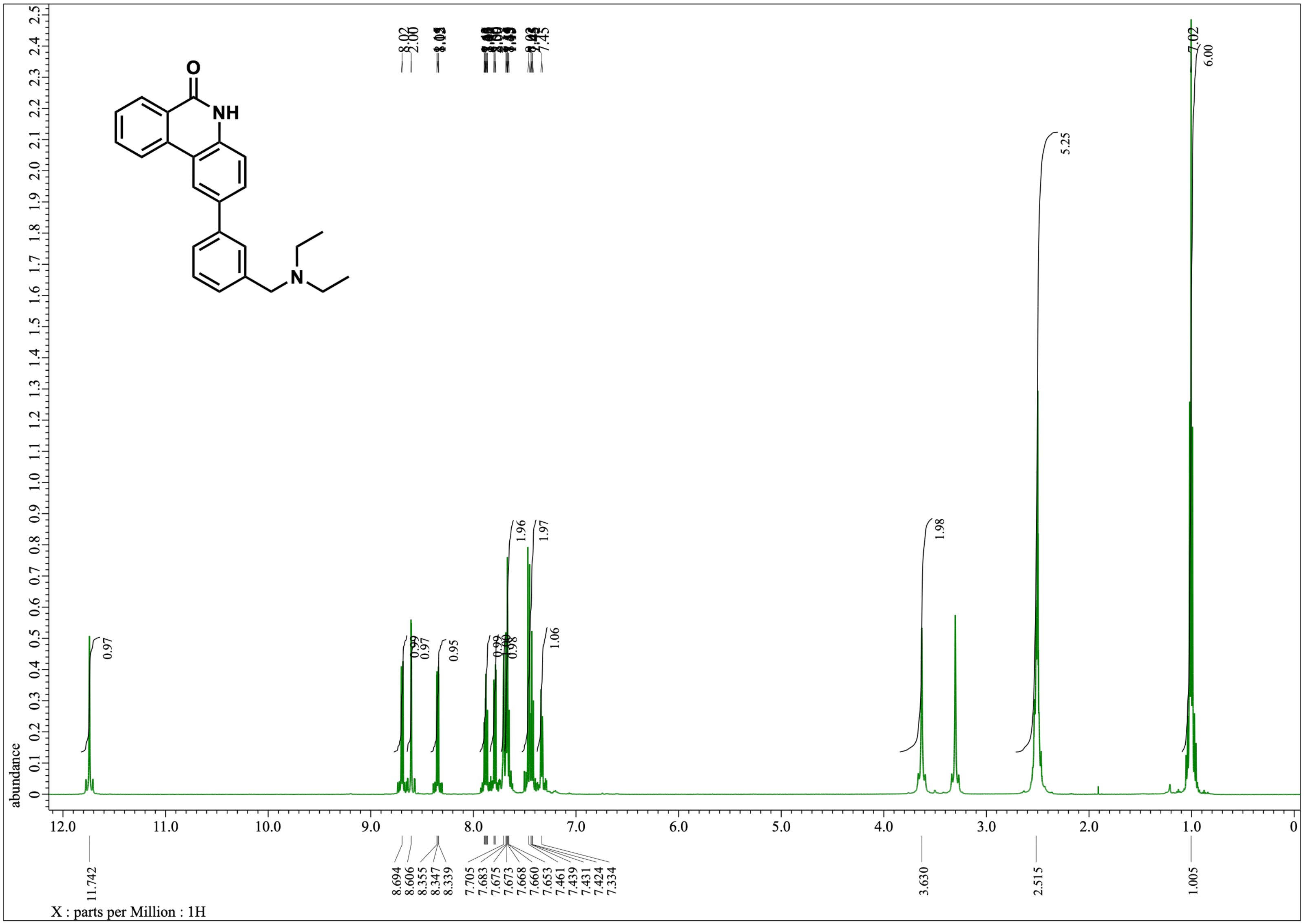

Figure S 91: ^{13}C-NMR (APT) spectra (125 MHz) of ICTR-096 in DMSO-d_6

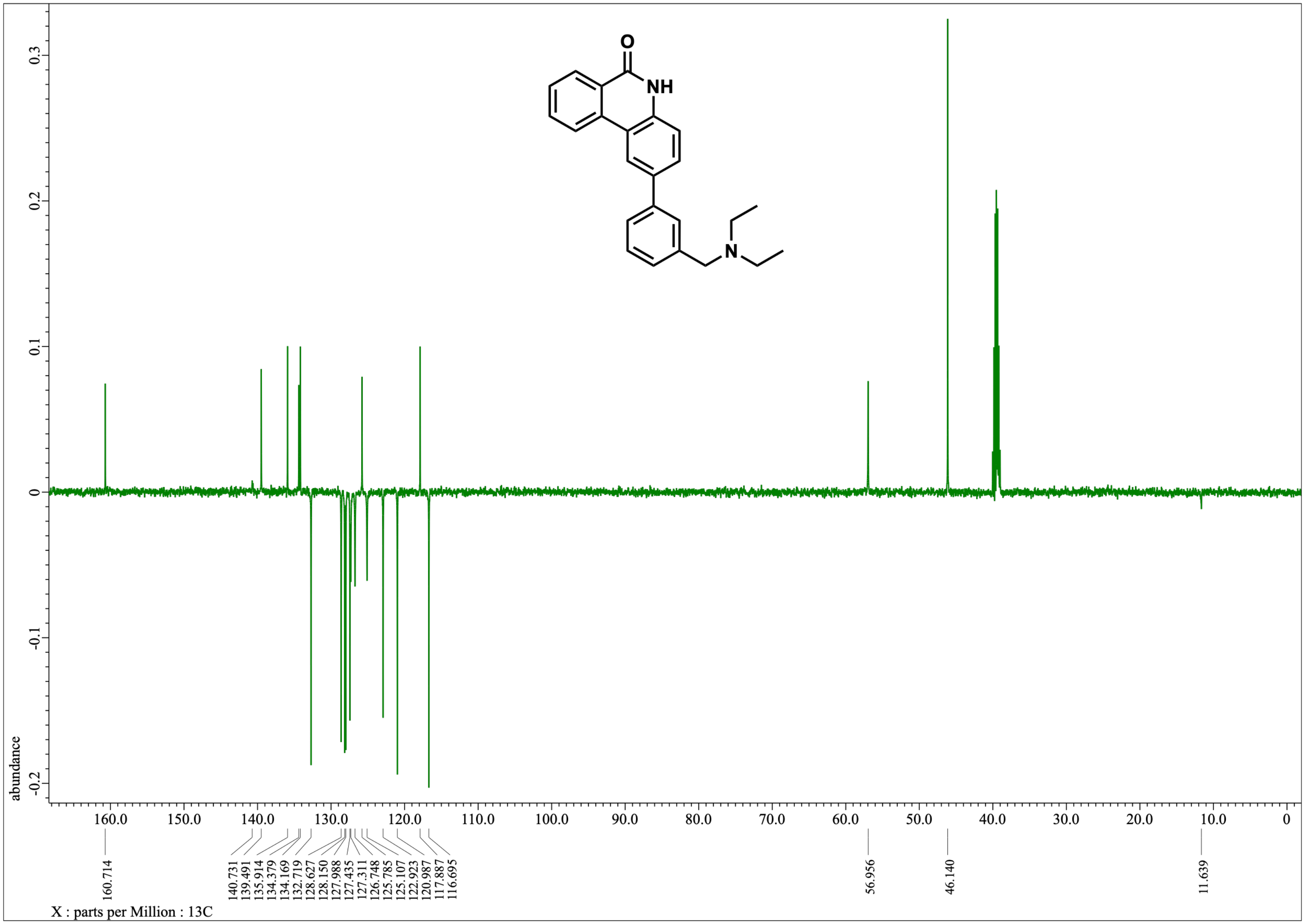

Figure S 92: ^{1}H-NMR spectra (500 MHz) of ICTR-108 in DMSO-d_6

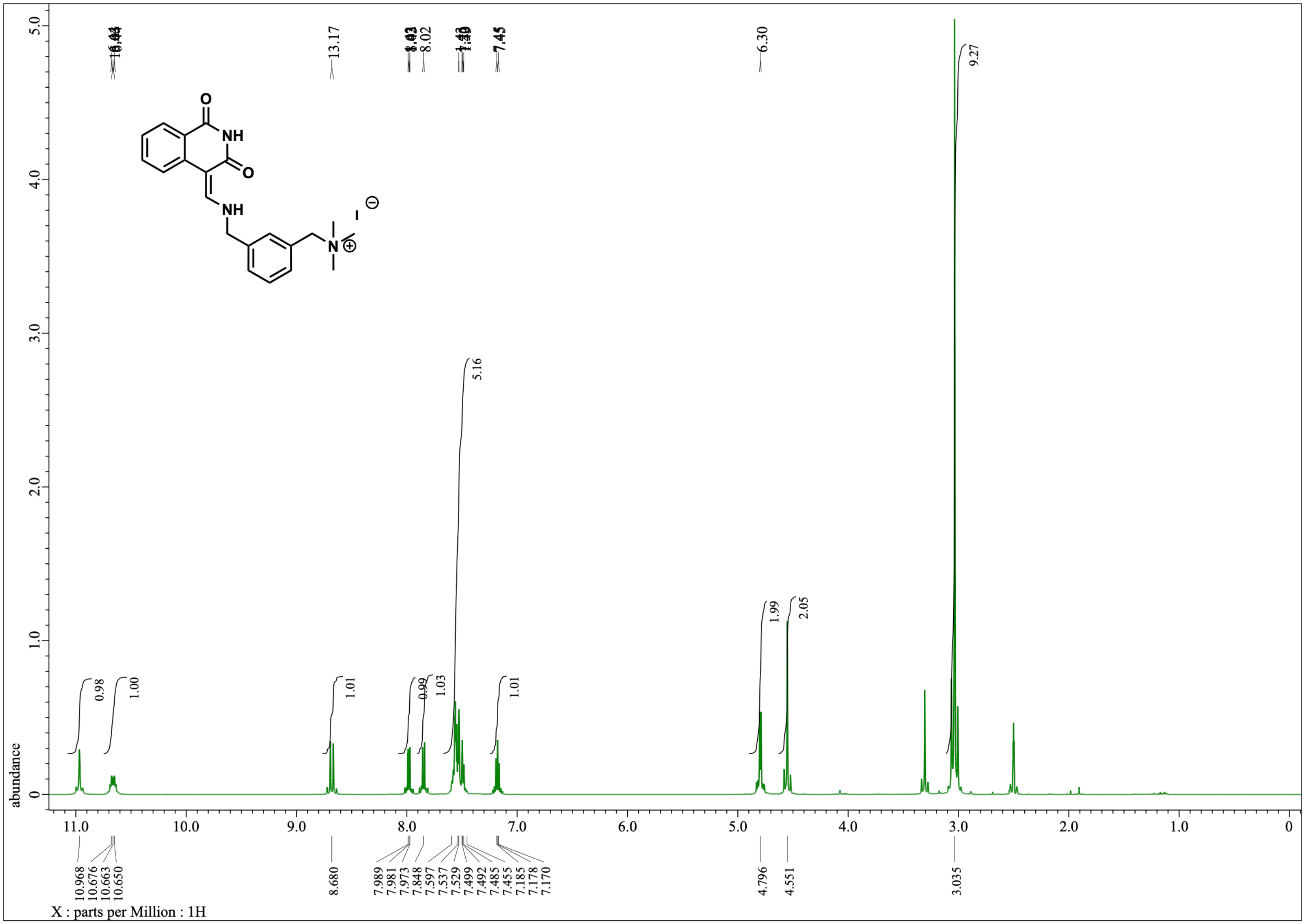

Figure S 93: ^{13}C-NMR (APT) spectra (125 MHz) of ICTR-108 in DMSO-d_6

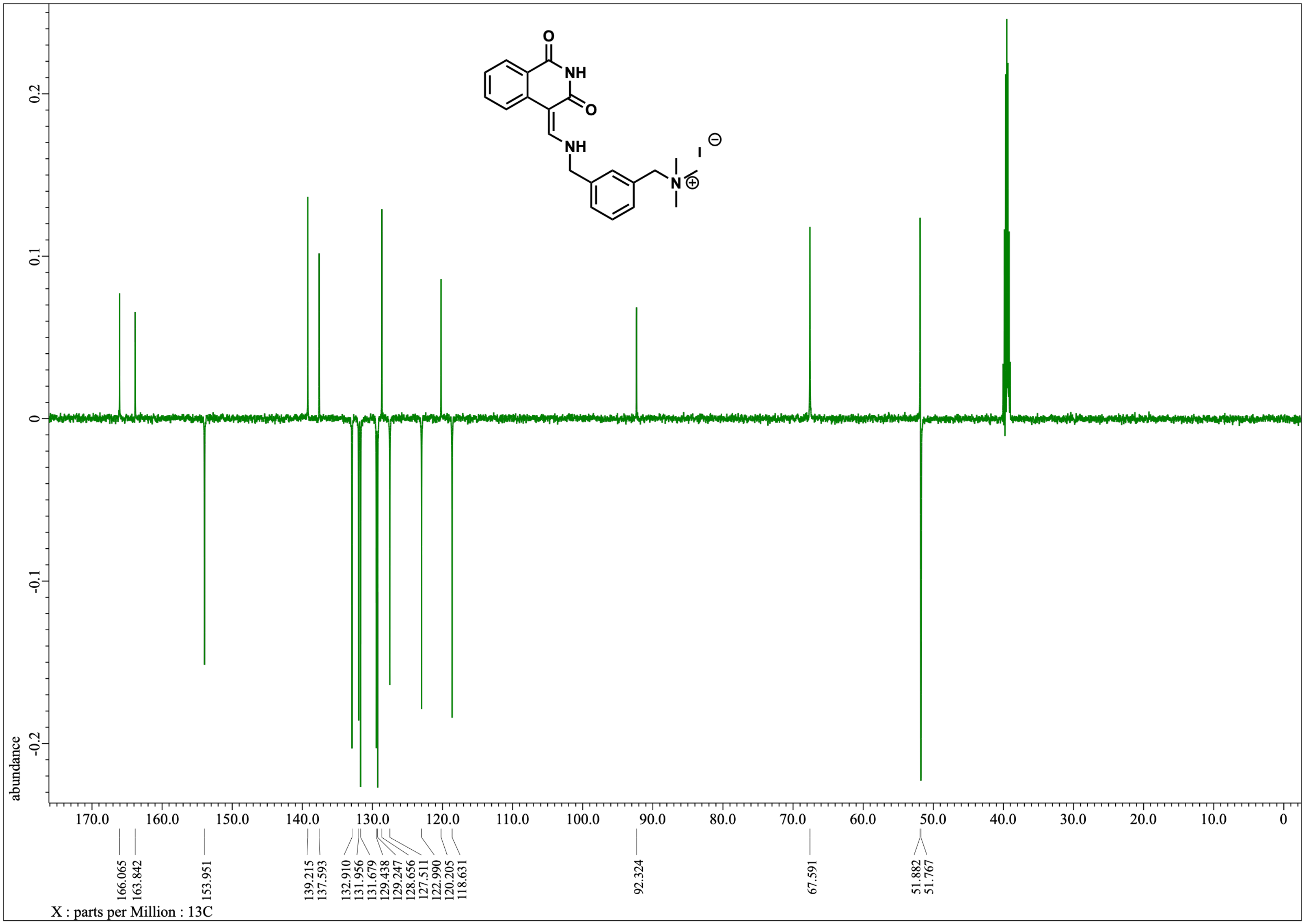

Figure S 94: ^{1}H-NMR spectra (500 MHz) of ICTR-119 in DMSO-d_6

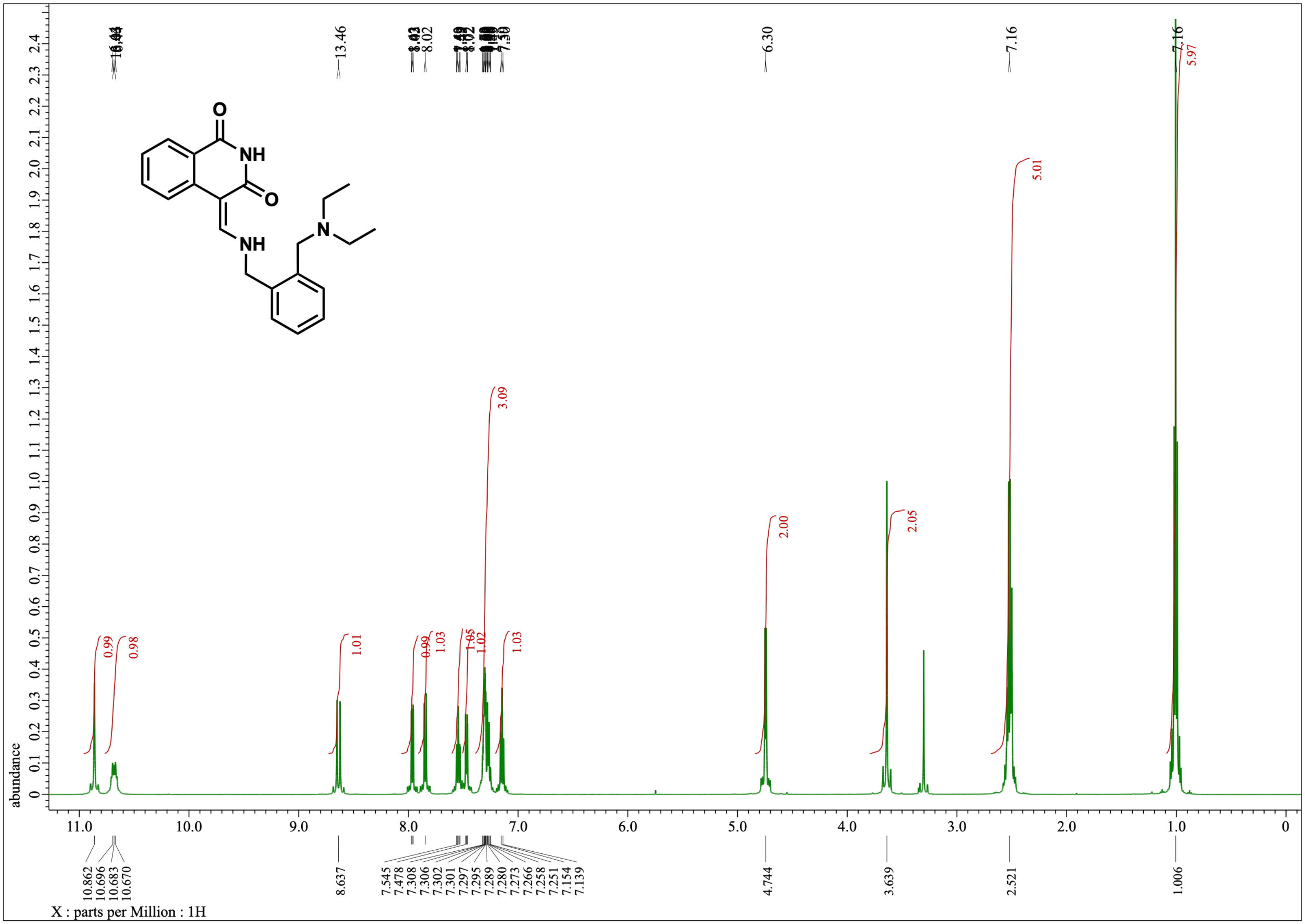

Figure S 95: ^{13}C-NMR (APT) spectra (125 MHz) of ICTR-119 in DMSO-d_6

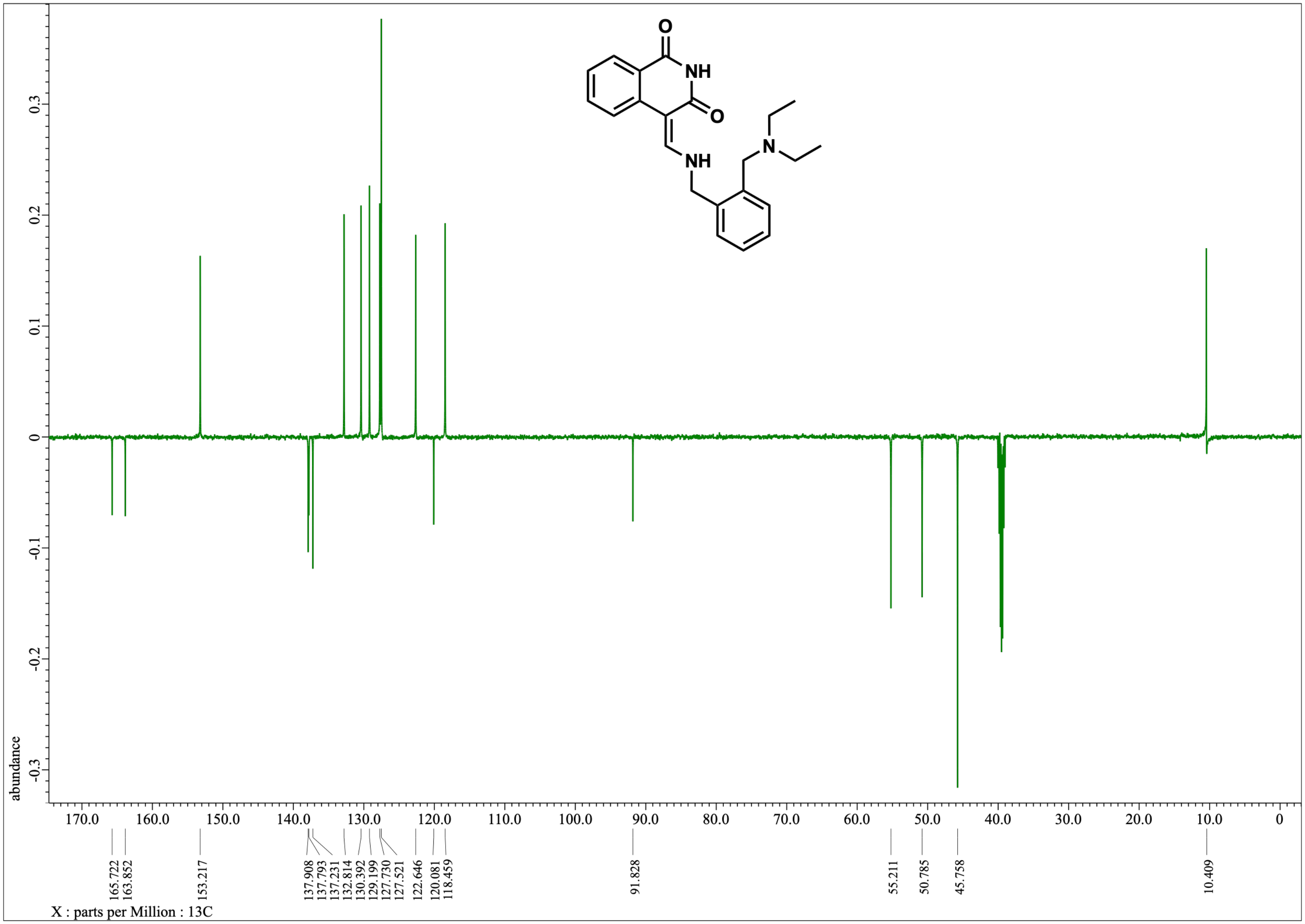

Figure S 96: ^{1}H-NMR spectra (500 MHz) of ICTR-120 in DMSO-d_6

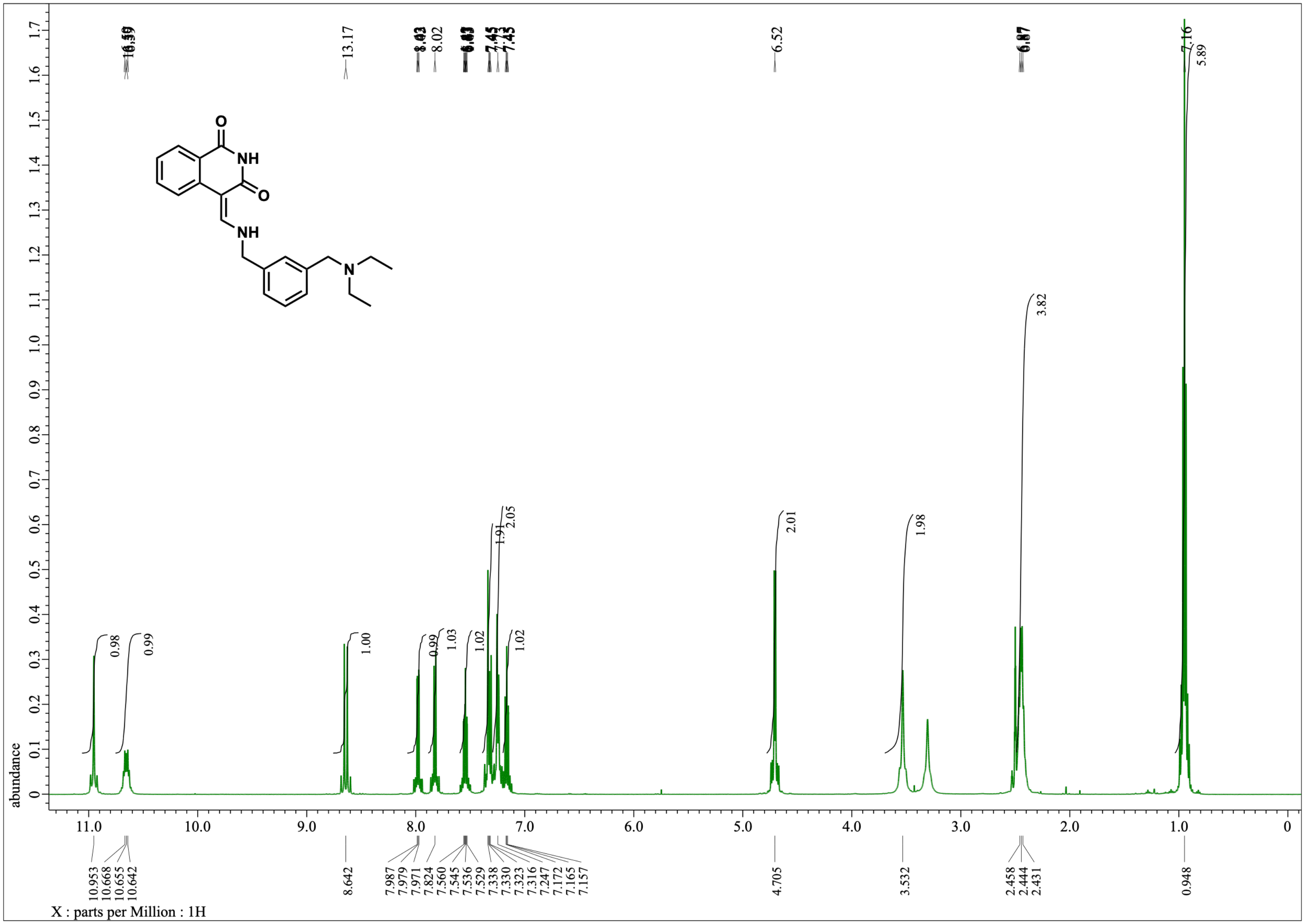

Figure S 97: ^{13}C-NMR (APT) spectra (125 MHz) of ICTR-120 in DMSO-d_6

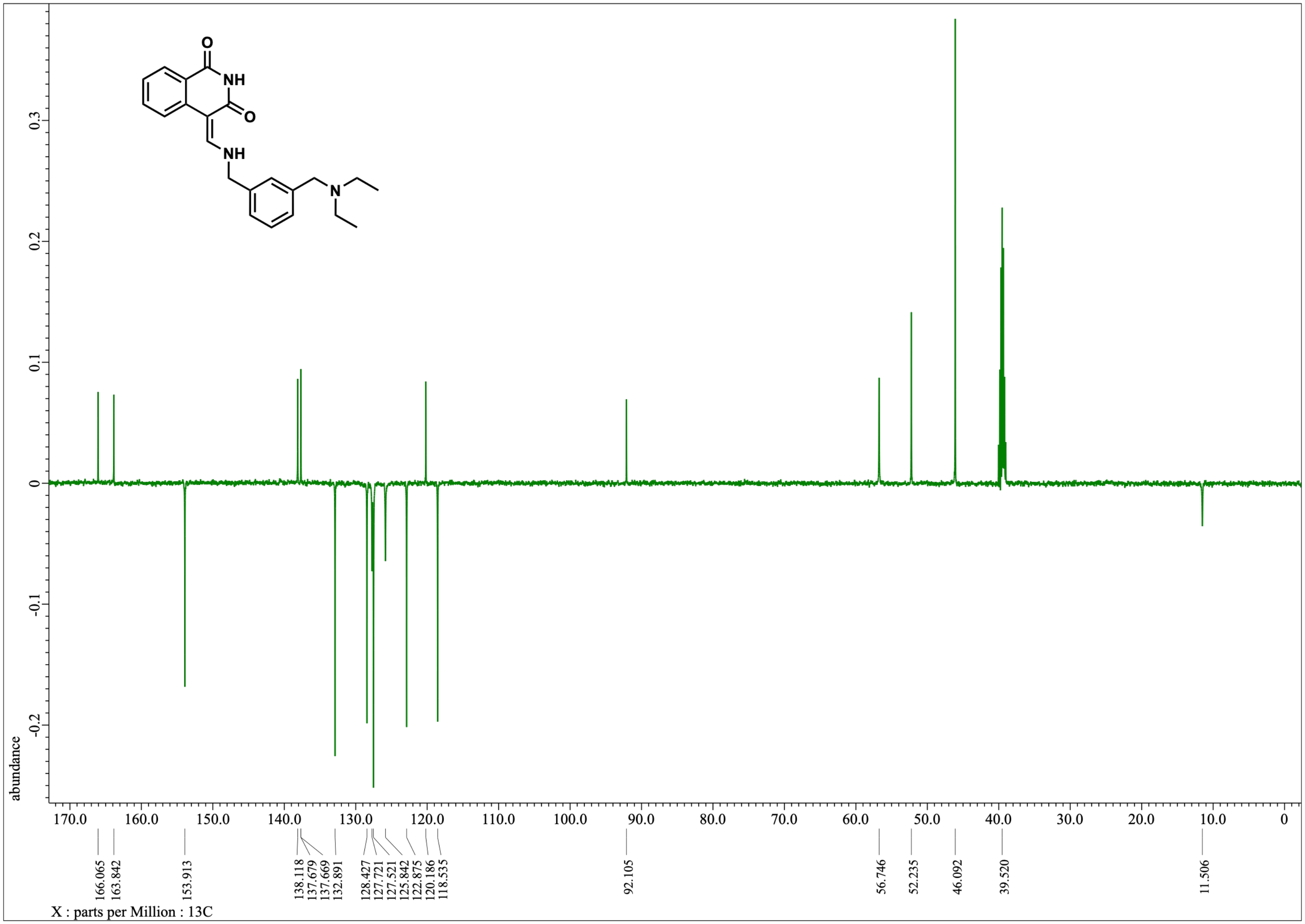

Supplement: Supplementary file 1 [file jm5c03281_si_004.pdf]
